# Expeditious diastereoselective synthesis of elaborated ketones via remote C*sp*^3^–H functionalization

**DOI:** 10.1038/ncomms13832

**Published:** 2017-01-13

**Authors:** Wei Shu, Adriana Lorente, Enrique Gómez-Bengoa, Cristina Nevado

**Affiliations:** 1 Department of Chemistry, University of Zürich, Winterthurerstrasse 190, CH-8057 Zürich, Switzerland; 2 Departamento de Química Orgánica I, Universidad del Pais Vasco, Apdo 1072, CP-20080 Donostia—San Sebastián, Spain

## Abstract

The quest for selective C–H functionalization reactions, able to provide new strategic opportunities for the rapid assembly of molecular complexity, represents a major focus of the chemical community. Examples of non-directed, remote C*sp*^3^–H activation to forge complex carbon frameworks remain scarce due to the kinetic stability and thus intrinsic challenge associated to the chemo-, regio- and stereoselective functionalization of aliphatic C–H bonds. Here we describe a radical-mediated, directing-group-free regioselective 1,5-hydrogen transfer of unactivated C*sp*^3^–H bonds followed by a second C*sp*^2^–H functionalization to produce, with exquisite stereoselectivity, a variety of elaborated fused ketones. This study demonstrates that aliphatic acids can be strategically harnessed as 1,2-diradical synthons and that secondary aliphatic C–H bonds can be engaged in stereoselective C–C bond-forming reactions, highlighting the potential of this protocol for target-oriented natural product and pharmaceutical synthesis.

## Abstract (web summary)

C-H activation is a powerful method to form functionalised molecules, but is particularly challenging for unactivated sp^3^ sites. Here the authors report a directing-group-free radical cascade process for converting vinyl azides and carboxylic acids to tetralone derivatives in high diastereoselectivity.

Strategies towards the efficient and selective activation of C–H bonds have been intensively explored in the context of chemical synthesis economy^1^. Direct C–H functionalizations are transformative as ubiquitous C–H bonds can be collected as functional group handles^2^,^3^ obviating the traditional requirement for functional group manipulation and exchange^4^. Multiple challenges still lay ahead, particularly in the case of C*sp*^3^–H bonds whose dissociation energy coupled with the energetic and spatial inaccessibility of the C–H bonding and antibonding orbitals makes them chemically inert (Fig. 1a1)^5^,^6^. To date, various methods for C*sp*^3^–H functionalization using transition metal catalysts have been developed, including directing group assisted C–H metalation (Fig. 1a2)^7^,^8^,^9^,^10^,^11^, and metal carbenoid or nitrenoid facilitated C–H insertion reactions via concerted^12^,^13^,^14^ or single-electron transfer processes (Fig. 1a3)^15^,^16^. Despite this broad array of methods, the field still faces significant challenges including the limited ability to discriminate between individual 3°, 2° or 1° C–H bonds, as well as the modest levels of stereocontrol commonly achieved in these transformations^17^. Nature seems to have solved these caveats with highly evolved enzymes that possess tridimensionally complex binding sites^18^. Small-molecule catalysts are not yet broadly applicable in highly functionalized chemical blueprints and thus, despite notable exceptions^19^,^20^, ambitious targets still remain far from straightforward reach via current C*sp*^3^–H functionalization methods. Radical-centred C–H functionalizations represent an alternative yet distinct option to activate isolated, aliphatic C–H bonds via an H-atom abstraction mechanism as seminally exemplified by the Hofmann–Löffler–Freytag reaction (Fig. 1a4)^21^,^22^. In this context, C*sp*^3^–H functionalizations using 1,n-H shifts rely on pre-performed radical precursors such as C*sp*^2^–halide bonds, azides, amidines and so on^23^,^24^,^25^,^26^,^27^,^28^, but due to the highly reactive nature of the free radical species involved, reaction control in terms of stereo- and site-selectivity remains challenging and thus applications in complex settings have been scarce.

Ketones are regarded as privileged functional groups from the viewpoint of their utility^29^ and ubiquity in a wide variety of biologically active natural products and pharmaceutical agents (Fig. 1b)^30^,^31^,^32^,^33^. Classical approaches to introduce ketones in complex scaffolds heavily rely on direct oxidation/reduction of other functional groups, including alcohols, alkenes, alkynes, carbonyl derivatives and nitriles. These protocols require time-consuming and labour-intensive processes to install the corresponding precursors and to pre-synthesize the original C-frameworks.

Here we present the straightforward preparation of complex ketones in a stereocontrolled manner capitalizing on the remote functionalization of C*sp*^3^–H bonds. We hypothesized that aliphatic carboxylic acids could be collected as 1,2-diradical synthons in the presence of vinyl azides^34^,^35^,^36^,^37^, through a radical-mediated decarboxylation process^38^,^39^. Notably, the reaction involves a regioselective, directing-group free activation of an unactivated aliphatic C–H bond via radical-mediated 1,5-hydrogen transfer and a C*sp*^2^–H functionalization relay via Minisci-type reaction^40^, a combination of steps thus far not reported in the literature (Fig. 1c). Secondary aliphatic C–H bonds, arguably the most difficult to oxidize selectively because of their bond strength, ubiquity and steric hindrance, can be engaged in a stereoselective C–C bond-forming unique cascade reaction that entitles the formation of two new C–C and one C=O bond streamlining the construction of fused ketone scaffolds present in a variety of bioactive molecules.

## Results

### Optimization of the reaction conditions

1-Methylcyclohexane-1-carboxylic acid and (1-azidovinyl)-benzene were selected as benchmark substrates to find the optimal reaction conditions. A preliminary screening confirmed the ability of catalytic amounts of silver in combination with a stoichiometric amount of oxidant to promote a radical decarboxylation process^41^,^42^. The substrates, combined in the presence of AgNO_3_ and K_2_S_2_O_8_ in acetonitrile for 10 h at 50 °C furnished the desired product (**1a**) in 5% yield (Table 1, entry 1). Other solvents were used with similar results (Table 1, entries 2 and 3). A more soluble silver salt such as Ag_2_CO_3_ was sought, which produced the desired product in 22% yield with acetone as solvent (Table 1, entry 4). The use of acetonitrile as a co-solvent in a 3:1 ratio, furnished **1a** in a promising 53% yield, likely due to the improved solubility of all reaction components. Replacing acetonitrile with dimethylformamide or dichloromethane proved not to be beneficial (Table 1, entries 5–7). In sharp contrast, a change in the composition of the solvent mixture (acetone: CH_3_CN, 2.5:1) produced **1a** in 58% yield (Table 1, entry 8). Basic additives (1 equivalent) were explored next revealing the positive effect of 2,6-lutidine in the reaction outcome (Table 1, entries 9–11). Finally, adjusting the amount of base to 1.2 equivalent furnished **1a** in an optimized 69% isolated yield (Table 1, entry 12). It is important to remark that, in all cases, compound **1a** was the only diastereoisomer detected in the crude reaction mixture.

### Substrate scope of the reaction

With the optimized reaction conditions in hand, we set out to explore the scope of this transformation. To this end, the substitution pattern on the aromatic ring of the vinyl azide was evaluated first (Table 2). The presence of electron withdrawing groups in *para* position to the vinyl azide moiety proved to be amenable to the standard reaction conditions as demonstrated by the efficiency of the reactions producing ketones **1b–d**. The presence of fluorine, chlorine or bromine atoms at this position was also well accommodated as **1e–g** could be isolated in good yields. These examples also showcase the functional group compatibility of this transformation as none of C*sp*^2^–F, –Cl, –Br bonds seems to affect (or interfere with) the desired reaction. Synthetically useful yields were also obtained with substrates bearing electron-donating groups at the *para* position (**1h**,**i**). An *ortho*-fluorobenzene vinyl azide could be efficiently engaged in this reaction, furnishing **1j** in 72% yield. 3-Fluoro, 3,4-difluoro, 3-methyl and 3-methoxy substituted substrates produced 3,4-dihydronaphthalen-1(2H)-ones **1k–n** in good yields with moderate *ortho*- regioselectivities. In contrast, 3-trifluoromethyl and 3-tertbutyl substituted substrates favoured the *para*-cyclized adducts **1o**,**p** with improved 6:1 and >20:1 ratio, respectively. These results clearly indicate that the regioselectivity is dictated by both the steric and electronic nature of the *meta*-substituents in the starting material. A quinoline derivative could also be selectively incorporated as demonstrated by the successful reaction to produce **1q**. X-ray diffraction analysis of **1a′** (obtained after reduction of the carbonyl group in **1a** and *p-*NO_2_ benzoylation of the secondary alcohol) confirmed the structural assignment of the reaction products and the *syn* relative configuration of the only diastereoisomer observed in these transformations.

**Figure 1:**
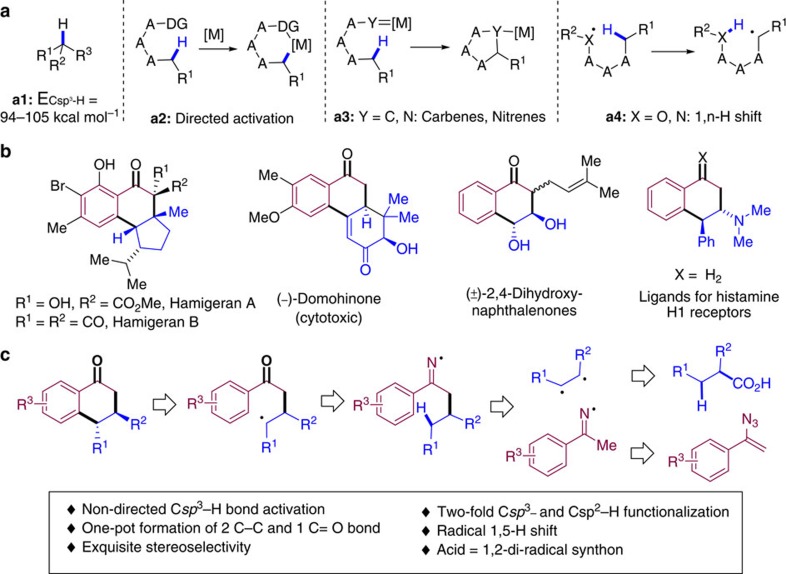
Significance and rational design of the reaction. (**a**) Bond dissociation energy of saturated C–H bonds. Strategies for C*sp*^3^–H activation. (**b**) Examples of elaborated fused ketones (and derivatives thereof) in bioactive molecules. (**c**) This work: stereoselective synthesis of elaborated ketones via space-enabled 1,5-H shift cascade.

Different aliphatic acids were studied next, and the results of these transformations have been summarized in Table 3. Five- and seven-membered tertiary carboxylic acids could be easily incorporated as demonstrated by the efficient transformations producing compounds **2a–c**. The reaction furnishing **2a** represents a straightforward route to the core structure of Hamigerans A and B, secondary metabolites with promising cytotoxic as well as potent antiviral activities (Fig. 1b)^30^,^43^. A tetrahydropyrane derivative (**2d**) could also be efficiently obtained in 56% yield. Acyclic substrates proved to be highly efficient partners in these transformations as well. Homobenzylic carboxylic acids bearing both electron-donating as well as electron-withdrawing groups could be efficiently coupled as demonstrated by the transformations producing **2e–j**. Fully aliphatic acyclic starting materials were also amenable to the reported conditions as shown by the reactions yielding ketones **2k**,**l**. Secondary carboxylic acids were also evaluated. A 2-tetrahydronaphthyl derivative produced the desired hexahydrochrysene-based ketone **2m** in synthetically useful yield, whereas β,γ-disubstituted 3,4-dihydronaphthalen-1(2H)-ones **2n–p** could be isolated in moderate to good yields as single diastereoisomers. The reaction protocol is also compatible with amino acids so that phenylalanine derivative **2q** could be isolated in 53% yield. Both benzofurane and quinoline derivatives proved to be amenable to the standard reaction conditions in the presence of 2,2-dimethyl-3-phenylpropanoic acid, delivering tricyclic adducts **2r** and **2s**, respectively. X-ray diffraction analysis of **2n** and **2s** confirmed the structural assignment of the reaction products and the *trans* relative configuration of the only diastereoisomer observed in the reaction of secondary acyclic substrates.

### Synthetic application

**Figure 2:**
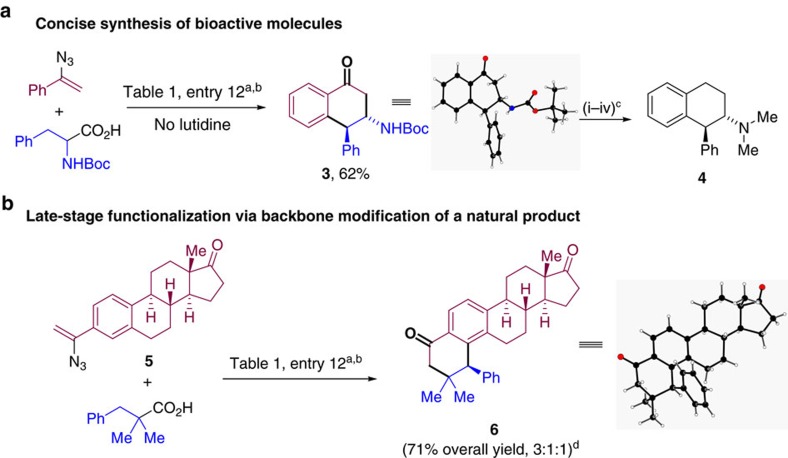
Synthetic applications. (**a**) Concise synthesis of bioactive molecules. (**b**) Late-stage functionalization via backbone modification of a natural product. ^a^See Table 1, entry 12 for detailed conditions. ^b^Isolated yields after column chromatography. ^c^Reaction conditions: (i) NaBH_4_ (1.3 equiv.), THF/MeOH, rt, 2 h; (THF, tetrahydrofuran; RT, room temperature) (ii) Pd/C (10 mol%), (NH_4_)O_2_CH (1 equiv.), HCO_2_H (4 equiv.), MeOH/H_2_O=4:1, 80 °C, 24 h, 81% for two steps; (iii) NaH (2 equiv.), MeI (3 equiv.), THF, 0 °C to rt, overnight, 92%; (iv) LiAlH_4_ (5 equiv), THF, reflux, 48 h, 84%. ^d^Ratio 3:1:1 corresponds to major regio- and diastereoisomer versus minor diastereo- and minor regioisomer, respectively. Major isomer was isolated in 45% yield.

The synthetic utility of these transformations was further demonstrated by the efficient conversion of (tert-butoxycarbonyl)phenylalanine into tetralone **3**. This compound provides a concise synthetic route (4 steps) to valuable molecules such as *trans*-1-phenyl-2-(dimethylamino)tetralin **4** (Fig. 2a). **4** and closely related compounds have been reported to be efficient ligands for human histamine H1-receptors with potential to treat neurodegenerative and neuropsychiatric disorders (Fig. 1b)^44^. X-ray diffraction analysis of **3** secured the relative configuration of the subsequent reaction products. We next sought to explore the possibility of applying this reaction in the context of a structure-diversification natural product synthesis setting^45^,^46^. To this end, we were pleased to observe the successful conversion of estrone-derived vinyl azide **5** with 2,2-dimethyl-3-phenylpropanoic acid into pentacyclic adduct **6** in overall 71% yield (Fig. 2b). These transformations highlight the potential of this methodology to broaden the structural diversity of highly complex biologically relevant blueprints and to impact structure-activity relationship (SAR) optimization in medicinal chemistry campaigns.

### Mechanistic investigation

**Figure 3:**
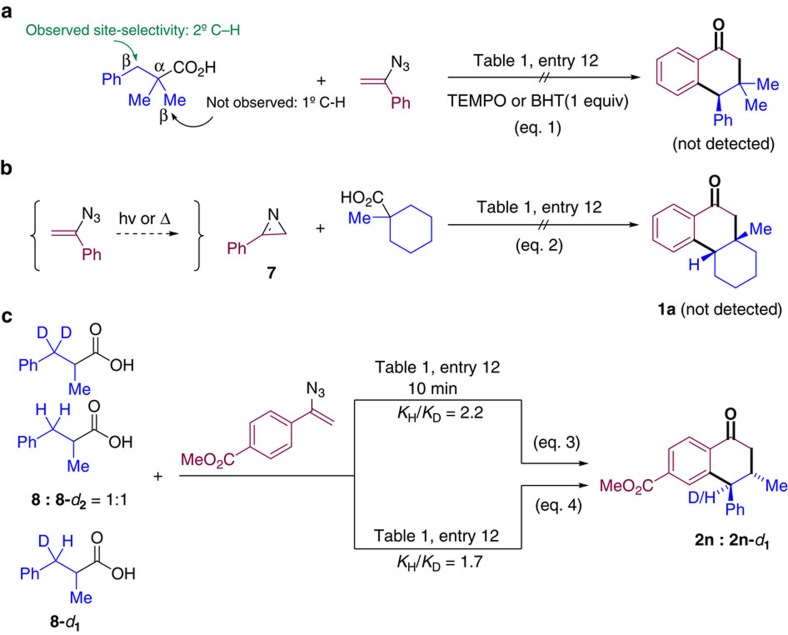
Mechanistic probes and deuterium-labelling experiments. (**a**) Control experiments with radical inhibitors. (**b**) Control experiments using 2*H*-azirine **7** as starting material instead of vinyl azide. (**c**) Inter- and intramolecular KIE experiment.

Diverse control experiments were designed to shed light on the mechanism of these transformations (Fig. 3a,b). The reaction of 1-methylcyclohexane-1-carboxylic acid and (1-azidovinyl)benzene was inhibited in the presence of TEMPO or BHT (Fig. 3a). In addition, the reactions of acids bearing both 1° and 2° C*sp*^3^–H groups in β-position to the carboxylic groups (Tables 2 and 3) proved to be site-selective, favouring functionalization of –CH_2_– over –CH_3_– in all studied cases, a reactivity trend consistent with the homolytic C–H bond dissociation energy (BDEs). These observations suggest a radical mechanism operating in the key C–H abstraction step.

2*H*-Azirines have been reported as intermediates in reactions involving vinyl azides as a result of the denitrogenative decomposition of the latter via vinyl nitrenes^47^,^48^. As shown in Fig. 3, 3-phenyl-2*H*-azirine **7** was not a productive partner under the standard conditions, which seems to rule out its participation in the present transformation (Fig. 3b). Deuterium-labelling experiments were also performed to gain additional mechanistic insights. A competition experiment was carried out using a 1:1 mixture of 2-methyl-3-phenylpropanoic acid **8** and its analogue **8**-*d*_2_ (Fig. 3c). The formation of **2n** and **2n**-*d*_1_ was monitored by both ^1^H nuclear magnetic resonance (NMR) and mass spectrometry so that a kinetic isotope effect (KIE) of 2.2 could be determined in this experiment based on detected product ratios. An intramolecular KIE of 1.7 was measured in the reaction of 2-methyl-3-phenylpropanoic-3-*d* acid (**8**-*d*_1_) with methyl 4-(1-azidovinyl)benzoate under the standard reaction conditions (equation 4). Both sets of KIE experiments are consistent with a reaction mechanism in which C–H bond cleavage is rate-limiting^49^,^50^.

**Figure 4:**
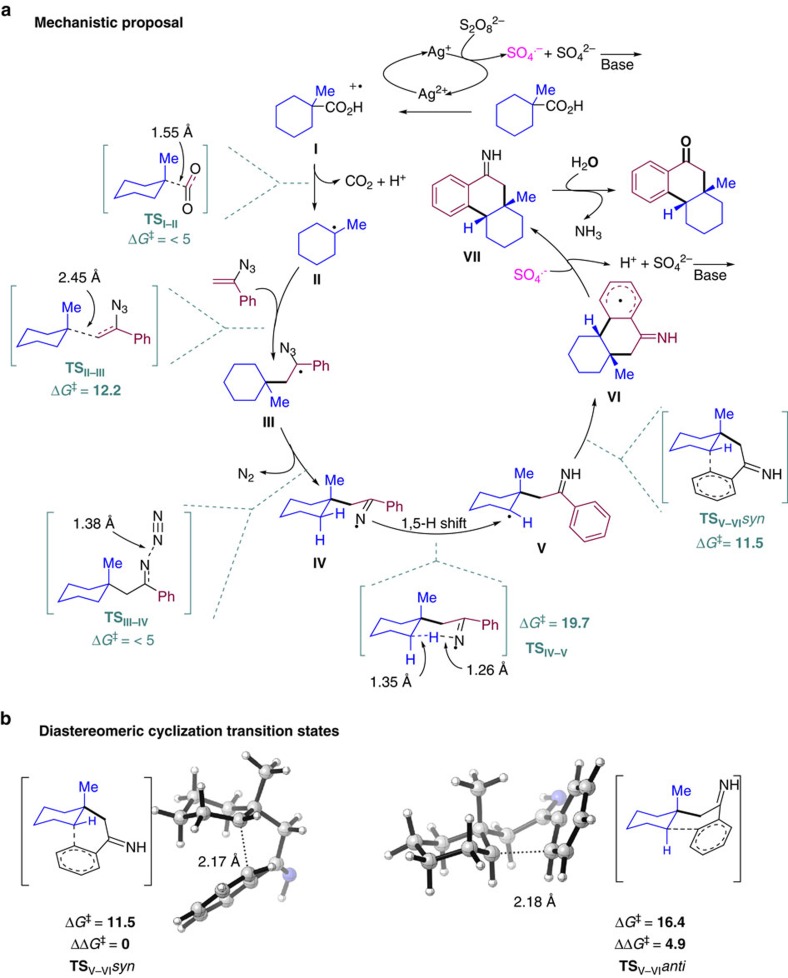
Mechanistic discussion. (**a**) Proposed reaction mechanism and transition states computed at M06-2X/6-311++G(d,p) (iefpcm, solvent=acetone) level. Energies are given in kcal mol^−1^ (relative to the sum of the starting materials, *G*_**I**_+*G*_vinylazide_=0 kcal mol^−1^). (**b**) Diastereoisomeric transition states for the cyclization step.

Density functional theory calculations were also carried out to gain additional insight into the reaction energy profile and the factors governing the observed stereoselectivity (Fig. 4). Ag(II) species are produced *in situ* as a result of the interaction of the silver(I) pre-catalyst with K_2_S_2_O_8_. In a single electron transfer (SET) process, the carboxylic acid is transformed into a radical cation **I**, which rapidly evolves via decarboxylation to produce **II** in a facile manner (TS_I–II_, Δ*G*^‡^<5 kcal mol^−1^)^38^,^39^,^42^,^43^. The alkyl radical intermediate undergoes addition onto the vinyl azide present in the reaction media to produce benzylic radical **III** (TS_II–III_, Δ*G*^‡^=12.2 kcal mol^−1^). Release of N_2_ is again highly favoured and results in the formation of imine radical **IV** (TS_III–IV_, Δ*G*^‡^<5 kcal mol^−1^)^34^,^35^,^36^,^37^. Although a single-electron recombination of **IV** with the metal centre could be envisioned, the reaction outcome suggests that a 1,5-H migration on the secondary aliphatic C–H bonds to produce a C-centred radical **V** is preferred^25^,^26^. The TS for this process (TS_IV–V_) presents the higher barrier along the reaction coordinate with a Δ*G*^‡^=19.7 kcal mol^−1^, which signalized the 1,5-H migration as rate-limiting step in these reactions. The results summarized in Tables 2 and 3 highlight the site selectivity of the reaction in favour of CH_2_ versus CH_3_ groups, in line with the homolytic binding dissociation energies of the different C*sp*^3^–H bonds. Radical **V** reacts with the aromatic ring^40^ to produce, on formation of a second C*sp*^2^–C*sp*^3^ bond, bicyclic intermediate **VI** as a single diastereoisomer. The aromatic radical is oxidized with SO_4_^ ˙−^, yielding imine intermediate **VII**. The hydrolysis of **VII** takes place in the presence of water in the reaction media furnishing the observed products. As shown in Fig. 4, the base seems to tame the acidic pH generated in the reaction media preventing the degradation of both starting materials and productive reaction intermediates.

The exquisite stereoselectivity of the reaction can be rationalized on the basis of the different interactions that build up in the TS connecting **V** and **VI**. As shown in Fig. 4b, TS_V–VI_*syn* is ca. 5 kcal mol^−1^ lower in energy than the corresponding TS TS_V–VI_*anti* as a result of the unfavourable steric interaction between the methyl group in axial relative position and the corresponding chain holding the aromatic ring (TS_V–VI_*syn* Δ*G*^‡^=11.5 versus TS_V–VI_*anti* Δ*G*^‡^=16.4 kcal mol^−1^). Analogously, the cyclization step in the case of acyclic carboxylic acid favour the *anti-*relative configuration in the final products.

In summary, a straightforward route to a variety of elaborated fused ketones is presented here based on a radical-mediated stereoselective C–H functionalization relay strategy. The reaction proceeds through a 1,5-H shift enabled by a directing-group free remote C*sp*^3^–H activation, followed by a C*sp*^2^–H functionalization in an intricate radical cascade. The use of cost-effective vinyl azides and aliphatic acids circumvents the traditional multi-step synthesis of pre-functionalized H-radical shift precursor. Notably, aliphatic acids serve as 1,2-diradical equivalents in these transformations in which two C–C and one C=O bond are formed in a single synthetic operation. Our mechanistic study indicates that the 1,5-H shift is connected to the rate-determining step of these transformations. The synthetic utility of this methodology was successfully demonstrated by the efficient synthesis of bioactive molecules and late-stage functionalization of natural products. We anticipate that this work will open new possibilities of employing hydrogen shift as a useful synthetic tool for undirected inert aliphatic C–H activation in the context of both pharmaceuticals and natural product synthesis.

## Methods

### General

Supplementary Figs 1–44 for the NMR spectra, Supplementary Figs 45 and 46 for spectra of KIE experiments, Supplementary Figs 47–51 for X-ray diffraction for **1a′**, **2n**, **2s**, **3** and **6**, Supplementary Tables 1–22 for X-ray diffraction analysis data for **1a′**, **2n**, **2s**, **3** and **6**, Supplementary Table 23 for computational study and Supplementary Methods for characterization data can be found in the Supplementary Information.

### General procedure for the reaction

Vinyl azide (0.3 mmol, 1.5 equiv.), carboxylic acid (0.2 mmol, 1.0 equiv.), Ag_2_CO_3_ (0.06 mmol, 0.3 equiv.) and K_2_S_2_O_8_ (0.4 mmol, 2.0 equiv.) were placed in a dry Schlenk tube. The reaction vessel was evacuated and filled with nitrogen three times. Acetonitrile (0.5 ml), acetone (0.2 ml), distilled water (1.5 ml) and 2,6-lutidine (0.24 mmol, 1.2 equiv.) were sequentially added at 25 °C. The reaction mixture was stirred at 50 °C for 10 h. The resulting mixture was extracted with EtOAc (15 ml) and the organic layer was washed with brine (10 ml), dried over anhydrous MgSO_4_, filtered and concentrated under reduced pressure. The crude product was purified by column chromatography on silica gel with hexane:ethyl acetate mixtures as eluent to give the corresponding products in pure form.

### Data availability

The X-ray crystallographic coordinates for structures reported in this study have been deposited at the Cambridge Crystallographic Data Centre (CCDC) under deposition numbers 1504120, 1445931, 1481107, 1445926 and 1445932. These data can be obtained free of charge from The CCDC via www.ccdc.cam.ac.uk/data_request/cif. The authors declare that all other data supporting the findings of this study are available within the article and its Supplementary Information files.

## Additional information

**How to cite this article:** Shu, W. *et al*. Expeditious diastereoselective synthesis of elaborated ketones via remote C*sp*^3^–H functionalization. *Nat. Commun.* **8,** 13832 doi: 10.1038/ncomms13832 (2017).

**Publisher's note:** Springer Nature remains neutral with regard to jurisdictional claims in published maps and institutional affiliations.

## Supplementary Material

Supplementary Information Supplementary figures, supplementary tables, supplementary methods and supplementary references.

## Figures and Tables

**Table 1:** Optimization of the reaction conditions.

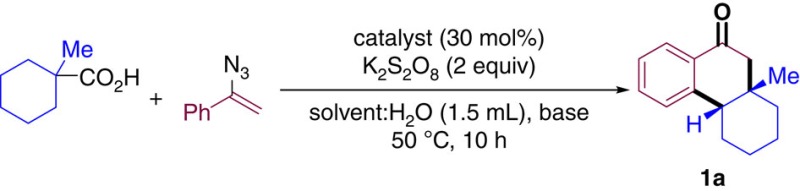					
**Entry**	**Catalyst**	**Solvent (ml)**	**Co-solvent (ml)**	**Base (equiv,)**	% yield 1a*
1	AgNO_3_	CH_3_CN (1.5)	—	—	5
2	AgNO_3_	Acetone (1.5)	—	—	8
3	AgNO_3_	CH_2_Cl_2_ (1.5)	—	—	Trace
4	Ag_2_CO_3_	Acetone (1.5)	—	—	22
5	Ag_2_CO_3_	Acetone (1.5)	CH_3_CN (0.5)	—	53
6	Ag_2_CO_3_	Acetone (1.5)	DMF (0.5)	—	31
7	Ag_2_CO_3_	Acetone (1.5)	CH_2_Cl_2_ (0.5)	—	8
8	Ag_2_CO_3_	Acetone (0.2)	CH_3_CN (0.5)	—	58
9	Ag_2_CO_3_	Acetone (0.2)	CH_3_CN (0.5)	2,6-lutidine (1)	62
10	Ag_2_CO_3_	Acetone (0.2)	CH_3_CN (0.5)	DIPEA (1)	31
11	Ag_2_CO_3_	Acetone (0.2)	CH_3_CN (0.5)	2,6-di-tertbutylpyridine (1)	47
**12**	**Ag**_**2**_**CO**_**3**_	**Acetone (0.2)**	**CH**_**3**_**CN (0.5)**	**2,6-lutidine (1.2)**	**68 (69)**

DIPEA: *N,N*-diisopropylethylamine.

^*^Yield determined by ^1^H-NMR with 1,3,5-trimethoxybenzene as internal standard. In brackets, isolated yield after column chromatography. The bold of entry 12 indicates this entry as the optimal conditions.

**Table 2:** Reaction scope on the vinyl azide.

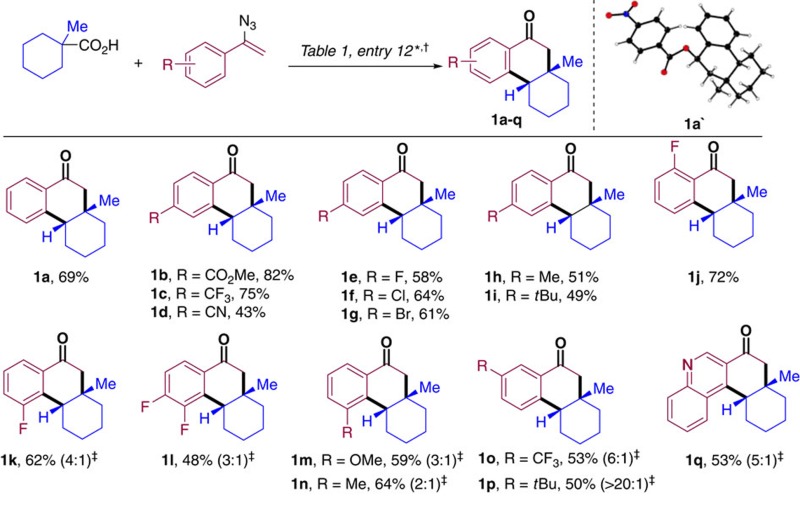

*See Table 1, entry 12 for detailed conditions.

†Isolated yields after column chromatography.

‡In brackets regioisomeric ratio determined by ^1^H-NMR of the crude reaction mixture. Major regioisomer depicted.

**Table 3:** Reaction scope on the carboxylic acid.

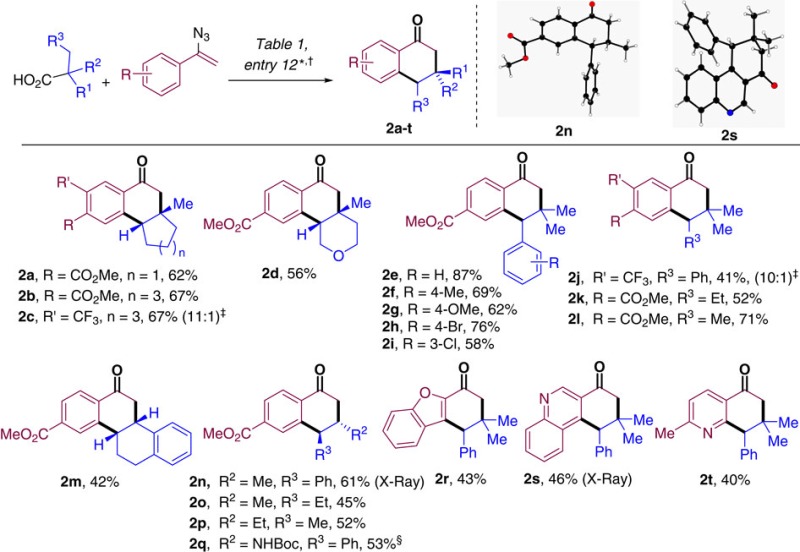

*See Table 1, entry 12 for detailed conditions. Unless otherwise stated, R=H, R′=H.

†Isolated yields after column chromatography.

‡In brackets regioisomeric ratio determined by ^1^H-NMR of the crude reaction mixture. Major regioisomer depicted.

§No lutidine was used.
