# Supplementary material for: Expeditious diastereoselective synthesis of elaborated ketones via remote Csp3–H functionalization
Source: Nat Commun. 2017 Jan 13;8:13832. doi: 10.1038/ncomms13832 (PMC5241786; doi:10.1038/ncomms13832)
Supplement: Supplementary Information — Supplementary figures, supplementary tables, supplementary methods and supplementary references. [file ncomms13832-s1.pdf]

Supplementary Figures

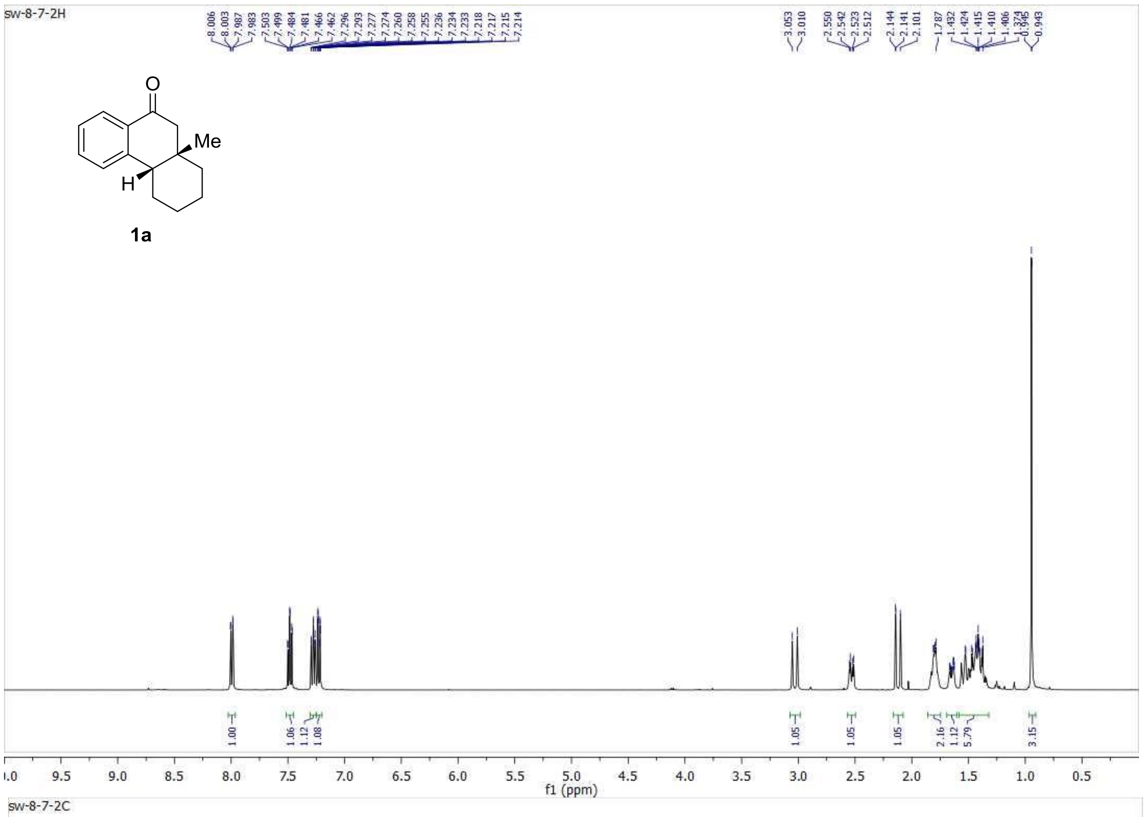

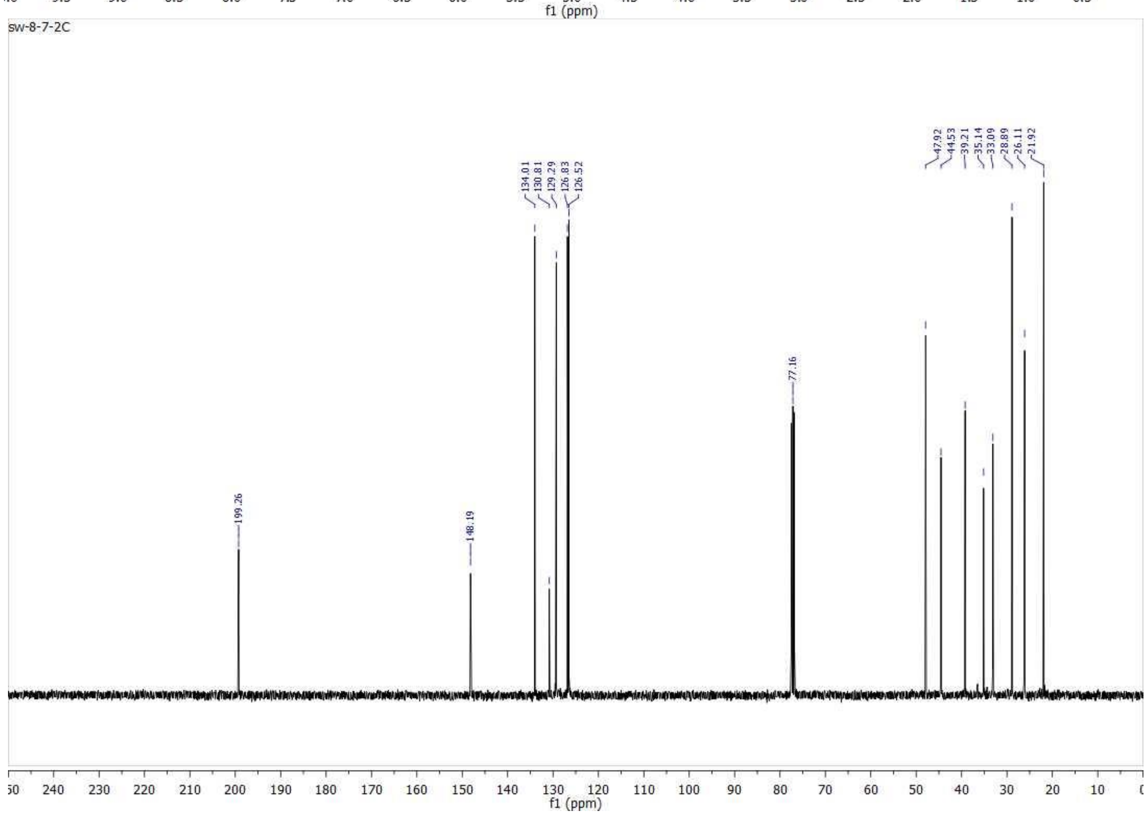

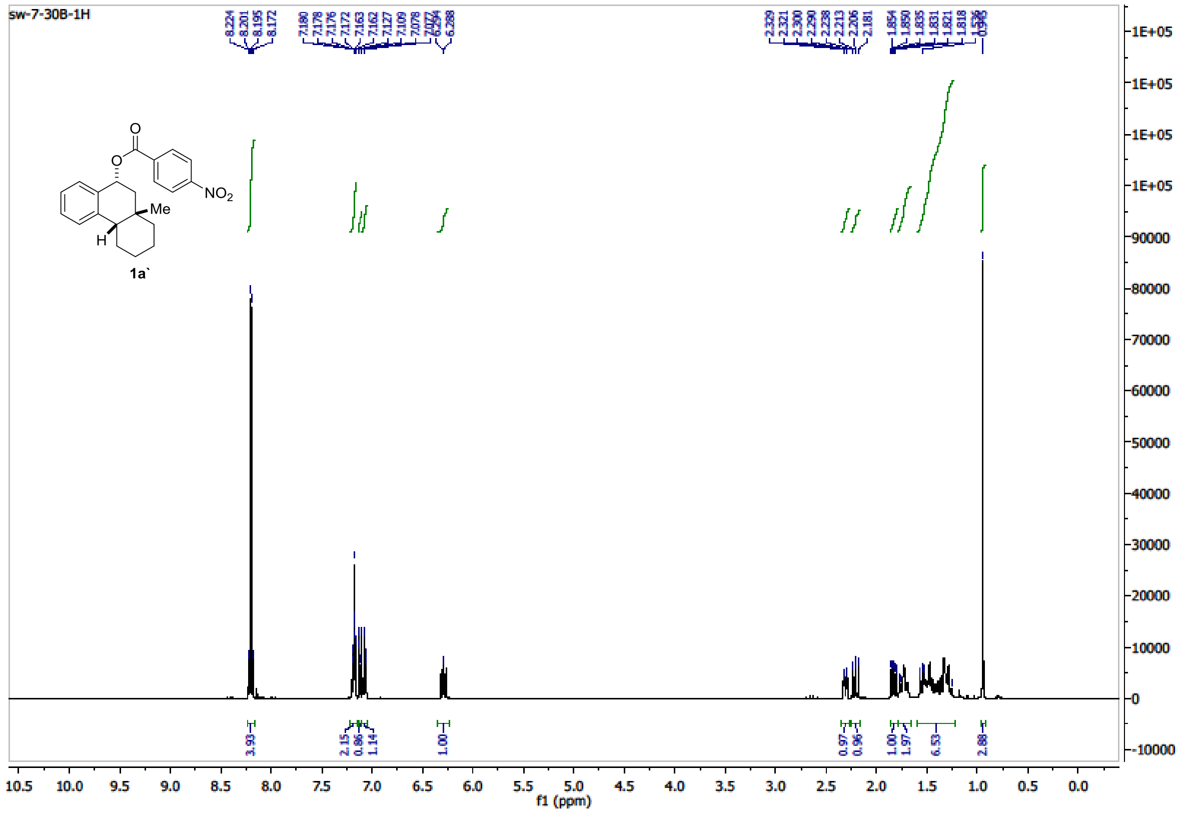

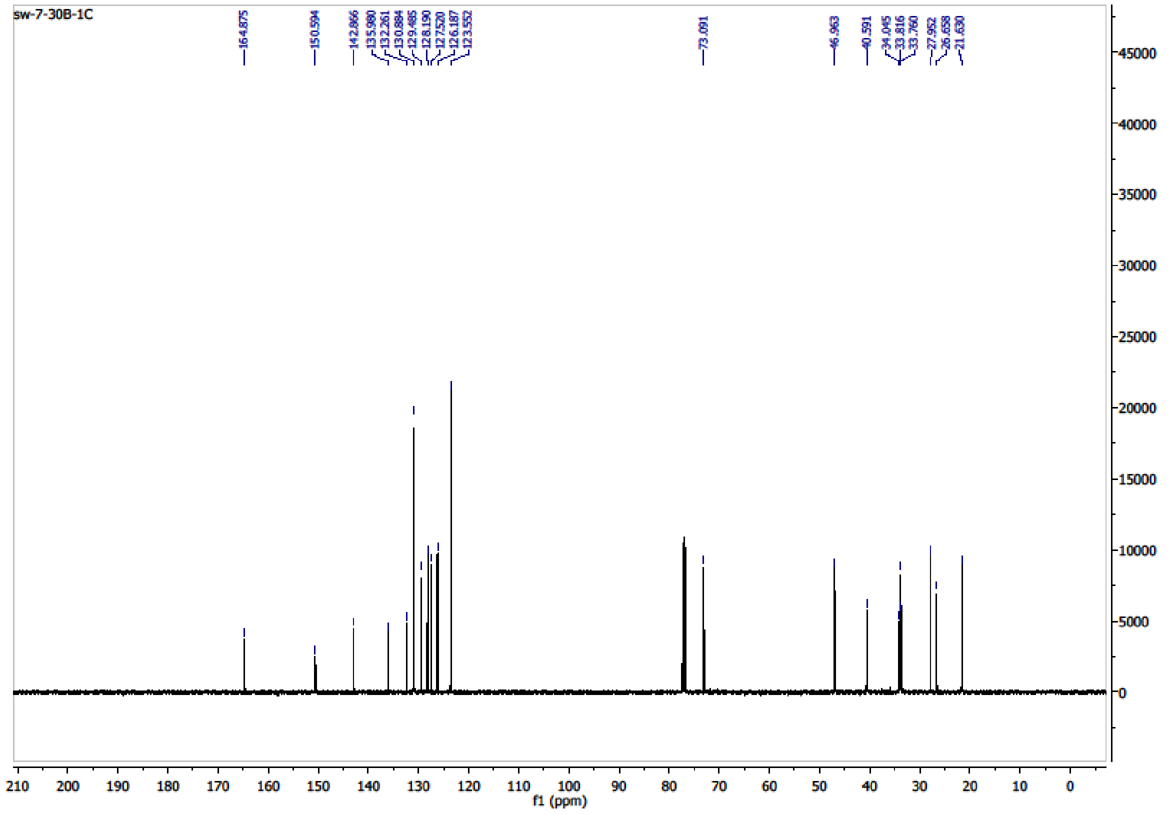

Supplementary Figure 1. ^{1}H and ^{13}C NMR Spectra of **1a** and **1a'**.

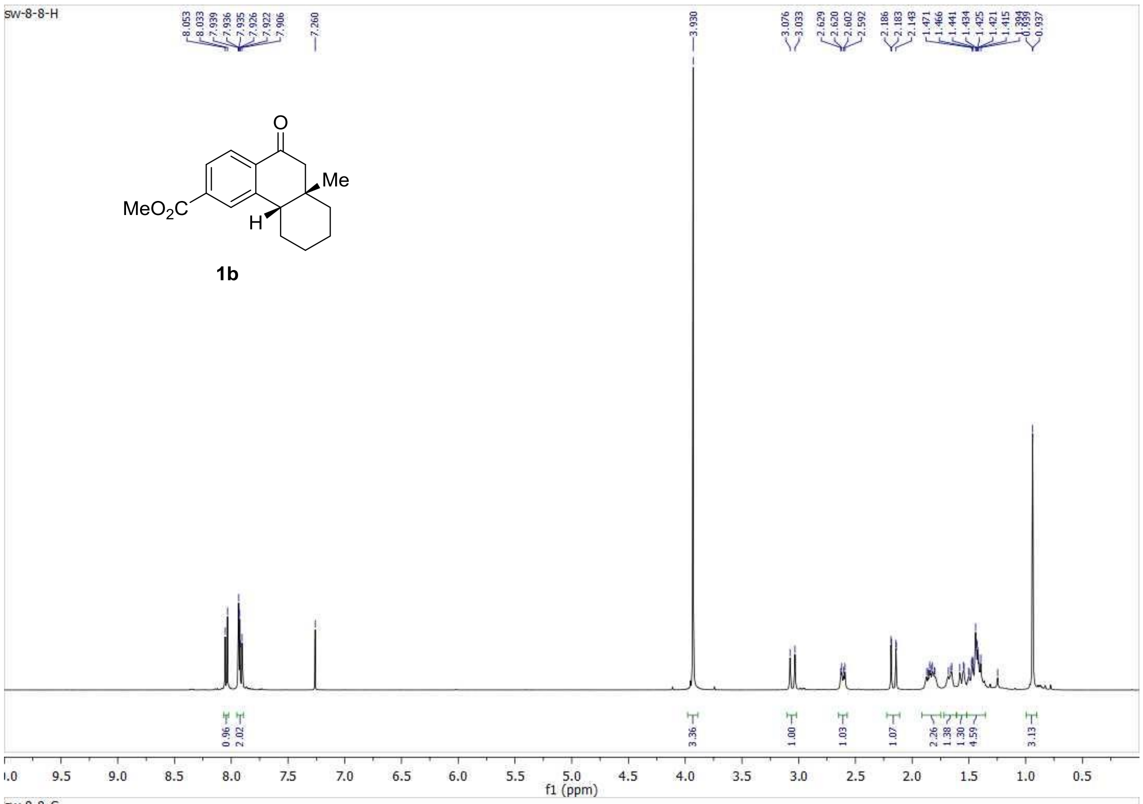

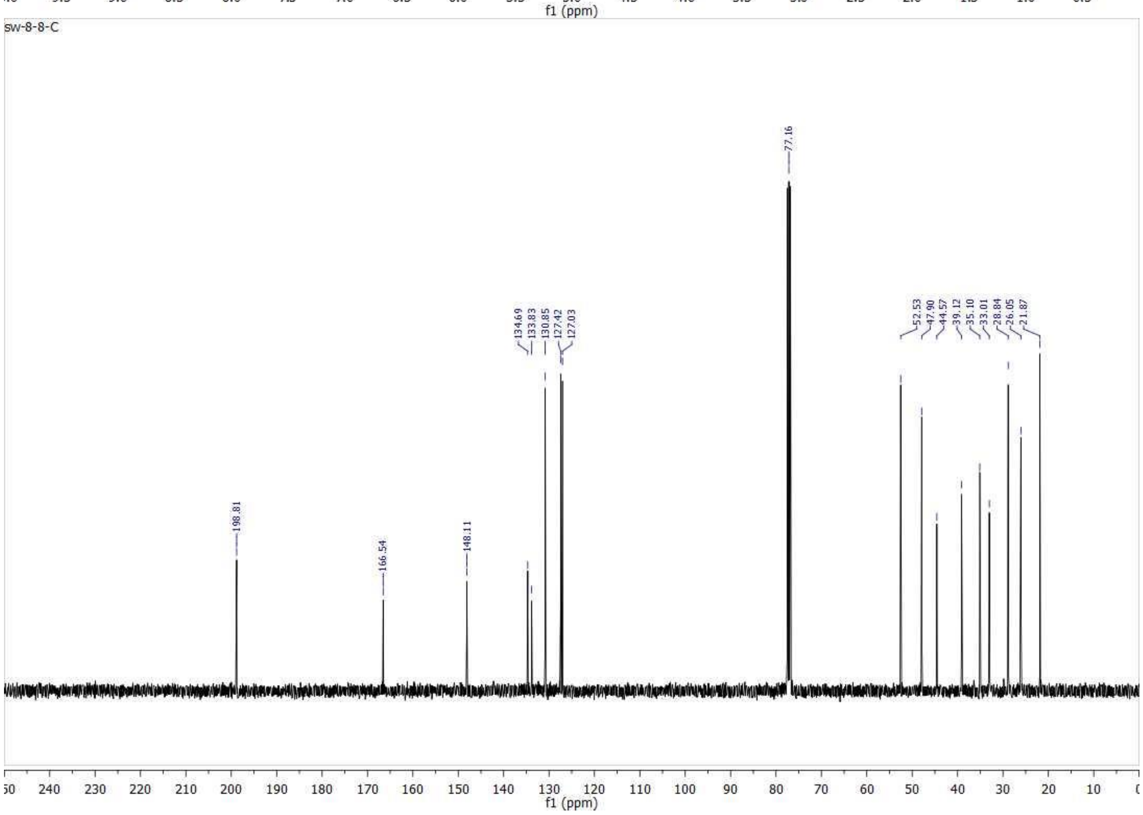

Supplementary Figure 2. ^{1}H and ^{13}C NMR Spectra of **1b**.

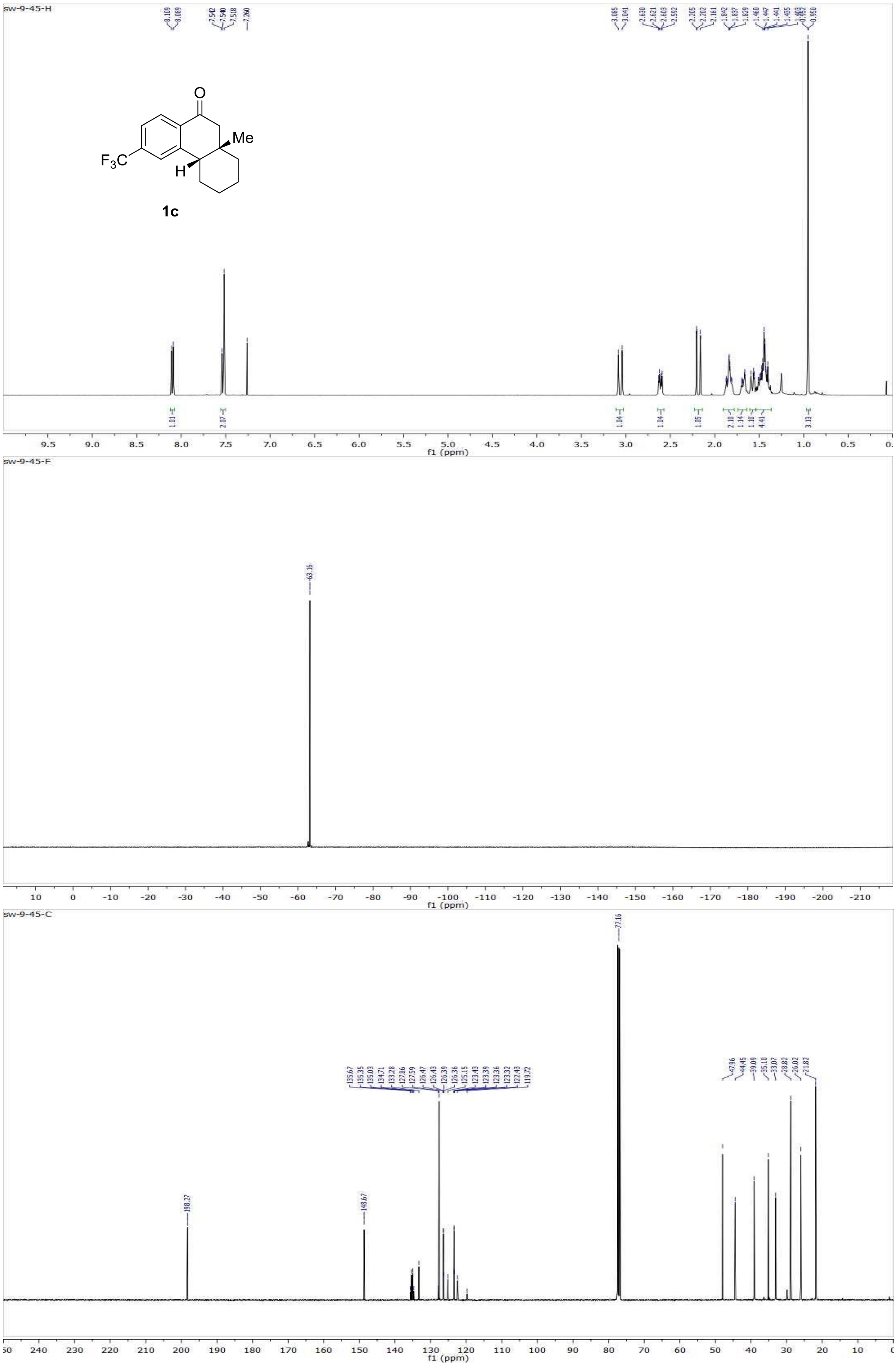

Supplementary Figure 3. ^{1}H, ^{19}F and ^{13}C NMR Spectra of **1c**.

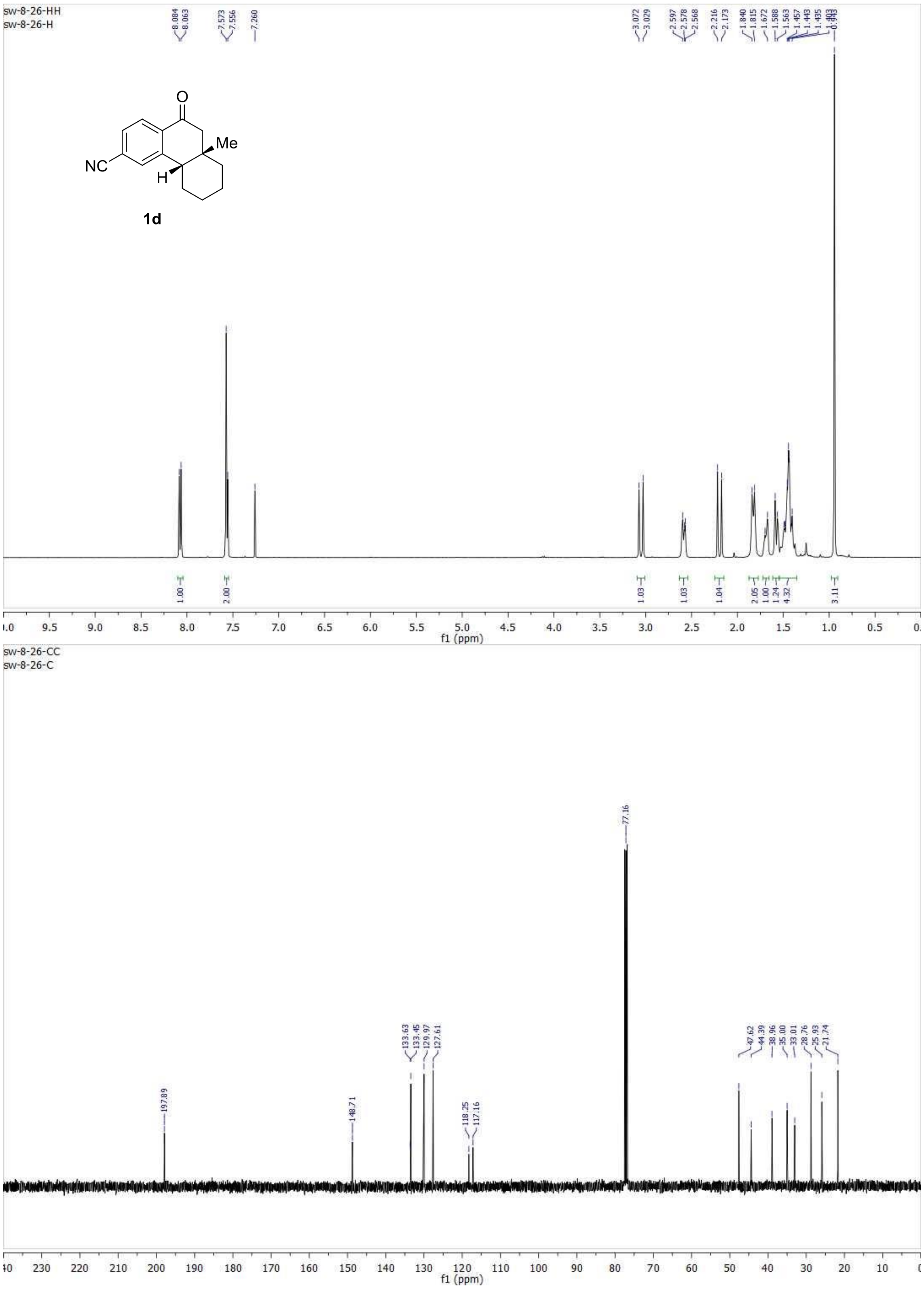

Supplementary Figure 4. ^{1}H and ^{13}C NMR Spectra of **1d**.

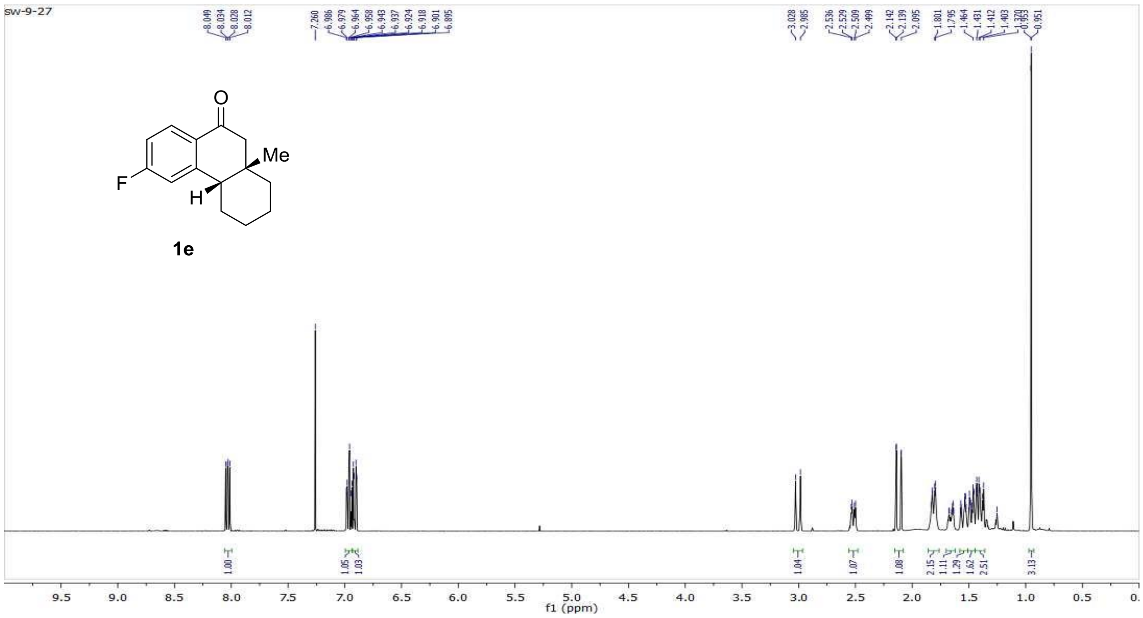

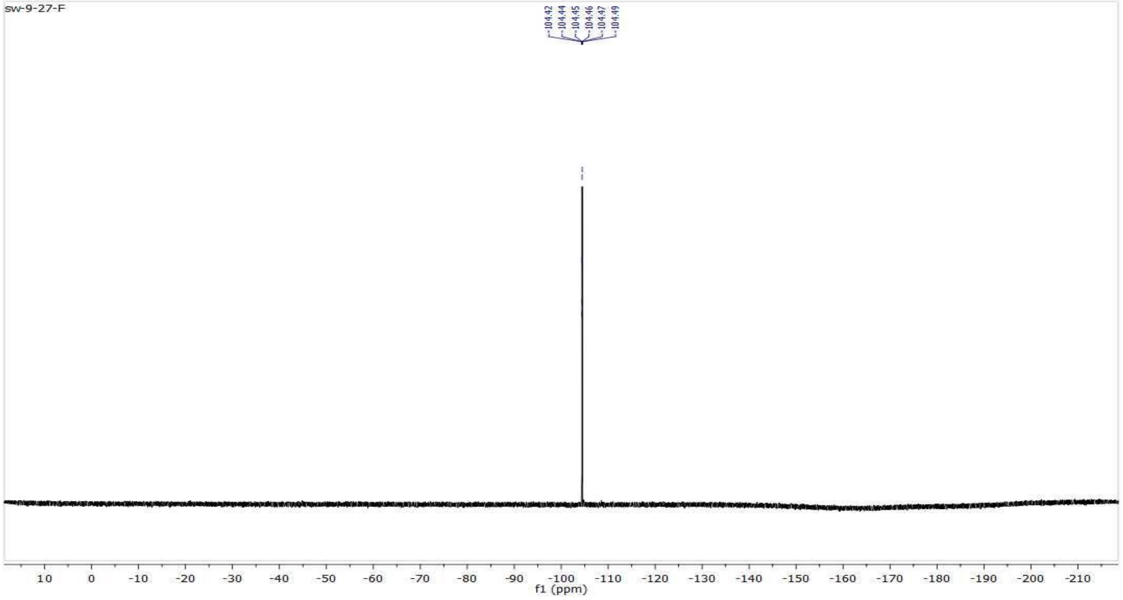

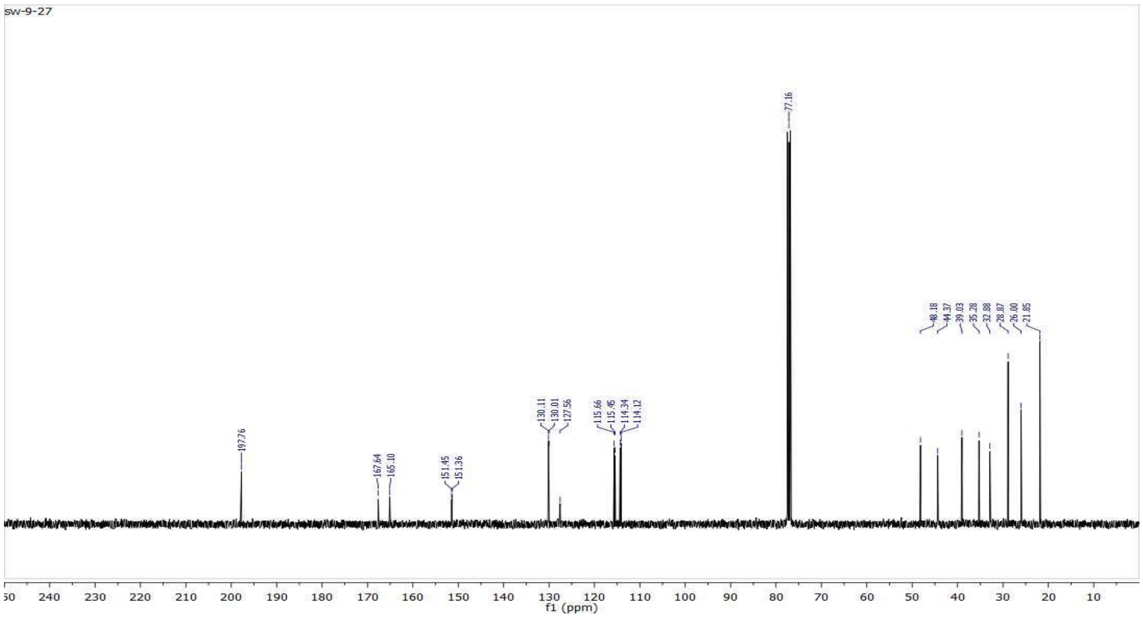

Supplementary Figure 5. ^{1}H, ^{19}F and ^{13}C NMR Spectra of **1e**.

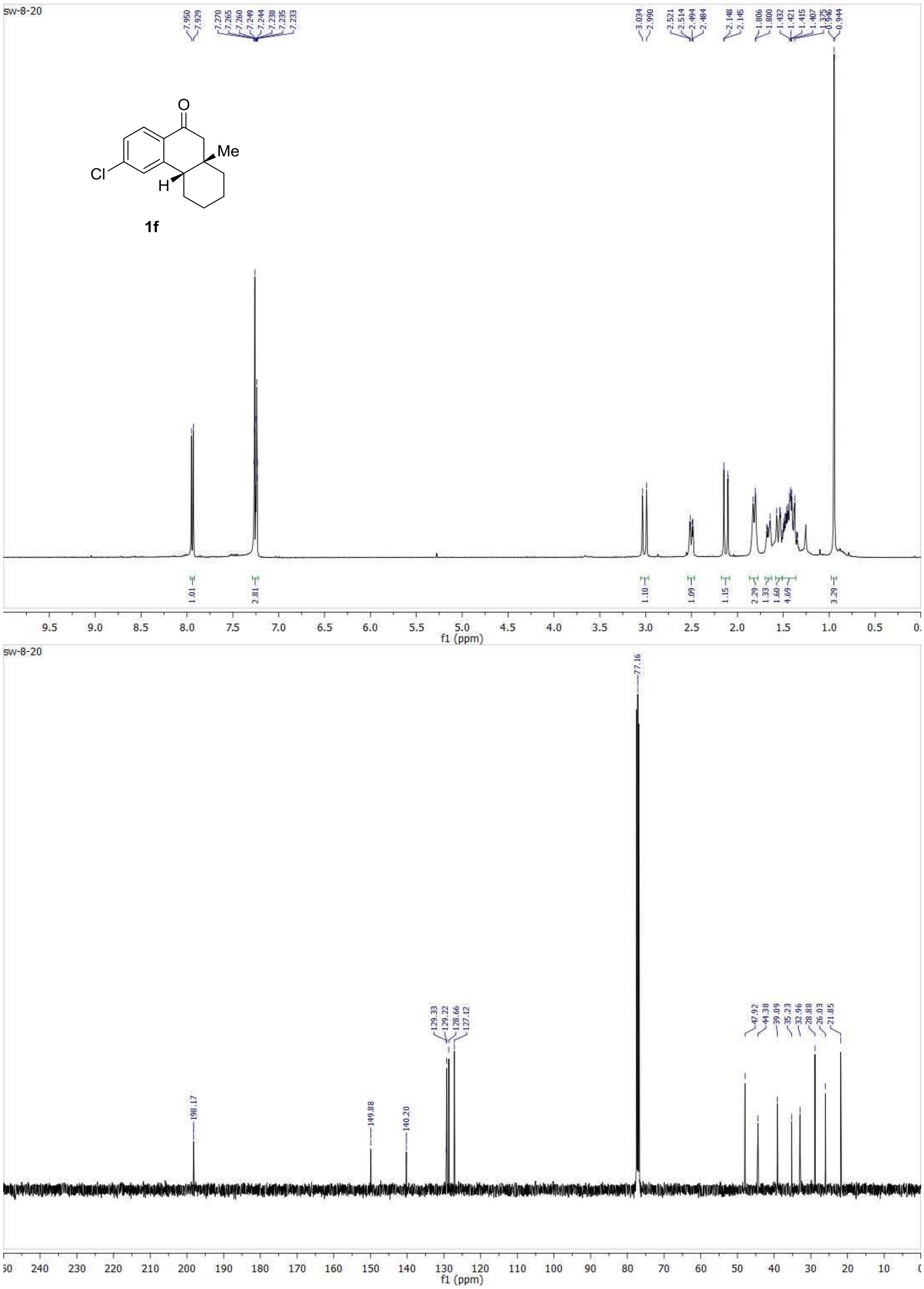

Supplementary Figure 6. ^{1}H and ^{13}C NMR Spectra of **1f**.

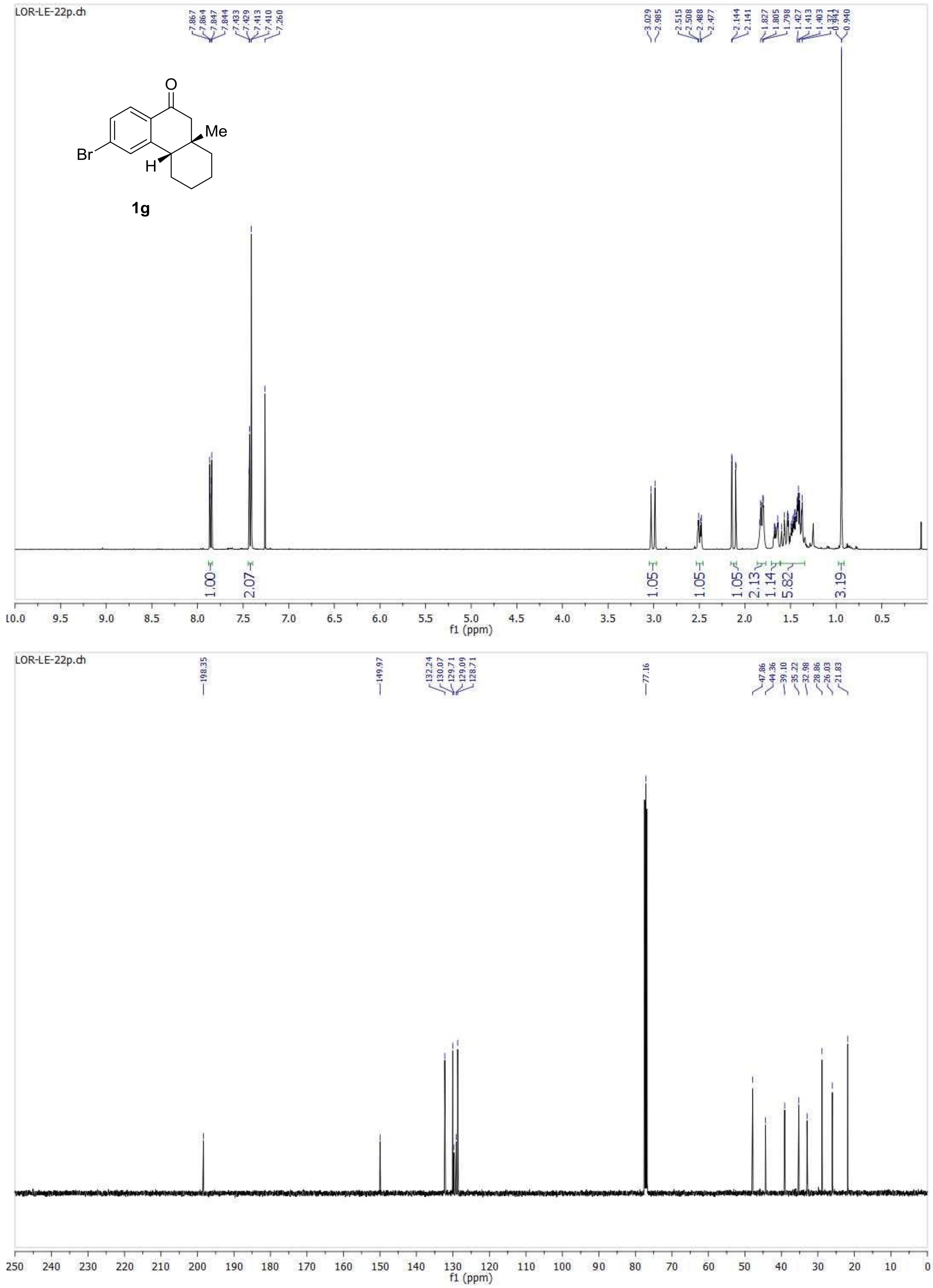

Supplementary Figure 7. ^{1}H and ^{13}C NMR Spectra of **1g**.

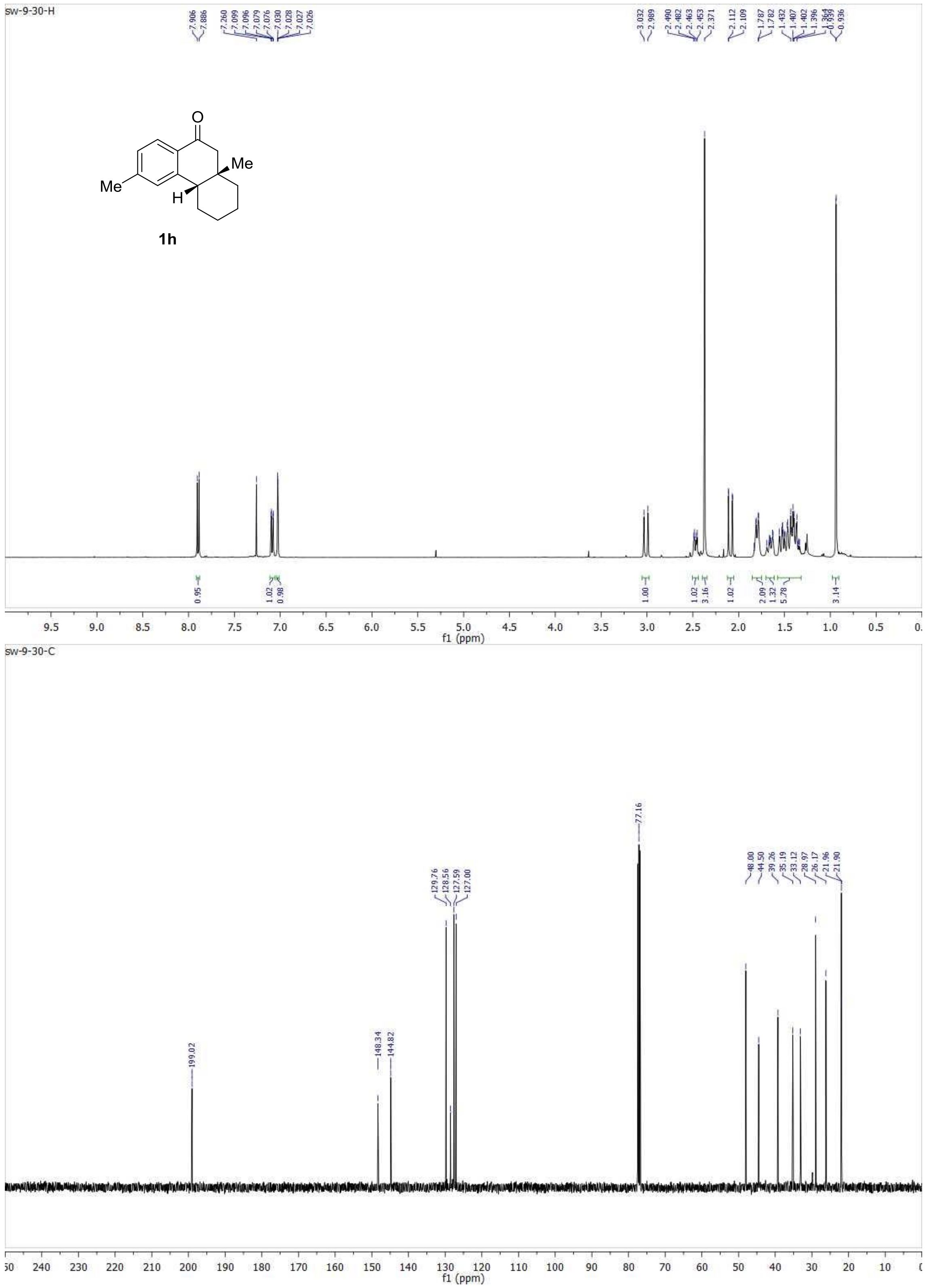

Supplementary Figure 8. ^{1}H and ^{13}C NMR Spectra of **1h**.

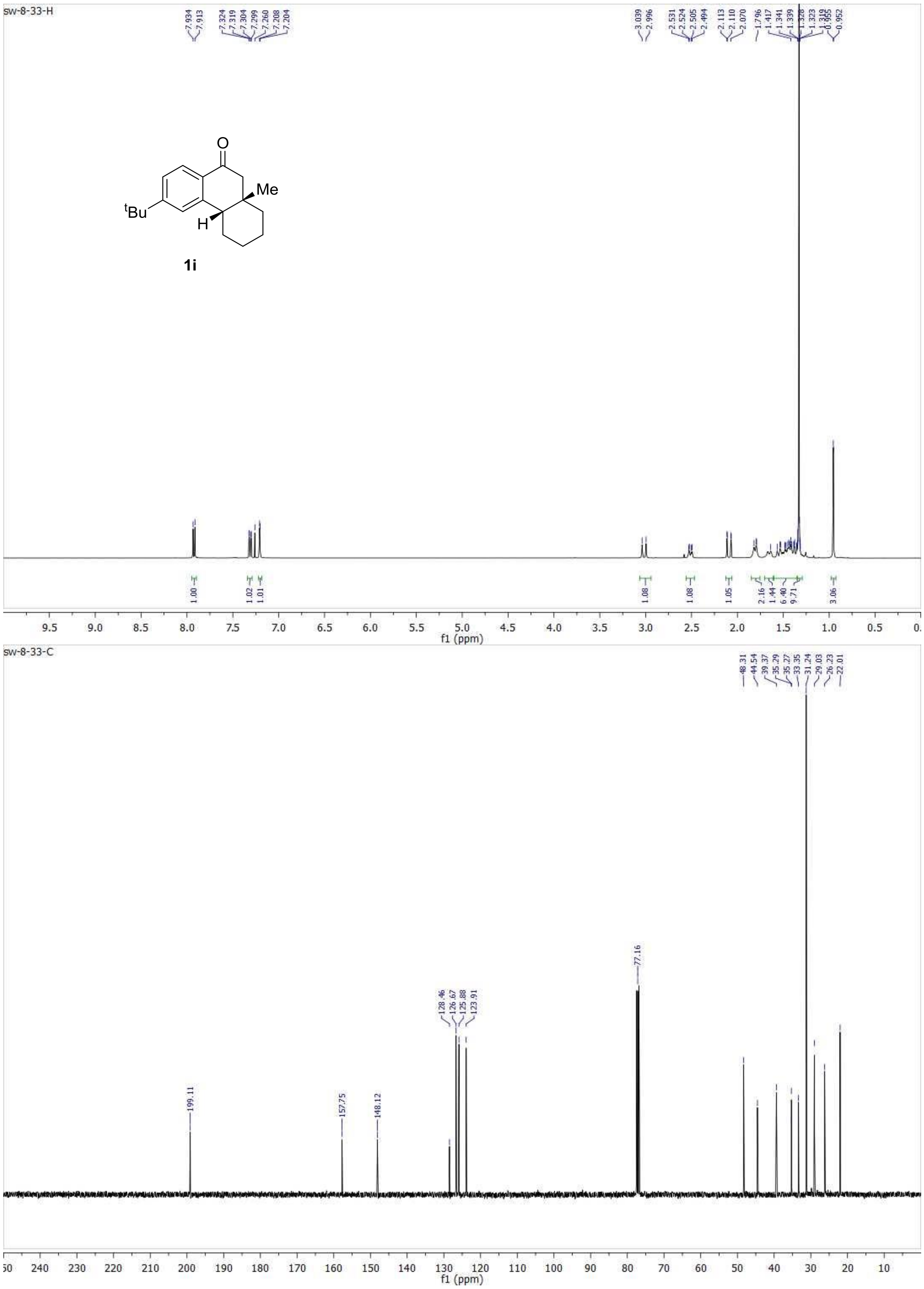

Supplementary Figure 9. ^{1}H and ^{13}C NMR Spectra of **1i**.

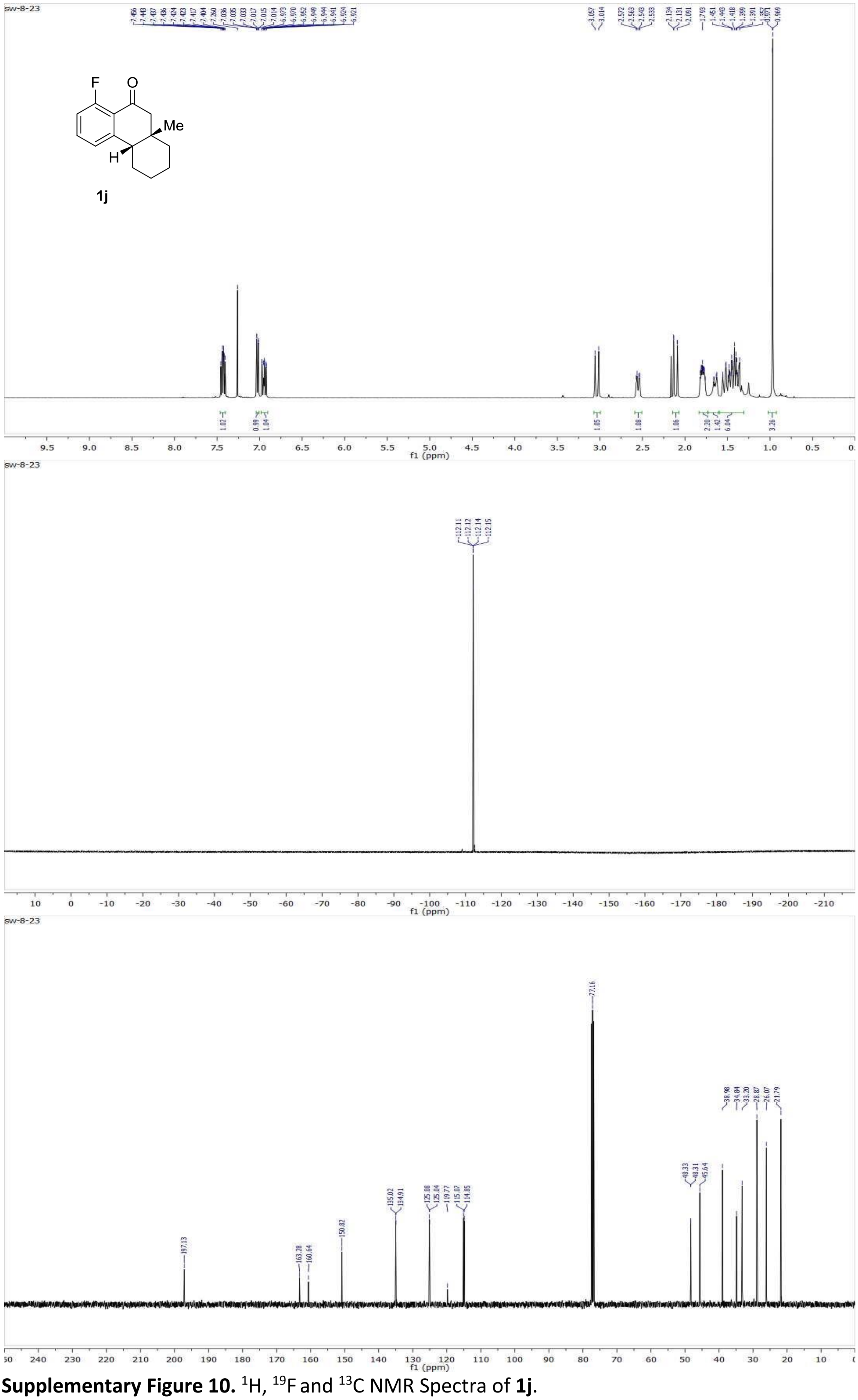

Supplementary Figure 10. ^{1}H, ^{19}F and ^{13}C NMR Spectra of **1j**.

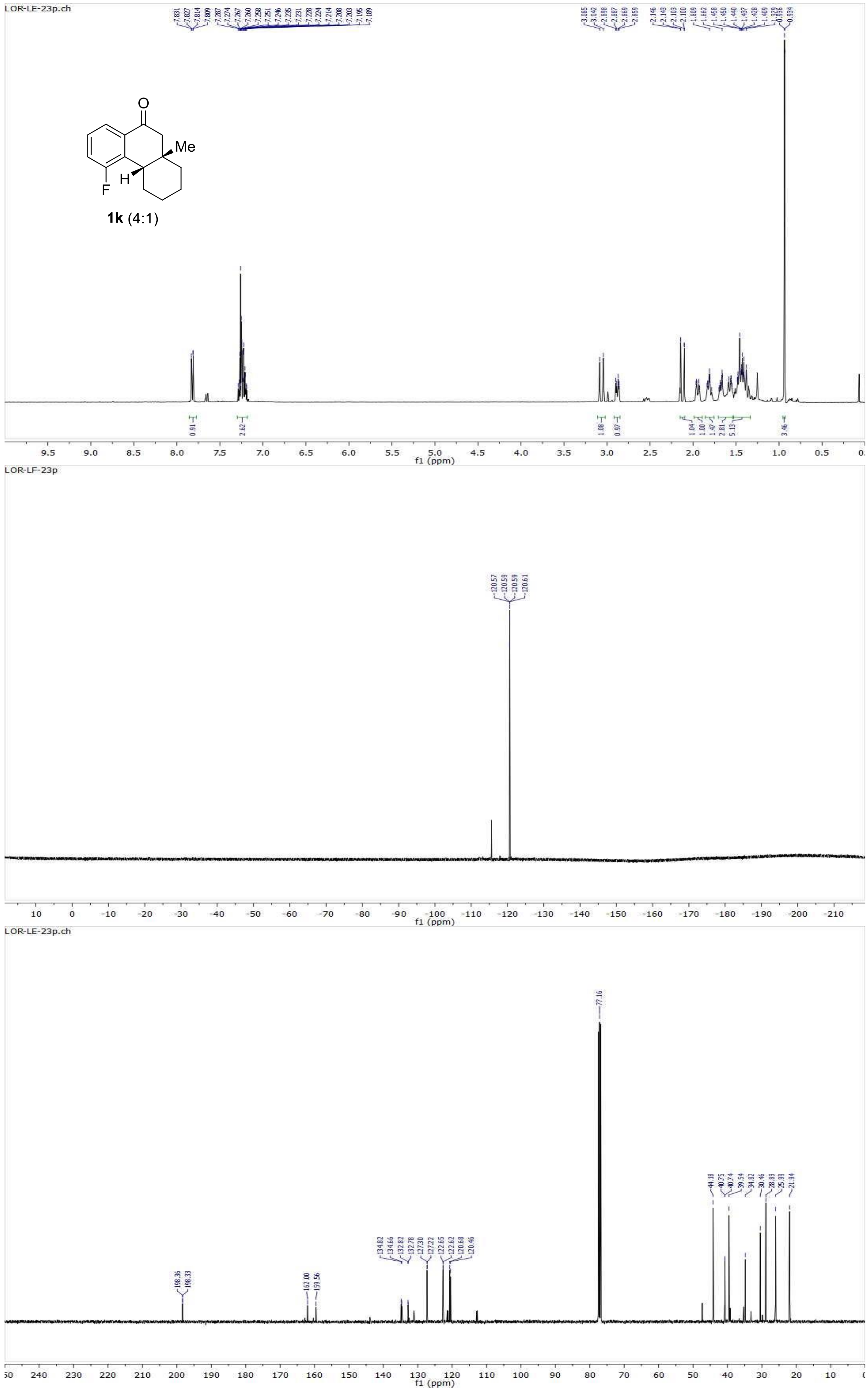

Supplementary Figure 11. ^{1}H, ^{19}F and ^{13}C NMR Spectra of **1k**.

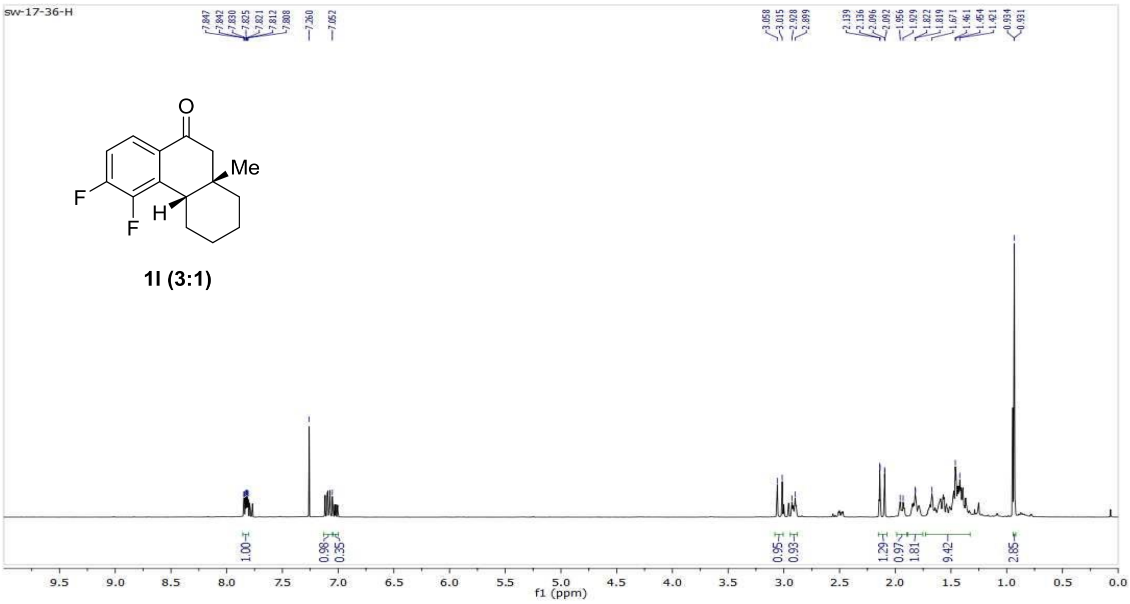

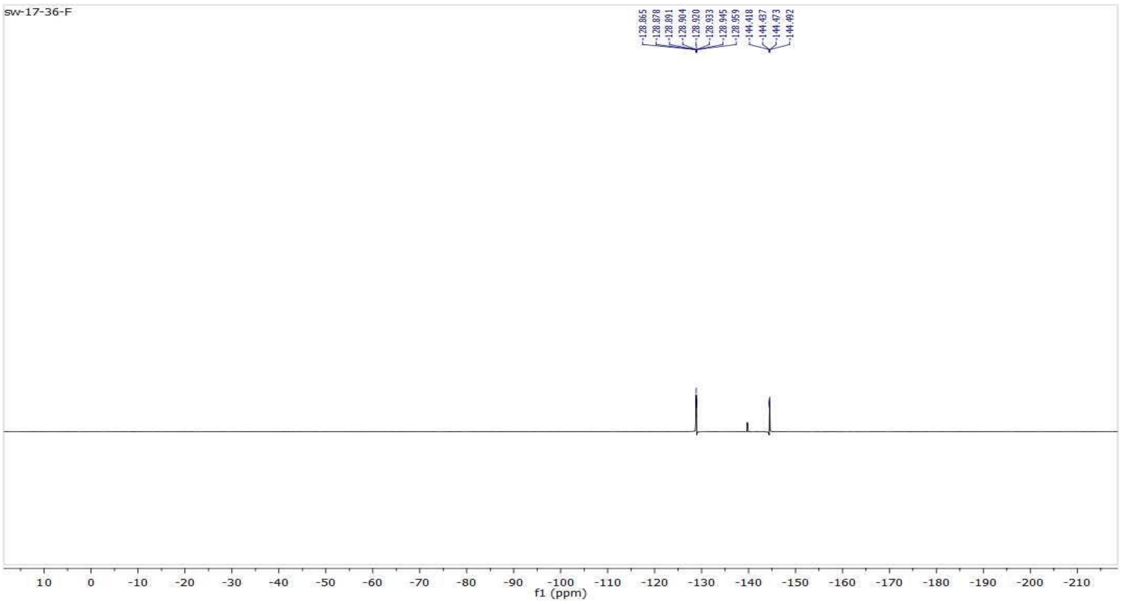

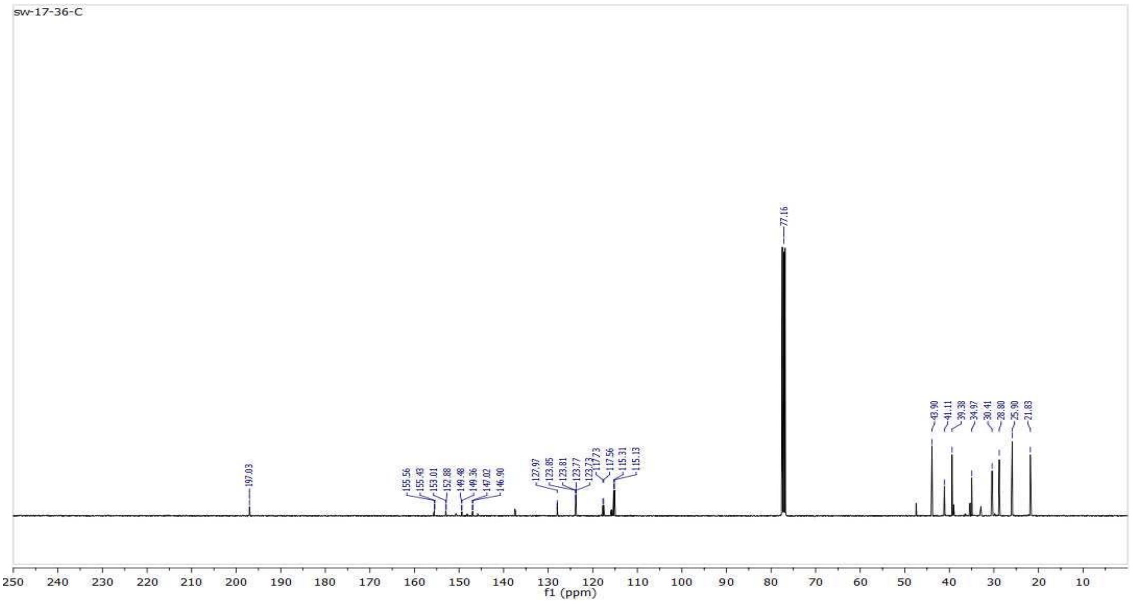

Supplementary Figure 12. ^{1}H, ^{19}F and ^{13}C NMR Spectra of **1l**.

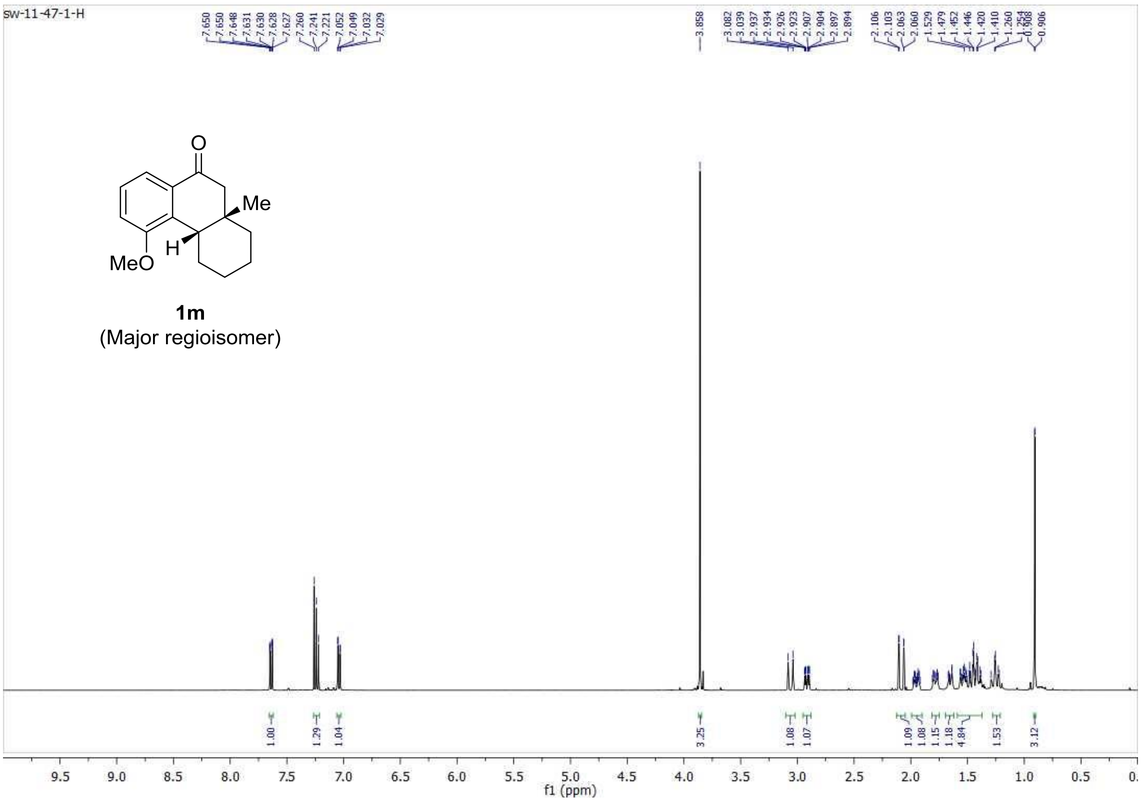
sw-11-47-1-H
7.650
7.650
7.648
7.631
7.630
7.628
7.627
7.260
7.241
7.221
7.052
7.049
7.032
7.029
3.858
3.082
3.039
2.937
2.934
2.926
2.923
2.907
2.904
2.897
2.894
2.106
2.103
2.063
2.060
1.529
1.479
1.462
1.446
1.420
1.410
1.260
0.908
0.906
O
Me
H
MeO
1m
(Major regioisomer)
f1 (ppm)

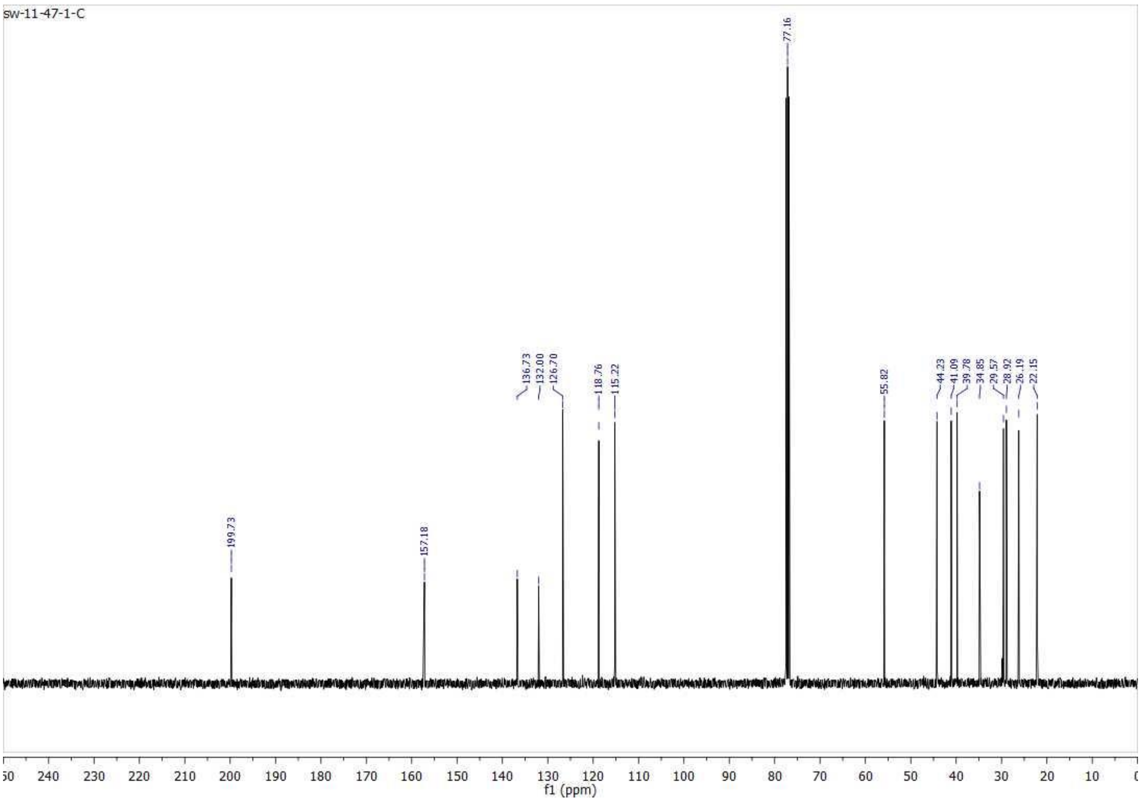
sw-11-47-1-C
199.73
157.18
136.73
132.00
126.70
118.76
115.22
77.16
55.82
44.23
41.09
39.78
34.85
29.57
28.92
26.19
22.15
f1 (ppm)

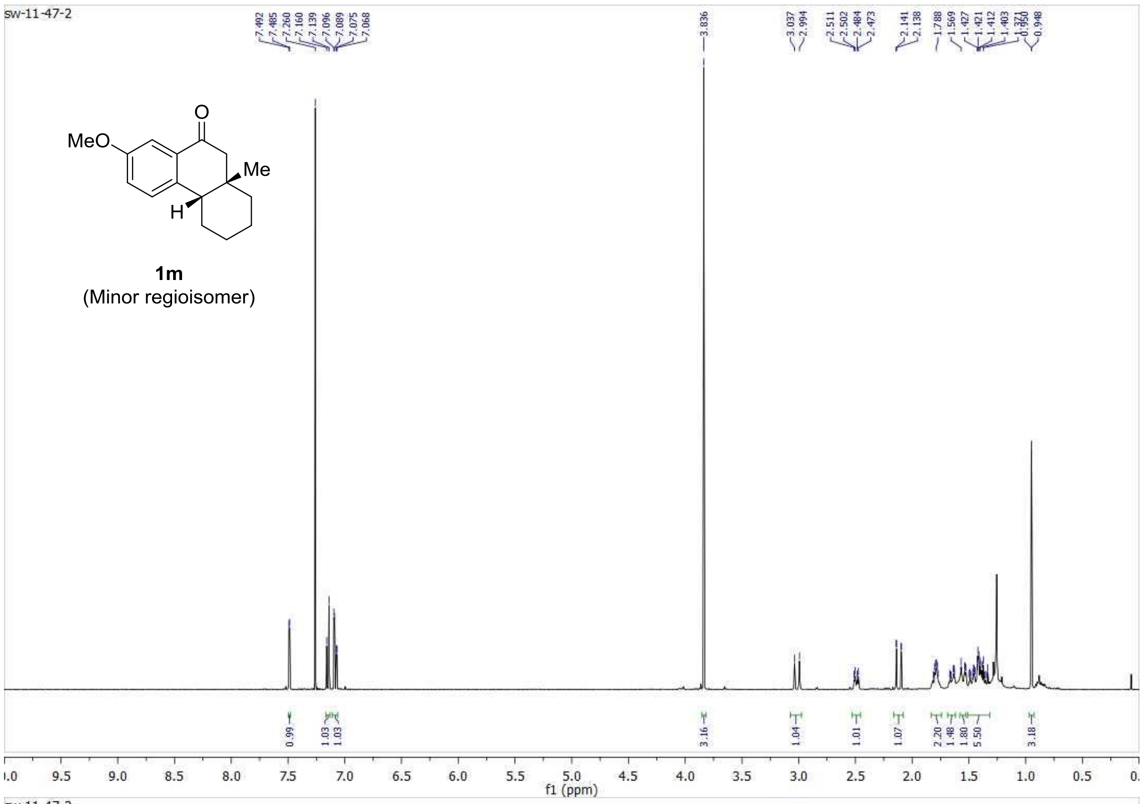

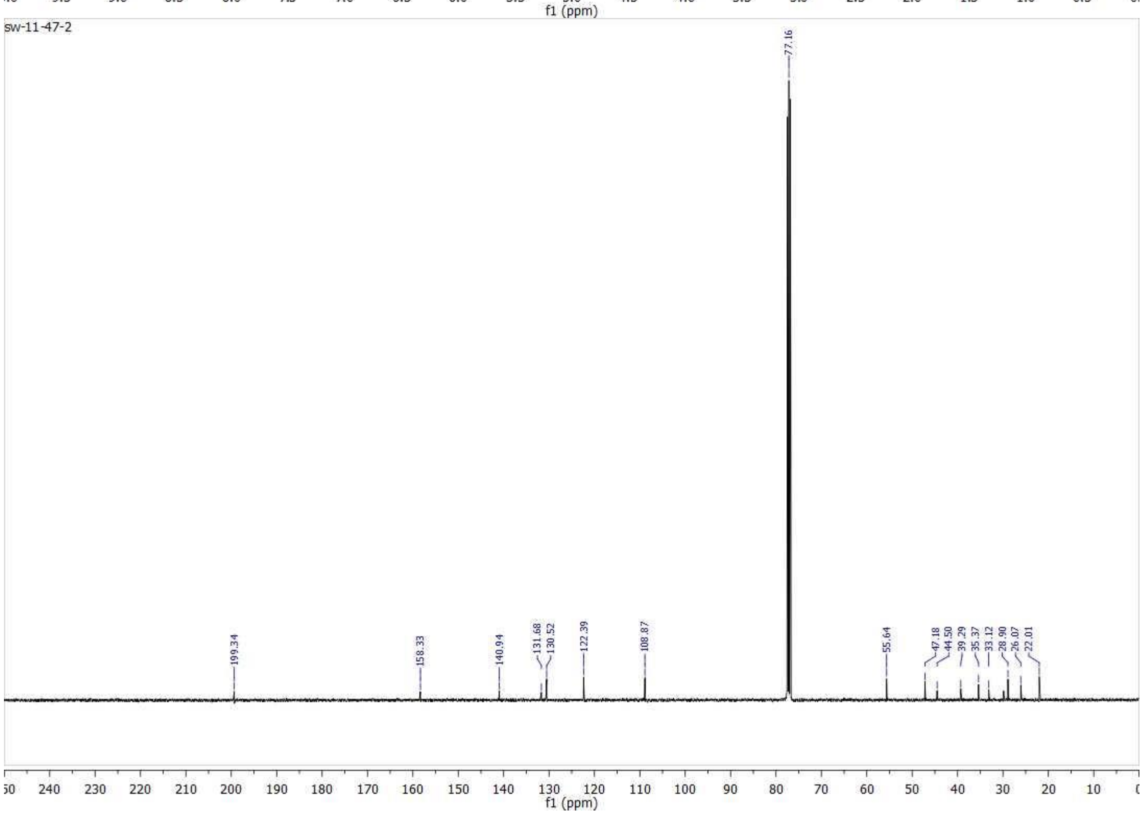

Supplementary Figure 13. ^{1}H and ^{13}C NMR Spectra of **1m**.

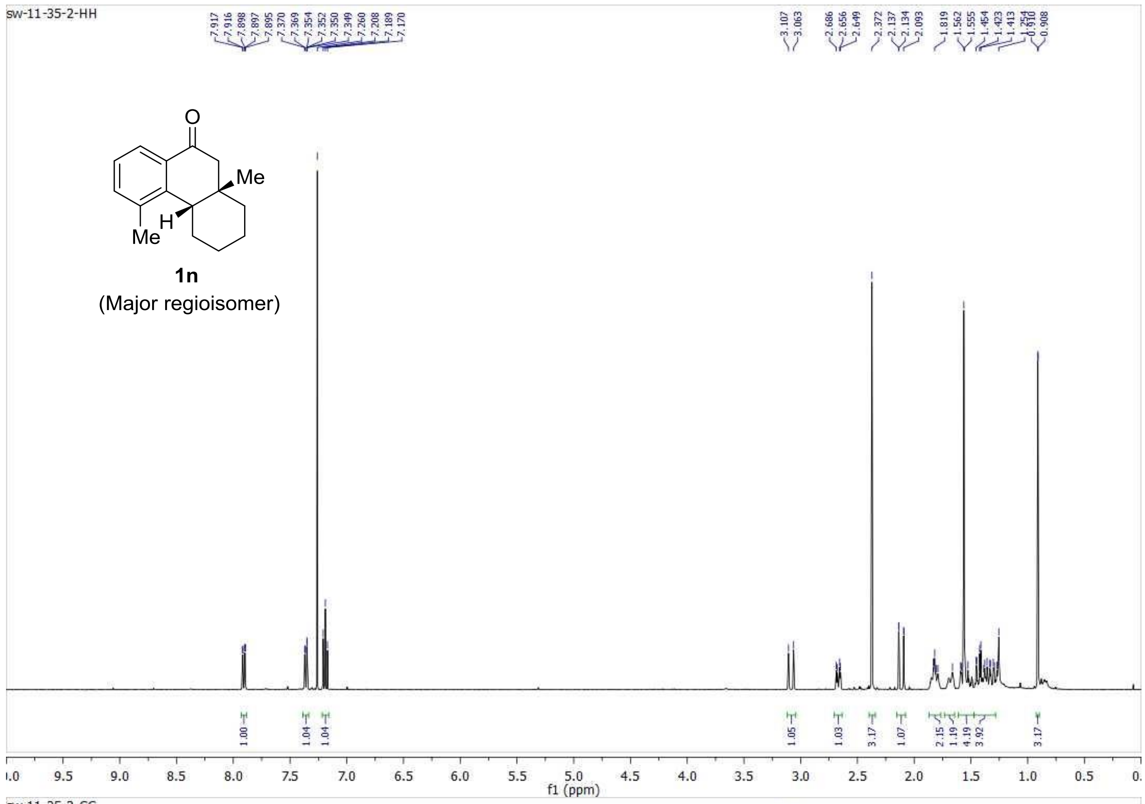

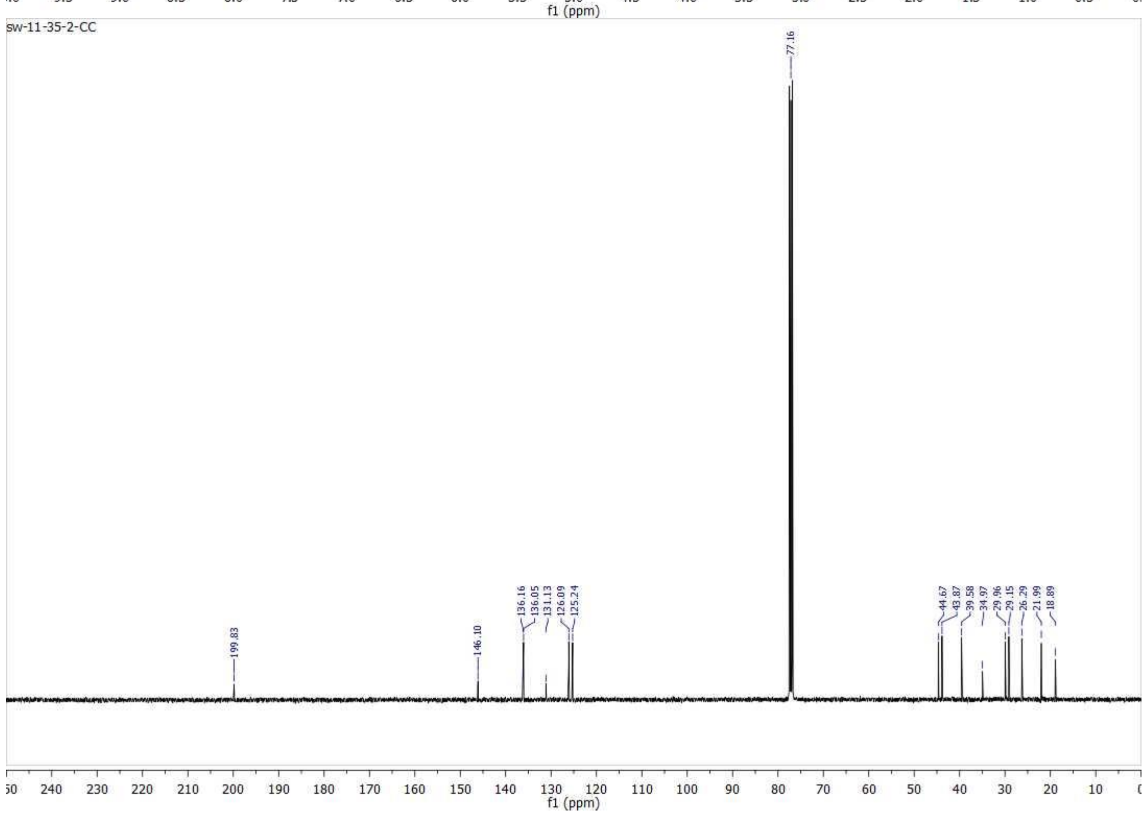

Supplementary Figure 14. ^{1}H and ^{13}C NMR Spectra of **1n**.

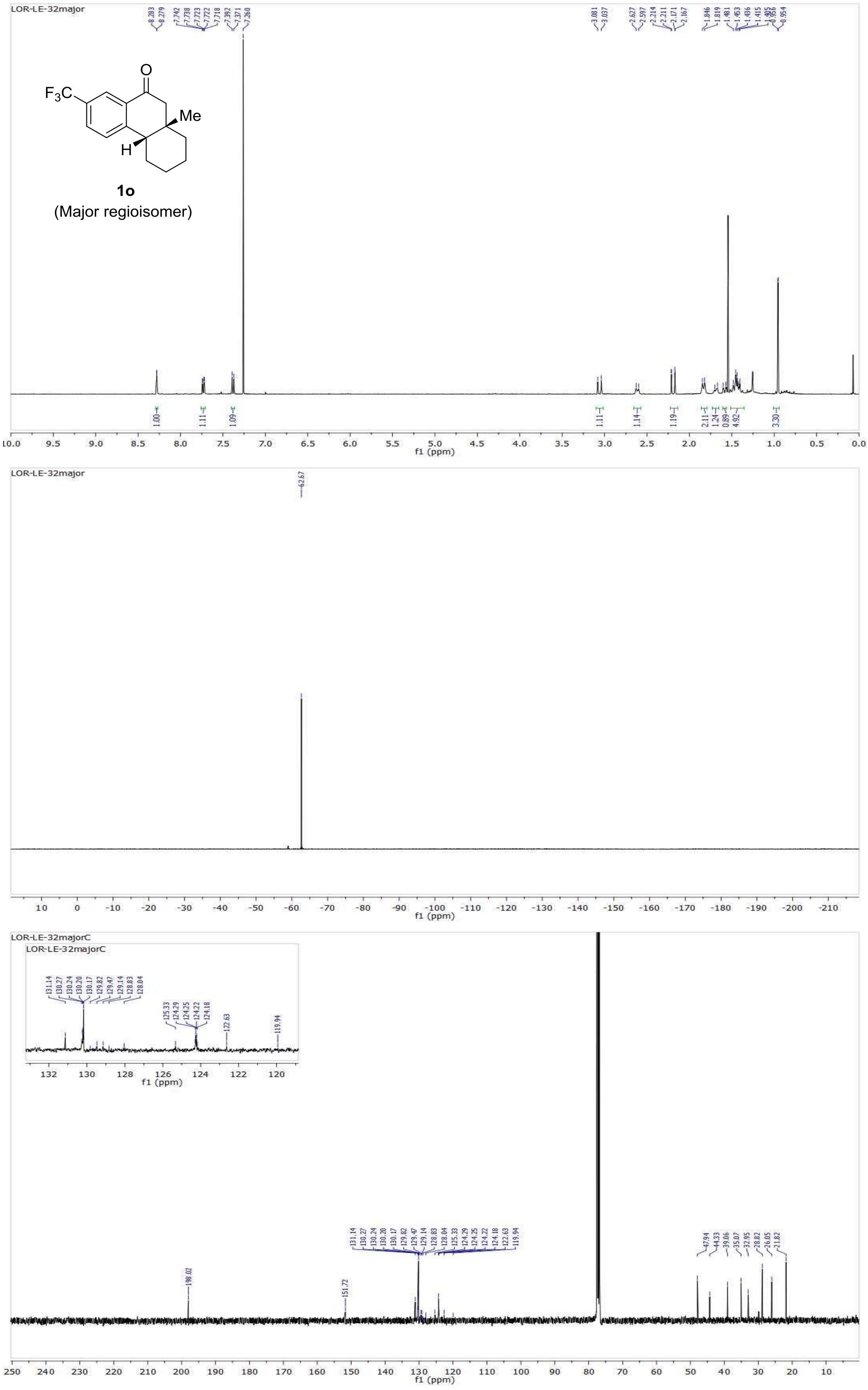

Supplementary Figure 15. ^{1}H, ^{19}F and ^{13}C NMR Spectra of **1o**.

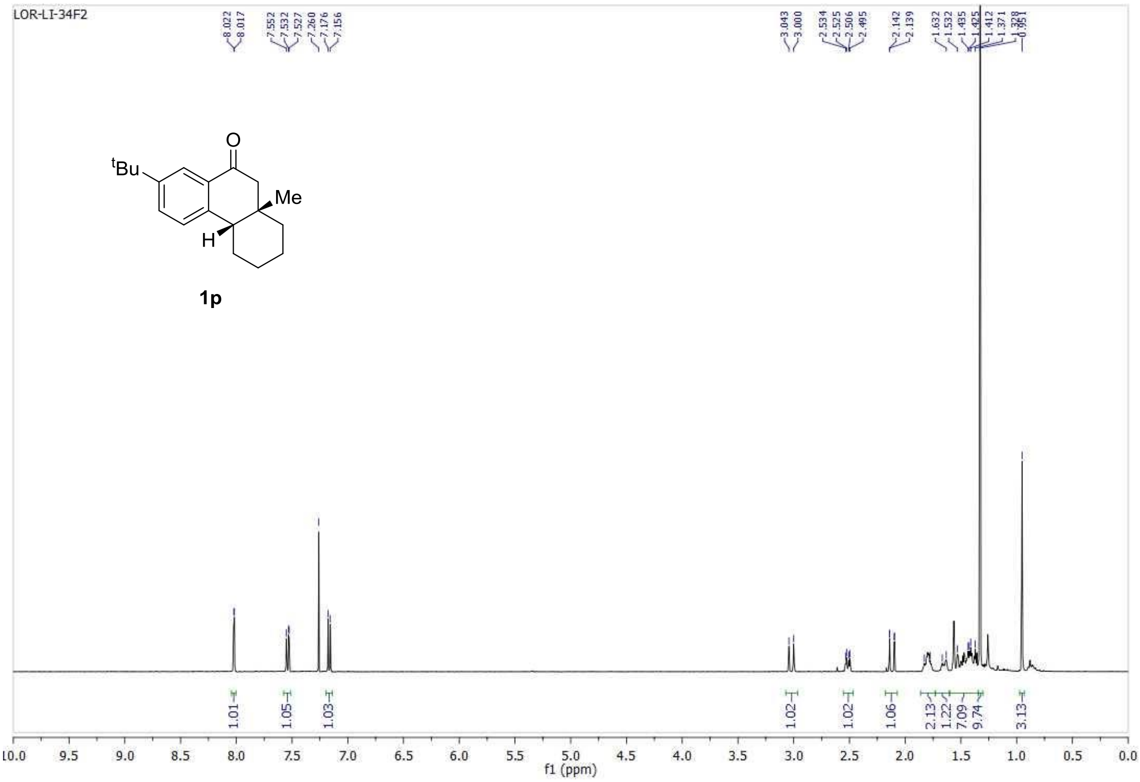

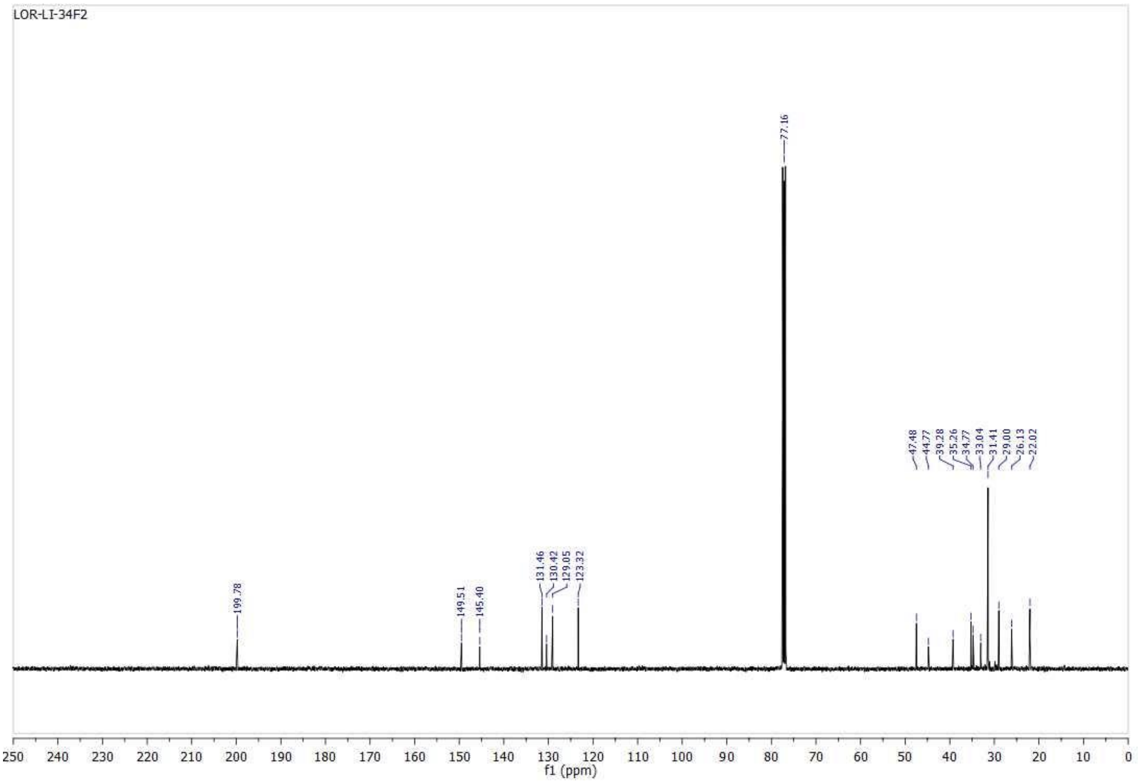

Supplementary Figure 16. ^{1}H and ^{13}C NMR Spectra of **1p**.

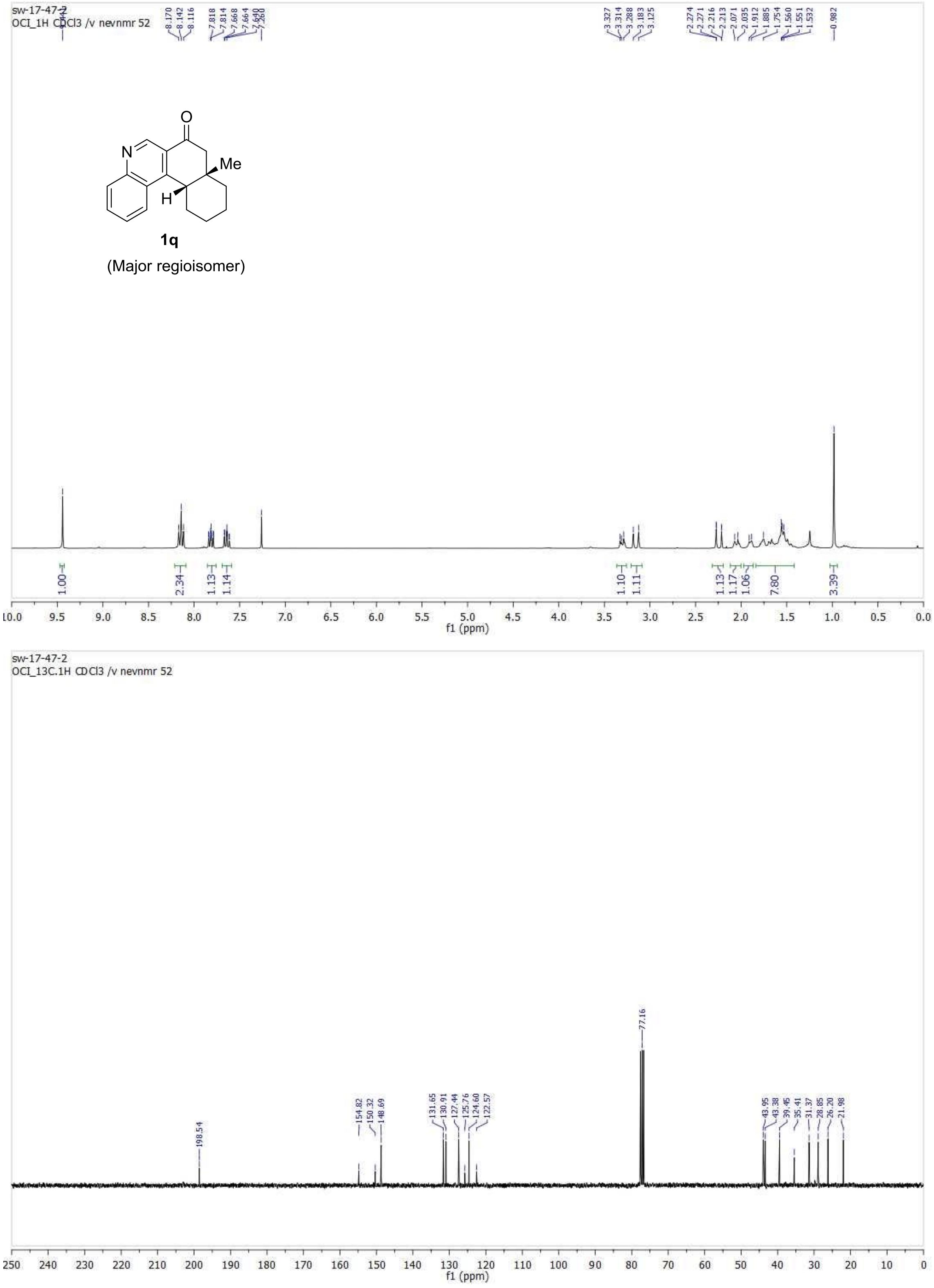

Supplementary Figure 17. ^{1}H and ^{13}C NMR Spectra of **1q**.

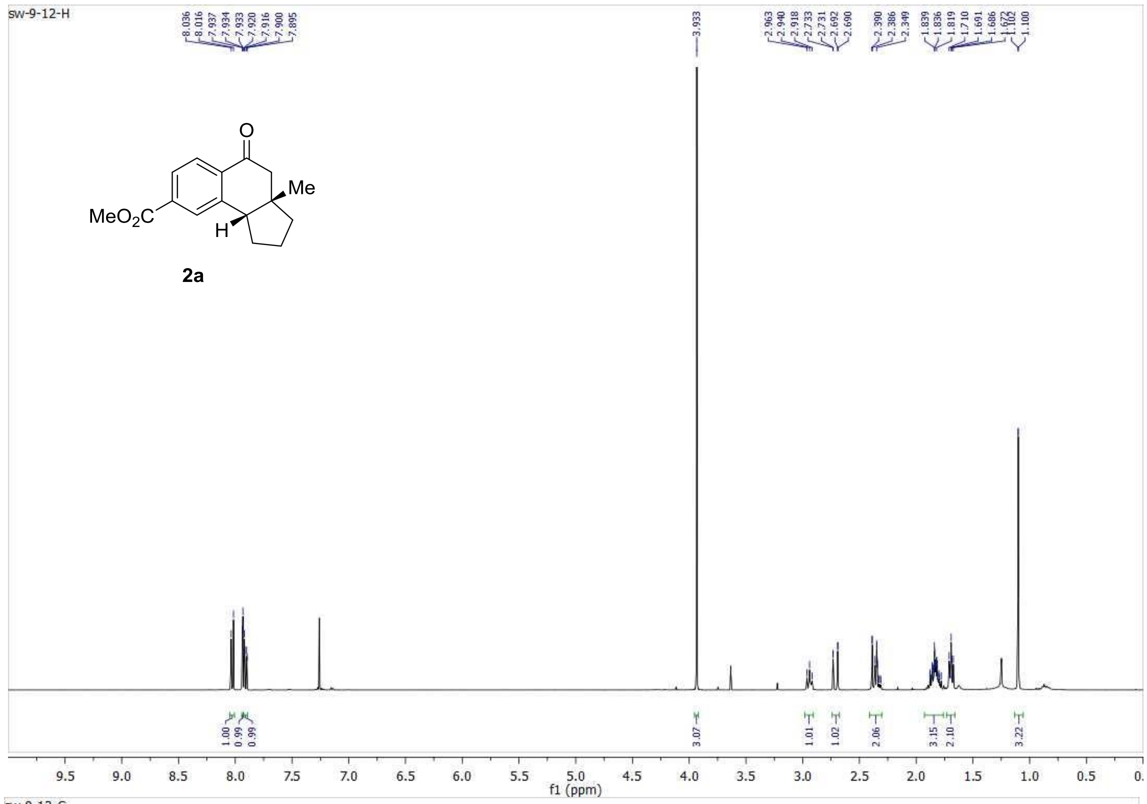

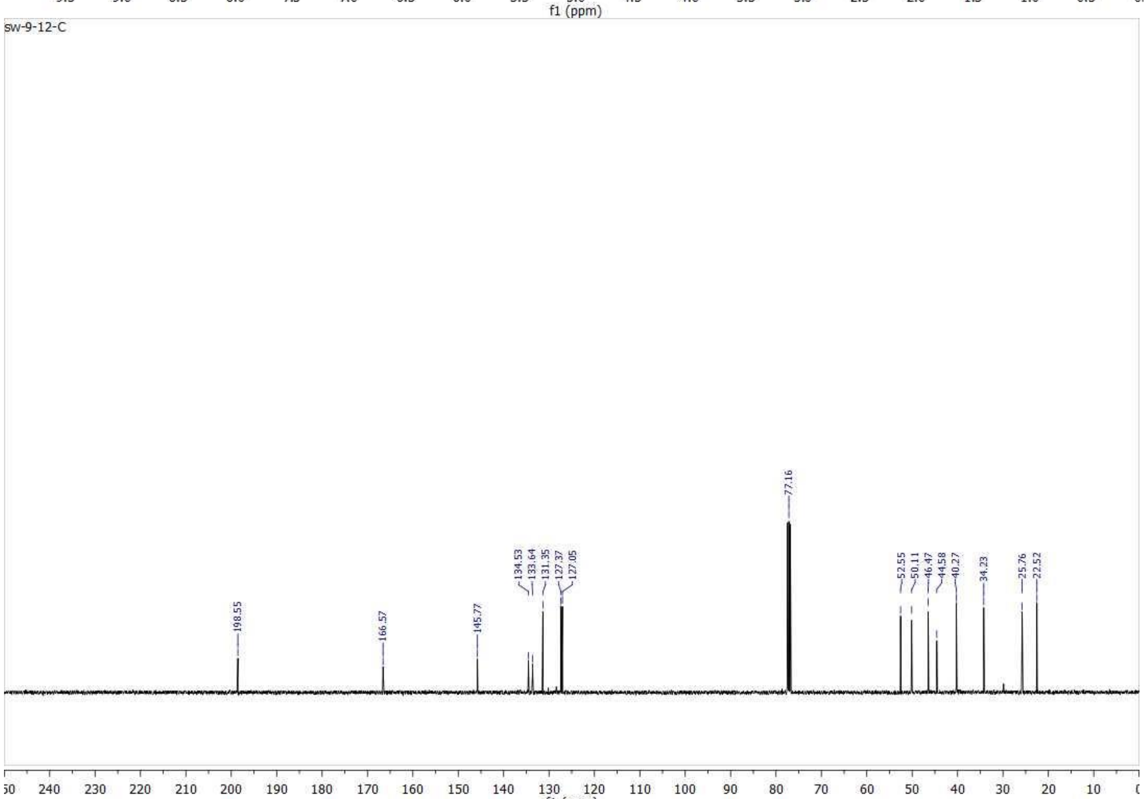

Supplementary Figure 18. ^{1}H and ^{13}C NMR Spectra of **2a**.

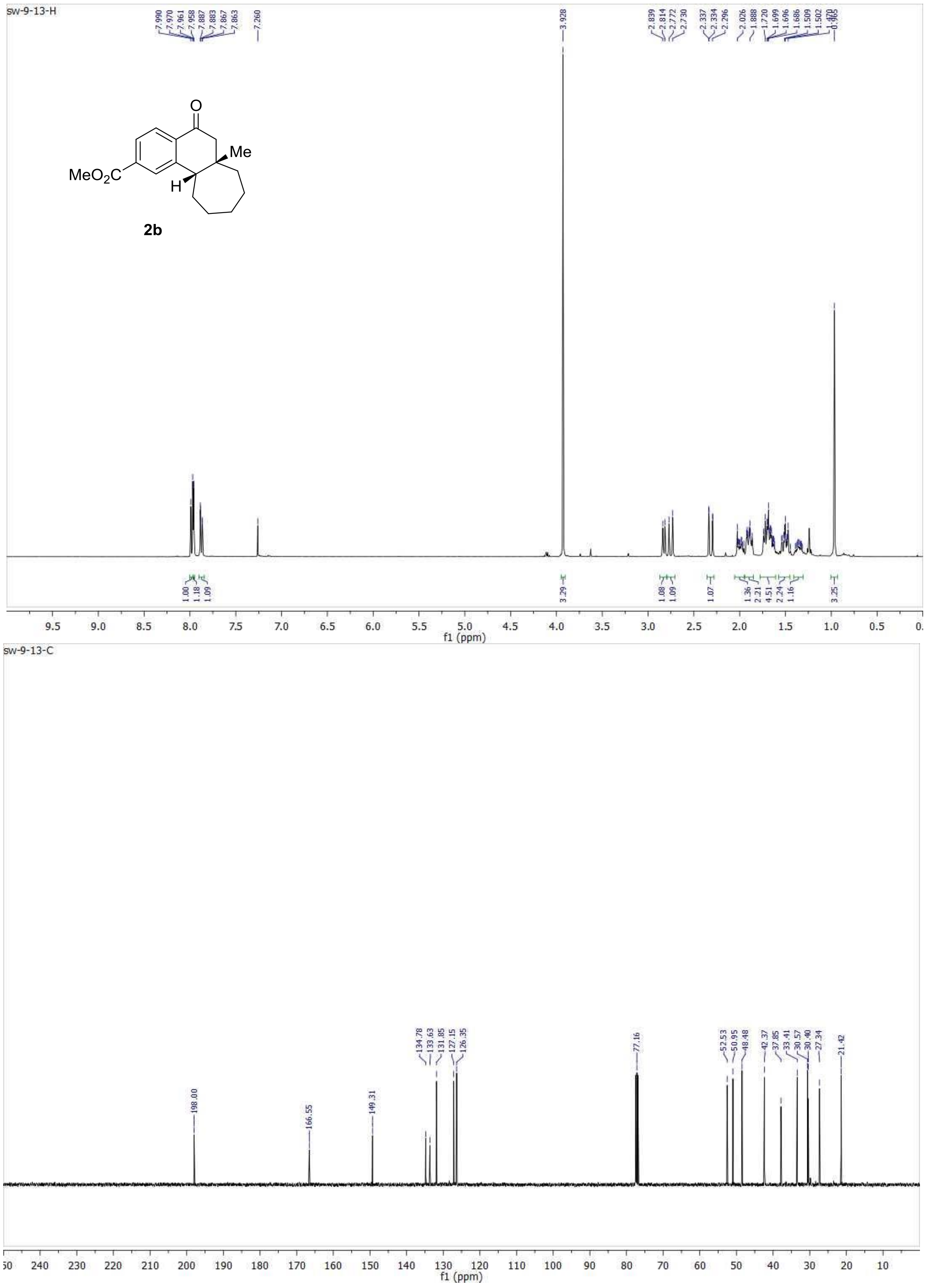

Supplementary Figure 19. ^{1}H and ^{13}C NMR Spectra of **2b**.

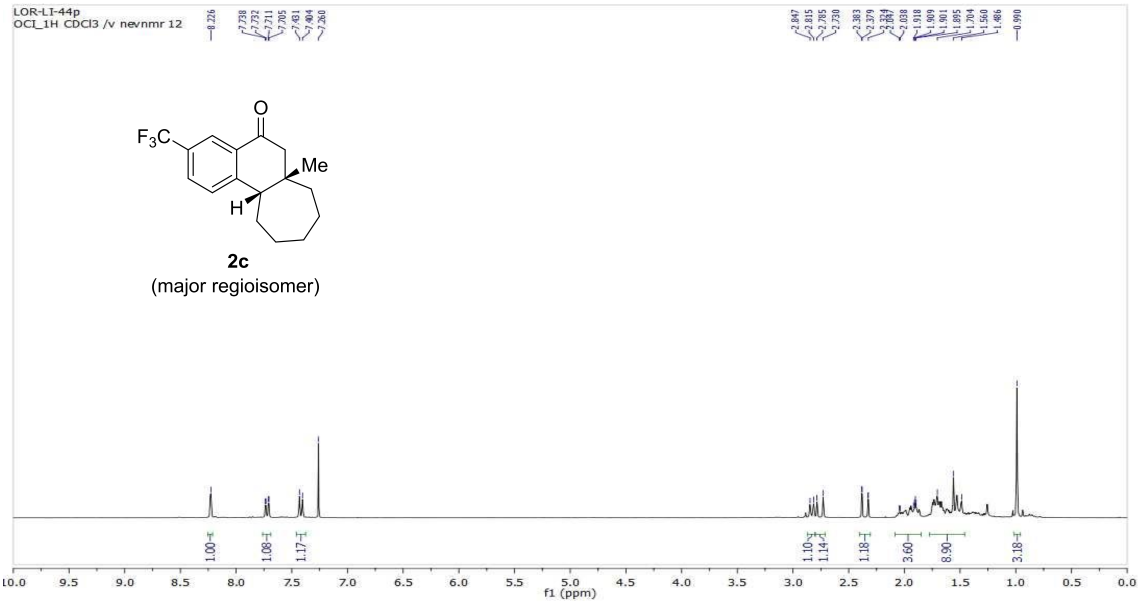

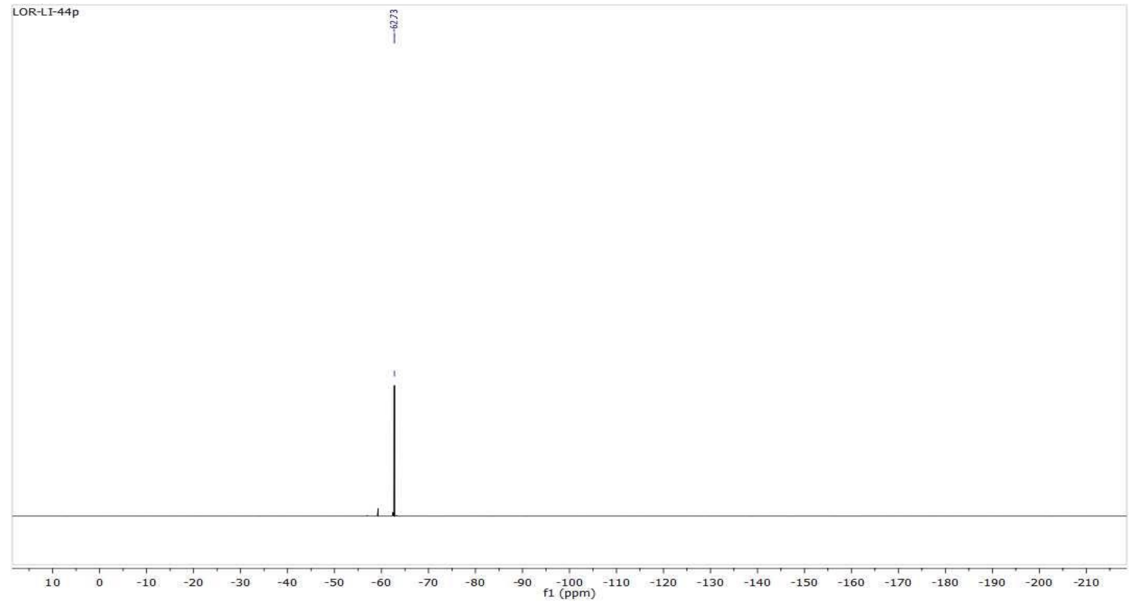

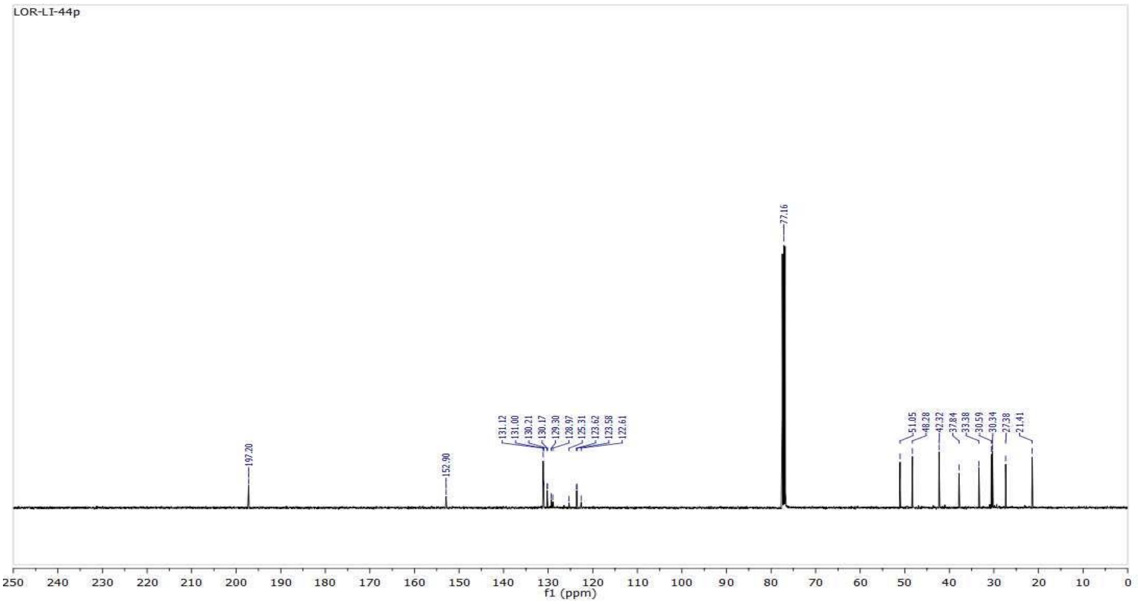

Supplementary Figure 20. ^{1}H, ^{19}F and ^{13}C NMR Spectra of **2c**.

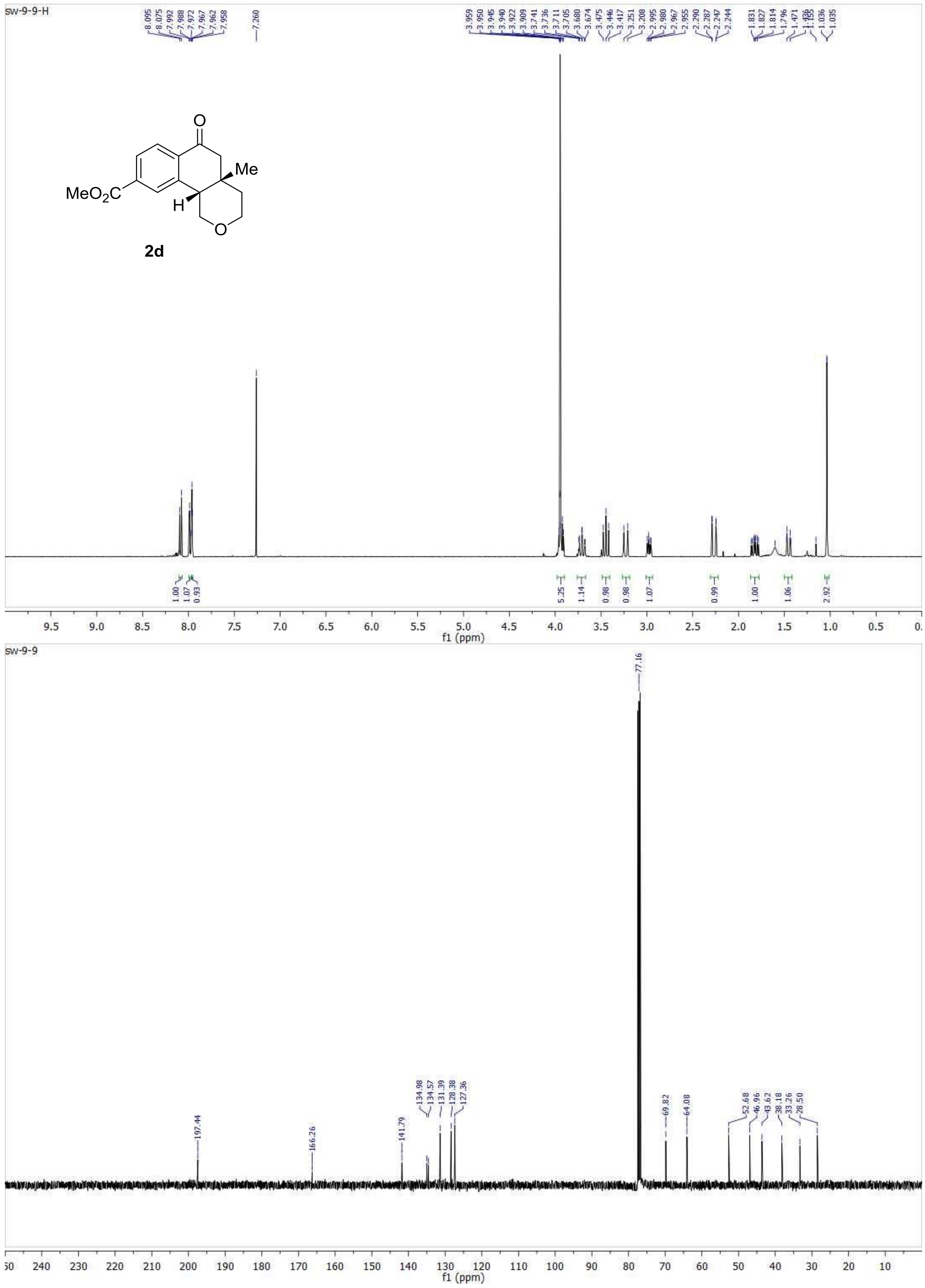

Supplementary Figure 21. ^{1}H and ^{13}C NMR Spectra of **2d**.

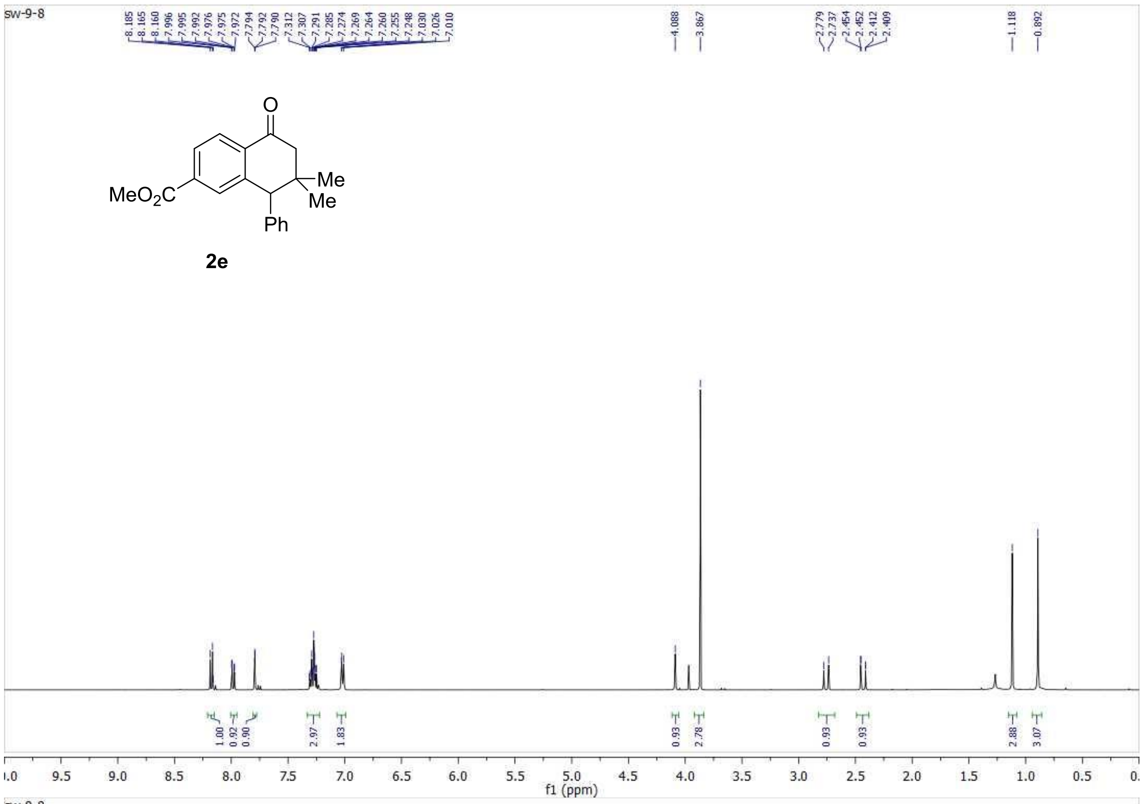

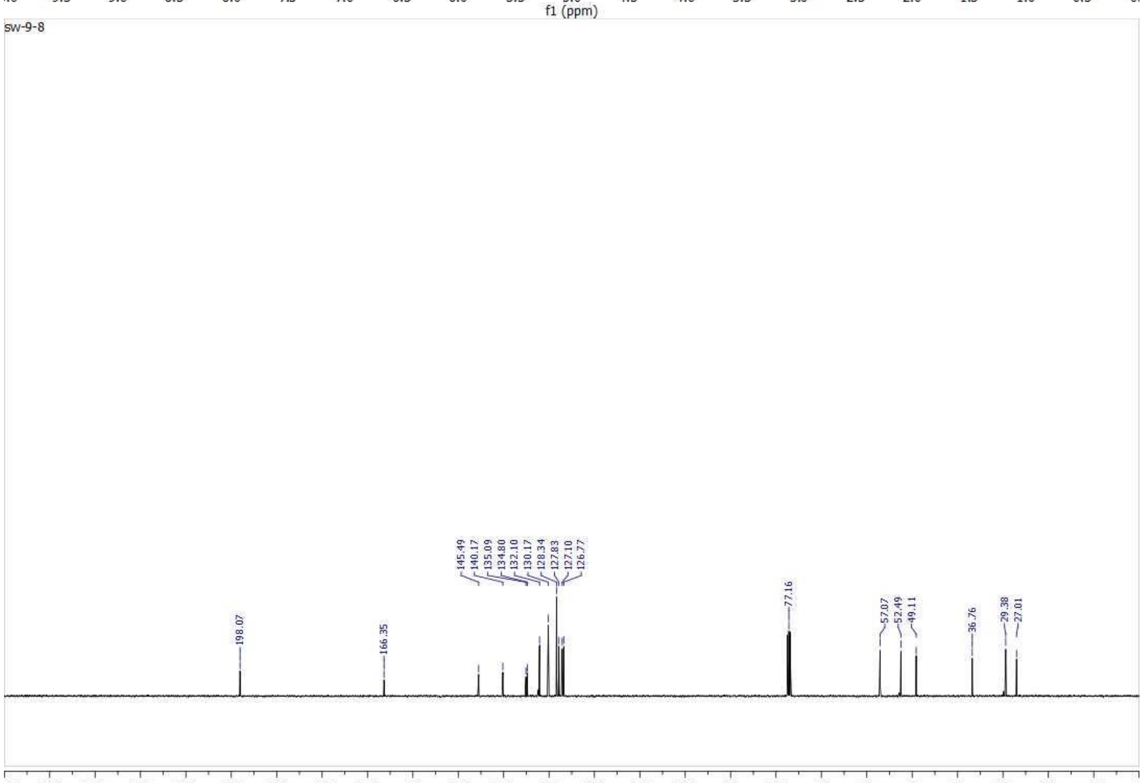

Supplementary Figure 22. ^{1}H and ^{13}C NMR Spectra of **2e**.

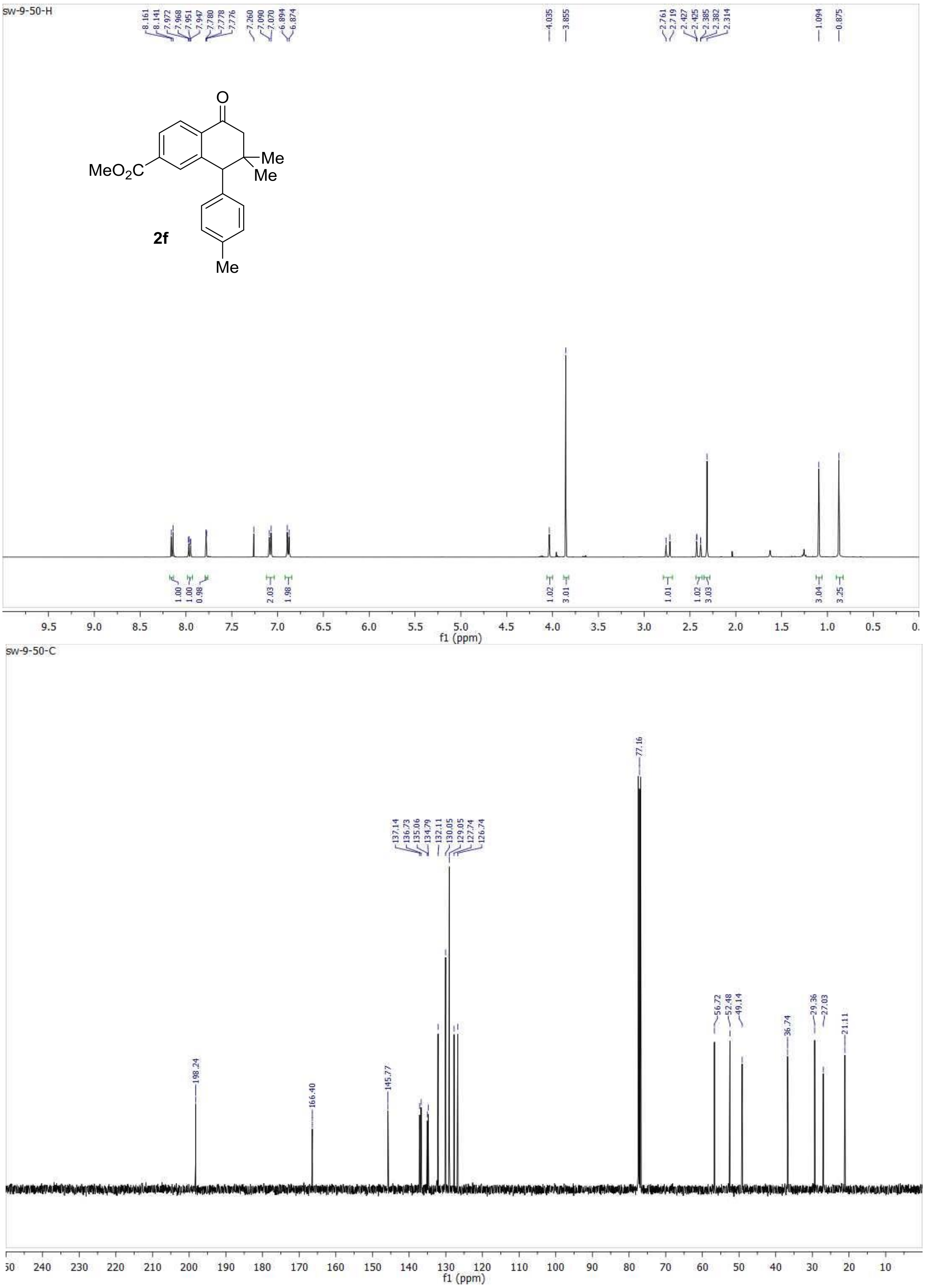

Supplementary Figure 23. ^{1}H and ^{13}C NMR Spectra of **2f**.

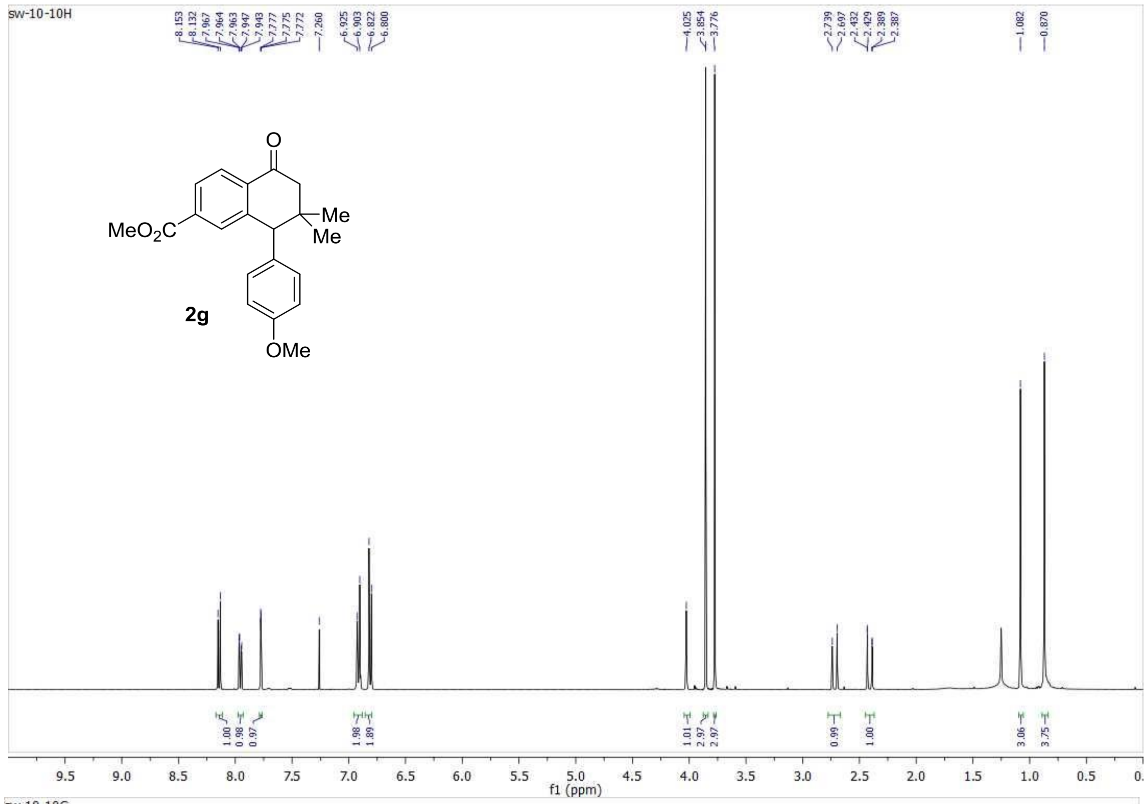

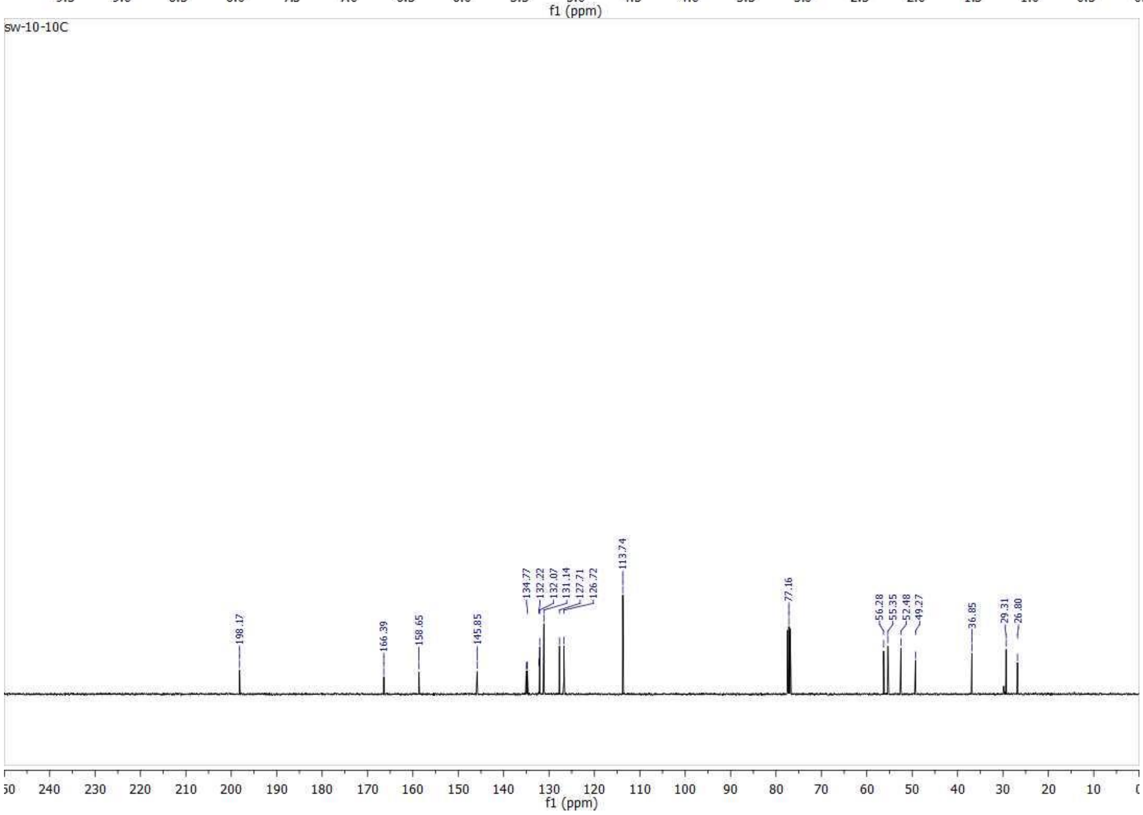

Supplementary Figure 24. ^{1}H and ^{13}C NMR Spectra of **2g**.

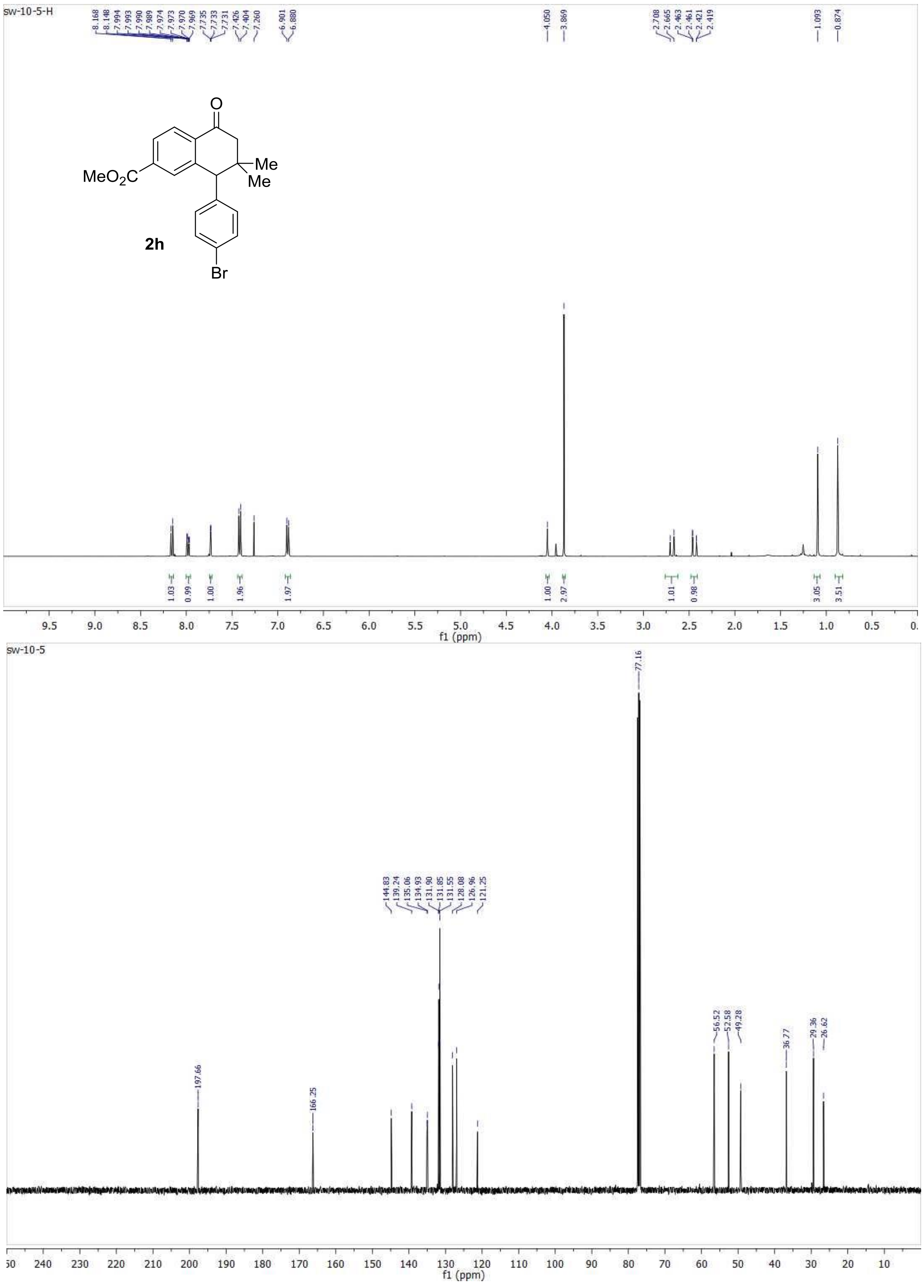

Supplementary Figure 25. ^{1}H and ^{13}C NMR Spectra of **2h**.

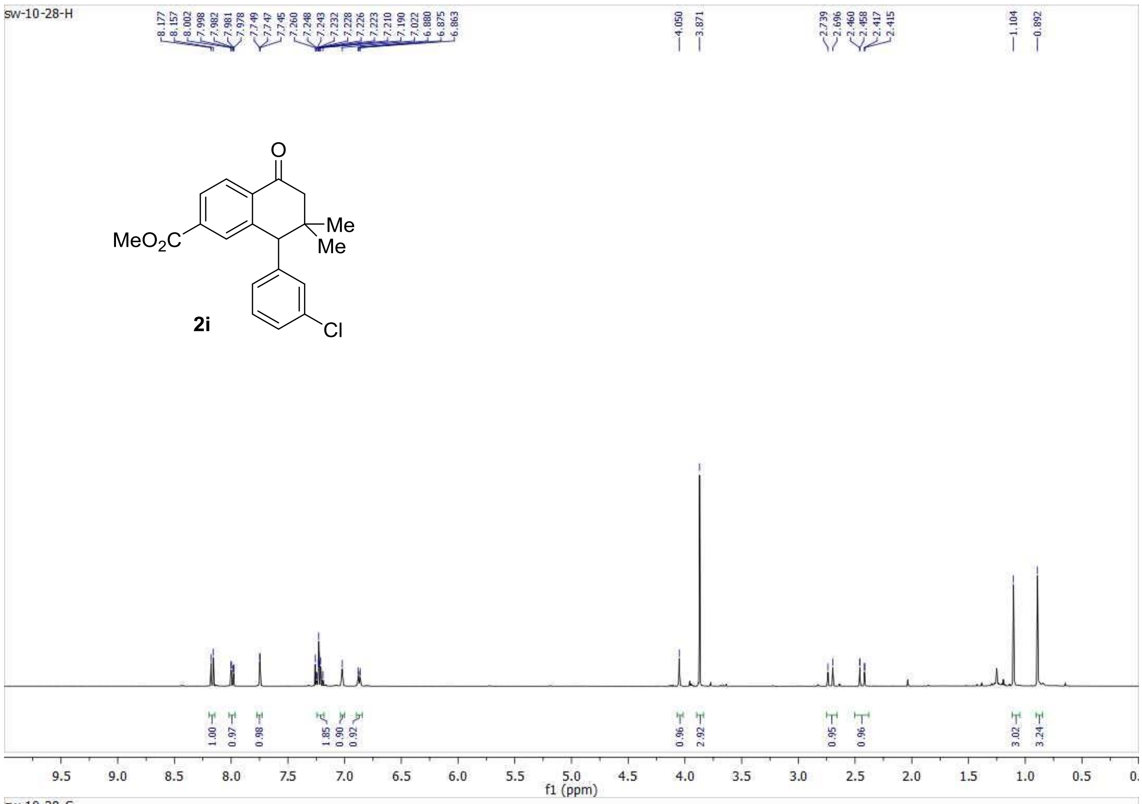

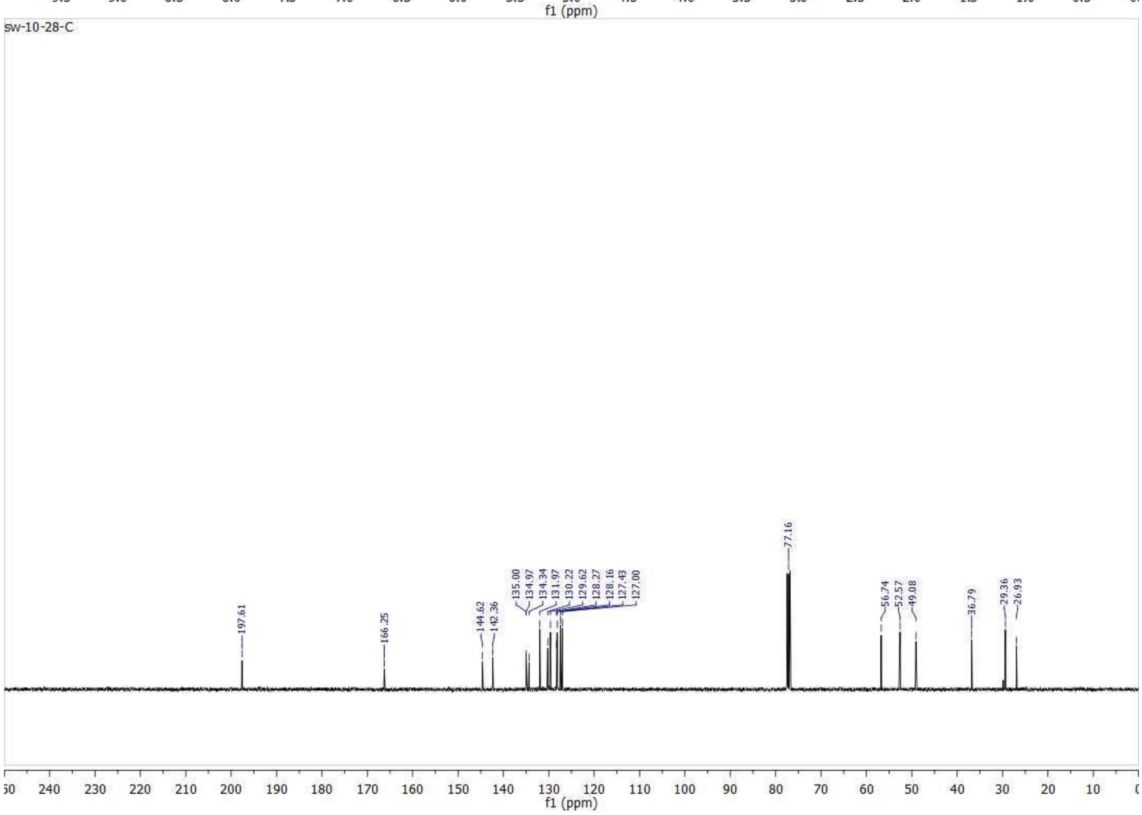

Supplementary Figure 26. ^{1}H and ^{13}C NMR Spectra of **2i**.

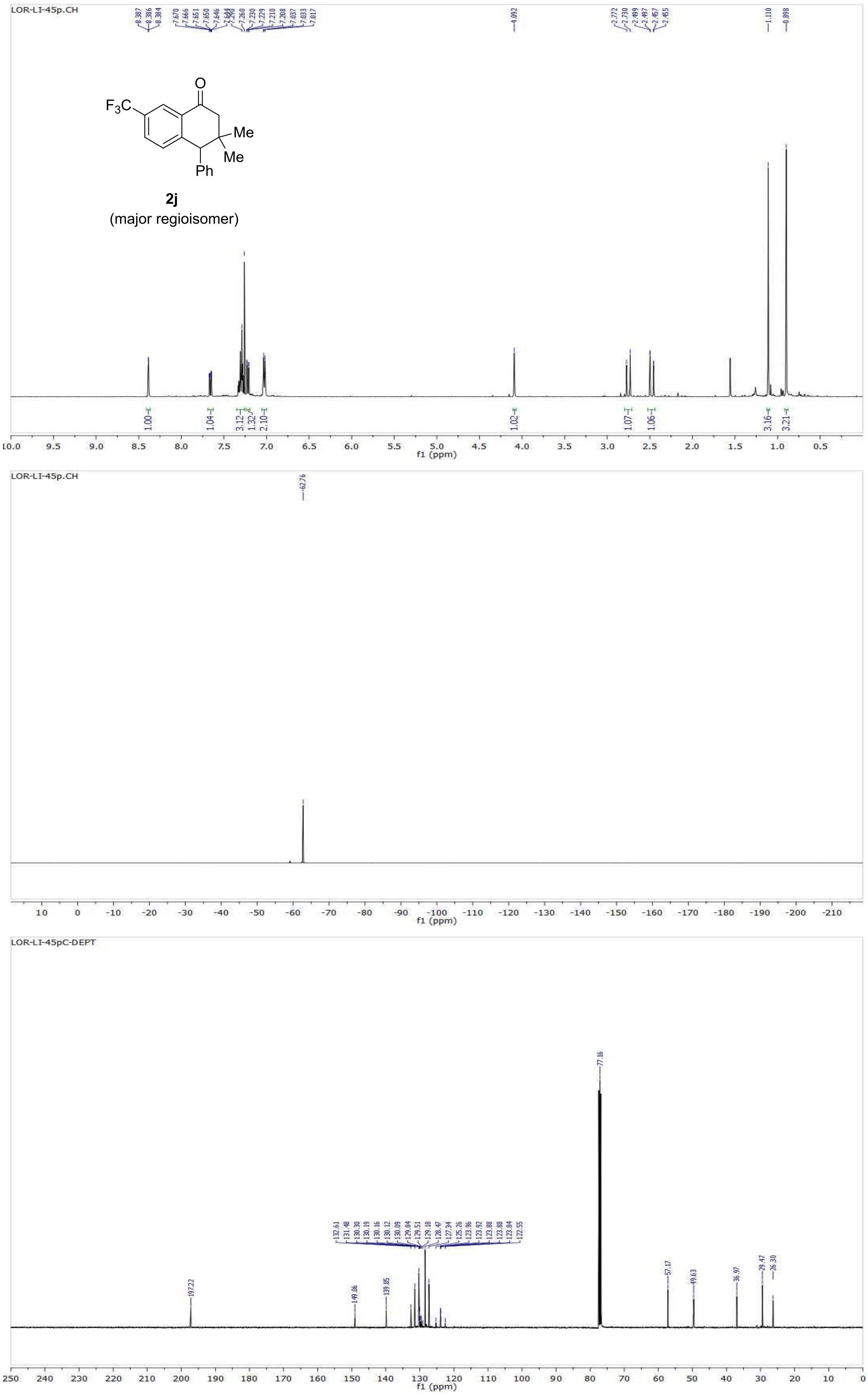

Supplementary Figure 27. ^{1}H, ^{19}F and ^{13}C NMR Spectra of **2j**.

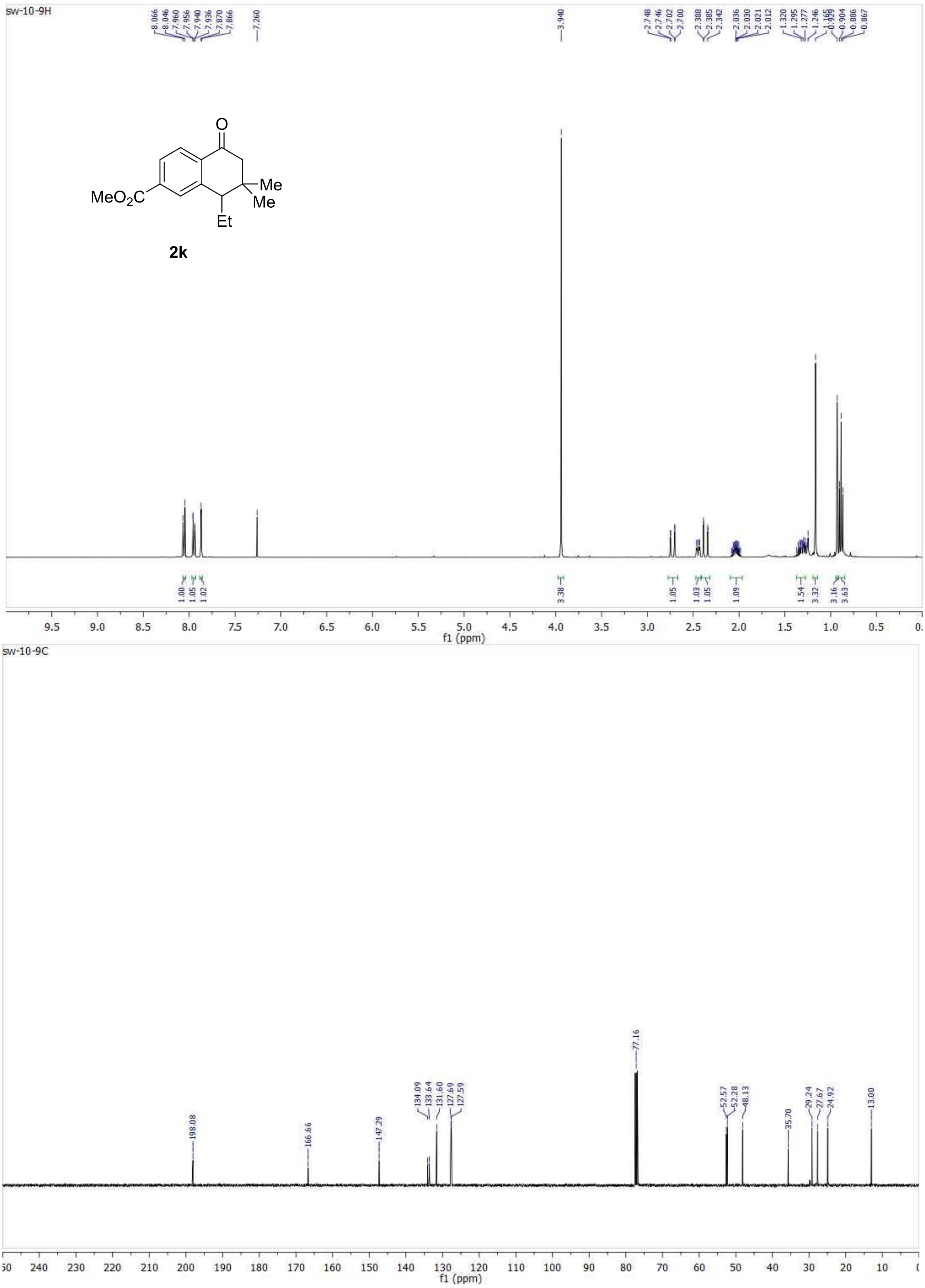

Supplementary Figure 28. ^{1}H and ^{13}C NMR Spectra of **2k**.

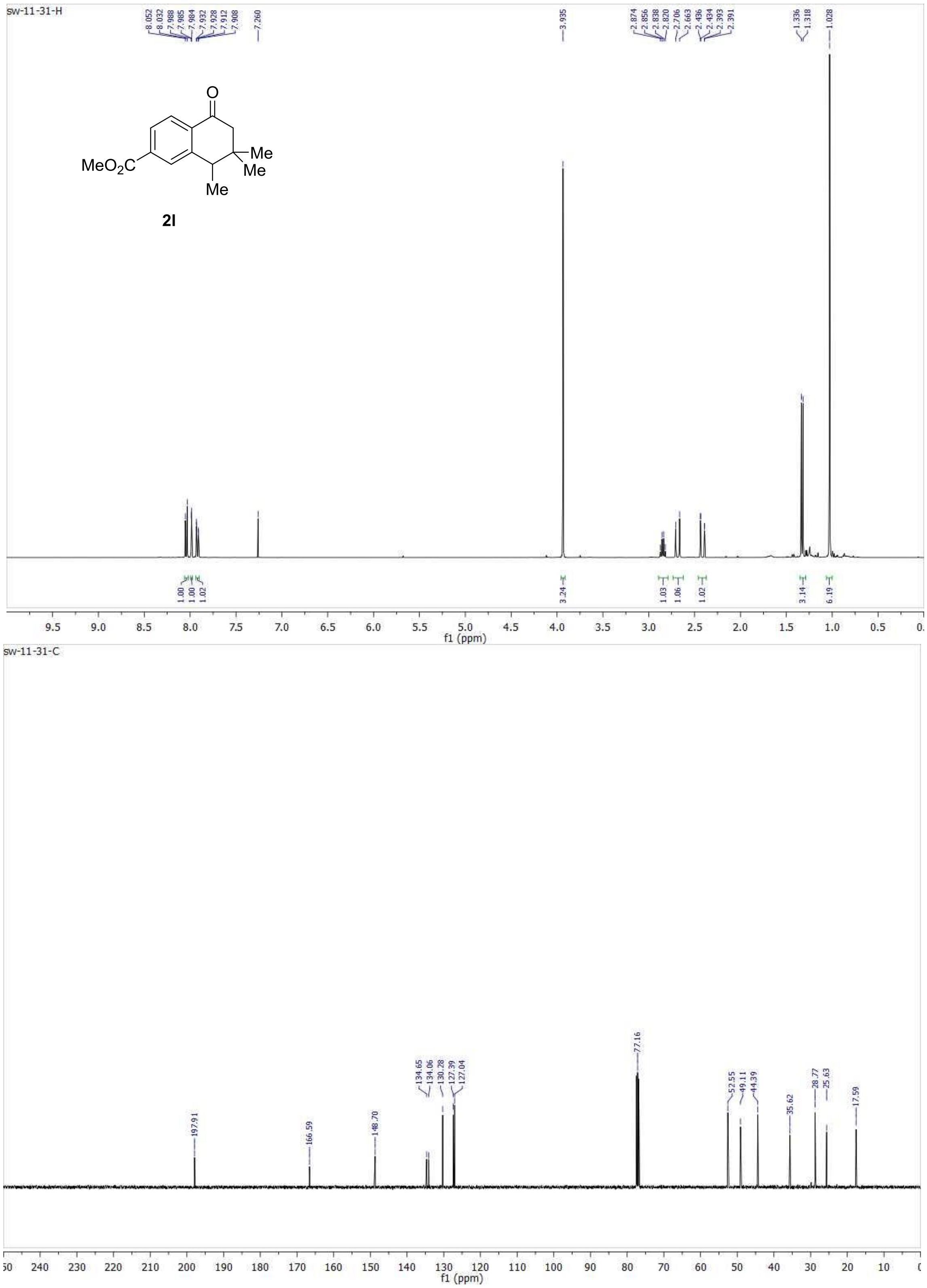

Supplementary Figure 29. ^{1}H and ^{13}C NMR Spectra of **2l**.

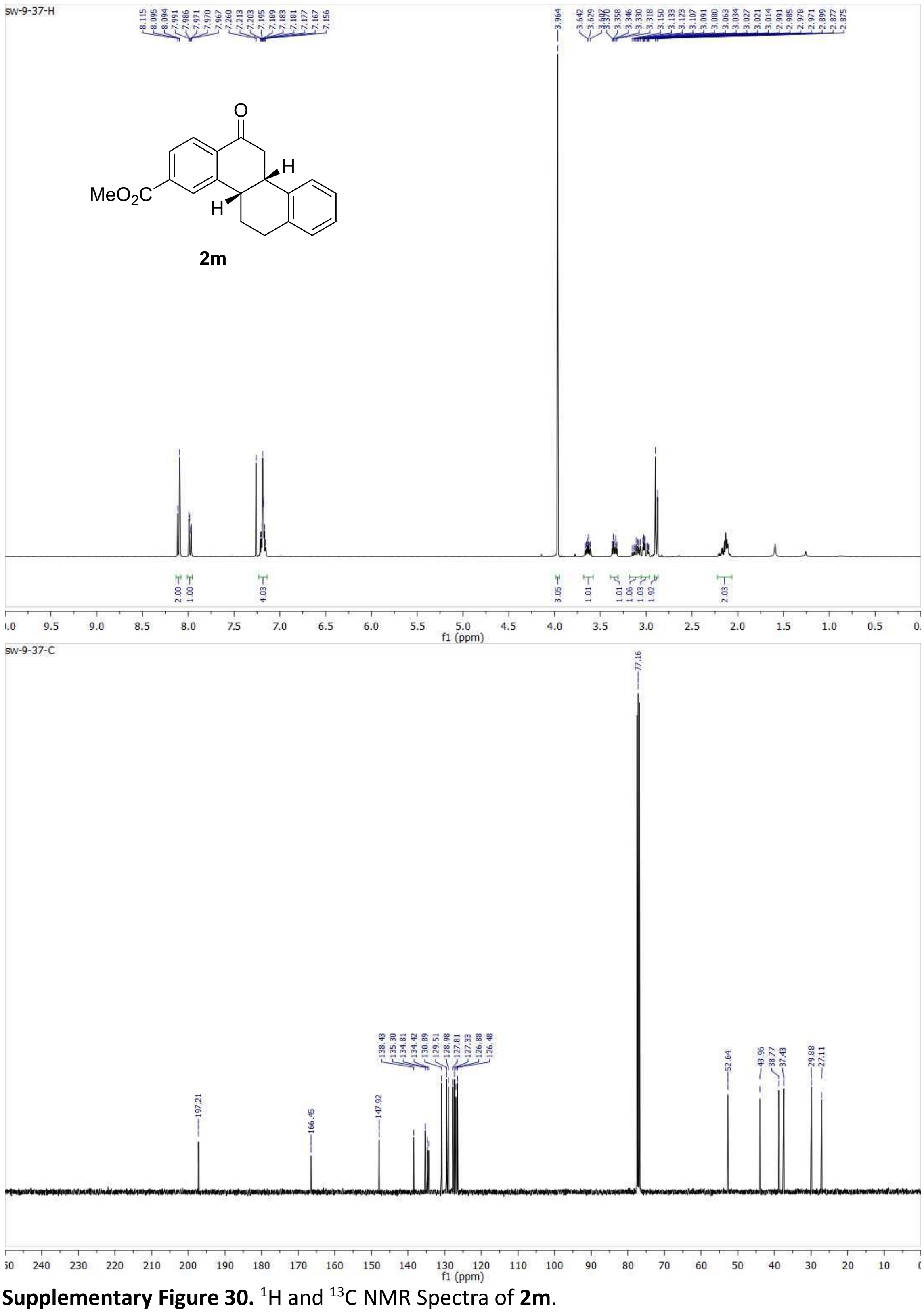

Supplementary Figure 30. ^{1}H and ^{13}C NMR Spectra of **2m**.

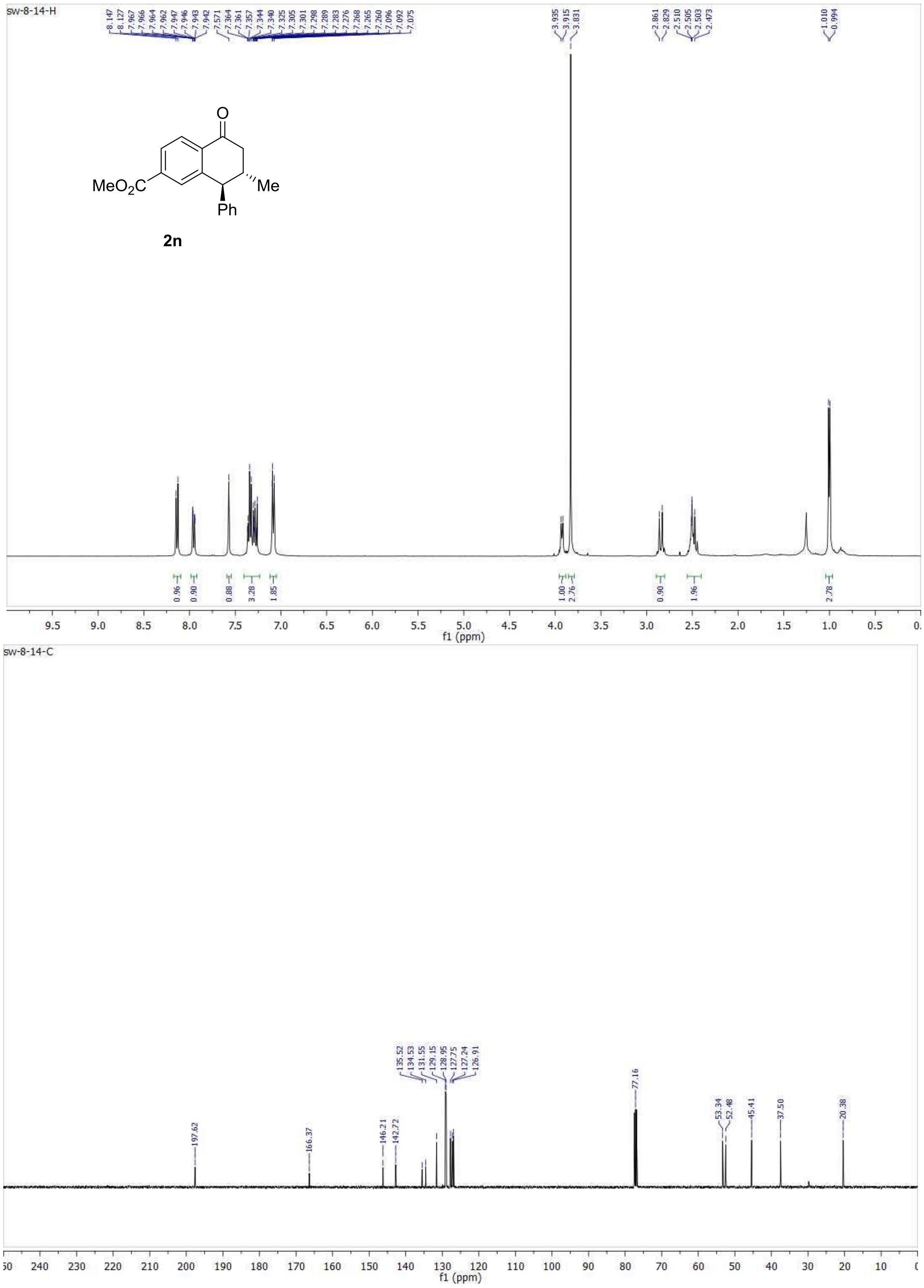

Supplementary Figure 31. ^{1}H and ^{13}C NMR Spectra of **2n**.

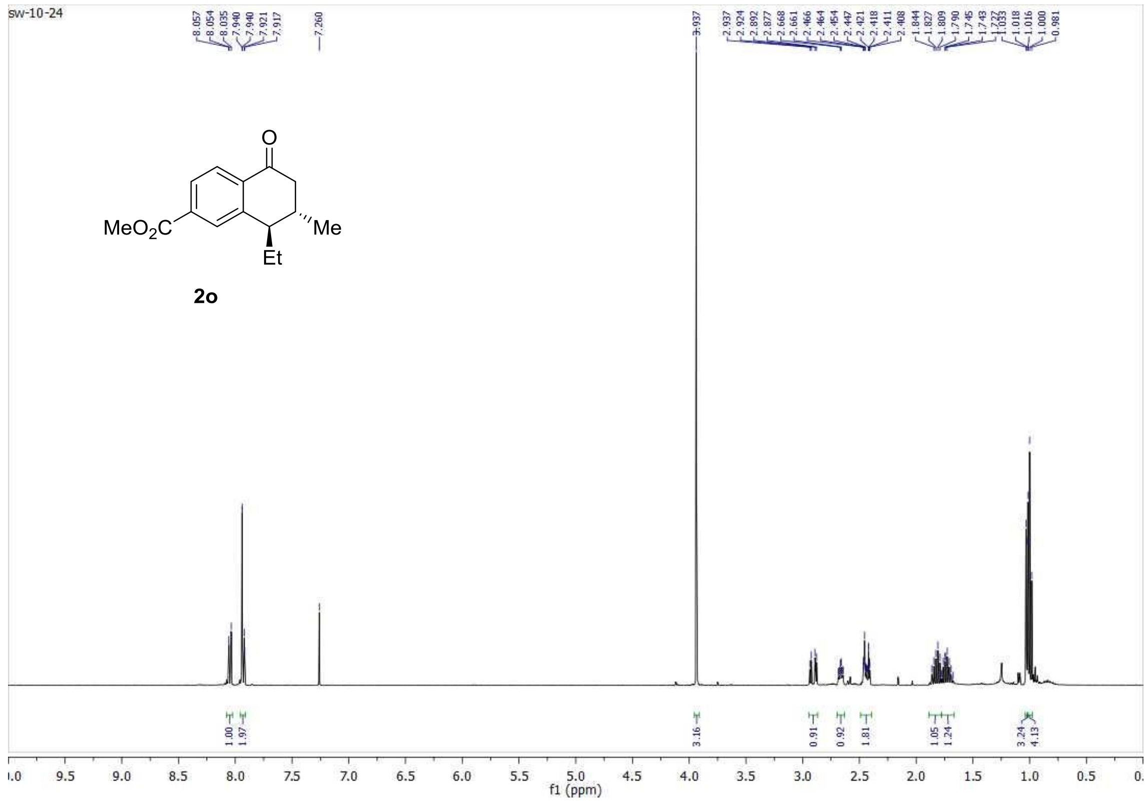

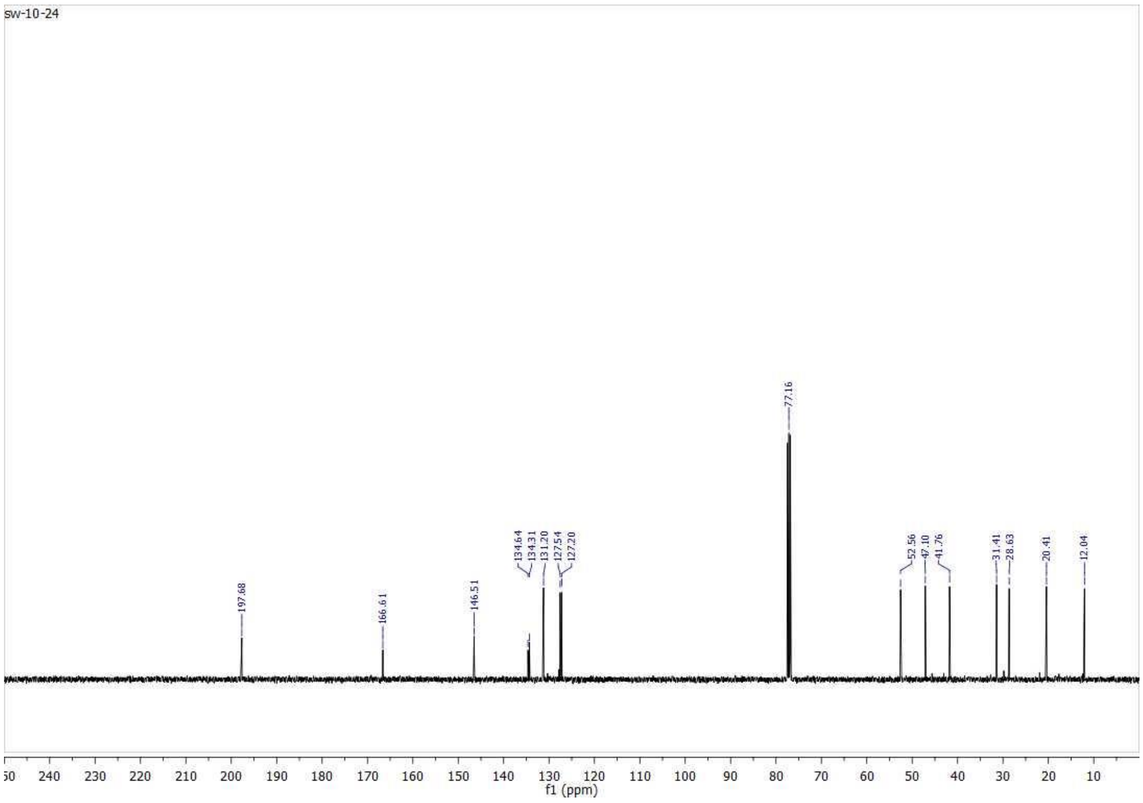

Supplementary Figure 32. ^{1}H and ^{13}C NMR Spectra of **2o**.

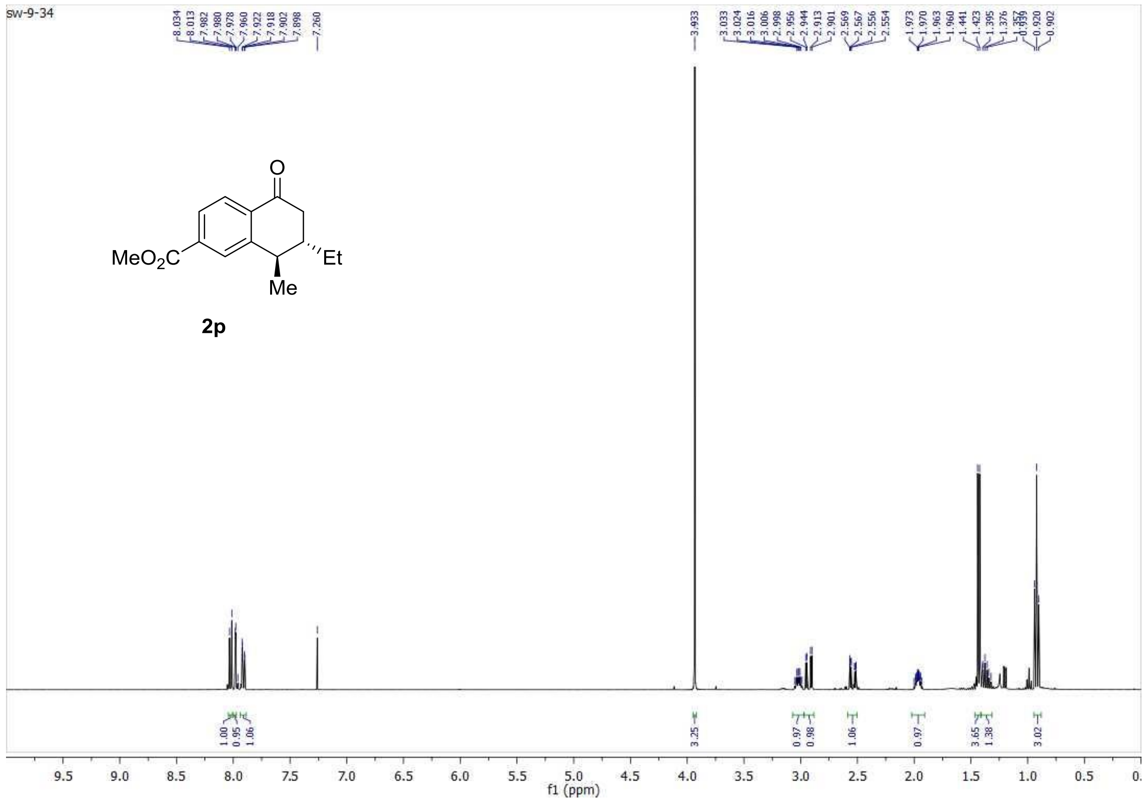

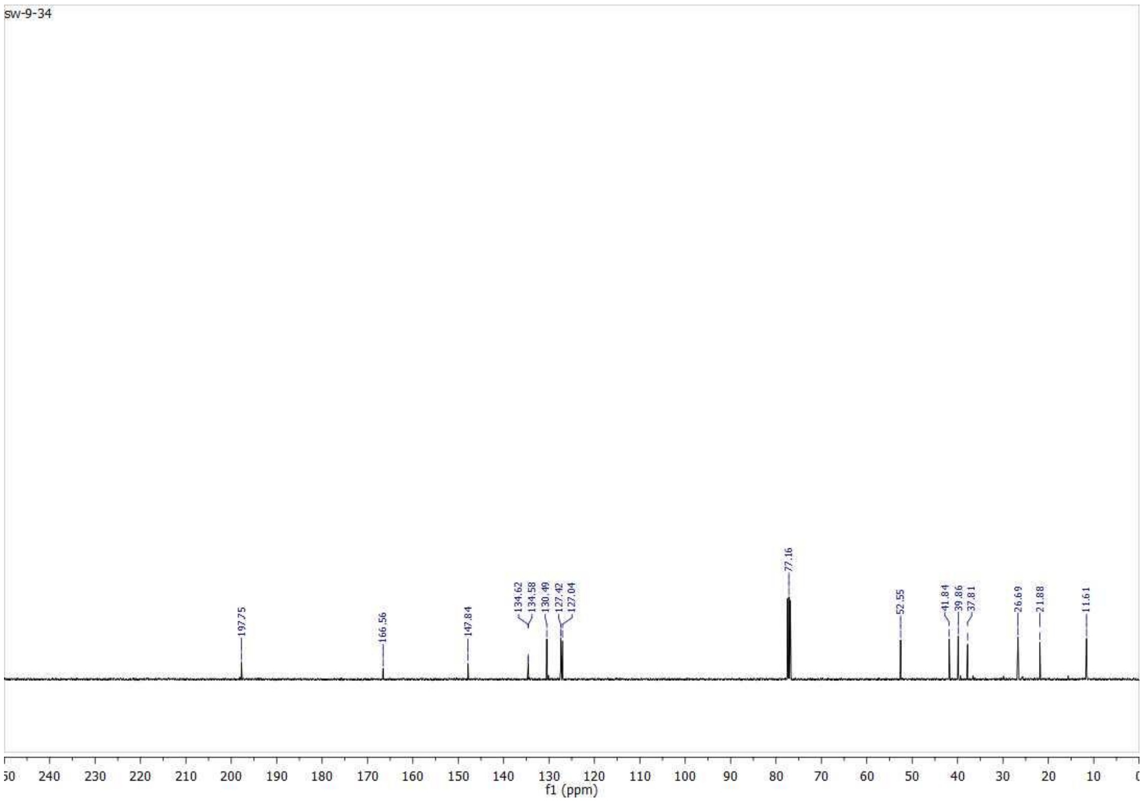

Supplementary Figure 33. ^{1}H and ^{13}C NMR Spectra of **2p**.

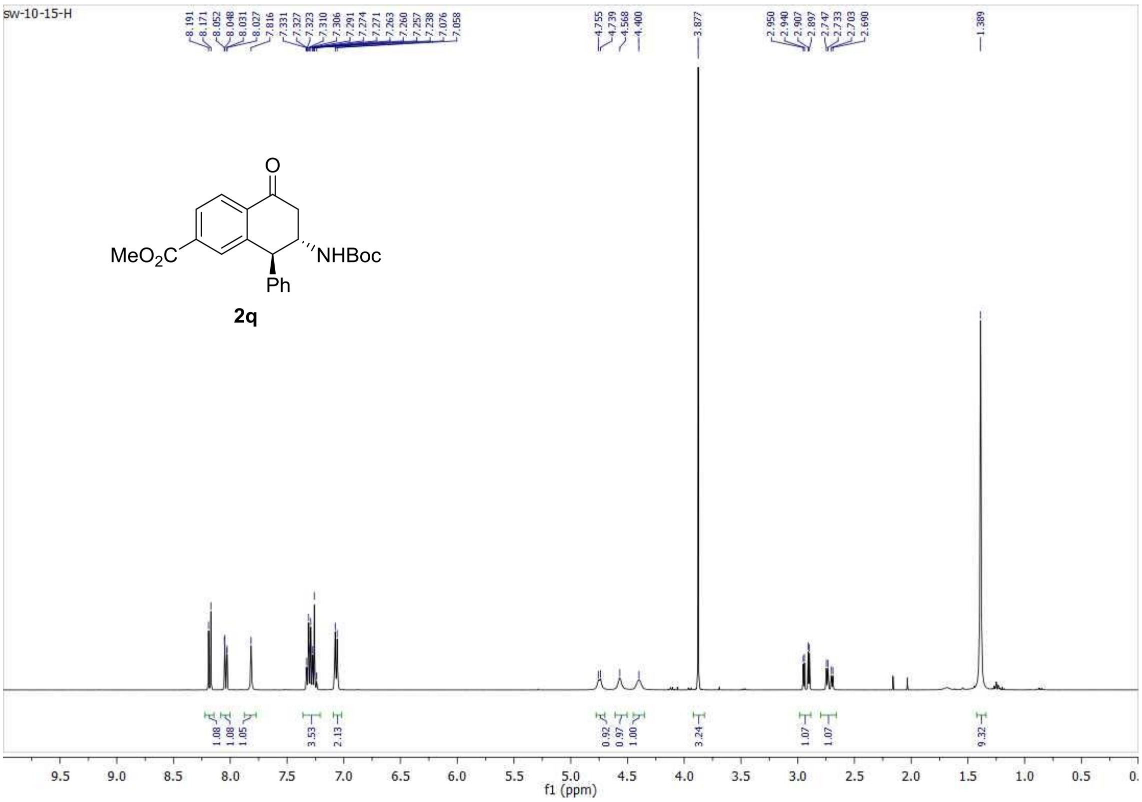

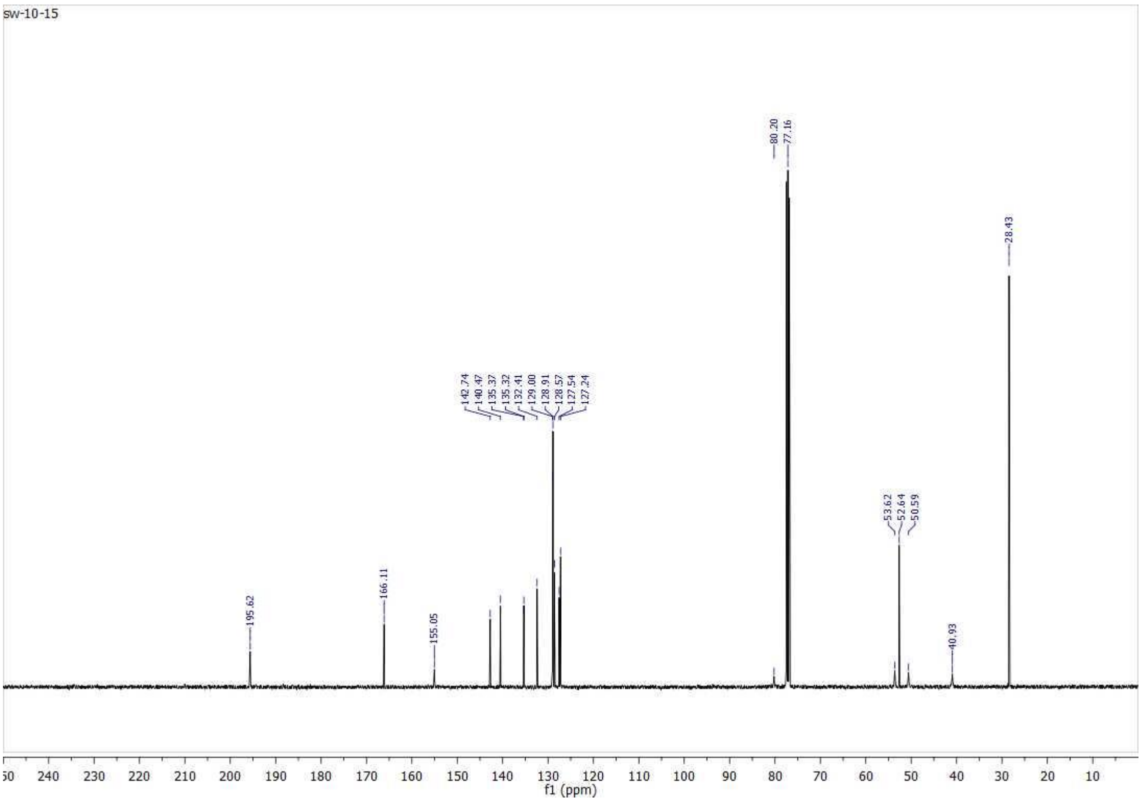

Supplementary Figure 34. ^{1}H and ^{13}C NMR Spectra of **2q**.

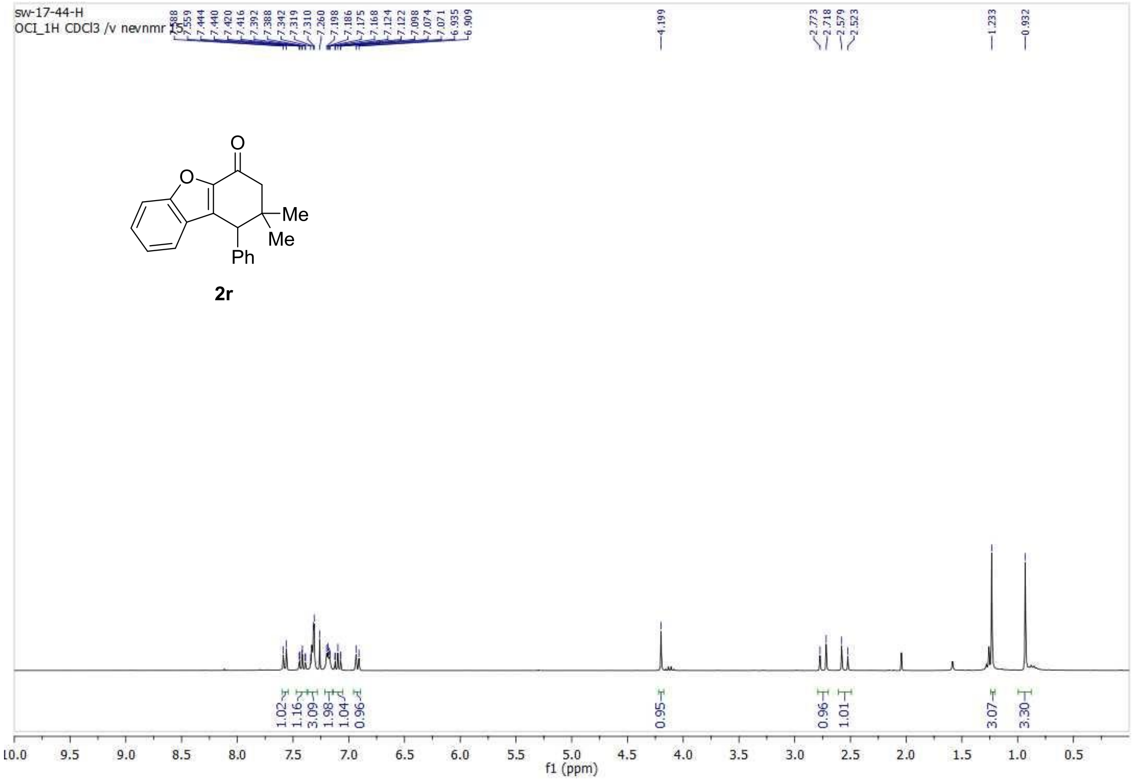

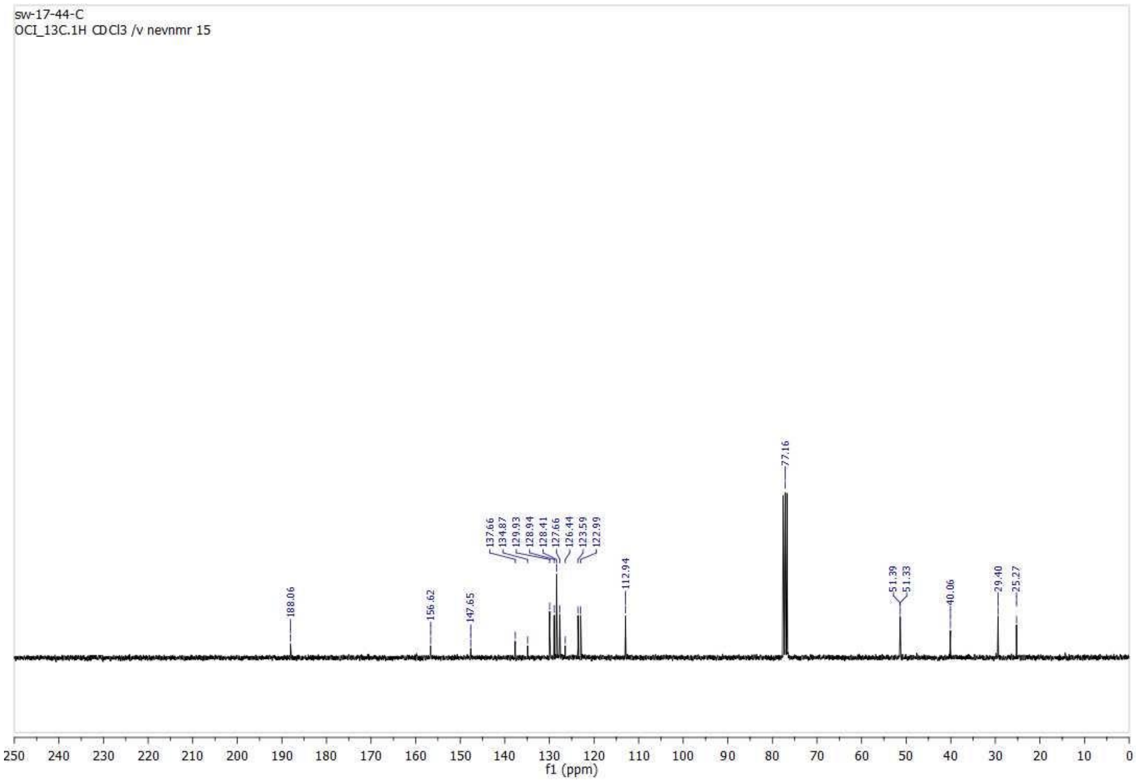

Supplementary Figure 35. ^{1}H and ^{13}C NMR Spectra of **2r**.

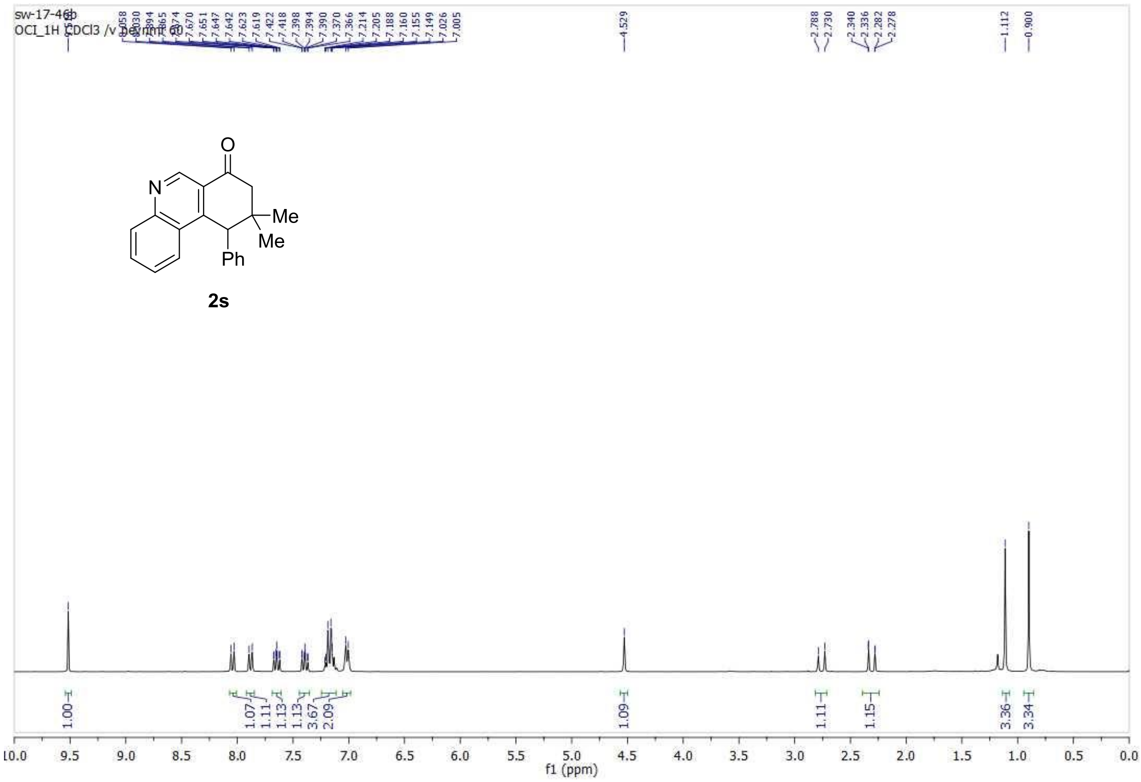

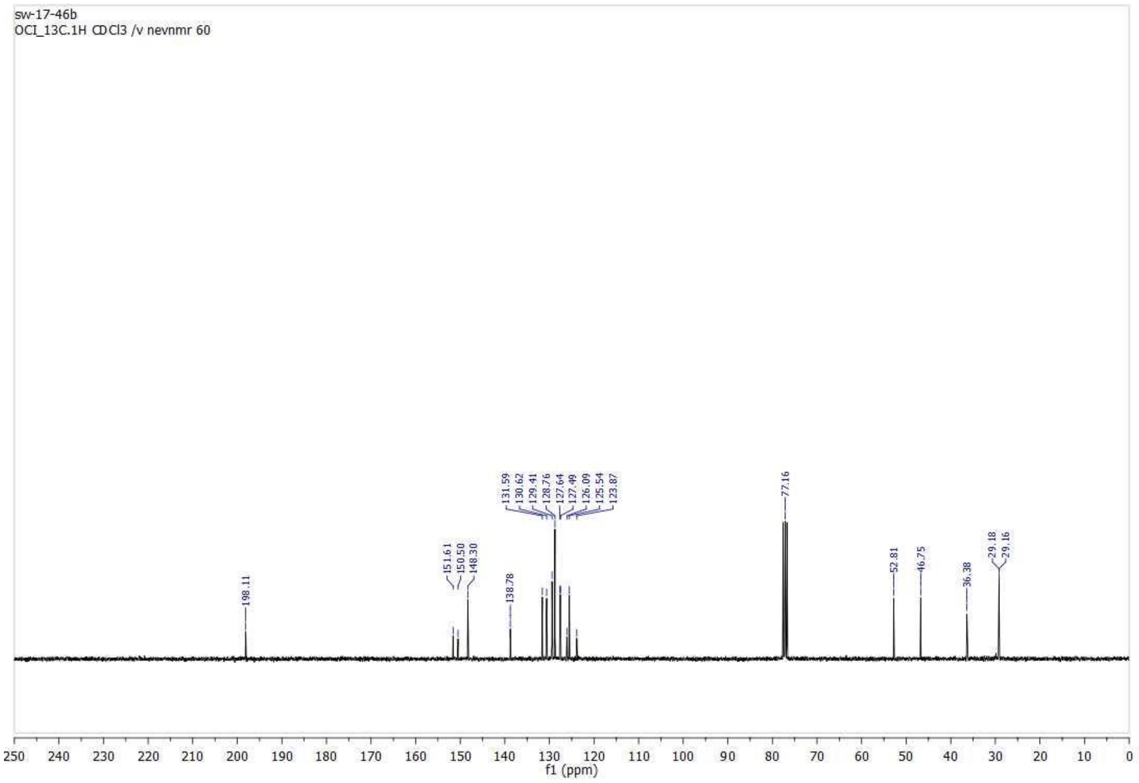

Supplementary Figure 36. ^{1}H and ^{13}C NMR Spectra of **2s**.

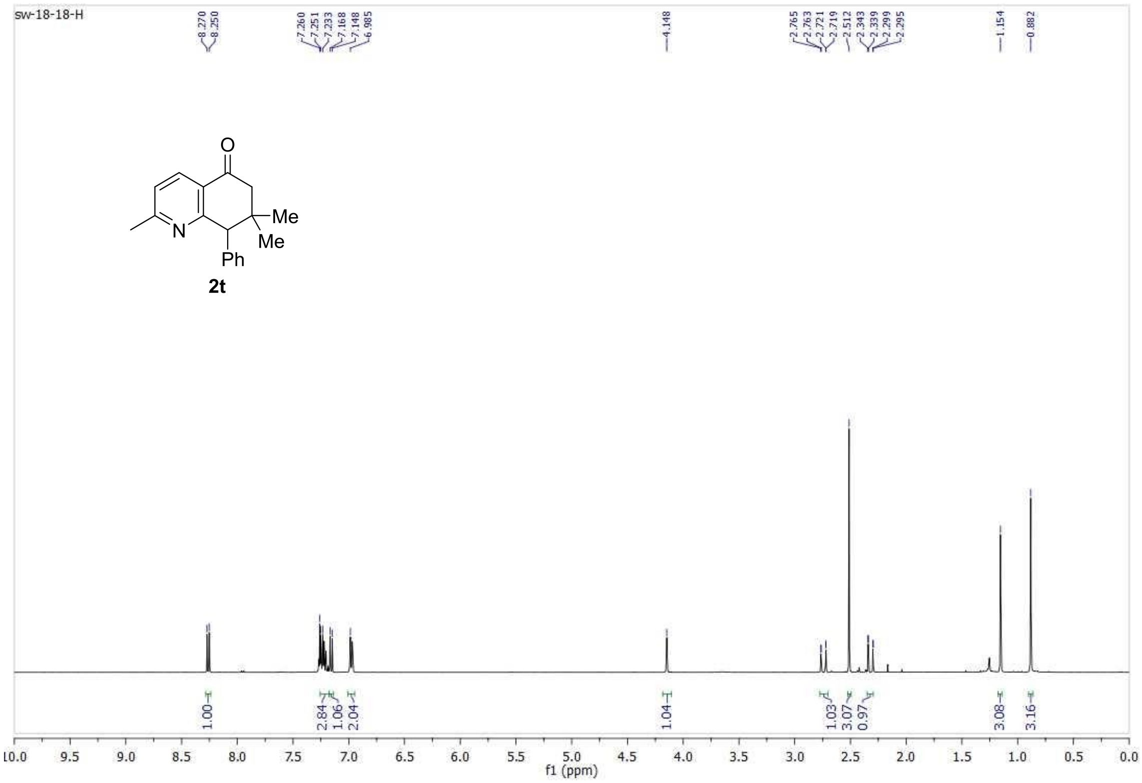

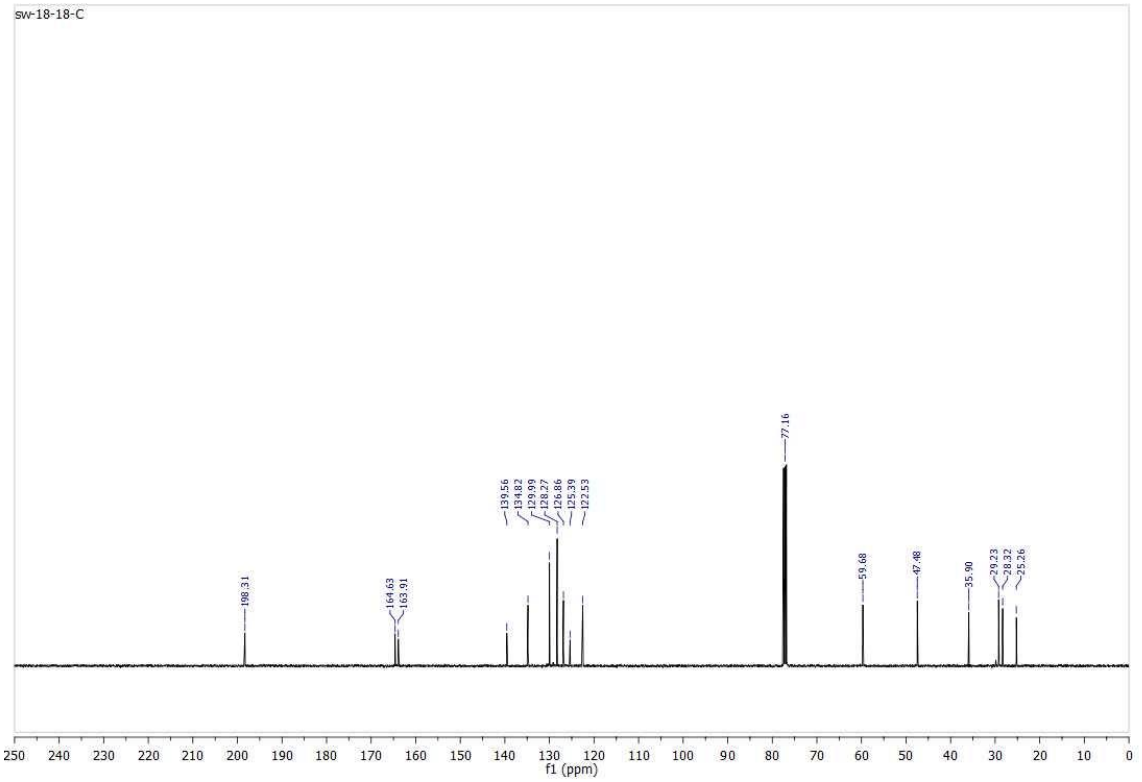

Supplementary Figure 37. ^{1}H and ^{13}C NMR Spectra of **2t**.

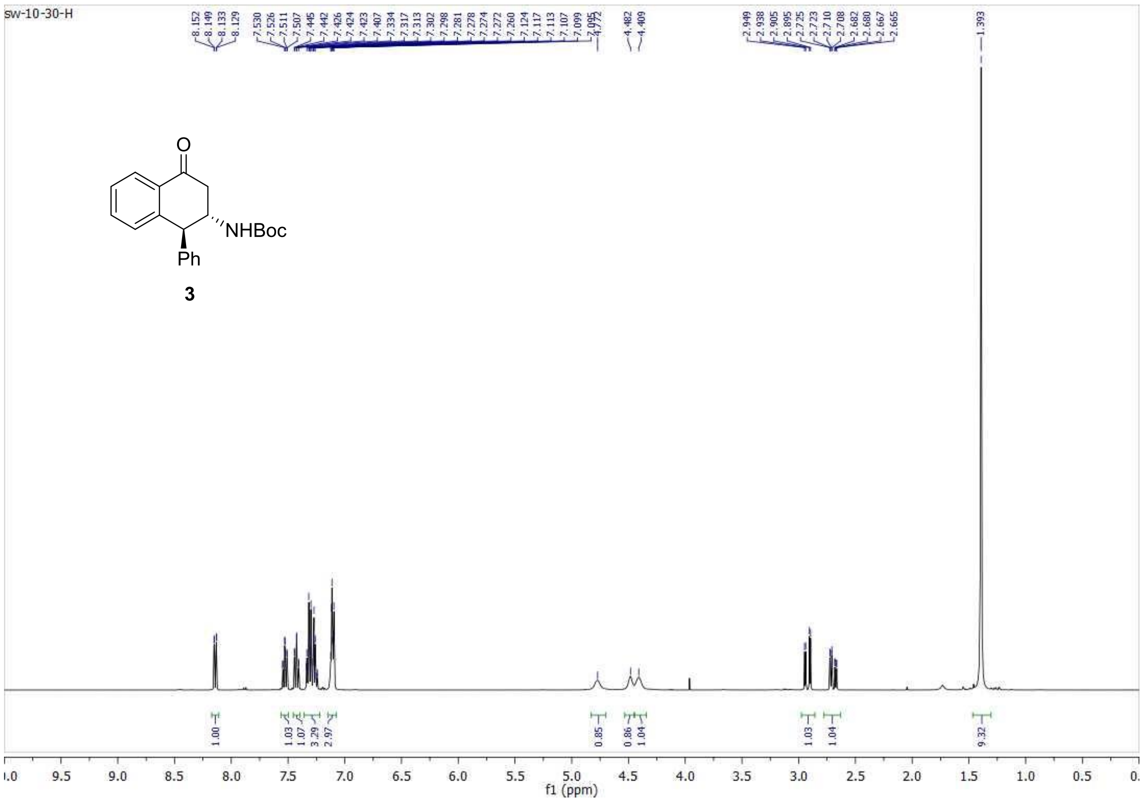

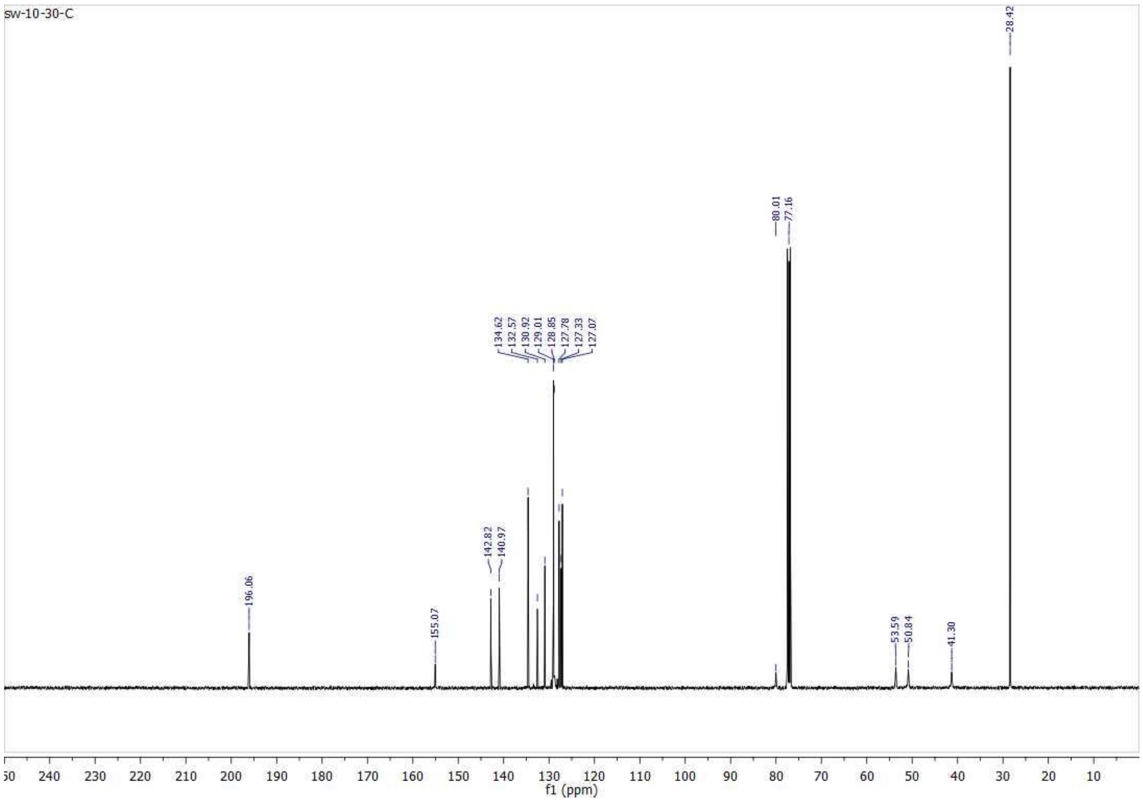

Supplementary Figure 38. ^{1}H and ^{13}C NMR Spectra of **3**.

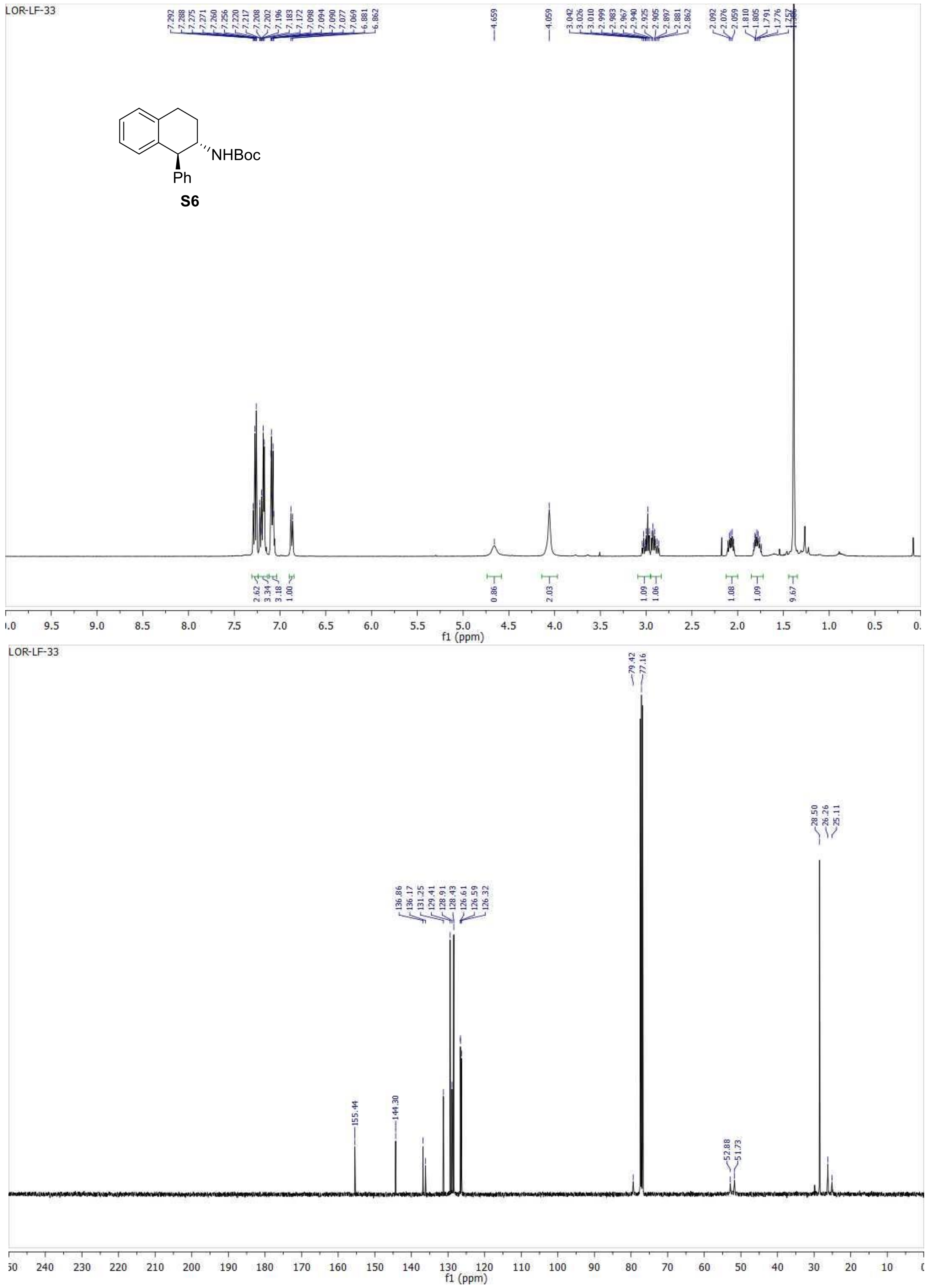

Supplementary Figure 39. ^{1}H and ^{13}C NMR Spectra of **S6**.

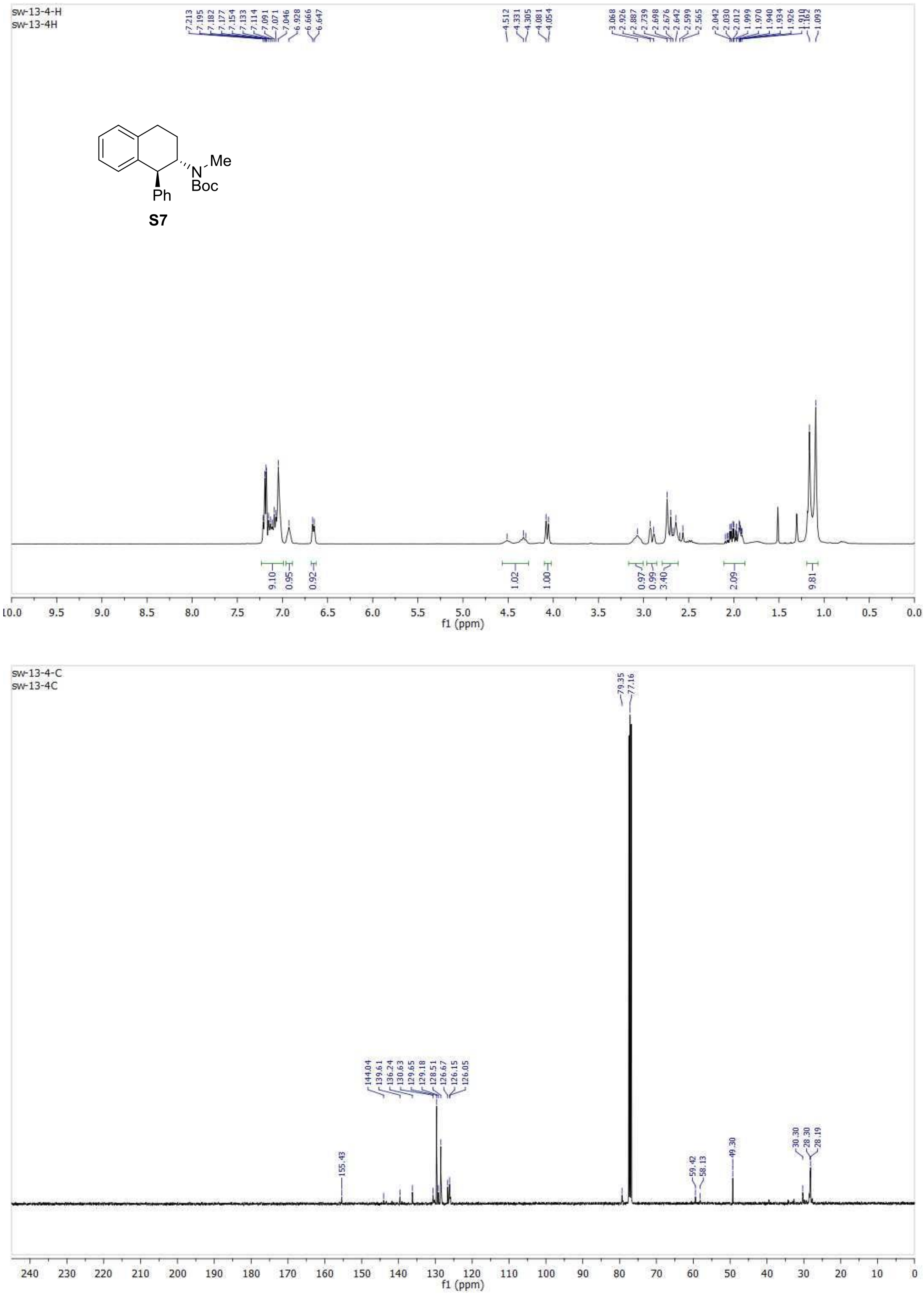

Supplementary Figure 40. ^{1}H and ^{13}C NMR Spectra of **S7**.

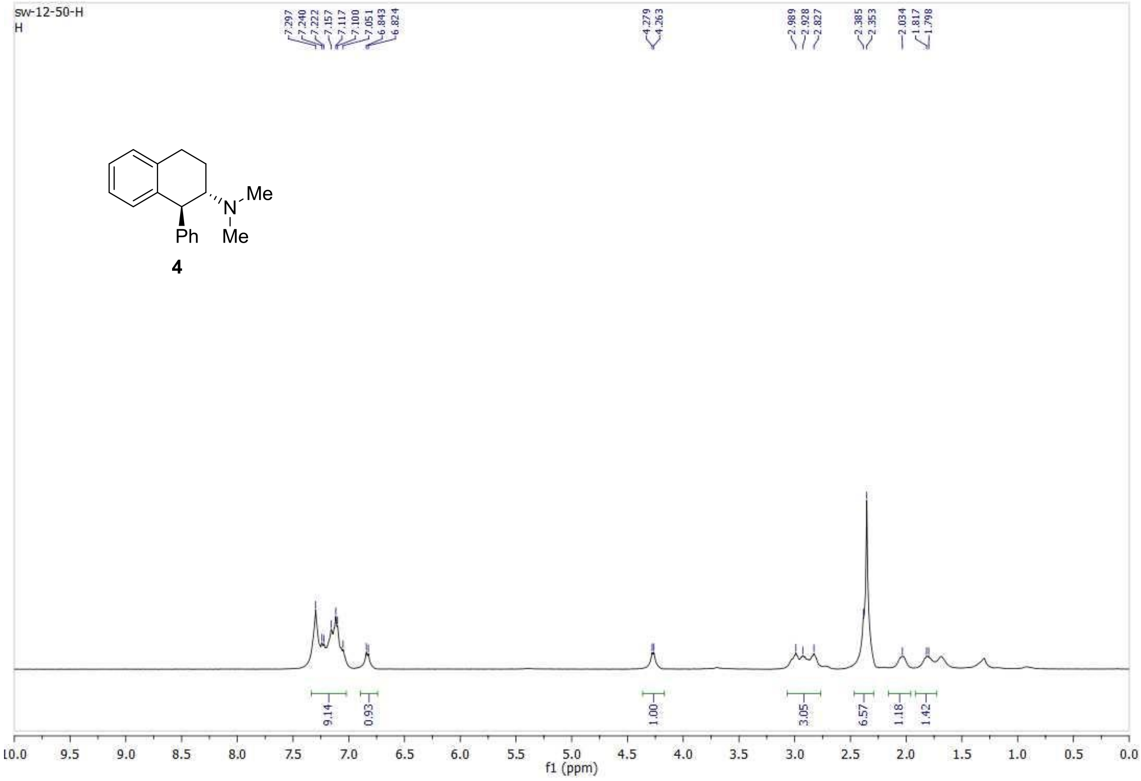

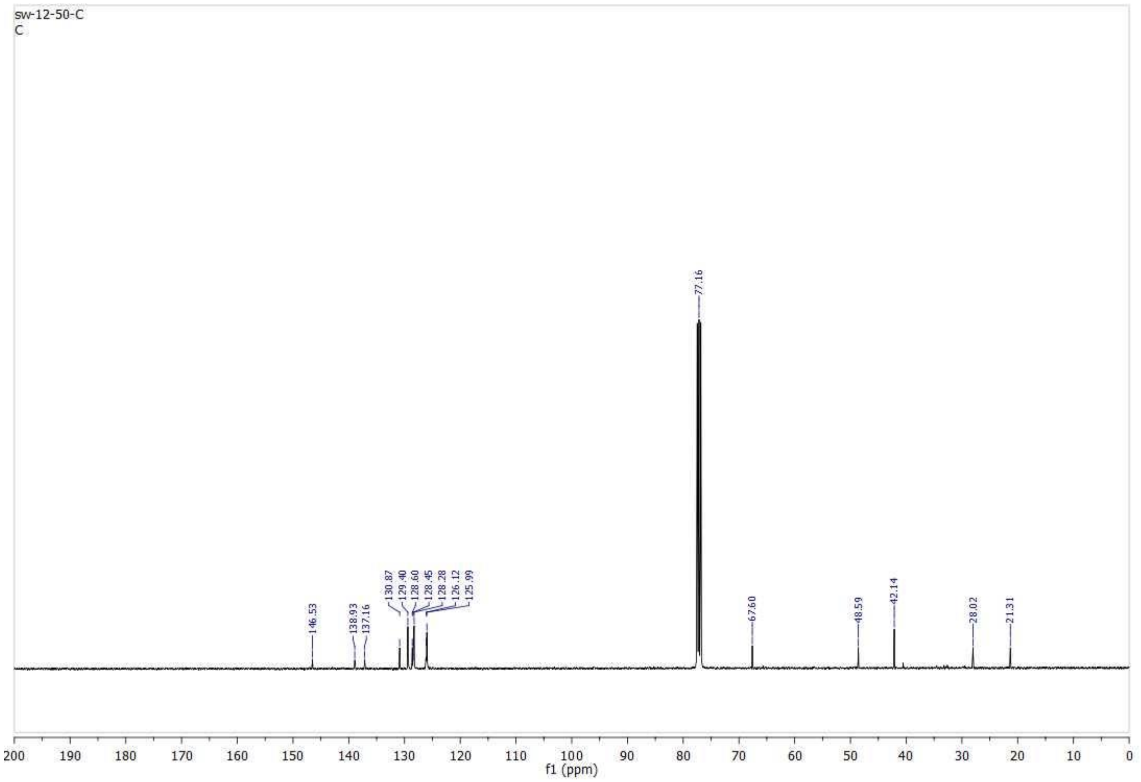

Supplementary Figure 41. ^{1}H and ^{13}C NMR Spectra of **4**.

.

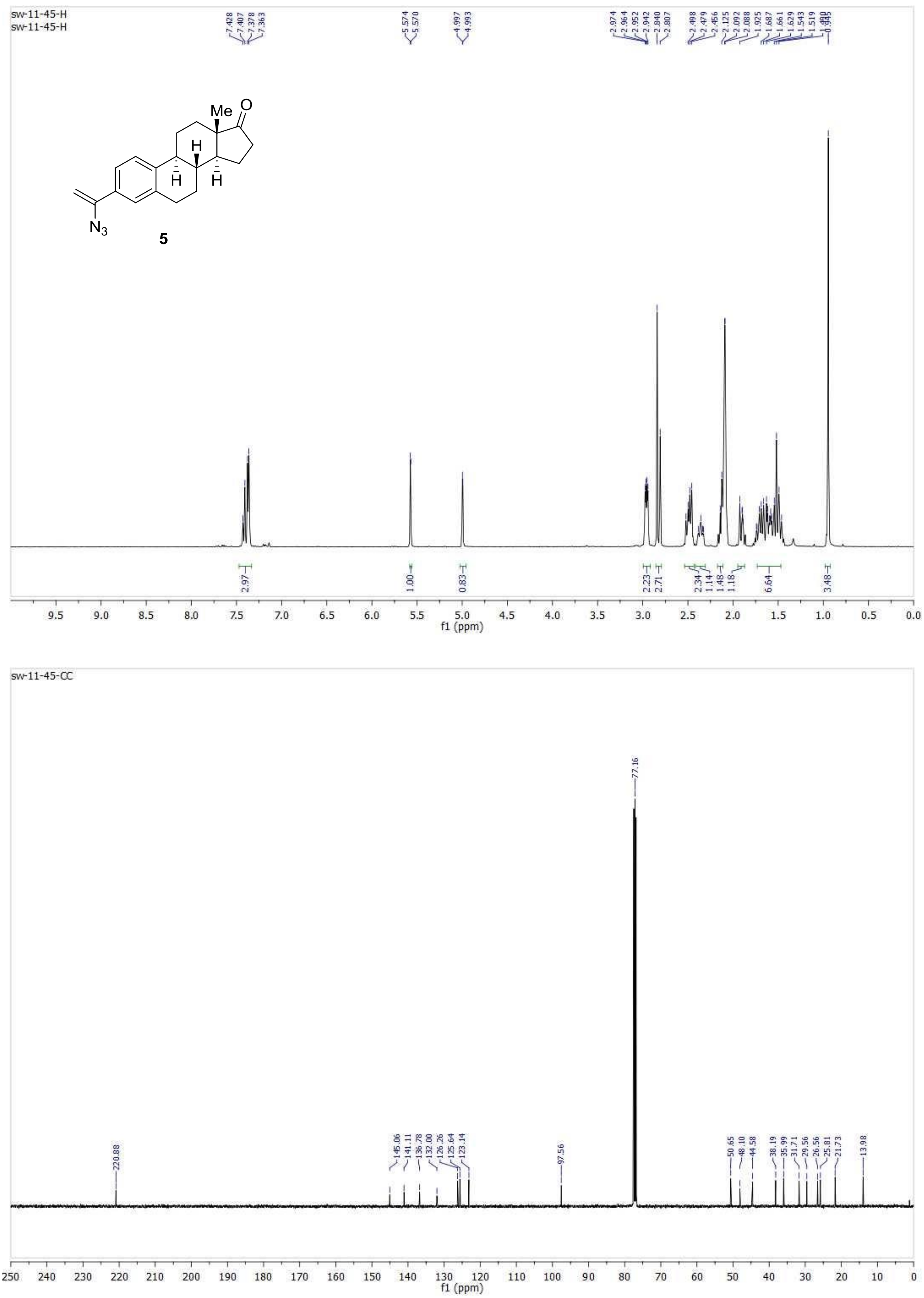

Supplementary Figure 42. ^{1}H and ^{13}C NMR Spectra of **5**.

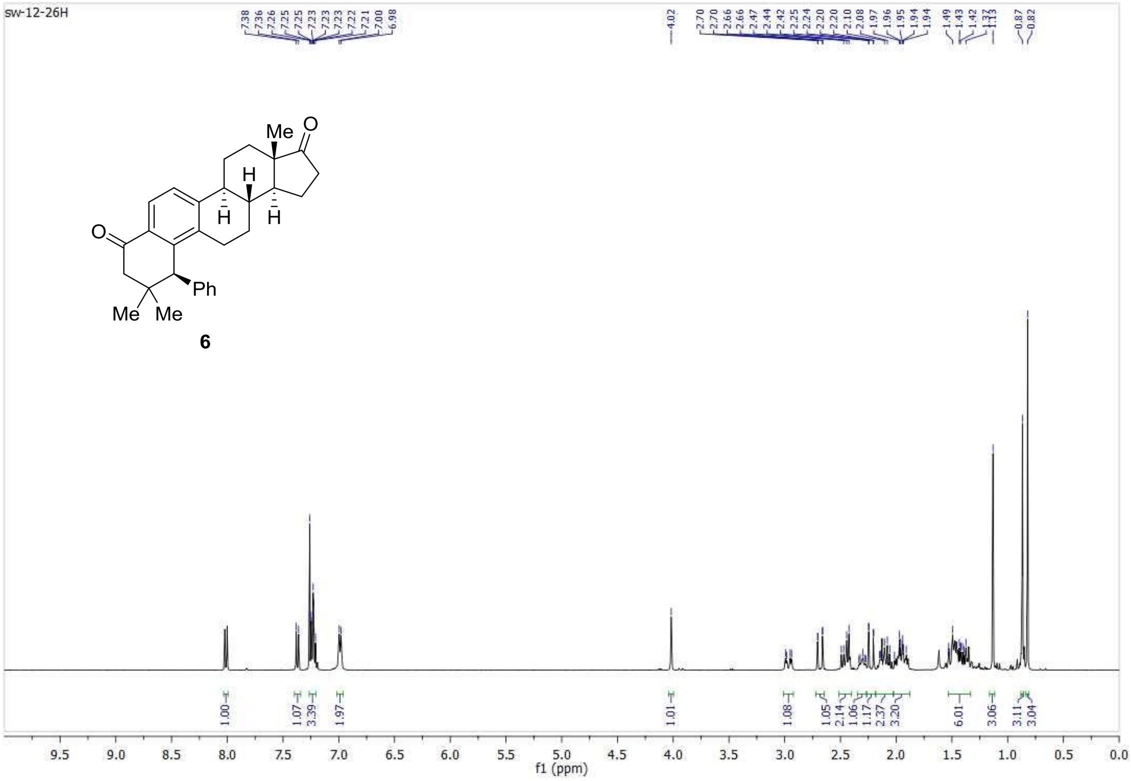

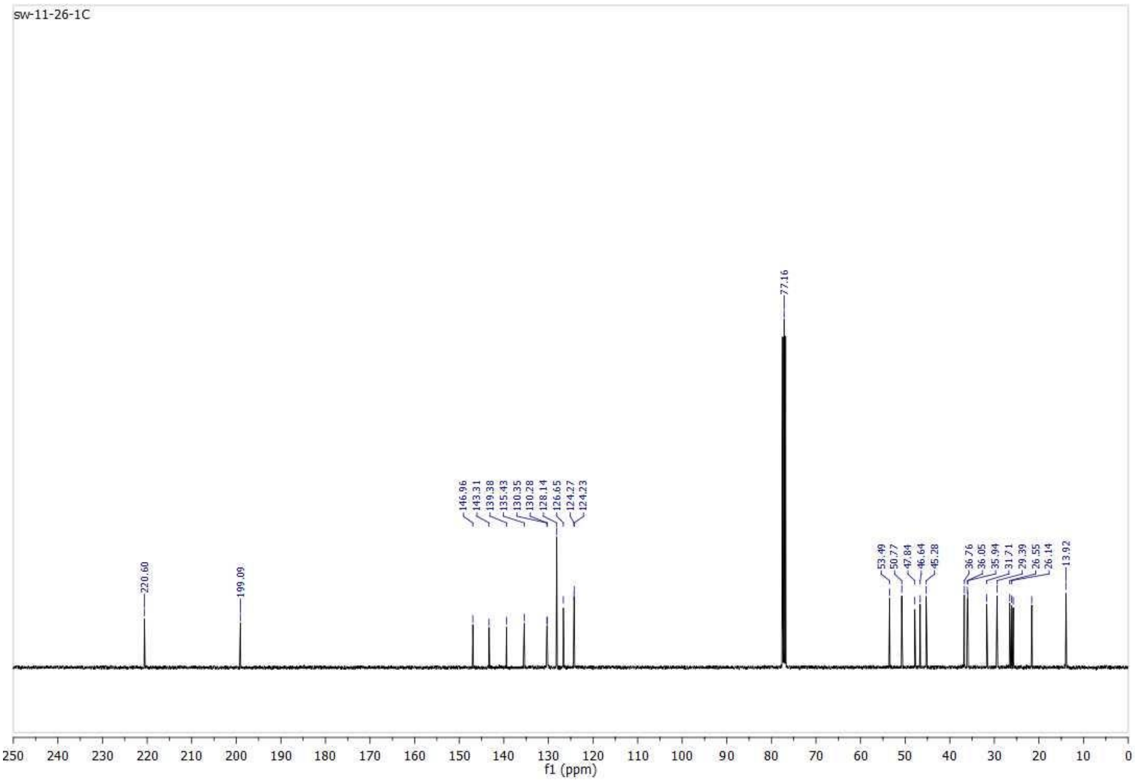

Supplementary Figure 43. ^{1}H and ^{13}C NMR Spectra of **6**.

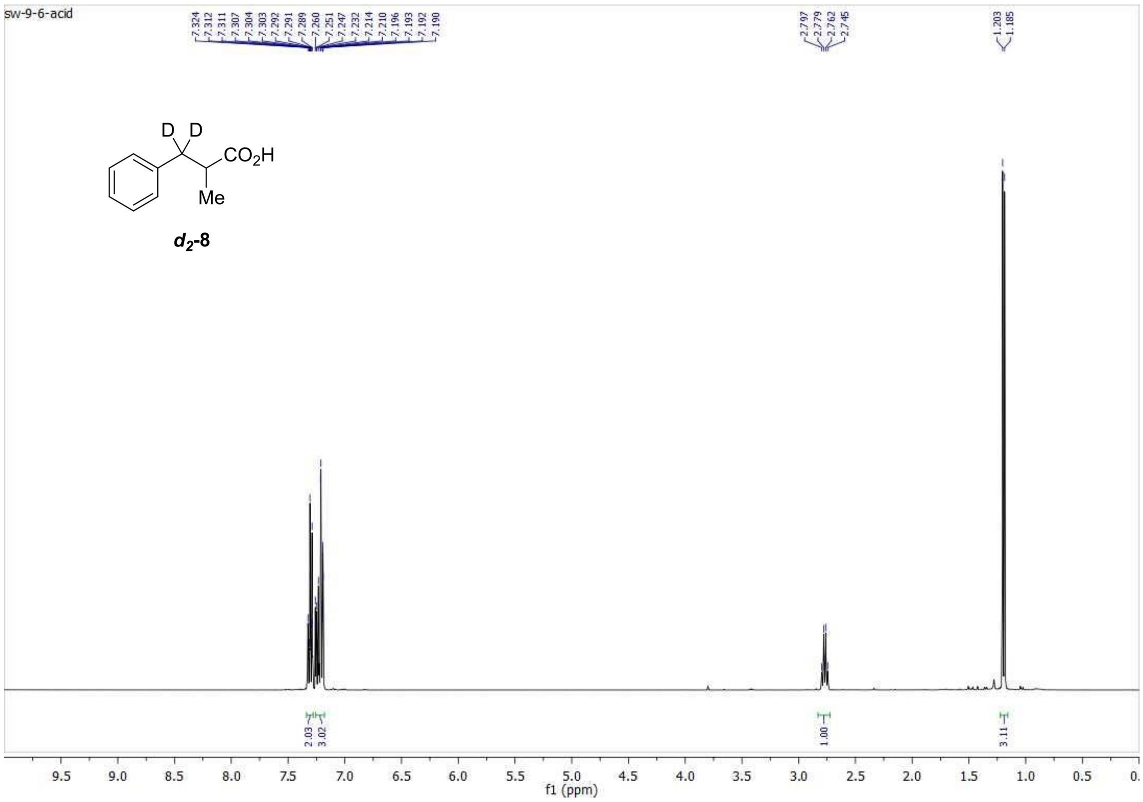

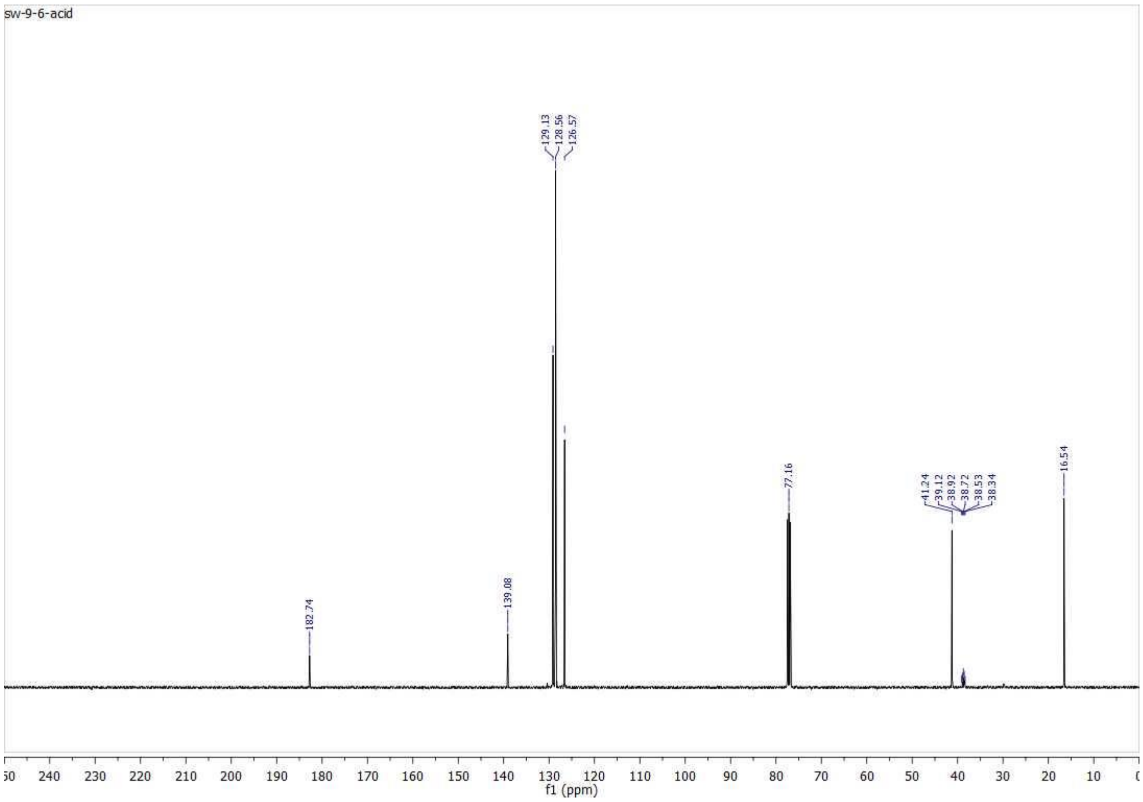

Supplementary Figure 44. ^{1}H and ^{13}C NMR Spectra of ***d₂*-8**.

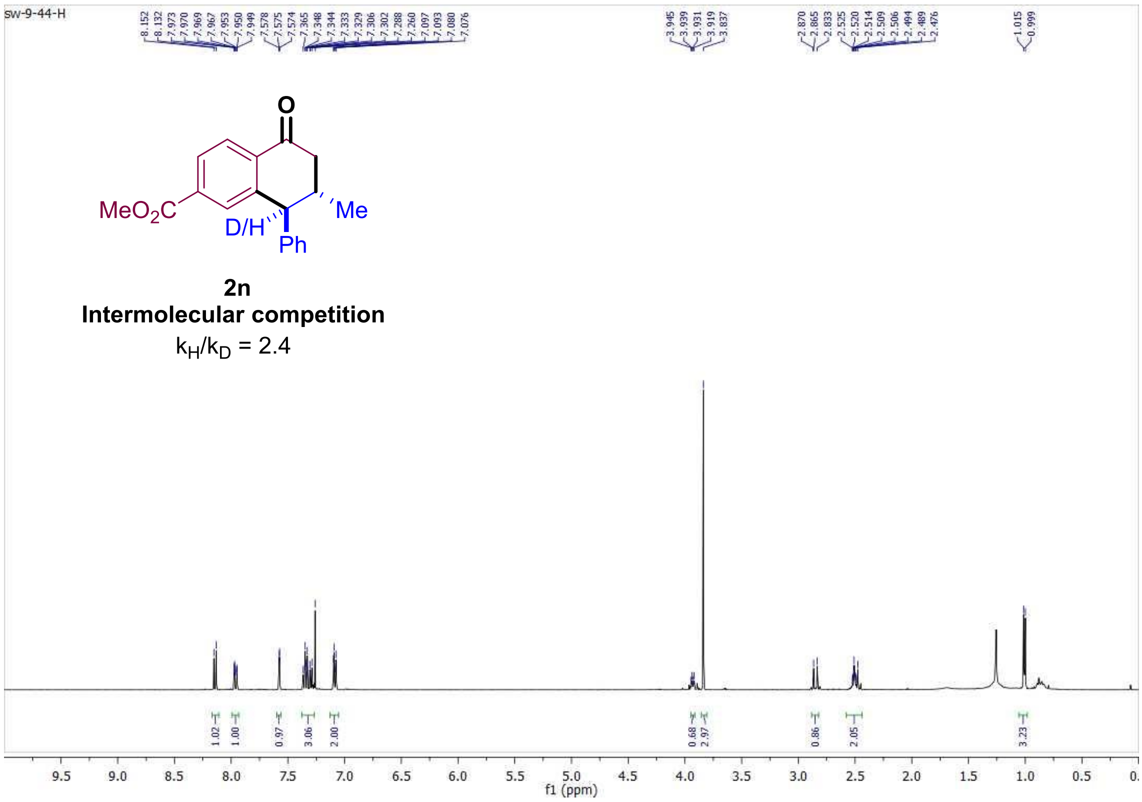
sw-9-44-H
MeO2C
D/H
Ph
Me
O
2n
Intermolecular competition
kH/kD = 2.4
f1 (ppm)

HR-ESI-MS (Bruker maXis)

Analysis Info

Analysis Name	D:\Data\Service\8358nehres.d	Acquisition Date	11/18/2015 11:06:00 AM
Method	tune_low_modified_09_01_14_pos_TuneMix.m	Operator	ust
Sample Name	sw-9-44	Instrument	maXis 255552.00033
Comment	Solvent: MeCN + NaI<br>Client: Shu		

Acquisition Parameter

Source Type	ESI	Ion Polarity	Positive	Set Nebulizer	0.5 Bar
Scan Begin	50 m/z	Set Capillary	4000 V	Set Dry Heater	180 °C
Scan End	3000 m/z	Set End Plate Offset	-500 V	Set Dry Gas	4.0 l/min

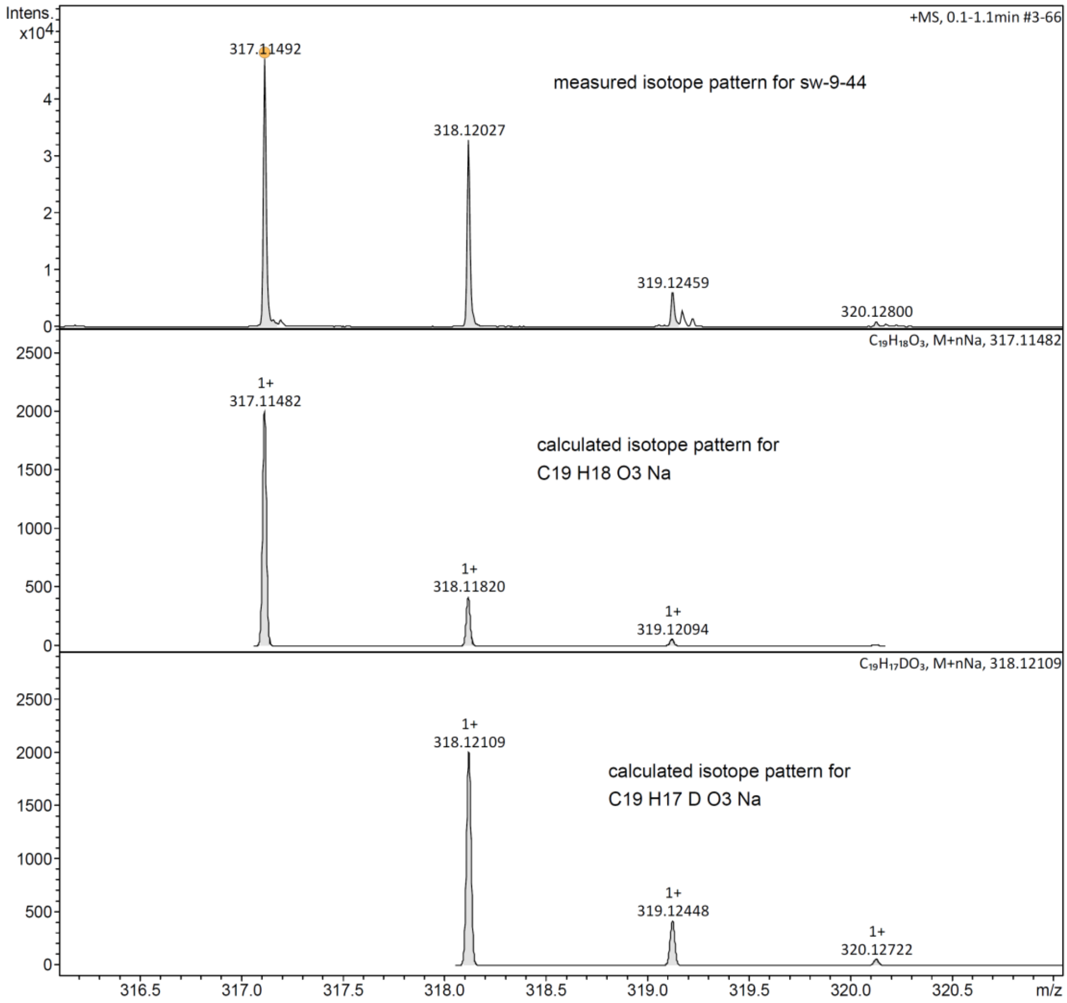

Bruker Compass DataAnalysis 4.2 printed: 11/30/2015 8:17:42 AM by: ust Page 1 of 2

HR-ESI-MS (Bruker maXis)

#	m/z	Res.	S/N	I	I %	FWHM
1	317.11492	20578	5998.7	47066	100.0	0.01541
2	318.12027	20369	4175.3	32760	69.6	0.01562
3	319.12459	17568	765.8	6010	12.8	0.01817
4	320.12800	17077	113.3	889	1.9	0.01875

measured isotope pattern for sw-9-44

#	m/z	Res.	S/N	I	I %	FWHM
1	317.11482	15246		2000	100.0	0.02080
2	318.11820	15294		417	20.9	0.02080
3	319.12094	15342		54	2.7	0.02080
4	320.12363	15391		5	0.3	0.02080

calculated isotope pattern for
C19 H18 O3 Na

#	m/z	Res.	S/N	I	I %	FWHM
1	318.12109	12624		2000	100.0	0.02520
2	319.12448	12664		417	20.9	0.02520
3	320.12722	12703		54	2.7	0.02520
4	321.12990	12743		5	0.3	0.02520

calculated isotope pattern for
C19 H17 D O3 Na

Calculated isotope pattern for
C19 H18 O3 Na : 2.4
C19 H17 D O3 Na : 1.0
317.1 100%
318.1 62.5%
319.1 11.4%

Calculated isotope pattern for
C19 H18 O3 Na : 2.05
C19 H17 D O3 Na : 1.00
317.1 100%
318.1 69.7%
319.1 12.9%

Bruker Compass DataAnalysis 4.2 printed: 11/30/2015 8:17:42 AM by: ust Page 2 of 2

Supplementary Figure 45. ^{1}H NMR and Mass Spectra of Intermolecular Competition Experiment.

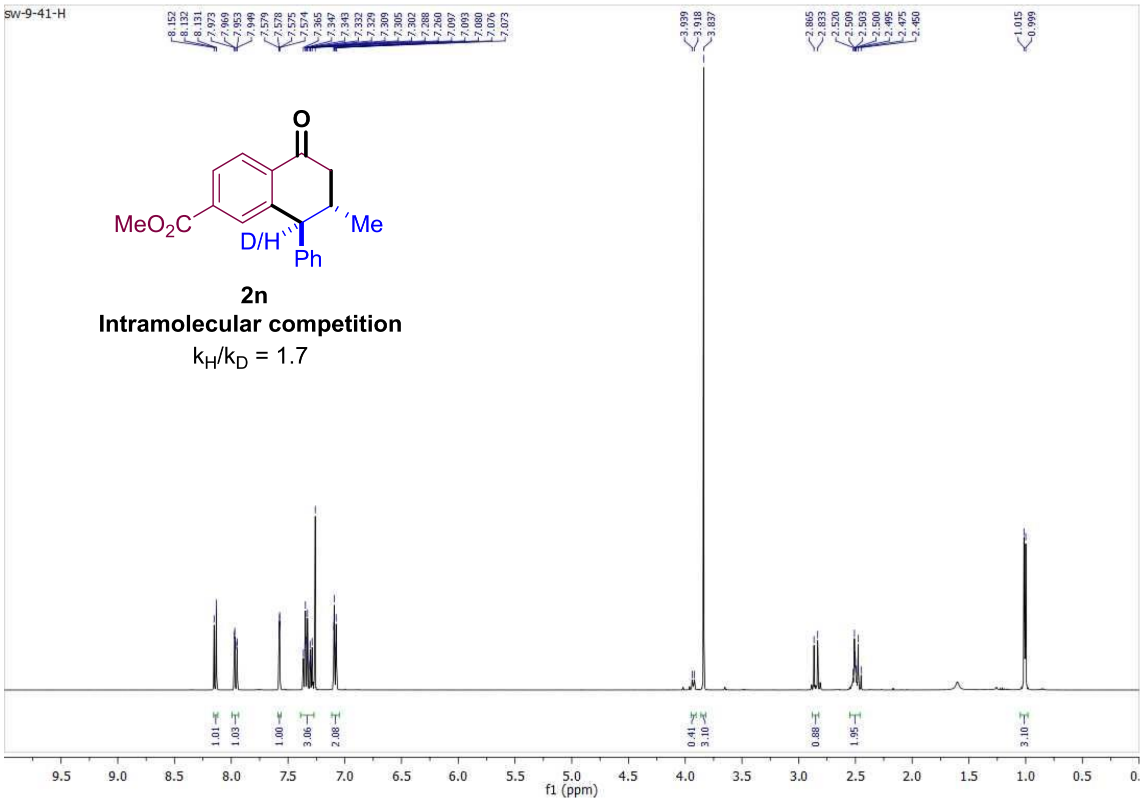

sw-9-41-H
MeO2C
D/H
Me
Ph
O
2n
Intramolecular competition
kH/kD = 1.7
f1 (ppm)

HR-ESI-MS (Bruker maXis)

Analysis Info

Analysis Name	D:\Data\Service\8357nehres.d	Acquisition Date	11/18/2015 10:30:41 AM
Method	tune_low_modified_09_01_14_pos_TuneMix.m	Operator	ust
Sample Name	sw-9-41	Instrument	maXis 255552.00033
Comment	Solvent: MeCN + NaI<br>Client: Shu		

Acquisition Parameter

Source Type	ESI	Ion Polarity	Positive	Set Nebulizer	0.5 Bar
Scan Begin	50 m/z	Set Capillary	4000 V	Set Dry Heater	180 °C
Scan End	3000 m/z	Set End Plate Offset	-500 V	Set Dry Gas	4.0 l/min

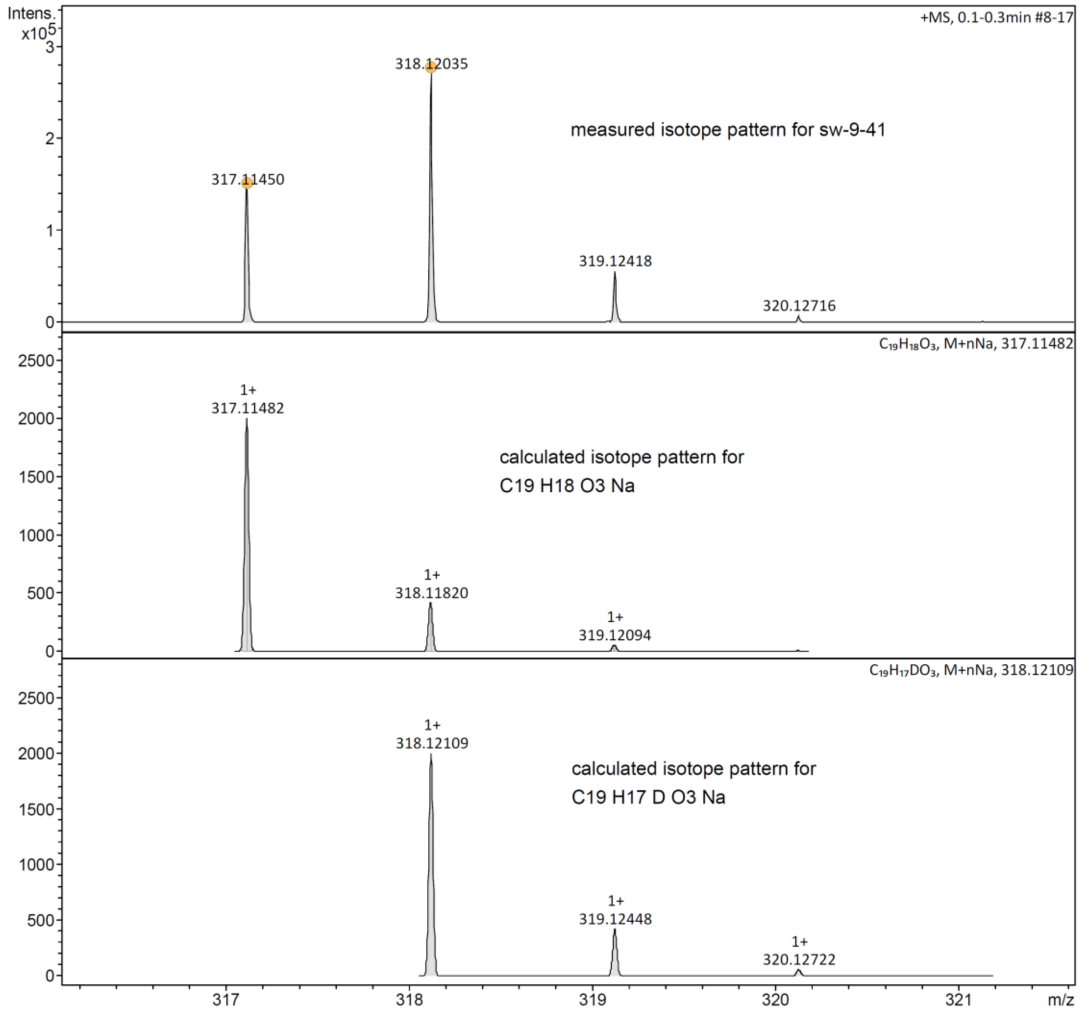

Bruker Compass DataAnalysis 4.2 printed: 11/30/2015 8:51:07 AM by: ust Page 1 of 2

HR-ESI-MS (Bruker maXis)

#	m/z	Res.	S/N	I	I %	FWHM
1	317.11450	19605	8969.2	144650	53.6	0.01617
2	318.12035	19682	16723.3	270032	100.0	0.01616
3	319.12418	20246	3432.7	55487	20.5	0.01576
4	320.12716	19388	448.5	7268	2.7	0.01651

measured isotope pattern for sw-9-41

#	m/z	Res.	S/N	I	I %	FWHM
1	317.11482	12584		2000	100.0	0.02520
2	318.11820	12624		417	20.9	0.02520
3	319.12094	12664		54	2.7	0.02520
4	320.12363	12703		5	0.3	0.02520

calculated isotope pattern for
C19 H18 O3 Na

#	m/z	Res.	S/N	I	I %	FWHM
1	318.12109	12624		2000	100.0	0.02520
2	319.12448	12664		417	20.9	0.02520
3	320.12722	12703		54	2.7	0.02520
4	321.12990	12743		5	0.3	0.02520

calculated isotope pattern for
C19 H17 D O3 Na

Calculated isotope pattern for
C19 H18 O3 Na : 1.0
C19 H17 D O3 Na : 1.7
317.1 53.8 %
318.1 100.0 %
319.1 20.0 %

Bruker Compass DataAnalysis 4.2 printed: 11/30/2015 8:51:07 AM by: ust Page 2 of 2

Supplementary Figure 46. ^{1}H NMR and Mass Spectra of Intramolecular Competition Experiment.

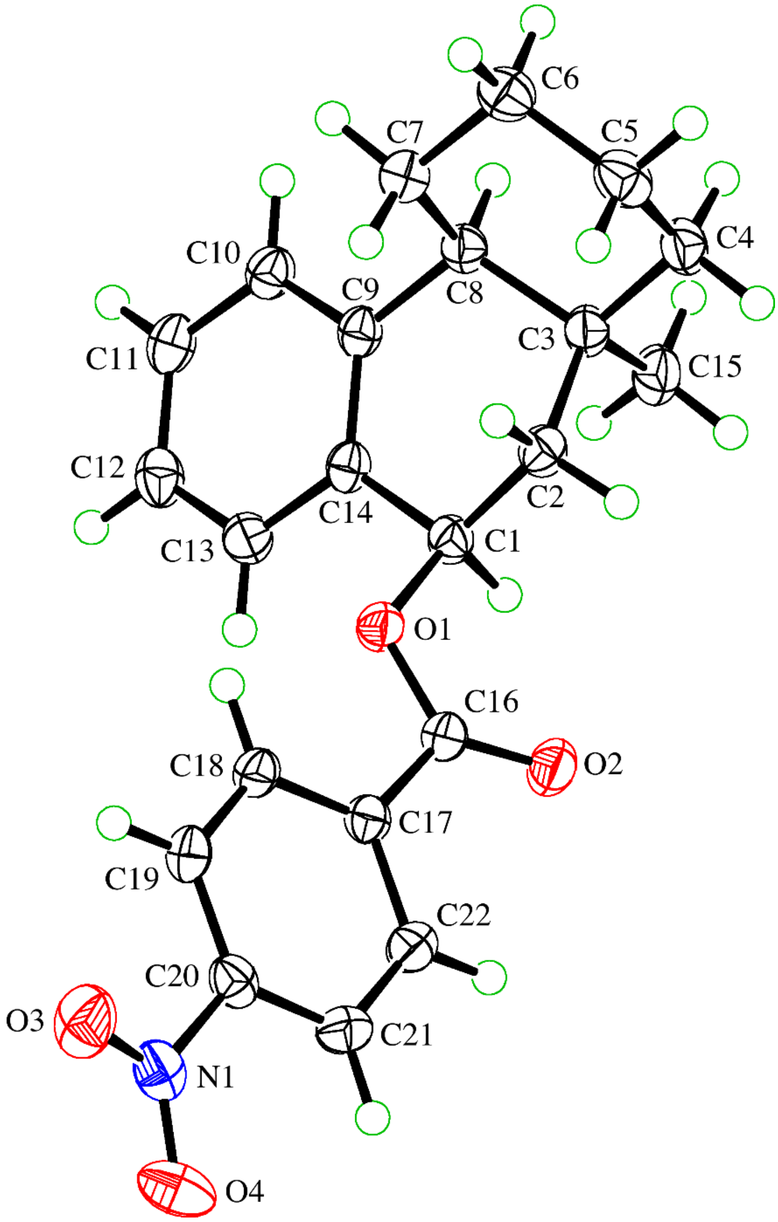

Supplementary Figure 47. X-ray diffraction structure of compound **1a`** (CCDC-1504120).

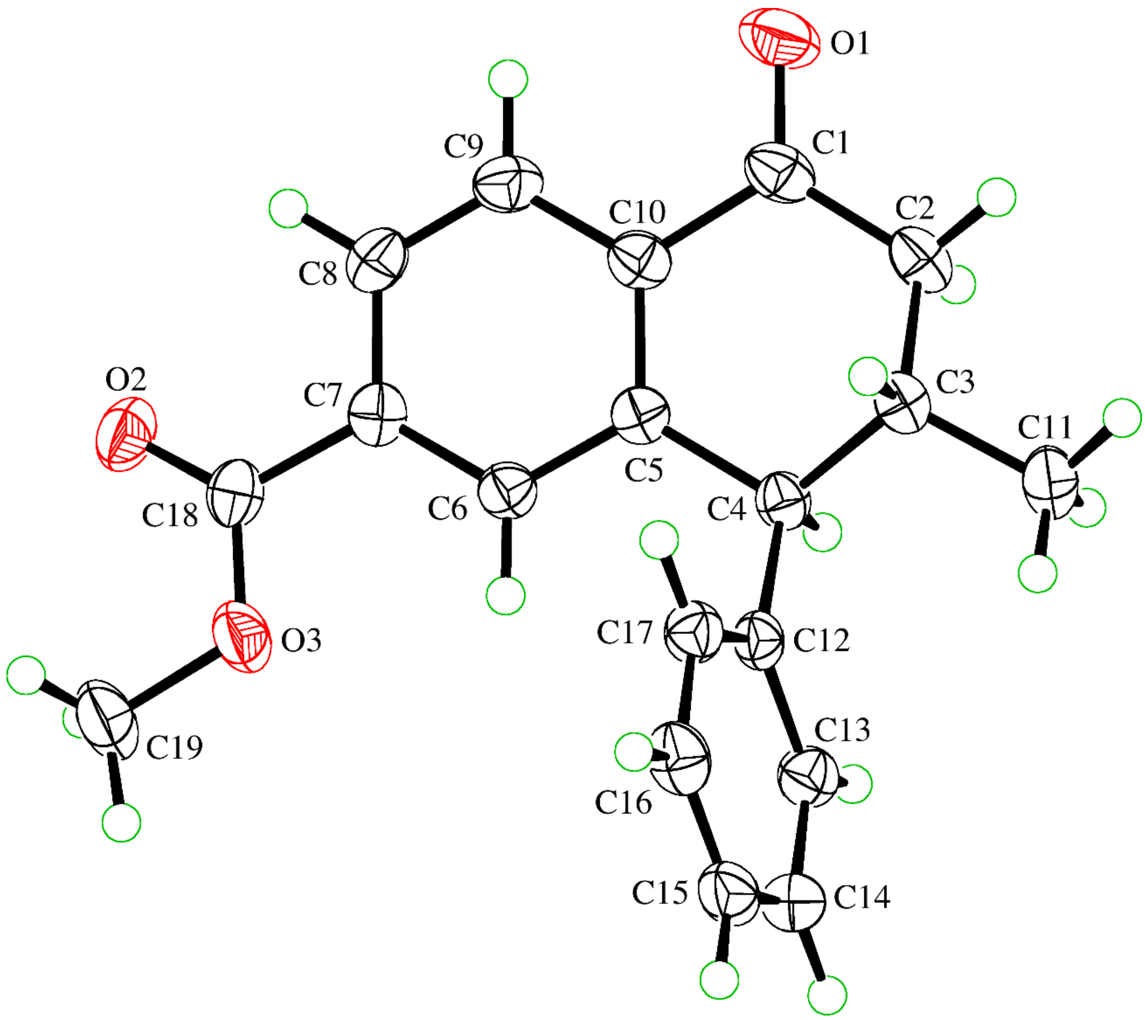

Supplementary Figure 48. X-ray diffraction structure of compound **2n** (CCDC-1445931).

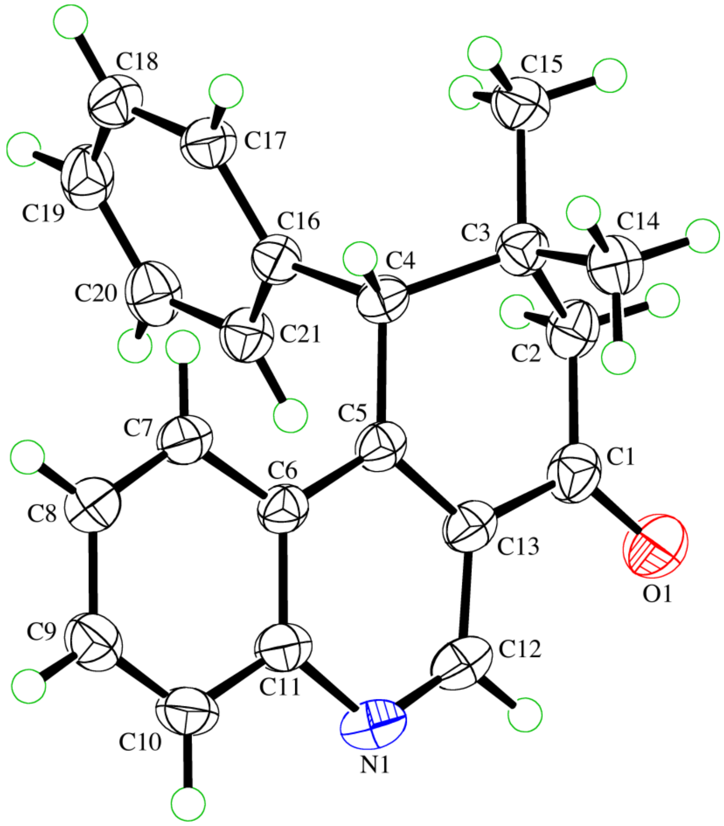

Supplementary Figure 49. X-ray diffraction structure of compound **2s** (CCDC- 1481107).

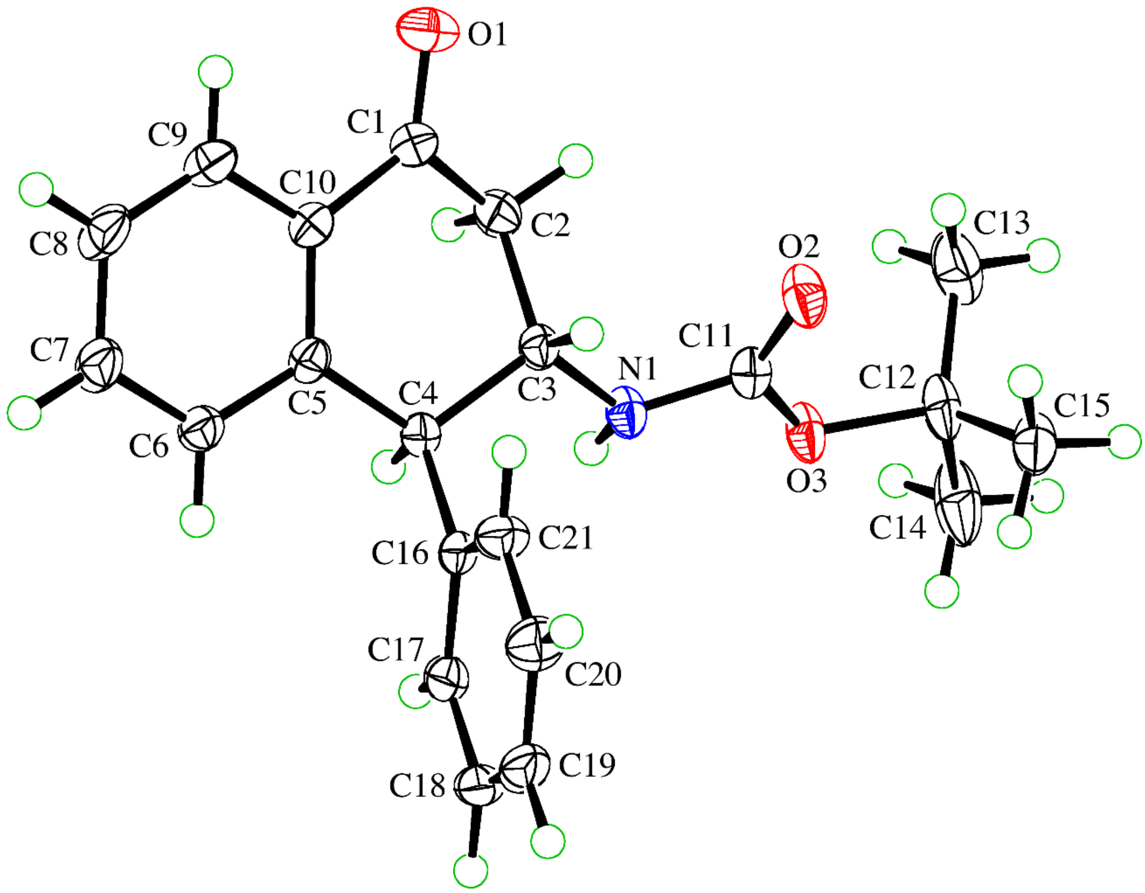

Supplementary Figure 50. X-ray diffraction structure of compound **3** (CCDC-1445926).

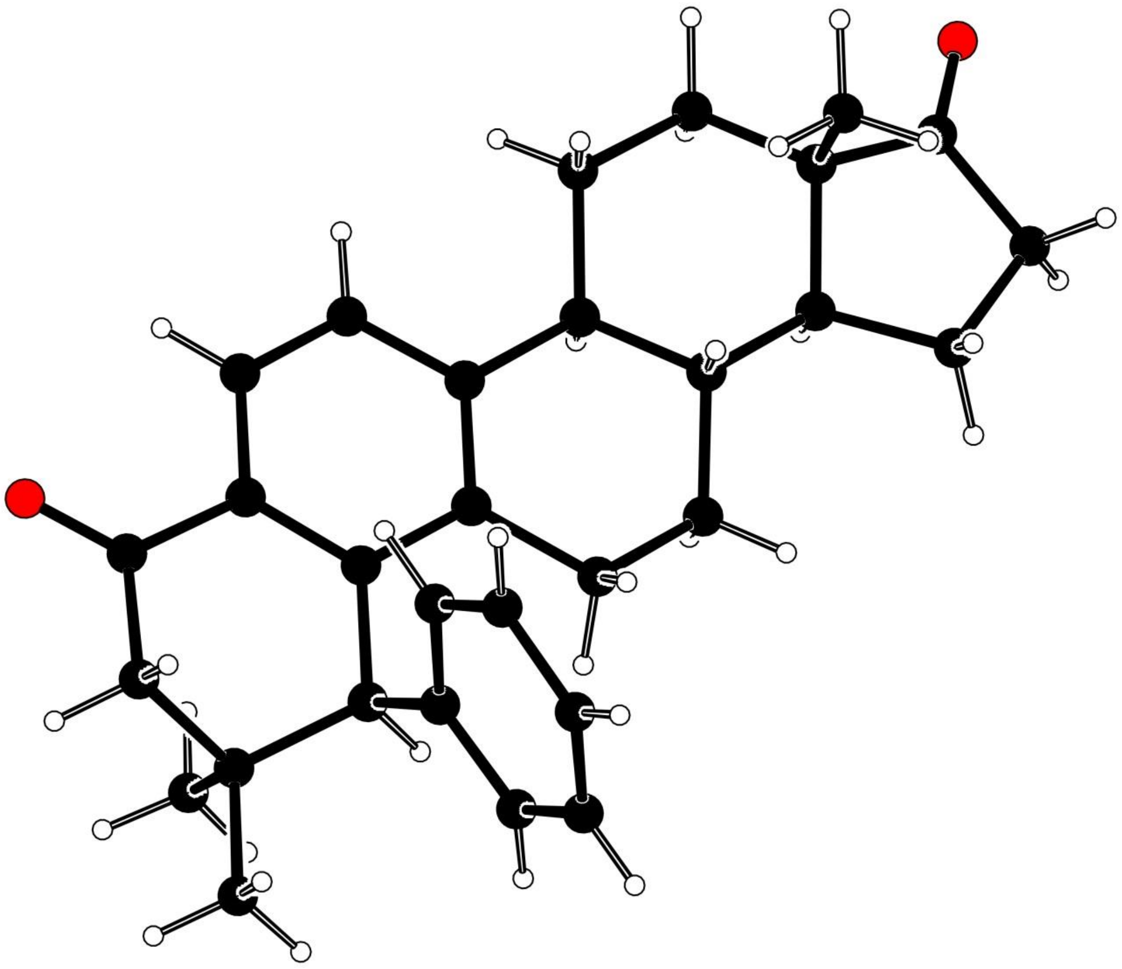

Supplementary Figure 51. X-ray diffraction structure of compound **6** (CCDC-1445932).

Supplementary Tables

Supplementary Table 1. Crystallographic data of **1a**`.

Crystallised from	diethyl ether / EtOAc / hexanes
Empirical formula	$C_{22}H_{23}NO_4$
Formula weight [g mol^{-1}]	365.41
Crystal colour, habit	colourless, prism
Crystal dimensions [mm]	0.10 × 0.16 × 0.19
Temperature [K]	160(1)
Crystal system	monoclinic
Space group	$P2_1/c$ (#14)
Z	4

Reflections for cell determination		8888
2θ range for cell determination [°]		6–149
Unit cell parameters	a [Å]	13.04096(16)
	b [Å]	11.42545(11)
	c [Å]	12.30399(18)
	α [°]	90
	β [°]	93.4225(12)
	γ [°]	90
	V [Å^3]	1830.01(4)
$F(000)$		776
D_X [g cm^{-3}]		1.326
μ(Cu $K\alpha$) [mm^{-1}]		0.739
Scan type		ω
$2\theta_{(max)}$ [°]		148.5
Transmission factors (min; max)		0.142; 1.000
Total reflections measured		17742
Symmetry independent reflections		3684
R_{int}		0.022
Reflections with $I > 2\sigma(I)$		3372
Reflections used in refinement		3684
Parameters refined		246
Final	$R(F)$ [$I > 2\sigma(I)$ reflections]	0.0340
	$wR(F^2)$ (all data)	0.0905
Weights:		$w = [\sigma^2(F_o^2) + (0.0421P)^2 + 0.5407P]^{-1}$ where $P = (F_o^2 + 2F_c^2)/3$
Goodness of fit		1.054
Secondary extinction coefficient		0.0010(2)

Final Δ_{max}/σ	0.001
$\Delta\rho$ (max; min) [e Å^{-3}]	0.26; -0.19
$\sigma(d_{(C-C)})$ [Å]	0.0014 – 0.002

Supplementary Table 2. Bond lengths (Å) of **1a`** with standard uncertainties in parentheses.

O(1)	-C(16)	1.3425(13)	C(7)	-C(8)	1.5383(15)
O(1)	-C(1)	1.4702(12)	C(8)	-C(9)	1.5144(15)
O(2)	-C(16)	1.2041(14)	C(9)	-C(10)	1.3976(15)
O(3)	-N(1)	1.2256(15)	C(9)	-C(14)	1.4009(15)
O(4)	-N(1)	1.2257(15)	C(10)	-C(11)	1.3839(16)
N(1)	-C(20)	1.4706(14)	C(11)	-C(12)	1.3882(17)
C(1)	-C(14)	1.5137(14)	C(12)	-C(13)	1.3844(16)
C(1)	-C(2)	1.5199(15)	C(13)	-C(14)	1.3963(15)
C(2)	-C(3)	1.5375(15)	C(16)	-C(17)	1.4942(15)
C(3)	-C(15)	1.5371(16)	C(17)	-C(18)	1.3906(15)
C(3)	-C(4)	1.5385(15)	C(17)	-C(22)	1.3953(15)
C(3)	-C(8)	1.5450(14)	C(18)	-C(19)	1.3880(16)
C(4)	-C(5)	1.5219(18)	C(19)	-C(20)	1.3836(16)
C(5)	-C(6)	1.5242(17)	C(20)	-C(21)	1.3814(17)
C(6)	-C(7)	1.5289(16)	C(21)	-C(22)	1.3808(17)

Supplementary Table 3. Bond angles (°) of **1a`** with standard uncertainties in parentheses.

C(16)	-O(1)	-C(1)	115.62(8)	C(10)	-C(9)	-C(8)	119.39(10)
O(3)	-N(1)	-O(4)	124.18(11)	C(14)	-C(9)	-C(8)	121.99(9)
O(3)	-N(1)	-C(20)	117.95(11)	C(11)	-C(10)	-C(9)	121.49(10)
O(4)	-N(1)	-C(20)	117.87(11)	C(10)	-C(11)	-C(12)	119.62(10)
O(1)	-C(1)	-C(14)	107.51(8)	C(13)	-C(12)	-C(11)	119.74(10)
O(1)	-C(1)	-C(2)	109.14(9)	C(12)	-C(13)	-C(14)	120.97(11)
C(14)	-C(1)	-C(2)	114.43(9)	C(13)	-C(14)	-C(9)	119.57(10)
C(1)	-C(2)	-C(3)	111.94(9)	C(13)	-C(14)	-C(1)	119.09(10)
C(15)	-C(3)	-C(2)	110.43(10)	C(9)	-C(14)	-C(1)	121.08(9)
C(15)	-C(3)	-C(4)	108.20(9)	O(2)	-C(16)	-O(1)	124.42(10)
C(2)	-C(3)	-C(4)	110.48(9)	O(2)	-C(16)	-C(17)	123.04(10)
C(15)	-C(3)	-C(8)	109.20(9)	O(1)	-C(16)	-C(17)	112.54(9)
C(2)	-C(3)	-C(8)	108.76(9)	C(18)	-C(17)	-C(22)	120.29(10)
C(4)	-C(3)	-C(8)	109.75(9)	C(18)	-C(17)	-C(16)	122.58(10)
C(5)	-C(4)	-C(3)	114.23(9)	C(22)	-C(17)	-C(16)	117.13(10)
C(4)	-C(5)	-C(6)	110.59(10)	C(19)	-C(18)	-C(17)	120.09(10)
C(5)	-C(6)	-C(7)	110.64(10)	C(20)	-C(19)	-C(18)	118.00(10)
C(6)	-C(7)	-C(8)	112.33(9)	C(21)	-C(20)	-C(19)	123.22(10)
C(9)	-C(8)	-C(7)	109.79(9)	C(21)	-C(20)	-N(1)	118.14(10)
C(9)	-C(8)	-C(3)	111.40(9)	C(19)	-C(20)	-N(1)	118.63(11)
C(7)	-C(8)	-C(3)	113.03(9)	C(22)	-C(21)	-C(20)	118.09(10)
C(10)	-C(9)	-C(14)	118.60(10)	C(21)	-C(22)	-C(17)	120.28(11)

Supplementary Table 4. Torsion angles (°) of **1a`** with standard uncertainties in parentheses.

C(16)	-O(1)	-C(1)	-C(14)	151.08(9)	C(12)	-C(13)	-C(14)	-C(9)	0.5(2)
C(16)	-O(1)	-C(1)	-C(2)	-84.3(1)	C(12)	-C(13)	-C(14)	-C(1)	-173.7(1)

O(1)	-C(1)	-C(2)	-C(3)	-162.36(8)	C(10)	-C(9)	-C(14)	-C(13)	-0.6(2)
C(14)	-C(1)	-C(2)	-C(3)	-41.9(1)	C(8)	-C(9)	-C(14)	-C(13)	178.1(1)
C(1)	-C(2)	-C(3)	-C(15)	-58.2(1)	C(10)	-C(9)	-C(14)	-C(1)	173.5(1)
C(1)	-C(2)	-C(3)	-C(4)	-177.84(9)	C(8)	-C(9)	-C(14)	-C(1)	-7.9(2)
C(1)	-C(2)	-C(3)	-C(8)	61.6(1)	O(1)	-C(1)	-C(14)	-C(13)	-49.8(1)
C(15)	-C(3)	-C(4)	-C(5)	171.3(1)	C(2)	-C(1)	-C(14)	-C(13)	-171.2(1)
C(2)	-C(3)	-C(4)	-C(5)	-67.7(1)	O(1)	-C(1)	-C(14)	-C(9)	136.1(1)
C(8)	-C(3)	-C(4)	-C(5)	52.3(1)	C(2)	-C(1)	-C(14)	-C(9)	14.7(1)
C(3)	-C(4)	-C(5)	-C(6)	-56.3(1)	C(1)	-O(1)	-C(16)	-O(2)	-1.4(2)
C(4)	-C(5)	-C(6)	-C(7)	56.3(1)	C(1)	-O(1)	-C(16)	-C(17)	179.32(8)
C(5)	-C(6)	-C(7)	-C(8)	-55.4(1)	O(2)	-C(16)	-C(17)	-C(18)	167.3(1)
C(6)	-C(7)	-C(8)	-C(9)	178.02(9)	O(1)	-C(16)	-C(17)	-C(18)	-13.4(2)
C(6)	-C(7)	-C(8)	-C(3)	53.0(1)	O(2)	-C(16)	-C(17)	-C(22)	-12.2(2)
C(15)	-C(3)	-C(8)	-C(9)	67.6(1)	O(1)	-C(16)	-C(17)	-C(22)	167.1(1)
C(2)	-C(3)	-C(8)	-C(9)	-53.0(1)	C(22)	-C(17)	-C(18)	-C(19)	-0.5(2)
C(4)	-C(3)	-C(8)	-C(9)	-173.93(9)	C(16)	-C(17)	-C(18)	-C(19)	180.0(1)
C(15)	-C(3)	-C(8)	-C(7)	-168.22(9)	C(17)	-C(18)	-C(19)	-C(20)	-1.2(2)
C(2)	-C(3)	-C(8)	-C(7)	71.2(1)	C(18)	-C(19)	-C(20)	-C(21)	1.7(2)
C(4)	-C(3)	-C(8)	-C(7)	-49.8(1)	C(18)	-C(19)	-C(20)	-N(1)	-177.2(1)
C(7)	-C(8)	-C(9)	-C(10)	80.3(1)	O(3)	-N(1)	-C(20)	-C(21)	160.8(1)
C(3)	-C(8)	-C(9)	-C(10)	-153.7(1)	O(4)	-N(1)	-C(20)	-C(21)	-18.5(2)
C(7)	-C(8)	-C(9)	-C(14)	-98.4(1)	O(3)	-N(1)	-C(20)	-C(19)	-20.3(2)
C(3)	-C(8)	-C(9)	-C(14)	27.6(1)	O(4)	-N(1)	-C(20)	-C(19)	160.5(1)
C(14)	-C(9)	-C(10)	-C(11)	0.0(2)	C(19)	-C(20)	-C(21)	-C(22)	-0.5(2)
C(8)	-C(9)	-C(10)	-C(11)	-178.8(1)	N(1)	-C(20)	-C(21)	-C(22)	178.4(1)
C(9)	-C(10)	-C(11)	-C(12)	0.8(2)	C(20)	-C(21)	-C(22)	-C(17)	-1.3(2)
C(10)	-C(11)	-C(12)	-C(13)	-0.9(2)	C(18)	-C(17)	-C(22)	-C(21)	1.7(2)

C(11) -C(12) -C(13) -C(14)	0.3(2)	C(16) -C(17) -C(22) -C(21)	-178.7(1)

Supplementary Table 5. Crystallographic data of **2n**.

Crystallised from		hexane / CH_2Cl_2/ EtOAc
Empirical formula		$C_{19}H_{18}O_3$
Formula weight [g mol^{-1}]		294.33
Crystal colour, habit		colourless, prism
Crystal dimensions [mm]		0.10 × 0.10 × 0.32
Temperature [K]		160(1)
Crystal system		monoclinic
Space group		$P2_1/c$ (#14)
Z		4
Reflections for cell determination		6601
2θ range for cell determination [°]		12–152
Unit cell parameters	a [Å]	7.96792(9)
	b [Å]	25.5109(3)
	c [Å]	7.49506(7)
	α [°]	90
	β [°]	98.9932(9)
	γ [°]	90
	V [Å^3]	1504.78(3)
F(000)		624
D_X [g cm^{-3}]		1.299
μ(Cu $K\alpha$) [mm^{-1}]		0.700
Scan type		ω
$2\theta_{(max)}$ [°]		152.3
Transmission factors (min; max)		0.824; 1.000
Total reflections measured		24865
Symmetry independent reflections		5245
R_{int}		0.049
Reflections with $I > 2\sigma(I)$		4764

Reflections used in refinement	5245
Parameters refined	203
Final *R*(*F*) [$I > 2\sigma(I)$ reflections]	0.0383
wR(F^2) (all data)	0.1130
Weights: $w = [\sigma^2(F_o^2) + (0.0695P)^2 + 0.1575P]^{-1}$ where $P = (F_o^2 + 2F_c^2)/3$	
Goodness of fit	1.048
Secondary extinction coefficient	0.0027(8)
Final Δ_{max}/σ	0.001
$\Delta\rho$ (max; min) [e Å^{-3}]	0.25; -0.18
$\sigma(d_{(C-C)})$ [Å]	0.0015 – 0.002

Supplementary Table 6. Bond lengths (Å) of **2n** with standard uncertainties in parentheses.

O(1)	-C(1)	1.2157(15)	C(5)	-C(10)	1.4056(16)
O(2)	-C(18)	1.2068(15)	C(6)	-C(7)	1.3909(16)
O(3)	-C(18)	1.3310(15)	C(7)	-C(8)	1.3928(17)
O(3)	-C(19)	1.4493(15)	C(7)	-C(18)	1.4932(16)
C(1)	-C(10)	1.4959(16)	C(8)	-C(9)	1.3790(18)
C(1)	-C(2)	1.4990(18)	C(9)	-C(10)	1.3961(17)
C(2)	-C(3)	1.5303(16)	C(12)	-C(13)	1.3868(16)
C(3)	-C(11)	1.5234(16)	C(12)	-C(17)	1.3934(16)
C(3)	-C(4)	1.5425(15)	C(13)	-C(14)	1.3893(18)
C(4)	-C(12)	1.5200(15)	C(14)	-C(15)	1.378(2)
C(4)	-C(5)	1.5250(15)	C(15)	-C(16)	1.384(2)
C(5)	-C(6)	1.3957(16)	C(16)	-C(17)	1.3867(17)

Supplementary Table 7. Bond angles (°) of **2n** with standard uncertainties in parentheses.

C(18)	-O(3)	-C(19)	115.61(10)	C(8)	-C(7)	-C(18)	117.93(11)
O(1)	-C(1)	-C(10)	121.54(12)	C(9)	-C(8)	-C(7)	119.33(11)
O(1)	-C(1)	-C(2)	121.86(11)	C(8)	-C(9)	-C(10)	120.61(11)
C(10)	-C(1)	-C(2)	116.55(10)	C(9)	-C(10)	-C(5)	120.59(11)
C(1)	-C(2)	-C(3)	113.13(10)	C(9)	-C(10)	-C(1)	118.29(11)
C(11)	-C(3)	-C(2)	110.16(10)	C(5)	-C(10)	-C(1)	121.03(11)
C(11)	-C(3)	-C(4)	111.82(10)	C(13)	-C(12)	-C(17)	118.73(11)
C(2)	-C(3)	-C(4)	109.65(9)	C(13)	-C(12)	-C(4)	120.79(10)
C(12)	-C(4)	-C(5)	112.86(9)	C(17)	-C(12)	-C(4)	120.38(10)
C(12)	-C(4)	-C(3)	110.29(9)	C(12)	-C(13)	-C(14)	120.62(12)
C(5)	-C(4)	-C(3)	111.56(9)	C(15)	-C(14)	-C(13)	120.29(12)
C(6)	-C(5)	-C(10)	118.08(11)	C(14)	-C(15)	-C(16)	119.63(12)

C(6)	-C(5)	-C(4)	120.21(10)	C(15)	-C(16)	-C(17)	120.26(12)
C(10)	-C(5)	-C(4)	121.64(10)	C(16)	-C(17)	-C(12)	120.47(12)
C(7)	-C(6)	-C(5)	120.90(11)	O(2)	-C(18)	-O(3)	123.56(11)
C(6)	-C(7)	-C(8)	120.46(11)	O(2)	-C(18)	-C(7)	123.64(12)
C(6)	-C(7)	-C(18)	121.59(11)	O(3)	-C(18)	-C(7)	112.80(10)

Supplementary Table 8. Torsion angles (°) of **2n** with standard uncertainties in parentheses.

O(1)	-C(1)	-C(2)	-C(3)	148.3(1)	C(4)	-C(5)	-C(10)	-C(1)	2.0(2)
C(10)	-C(1)	-C(2)	-C(3)	-34.2(2)	O(1)	-C(1)	-C(10)	-C(9)	4.5(2)
C(1)	-C(2)	-C(3)	-C(11)	-178.3(1)	C(2)	-C(1)	-C(10)	-C(9)	-173.0(1)
C(1)	-C(2)	-C(3)	-C(4)	58.3(1)	O(1)	-C(1)	-C(10)	-C(5)	-178.9(1)
C(11)	-C(3)	-C(4)	-C(12)	60.0(1)	C(2)	-C(1)	-C(10)	-C(5)	3.6(2)
C(2)	-C(3)	-C(4)	-C(12)	-177.51(9)	C(5)	-C(4)	-C(12)	-C(13)	126.4(1)
C(11)	-C(3)	-C(4)	-C(5)	-173.74(9)	C(3)	-C(4)	-C(12)	-C(13)	-108.1(1)
C(2)	-C(3)	-C(4)	-C(5)	-51.3(1)	C(5)	-C(4)	-C(12)	-C(17)	-57.1(1)
C(12)	-C(4)	-C(5)	-C(6)	-35.6(1)	C(3)	-C(4)	-C(12)	-C(17)	68.4(1)
C(3)	-C(4)	-C(5)	-C(6)	-160.4(1)	C(17)	-C(12)	-C(13)	-C(14)	-0.2(2)
C(12)	-C(4)	-C(5)	-C(10)	147.4(1)	C(4)	-C(12)	-C(13)	-C(14)	176.4(1)
C(3)	-C(4)	-C(5)	-C(10)	22.6(1)	C(12)	-C(13)	-C(14)	-C(15)	-0.1(2)
C(10)	-C(5)	-C(6)	-C(7)	-0.3(2)	C(13)	-C(14)	-C(15)	-C(16)	0.3(2)
C(4)	-C(5)	-C(6)	-C(7)	-177.4(1)	C(14)	-C(15)	-C(16)	-C(17)	-0.4(2)
C(5)	-C(6)	-C(7)	-C(8)	-0.6(2)	C(15)	-C(16)	-C(17)	-C(12)	0.1(2)
C(5)	-C(6)	-C(7)	-C(18)	178.0(1)	C(13)	-C(12)	-C(17)	-C(16)	0.1(2)
C(6)	-C(7)	-C(8)	-C(9)	0.3(2)	C(4)	-C(12)	-C(17)	-C(16)	-176.4(1)
C(18)	-C(7)	-C(8)	-C(9)	-178.3(1)	C(19)	-O(3)	-C(18)	-O(2)	-1.2(2)
C(7)	-C(8)	-C(9)	-C(10)	0.8(2)	C(19)	-O(3)	-C(18)	-C(7)	179.6(1)
C(8)	-C(9)	-C(10)	-C(5)	-1.8(2)	C(6)	-C(7)	-C(18)	-O(2)	-168.0(1)
C(8)	-C(9)	-C(10)	-C(1)	174.8(1)	C(8)	-C(7)	-C(18)	-O(2)	10.7(2)
C(6)	-C(5)	-C(10)	-C(9)	1.5(2)	C(6)	-C(7)	-C(18)	-O(3)	11.2(2)
C(4)	-C(5)	-C(10)	-C(9)	178.6(1)	C(8)	-C(7)	-C(18)	-O(3)	-170.2(1)
C(6)	-C(5)	-C(10)	-C(1)	-175.0(1)					

Supplementary Table 9. Crystallographic data of **2s.**

Crystallised from	CH_2Cl_2/ hexane
Empirical formula	$C_{21}H_{19}NO$
Formula weight [g mol^{-1}]	301.37
Crystal colour, habit	colourless, prism
Crystal dimensions [mm]	$0.12 \times 0.13 \times 0.35$
Temperature [K]	160(1)
Crystal system	monoclinic
Space group	$P2_1/c$ (#14)
Z	8

Reflections for cell determination		42946
2[illegible] range for cell determination [°]		4−52
Unit cell parameters	*a* [Å]	13.6303(2)
	b [Å]	13.6026(2)
	c [Å]	17.1902(3)
	α [°]	90
	β [°]	91.6698(9)
	γ [°]	90
	V [Å^3]	3185.84(9)
F(000)		1280
D_X [g cm^{-3}]		1.257
μ(Mo $K\alpha$) [mm^{-1}]		0.0770
Scan type		ω
2θ(max) [°]		52.1
Transmission factors (min; max)		0.926; 0.993
Total reflections measured		51970
Symmetry independent reflections		6272
R_{int}		0.071
Reflections with $I > 2\sigma(I)$		4462
Reflections used in refinement		6272
Parameters refined		420
Final	$R(F)$ [$I > 2\sigma(I)$ reflections]	0.0485
	$wR(F^2)$ (all data)	0.1284
Weights:	w = [[illegible]$^2(F_o^2) + (0.0576P)^2 + 0.8011P]^{-1}$ where $P = (F_o^2 + 2F_c^2)/3$	
Goodness of fit		1.067
Secondary extinction coefficient		0.0072(9)
Final Δ_{max}/σ		0.000
$\Delta\rho$ (max; min) [e Å^{-3}]		0.28; -0.34
$\sigma(d_{(C-C)})$ [Å]		0.002 – 0.003

Supplementary Table 10. Bond lengths (Å) of **2s** with standard uncertainties in parentheses.

O(1)	-C(1)	1.223(2)	O(2)	-C(31)	1.223(2)
N(1)	-C(12)	1.309(2)	N(2)	-C(42)	1.304(2)
N(1)	-C(11)	1.375(2)	N(2)	-C(41)	1.373(2)
C(1)	-C(13)	1.486(2)	C(31)	-C(43)	1.489(2)
C(1)	-C(2)	1.496(2)	C(31)	-C(32)	1.496(2)
C(2)	-C(3)	1.536(2)	C(32)	-C(33)	1.537(2)

C(3)	-C(14)	1.534(2)	C(33)	-C(45)	1.528(2)
C(3)	-C(15)	1.534(2)	C(33)	-C(44)	1.532(2)
C(3)	-C(4)	1.565(2)	C(33)	-C(34)	1.561(2)
C(4)	-C(5)	1.519(2)	C(34)	-C(35)	1.520(2)
C(4)	-C(16)	1.532(2)	C(34)	-C(46)	1.530(2)
C(5)	-C(13)	1.383(2)	C(35)	-C(43)	1.383(2)
C(5)	-C(6)	1.432(2)	C(35)	-C(36)	1.429(2)
C(6)	-C(7)	1.419(2)	C(36)	-C(37)	1.414(2)
C(6)	-C(11)	1.423(2)	C(36)	-C(41)	1.426(2)
C(7)	-C(8)	1.366(2)	C(37)	-C(38)	1.368(2)
C(8)	-C(9)	1.406(2)	C(38)	-C(39)	1.405(2)
C(9)	-C(10)	1.363(2)	C(39)	-C(40)	1.364(3)
C(10)	-C(11)	1.413(2)	C(40)	-C(41)	1.412(2)
C(12)	-C(13)	1.412(2)	C(42)	-C(43)	1.417(2)
C(16)	-C(21)	1.393(2)	C(46)	-C(51)	1.393(2)
C(16)	-C(17)	1.393(2)	C(46)	-C(47)	1.395(2)
C(17)	-C(18)	1.387(2)	C(47)	-C(48)	1.388(3)
C(18)	-C(19)	1.387(3)	C(48)	-C(49)	1.383(3)
C(19)	-C(20)	1.384(3)	C(49)	-C(50)	1.378(3)
C(20)	-C(21)	1.387(2)	C(50)	-C(51)	1.391(2)

Supplementary Table 11. Bond angles (°) of **2s** with standard uncertainties in parentheses.

C(12)	-N(1)	-C(11)	116.27(15)	C(42)	-N(2)	-C(41)	116.74(15)
O(1)	-C(1)	-C(13)	120.18(16)	O(2)	-C(31)	-C(43)	120.76(16)
O(1)	-C(1)	-C(2)	122.47(17)	O(2)	-C(31)	-C(32)	122.20(17)
C(13)	-C(1)	-C(2)	117.34(14)	C(43)	-C(31)	-C(32)	117.02(14)
C(1)	-C(2)	-C(3)	112.99(14)	C(31)	-C(32)	-C(33)	113.51(14)
C(14)	-C(3)	-C(15)	107.76(14)	C(45)	-C(33)	-C(44)	107.85(14)
C(14)	-C(3)	-C(2)	109.78(14)	C(45)	-C(33)	-C(32)	109.40(14)
C(15)	-C(3)	-C(2)	108.89(14)	C(44)	-C(33)	-C(32)	109.26(14)
C(14)	-C(3)	-C(4)	109.74(13)	C(45)	-C(33)	-C(34)	112.00(13)
C(15)	-C(3)	-C(4)	111.56(14)	C(44)	-C(33)	-C(34)	109.03(13)
C(2)	-C(3)	-C(4)	109.08(13)	C(32)	-C(33)	-C(34)	109.26(13)
C(5)	-C(4)	-C(16)	109.74(13)	C(35)	-C(34)	-C(46)	111.33(13)
C(5)	-C(4)	-C(3)	111.60(13)	C(35)	-C(34)	-C(33)	110.69(13)
C(16)	-C(4)	-C(3)	113.60(13)	C(46)	-C(34)	-C(33)	112.91(13)
C(13)	-C(5)	-C(6)	117.01(14)	C(43)	-C(35)	-C(36)	117.44(15)
C(13)	-C(5)	-C(4)	121.30(15)	C(43)	-C(35)	-C(34)	121.09(15)
C(6)	-C(5)	-C(4)	121.63(14)	C(36)	-C(35)	-C(34)	121.47(14)
C(7)	-C(6)	-C(11)	118.00(15)	C(37)	-C(36)	-C(41)	118.05(15)
C(7)	-C(6)	-C(5)	123.55(14)	C(37)	-C(36)	-C(35)	123.60(15)

C(11)	-C(6)	-C(5)	118.45(15)	C(41)	-C(36)	-C(35)	118.35(15)
C(8)	-C(7)	-C(6)	120.88(15)	C(38)	-C(37)	-C(36)	120.78(15)
C(7)	-C(8)	-C(9)	120.82(16)	C(37)	-C(38)	-C(39)	120.97(17)
C(10)	-C(9)	-C(8)	119.90(17)	C(40)	-C(39)	-C(38)	119.82(17)
C(9)	-C(10)	-C(11)	120.88(16)	C(39)	-C(40)	-C(41)	120.84(16)
N(1)	-C(11)	-C(10)	117.34(15)	N(2)	-C(41)	-C(40)	117.63(15)
N(1)	-C(11)	-C(6)	123.16(16)	N(2)	-C(41)	-C(36)	122.83(16)
C(10)	-C(11)	-C(6)	119.50(16)	C(40)	-C(41)	-C(36)	119.53(16)
N(1)	-C(12)	-C(13)	125.44(16)	N(2)	-C(42)	-C(43)	125.43(17)
C(5)	-C(13)	-C(12)	119.68(16)	C(35)	-C(43)	-C(42)	119.21(16)
C(5)	-C(13)	-C(1)	121.88(15)	C(35)	-C(43)	-C(31)	122.24(15)
C(12)	-C(13)	-C(1)	118.43(15)	C(42)	-C(43)	-C(31)	118.54(15)
C(21)	-C(16)	-C(17)	118.20(15)	C(51)	-C(46)	-C(47)	118.24(15)
C(21)	-C(16)	-C(4)	122.00(14)	C(51)	-C(46)	-C(34)	122.12(14)
C(17)	-C(16)	-C(4)	119.79(14)	C(47)	-C(46)	-C(34)	119.53(14)
C(18)	-C(17)	-C(16)	121.17(16)	C(48)	-C(47)	-C(46)	120.68(17)
C(19)	-C(18)	-C(17)	120.06(16)	C(49)	-C(48)	-C(47)	120.25(17)
C(20)	-C(19)	-C(18)	119.25(16)	C(50)	-C(49)	-C(48)	119.76(17)
C(19)	-C(20)	-C(21)	120.66(17)	C(49)	-C(50)	-C(51)	120.20(17)
C(20)	-C(21)	-C(16)	120.64(16)	C(50)	-C(51)	-C(46)	120.78(16)

Supplementary Table 12. Torsion angles (°) of **2s** with standard uncertainties in parentheses.

O(1)	-C(1)	-C(2)	-C(3)	-148.0(2)	O(2)	-C(31)	-C(32)	-C(33)	151.4(2)
C(13)	-C(1)	-C(2)	-C(3)	33.2(2)	C(43)	-C(31)	-C(32)	-C(33)	-29.6(2)
C(1)	-C(2)	-C(3)	-C(14)	64.0(2)	C(31)	-C(32)	-C(33)	-C(45)	178.5(1)
C(1)	-C(2)	-C(3)	-C(15)	-178.2(1)	C(31)	-C(32)	-C(33)	-C(44)	-63.7(2)
C(1)	-C(2)	-C(3)	-C(4)	-56.2(2)	C(31)	-C(32)	-C(33)	-C(34)	55.5(2)
C(14)	-C(3)	-C(4)	-C(5)	-68.5(2)	C(45)	-C(33)	-C(34)	-C(35)	-175.2(1)
C(15)	-C(3)	-C(4)	-C(5)	172.1(1)	C(44)	-C(33)	-C(34)	-C(35)	65.5(2)
C(2)	-C(3)	-C(4)	-C(5)	51.8(2)	C(32)	-C(33)	-C(34)	-C(35)	-53.9(2)
C(14)	-C(3)	-C(4)	-C(16)	166.8(1)	C(45)	-C(33)	-C(34)	-C(46)	-49.7(2)
C(15)	-C(3)	-C(4)	-C(16)	47.4(2)	C(44)	-C(33)	-C(34)	-C(46)	-169.0(1)
C(2)	-C(3)	-C(4)	-C(16)	-72.9(2)	C(32)	-C(33)	-C(34)	-C(46)	71.7(2)
C(16)	-C(4)	-C(5)	-C(13)	101.0(2)	C(46)	-C(34)	-C(35)	-C(43)	-97.9(2)
C(3)	-C(4)	-C(5)	-C(13)	-25.9(2)	C(33)	-C(34)	-C(35)	-C(43)	28.5(2)
C(16)	-C(4)	-C(5)	-C(6)	-76.0(2)	C(46)	-C(34)	-C(35)	-C(36)	81.3(2)
C(3)	-C(4)	-C(5)	-C(6)	157.1(1)	C(33)	-C(34)	-C(35)	-C(36)	-152.2(1)
C(13)	-C(5)	-C(6)	-C(7)	-179.8(2)	C(43)	-C(35)	-C(36)	-C(37)	-180.0(2)
C(4)	-C(5)	-C(6)	-C(7)	-2.7(2)	C(34)	-C(35)	-C(36)	-C(37)	0.7(2)
C(13)	-C(5)	-C(6)	-C(11)	-0.3(2)	C(43)	-C(35)	-C(36)	-C(41)	-0.5(2)
C(4)	-C(5)	-C(6)	-C(11)	176.8(1)	C(34)	-C(35)	-C(36)	-C(41)	-179.8(1)
C(11)	-C(6)	-C(7)	-C(8)	-0.5(2)	C(41)	-C(36)	-C(37)	-C(38)	0.8(2)
C(5)	-C(6)	-C(7)	-C(8)	179.0(2)	C(35)	-C(36)	-C(37)	-C(38)	-179.7(2)
C(6)	-C(7)	-C(8)	-C(9)	0.3(3)	C(36)	-C(37)	-C(38)	-C(39)	-0.9(2)
C(7)	-C(8)	-C(9)	-C(10)	0.4(3)	C(37)	-C(38)	-C(39)	-C(40)	0.2(3)
C(8)	-C(9)	-C(10)	-C(11)	-1.0(3)	C(38)	-C(39)	-C(40)	-C(41)	0.4(3)
C(12)	-N(1)	-C(11)	-C(10)	-179.9(2)	C(42)	-N(2)	-C(41)	-C(40)	179.9(2)
C(12)	-N(1)	-C(11)	-C(6)	0.1(2)	C(42)	-N(2)	-C(41)	-C(36)	-0.6(3)
C(9)	-C(10)	-C(11)	-N(1)	-179.1(2)	C(39)	-C(40)	-C(41)	-N(2)	179.1(2)
C(9)	-C(10)	-C(11)	-C(6)	0.8(3)	C(39)	-C(40)	-C(41)	-C(36)	-0.4(3)
C(7)	-C(6)	-C(11)	-N(1)	179.8(2)	C(37)	-C(36)	-C(41)	-N(2)	-179.7(2)

C(5)	-C(6)	-C(11)	-N(1)	0.3(2)	C(35)	-C(36)	-C(41)	-N(2)	0.8(2)
C(7)	-C(6)	-C(11)	-C(10)	-0.1(2)	C(37)	-C(36)	-C(41)	-C(40)	-0.2(2)
C(5)	-C(6)	-C(11)	-C(10)	-179.6(2)	C(35)	-C(36)	-C(41)	-C(40)	-179.7(2)
C(11)	-N(1)	-C(12)	-C(13)	-0.6(3)	C(41)	-N(2)	-C(42)	-C(43)	0.1(3)
C(6)	-C(5)	-C(13)	-C(12)	-0.1(2)	C(36)	-C(35)	-C(43)	-C(42)	0.1(2)
C(4)	-C(5)	-C(13)	-C(12)	-177.3(1)	C(34)	-C(35)	-C(43)	-C(42)	179.4(2)
C(6)	-C(5)	-C(13)	-C(1)	178.7(1)	C(36)	-C(35)	-C(43)	-C(31)	178.9(2)
C(4)	-C(5)	-C(13)	-C(1)	1.6(2)	C(34)	-C(35)	-C(43)	-C(31)	-1.8(2)
N(1)	-C(12)	-C(13)	-C(5)	0.6(3)	N(2)	-C(42)	-C(43)	-C(35)	0.2(3)
N(1)	-C(12)	-C(13)	-C(1)	-178.2(2)	N(2)	-C(42)	-C(43)	-C(31)	-178.7(2)
O(1)	-C(1)	-C(13)	-C(5)	176.3(2)	O(2)	-C(31)	-C(43)	-C(35)	-179.2(2)
C(2)	-C(1)	-C(13)	-C(5)	-4.9(2)	C(32)	-C(31)	-C(43)	-C(35)	1.8(2)
O(1)	-C(1)	-C(13)	-C(12)	-4.8(3)	O(2)	-C(31)	-C(43)	-C(42)	-0.4(3)
C(2)	-C(1)	-C(13)	-C(12)	174.0(2)	C(32)	-C(31)	-C(43)	-C(42)	-179.4(2)
C(5)	-C(4)	-C(16)	-C(21)	-38.0(2)	C(35)	-C(34)	-C(46)	-C(51)	38.7(2)
C(3)	-C(4)	-C(16)	-C(21)	87.7(2)	C(33)	-C(34)	-C(46)	-C(51)	-86.5(2)
C(5)	-C(4)	-C(16)	-C(17)	143.5(2)	C(35)	-C(34)	-C(46)	-C(47)	-145.0(2)
C(3)	-C(4)	-C(16)	-C(17)	-90.8(2)	C(33)	-C(34)	-C(46)	-C(47)	89.7(2)
C(21)	-C(16)	-C(17)	-C(18)	-1.3(2)	C(51)	-C(46)	-C(47)	-C(48)	2.8(3)
C(4)	-C(16)	-C(17)	-C(18)	177.3(2)	C(34)	-C(46)	-C(47)	-C(48)	-173.5(2)
C(16)	-C(17)	-C(18)	-C(19)	0.7(3)	C(46)	-C(47)	-C(48)	-C(49)	-0.4(3)
C(17)	-C(18)	-C(19)	-C(20)	-0.1(3)	C(47)	-C(48)	-C(49)	-C(50)	-1.9(3)
C(18)	-C(19)	-C(20)	-C(21)	0.0(3)	C(48)	-C(49)	-C(50)	-C(51)	1.7(3)
C(19)	-C(20)	-C(21)	-C(16)	-0.6(3)	C(49)	-C(50)	-C(51)	-C(46)	0.9(3)
C(17)	-C(16)	-C(21)	-C(20)	1.2(2)	C(47)	-C(46)	-C(51)	-C(50)	-3.1(3)
C(4)	-C(16)	-C(21)	-C(20)	-177.3(2)	C(34)	-C(46)	-C(51)	-C(50)	173.2(2)

Supplementary Table 13. Crystallographic data of **3**.

Crystallised from		CH_2Cl_2/ hexane / EtOAc
Empirical formula		$C_{21}H_{23}NO_3$
Formula weight [g mol^{-1}]		337.40
Crystal colour, habit		colourless, needle
Crystal dimensions [mm]		0.07 × 0.13 × 0.30
Temperature [K]		160(1)
Crystal system		monoclinic
Space group		$P2_1/c$ (#14)
Z		4
Reflections for cell determination		11076
2θ range for cell determination [°]		5−153
Unit cell parameters	*a* [Å]	16.35605(13)
	b [Å]	11.18341(10)
	c [Å]	9.87304(9)
	α [°]	90

β [°]	97.1416(8)
γ [°]	90
V [Å^3]	1791.93(3)
F(000)	720
D_x [g cm^{-3}]	1.251
μ(Cu $K\alpha$) [mm^{-1}]	0.667
Scan type	ω
$2\theta_{(max)}$ [°]	153.0
Transmission factors (min; max)	0.713; 1.000
Total reflections measured	18925
Symmetry independent reflections	3731
R_{int}	0.018
Reflections with $I > 2\sigma(I)$	3446
Reflections used in refinement	3731
Parameters refined	234
Final $R(F)$ [$I > 2\sigma(I)$ reflections]	0.0344
$wR(F^2)$ (all data)	0.0930
Weights:	$w = [\sigma^2(F_o^2) + (0.0456P)^2 + 0.4776P]^{-1}$ where $P = (F_o^2 + 2F_c^2)/3$
Goodness of fit	1.063
Secondary extinction coefficient	0.0011(2)
Final Δ_{max}/σ	0.001
$\Delta\rho$ (max; min) [e Å^{-3}]	0.26; -0.22
$\sigma(d_{(C-C)})$ [Å]	0.0012 – 0.0019

Supplementary Table 14. Bond lengths (Å) of **3** with standard uncertainties in parentheses.

O(1)	-C(1)	1.2180(14)	C(6)	-C(7)	1.3860(14)
O(2)	-C(11)	1.2181(13)	C(7)	-C(8)	1.3890(17)
O(3)	-C(11)	1.3418(12)	C(8)	-C(9)	1.3766(16)
O(3)	-C(12)	1.4791(11)	C(9)	-C(10)	1.3990(14)
N(1)	-C(11)	1.3531(12)	C(12)	-C(13)	1.5161(18)
N(1)	-C(3)	1.4555(12)	C(12)	-C(15)	1.5162(16)
C(1)	-C(10)	1.4823(14)	C(12)	-C(14)	1.5248(17)
C(1)	-C(2)	1.5032(15)	C(16)	-C(17)	1.3883(14)

C(2)	-C(3)	1.5310(13)	C(16)	-C(21)	1.3928(14)
C(3)	-C(4)	1.5389(12)	C(17)	-C(18)	1.3984(16)
C(4)	-C(16)	1.5219(13)	C(18)	-C(19)	1.3758(19)
C(4)	-C(5)	1.5269(12)	C(19)	-C(20)	1.3835(17)
C(5)	-C(6)	1.3975(13)	C(20)	-C(21)	1.3885(15)
C(5)	-C(10)	1.3999(14)			

Supplementary Table 15. Bond angles (°) of **3** with standard uncertainties in parentheses.

C(11)	-O(3)	-C(12)	121.86(8)	C(9)	-C(10)	-C(1)	118.84(9)
C(11)	-N(1)	-C(3)	120.42(8)	C(5)	-C(10)	-C(1)	120.62(9)
O(1)	-C(1)	-C(10)	122.18(10)	O(2)	-C(11)	-O(3)	125.94(9)
O(1)	-C(1)	-C(2)	122.41(10)	O(2)	-C(11)	-N(1)	124.40(9)
C(10)	-C(1)	-C(2)	115.40(9)	O(3)	-C(11)	-N(1)	109.65(8)
C(1)	-C(2)	-C(3)	110.49(8)	O(3)	-C(12)	-C(13)	109.80(9)
N(1)	-C(3)	-C(2)	111.29(8)	O(3)	-C(12)	-C(15)	110.93(9)
N(1)	-C(3)	-C(4)	109.13(8)	C(13)	-C(12)	-C(15)	112.46(10)
C(2)	-C(3)	-C(4)	111.39(8)	O(3)	-C(12)	-C(14)	101.35(8)
C(16)	-C(4)	-C(5)	110.69(7)	C(13)	-C(12)	-C(14)	111.99(11)
C(16)	-C(4)	-C(3)	111.40(7)	C(15)	-C(12)	-C(14)	109.78(11)
C(5)	-C(4)	-C(3)	111.95(8)	C(17)	-C(16)	-C(21)	118.48(9)
C(6)	-C(5)	-C(10)	118.00(9)	C(17)	-C(16)	-C(4)	121.56(9)
C(6)	-C(5)	-C(4)	119.09(9)	C(21)	-C(16)	-C(4)	119.94(9)
C(10)	-C(5)	-C(4)	122.91(8)	C(16)	-C(17)	-C(18)	120.29(10)
C(7)	-C(6)	-C(5)	120.96(10)	C(19)	-C(18)	-C(17)	120.68(10)
C(6)	-C(7)	-C(8)	120.57(10)	C(18)	-C(19)	-C(20)	119.39(10)
C(9)	-C(8)	-C(7)	119.17(10)	C(19)	-C(20)	-C(21)	120.24(11)
C(8)	-C(9)	-C(10)	120.72(10)	C(20)	-C(21)	-C(16)	120.91(10)
C(9)	-C(10)	-C(5)	120.39(9)				

Supplementary Table 16. Torsion angles (°) of **3** with standard uncertainties in parentheses.

O(1)	-C(1)	-C(2)	-C(3)	136.4(1)	C(4)	-C(5)	-C(10)	-C(1)	8.8(1)
C(10)	-C(1)	-C(2)	-C(3)	-45.0(1)	O(1)	-C(1)	-C(10)	-C(9)	13.9(2)
C(11)	-N(1)	-C(3)	-C(2)	73.9(1)	C(2)	-C(1)	-C(10)	-C(9)	-164.71(9)
C(11)	-N(1)	-C(3)	-C(4)	-162.78(8)	O(1)	-C(1)	-C(10)	-C(5)	-170.7(1)
C(1)	-C(2)	-C(3)	-N(1)	-176.83(8)	C(2)	-C(1)	-C(10)	-C(5)	10.7(1)
C(1)	-C(2)	-C(3)	-C(4)	61.1(1)	C(12)	-O(3)	-C(11)	-O(2)	-1.7(2)
N(1)	-C(3)	-C(4)	-C(16)	70.4(1)	C(12)	-O(3)	-C(11)	-N(1)	179.60(9)
C(2)	-C(3)	-C(4)	-C(16)	-166.34(8)	C(3)	-N(1)	-C(11)	-O(2)	11.0(2)
N(1)	-C(3)	-C(4)	-C(5)	-165.06(7)	C(3)	-N(1)	-C(11)	-O(3)	-170.29(8)
C(2)	-C(3)	-C(4)	-C(5)	-41.8(1)	C(11)	-O(3)	-C(12)	-C(13)	-64.4(1)
C(16)	-C(4)	-C(5)	-C(6)	-47.6(1)	C(11)	-O(3)	-C(12)	-C(15)	60.6(1)
C(3)	-C(4)	-C(5)	-C(6)	-172.53(8)	C(11)	-O(3)	-C(12)	-C(14)	177.1(1)
C(16)	-C(4)	-C(5)	-C(10)	132.54(9)	C(5)	-C(4)	-C(16)	-C(17)	118.01(9)
C(3)	-C(4)	-C(5)	-C(10)	7.6(1)	C(3)	-C(4)	-C(16)	-C(17)	-116.74(9)

C(10)	-C(5)	-C(6)	-C(7)	-1.1(2)	C(5)	-C(4)	-C(16)	-C(21)	-60.4(1)
C(4)	-C(5)	-C(6)	-C(7)	179.07(9)	C(3)	-C(4)	-C(16)	-C(21)	64.8(1)
C(5)	-C(6)	-C(7)	-C(8)	-2.5(2)	C(21)	-C(16)	-C(17)	-C(18)	-0.6(1)
C(6)	-C(7)	-C(8)	-C(9)	2.8(2)	C(4)	-C(16)	-C(17)	-C(18)	-179.03(9)
C(7)	-C(8)	-C(9)	-C(10)	0.5(2)	C(16)	-C(17)	-C(18)	-C(19)	-0.3(2)
C(8)	-C(9)	-C(10)	-C(5)	-4.0(2)	C(17)	-C(18)	-C(19)	-C(20)	0.8(2)
C(8)	-C(9)	-C(10)	-C(1)	171.4(1)	C(18)	-C(19)	-C(20)	-C(21)	-0.4(2)
C(6)	-C(5)	-C(10)	-C(9)	4.3(1)	C(19)	-C(20)	-C(21)	-C(16)	-0.5(2)
C(4)	-C(5)	-C(10)	-C(9)	-175.87(9)	C(17)	-C(16)	-C(21)	-C(20)	1.0(2)
C(6)	-C(5)	-C(10)	-C(1)	-171.13(9)	C(4)	-C(16)	-C(21)	-C(20)	179.48(9)

Supplementary Table 17. Selected bond lengths (Å) and angles (°) involving H-atoms of **3**.

N(1)	-H(1)		0.868(14)				
C(11)	-N(1)	-H(1)	116.1(9)	C(3)	-N(1)	-H(1)	117.4(9)

Supplementary Table 18. Hydrogen bonding geometry (Å, °) of **3**.

D	H	A	D-H	H···A	D···A	D-H···A
N(1)	-H(1)	···O(2')	0.868(14)	2.172(14)	2.9351(11)	146.5(12)

Primed atoms refer to the molecule in the following symmetry related positions:

' $x, \frac{1}{2}-y, \frac{1}{2}+z$

Intermolecular N-H···O hydrogen bonds between the amide N-H group and the amide O-atom of a neighbouring molecule link the molecules into extended chains which run parallel to the [001] direction and can be described by a graph set motif[8] of C(4).

Supplementary Table 19. Crystallographic data of **6**.

Crystallised from	$CDCl_3$ / EtOAc
Empirical formula	$C_{30}H_{34}O_2$
Formula weight [g mol^{-1}]	426.57
Crystal colour, habit	colourless, tablet
Crystal dimensions [mm]	0.07 × 0.20 × 0.22
Temperature [K]	160(1)
Crystal system	triclinic
Space group	*P*1 (#1)
Z	2
Reflections for cell determination	15542
2θ range for cell determination [°]	6–153
Unit cell parameters *a* [Å]	7.09493(18)

b [Å]	13.0276(3)
c [Å]	13.3193(4)
α [°]	92.888(2)
β [°]	104.910(2)
γ [°]	91.376(2)
V [Å^3]	1187.26(5)
F(000)	460
D_X [g cm^{-3}]	1.193
μ(Cu $K\alpha$) [mm^{-1}]	0.560
Scan type	ω
$2\theta_{(max)}$ [°]	153.3
Transmission factors (min; max)	0.730; 1.000
Total reflections measured	24109
Symmetry independent reflections	8530
R_{int}	0.023
Reflections with $I > 2\sigma(I)$	8297
Reflections used in refinement	8530
Parameters refined; restraints	638; 204
Final $R(F)$ [$I > 2\sigma(I)$ reflections]	0.0337
$wR(F^2)$ (all data)	0.0905
Weights:	$w = [\sigma^2(F_o^2) + (0.061P)^2 + 0.0803P]^{-1}$ where $P = (F_o^2 + 2F_c^2)/3$
Goodness of fit	1.021
Final Δ_{max}/σ	0.002
$\Delta\rho$ (max; min) [e Å^{-3}]	0.16; -0.17
$\sigma(d_{(C-C)})$ [Å]	0.002 – 0.007

Supplementary Table 20. Bond lengths (Å) of **6** with standard uncertainties in parentheses.

O(1)	-C(1)	1.204(3)	C(25b)	-C(26b)	1.380(7)
O(2)	-C(18)	1.211(3)	C(26b)	-C(27b)	1.393(6)
C(1)	-C(9)	1.515(3)	C(27b)	-C(28b)	1.399(5)
C(1)	-C(2)	1.528(3)	O(3)	-C(31)	1.204(3)
C(2)	-C(3)	1.548(3)	O(4)	-C(48)	1.216(3)
C(3)	-C(4)	1.538(3)	C(31)	-C(39)	1.519(3)

C(4)	-C(5)	1.519(2)
C(4)	-C(9)	1.543(2)
C(5)	-C(11)	1.524(2)
C(5)	-C(6)	1.541(2)
C(6)	-C(22)	1.524(2)
C(6)	-C(7)	1.541(2)
C(7)	-C(8)	1.537(3)
C(8)	-C(9)	1.518(3)
C(9)	-C(10)	1.543(3)
C(11)	-C(12)	1.526(2)
C(12)	-C(13)	1.517(2)
C(13)	-C(22)	1.406(2)
C(13)	-C(14)	1.411(2)
C(14)	-C(19)	1.401(2)
C(14)	-C(15)	1.522(2)
C(15)	-C(23a)	1.520(4)
C(15)	-C(23b)	1.541(4)
C(15)	-C(16)	1.561(2)
C(16)	-C(29)	1.532(3)
C(16)	-C(30)	1.535(3)
C(16)	-C(17)	1.535(2)
C(17)	-C(18)	1.499(3)
C(18)	-C(19)	1.494(3)
C(19)	-C(20)	1.397(3)
C(20)	-C(21)	1.374(3)
C(21)	-C(22)	1.402(2)
C(23a)	-C(24a)	1.393(5)
C(23a)	-C(28a)	1.403(5)
C(24a)	-C(25a)	1.397(5)
C(25a)	-C(26a)	1.378(7)
C(26a)	-C(27a)	1.393(6)
C(27a)	-C(28a)	1.402(5)
C(23b)	-C(24b)	1.401(5)
C(23b)	-C(28b)	1.405(5)
C(24b)	-C(25b)	1.395(5)
C(31)	-C(32)	1.532(3)
C(32)	-C(33)	1.545(3)
C(33)	-C(34)	1.531(3)
C(34)	-C(35)	1.523(2)
C(34)	-C(39)	1.541(2)
C(35)	-C(41)	1.525(2)
C(35)	-C(36)	1.538(2)
C(36)	-C(52)	1.522(2)
C(36)	-C(37)	1.539(2)
C(37)	-C(38)	1.541(3)
C(38)	-C(39)	1.519(3)
C(39)	-C(40)	1.545(3)
C(41)	-C(42)	1.520(3)
C(42)	-C(43)	1.515(2)
C(43)	-C(52)	1.409(2)
C(43)	-C(44)	1.414(2)
C(44)	-C(49)	1.405(2)
C(44)	-C(45)	1.528(2)
C(45)	-C(53)	1.528(2)
C(45)	-C(46)	1.564(2)
C(46)	-C(59)	1.525(2)
C(46)	-C(47)	1.536(2)
C(46)	-C(60)	1.537(2)
C(47)	-C(48)	1.495(3)
C(48)	-C(49)	1.488(3)
C(49)	-C(50)	1.393(3)
C(50)	-C(51)	1.371(3)
C(51)	-C(52)	1.405(2)
C(53)	-C(54)	1.391(3)
C(53)	-C(58)	1.397(3)
C(54)	-C(55)	1.399(4)
C(55)	-C(56)	1.386(5)
C(56)	-C(57)	1.370(5)
C(57)	-C(58)	1.386(3)

Supplementary Table 21. Bond angles (°) of **6** with standard uncertainties in parentheses.

O(1)	-C(1)	-C(9)	126.9(2)
O(1)	-C(1)	-C(2)	125.0(2)
C(9)	-C(1)	-C(2)	107.99(17)
C(1)	-C(2)	-C(3)	105.38(17)
C(4)	-C(3)	-C(2)	102.12(17)
C(5)	-C(4)	-C(3)	120.18(16)
C(5)	-C(4)	-C(9)	113.40(14)
C(3)	-C(4)	-C(9)	104.01(14)
C(25b)	-C(24b)	-C(23b)	120.3(4)
C(26b)	-C(25b)	-C(24b)	120.5(4)
C(25b)	-C(26b)	-C(27b)	120.2(3)
C(26b)	-C(27b)	-C(28b)	119.9(4)
C(27b)	-C(28b)	-C(23b)	120.3(4)
O(3)	-C(31)	-C(39)	127.1(2)
O(3)	-C(31)	-C(32)	125.2(2)
C(39)	-C(31)	-C(32)	107.69(17)

C(4)	-C(5)	-C(11)	111.32(14)	C(31)	-C(32)	-C(33)	105.50(19)
C(4)	-C(5)	-C(6)	108.46(14)	C(34)	-C(33)	-C(32)	102.36(18)
C(11)	-C(5)	-C(6)	108.71(13)	C(35)	-C(34)	-C(33)	120.45(15)
C(22)	-C(6)	-C(5)	111.52(14)	C(35)	-C(34)	-C(39)	112.89(14)
C(22)	-C(6)	-C(7)	113.71(15)	C(33)	-C(34)	-C(39)	103.50(16)
C(5)	-C(6)	-C(7)	112.02(14)	C(34)	-C(35)	-C(41)	111.92(14)
C(8)	-C(7)	-C(6)	112.18(16)	C(34)	-C(35)	-C(36)	108.36(13)
C(9)	-C(8)	-C(7)	110.94(16)	C(41)	-C(35)	-C(36)	108.07(14)
C(1)	-C(9)	-C(8)	116.93(17)	C(52)	-C(36)	-C(35)	112.01(13)
C(1)	-C(9)	-C(10)	107.24(15)	C(52)	-C(36)	-C(37)	114.42(15)
C(8)	-C(9)	-C(10)	110.02(17)	C(35)	-C(36)	-C(37)	111.90(15)
C(1)	-C(9)	-C(4)	98.47(15)	C(36)	-C(37)	-C(38)	112.12(16)
C(8)	-C(9)	-C(4)	110.09(15)	C(39)	-C(38)	-C(37)	110.04(16)
C(10)	-C(9)	-C(4)	113.80(16)	C(38)	-C(39)	-C(31)	116.78(16)
C(5)	-C(11)	-C(12)	111.23(14)	C(38)	-C(39)	-C(34)	109.62(15)
C(13)	-C(12)	-C(11)	115.05(15)	C(31)	-C(39)	-C(34)	100.22(15)
C(22)	-C(13)	-C(14)	119.83(14)	C(38)	-C(39)	-C(40)	111.31(18)
C(22)	-C(13)	-C(12)	121.14(15)	C(31)	-C(39)	-C(40)	104.78(17)
C(14)	-C(13)	-C(12)	118.99(15)	C(34)	-C(39)	-C(40)	113.76(16)
C(19)	-C(14)	-C(13)	119.66(15)	C(42)	-C(41)	-C(35)	111.53(14)
C(19)	-C(14)	-C(15)	120.02(15)	C(43)	-C(42)	-C(41)	114.83(15)
C(13)	-C(14)	-C(15)	120.28(14)	C(52)	-C(43)	-C(44)	120.19(14)
C(23a)	-C(15)	-C(14)	112.9(8)	C(52)	-C(43)	-C(42)	120.44(16)
C(14)	-C(15)	-C(23b)	108.1(7)	C(44)	-C(43)	-C(42)	119.33(15)
C(23a)	-C(15)	-C(16)	113.7(12)	C(49)	-C(44)	-C(43)	119.30(15)
C(14)	-C(15)	-C(16)	111.62(14)	C(49)	-C(44)	-C(45)	120.40(15)
C(23b)	-C(15)	-C(16)	114.6(11)	C(43)	-C(44)	-C(45)	120.30(14)
C(29)	-C(16)	-C(30)	108.29(16)	C(53)	-C(45)	-C(44)	110.44(14)
C(29)	-C(16)	-C(17)	109.18(16)	C(53)	-C(45)	-C(46)	113.83(14)
C(30)	-C(16)	-C(17)	109.76(16)	C(44)	-C(45)	-C(46)	112.61(14)
C(29)	-C(16)	-C(15)	111.72(15)	C(59)	-C(46)	-C(47)	109.02(16)
C(30)	-C(16)	-C(15)	108.85(15)	C(59)	-C(46)	-C(60)	107.98(15)
C(17)	-C(16)	-C(15)	109.02(15)	C(47)	-C(46)	-C(60)	109.46(15)
C(18)	-C(17)	-C(16)	112.71(16)	C(59)	-C(46)	-C(45)	112.43(15)
O(2)	-C(18)	-C(19)	121.8(2)	C(47)	-C(46)	-C(45)	109.13(15)
O(2)	-C(18)	-C(17)	120.7(2)	C(60)	-C(46)	-C(45)	108.77(15)
C(19)	-C(18)	-C(17)	117.55(16)	C(48)	-C(47)	-C(46)	111.84(16)
C(20)	-C(19)	-C(14)	120.08(17)	O(4)	-C(48)	-C(49)	121.1(2)
C(20)	-C(19)	-C(18)	117.94(16)	O(4)	-C(48)	-C(47)	121.8(2)
C(14)	-C(19)	-C(18)	121.97(17)	C(49)	-C(48)	-C(47)	117.10(17)
C(21)	-C(20)	-C(19)	120.00(16)	C(50)	-C(49)	-C(44)	120.00(17)
C(20)	-C(21)	-C(22)	121.46(17)	C(50)	-C(49)	-C(48)	118.16(16)
C(21)	-C(22)	-C(13)	118.93(16)	C(44)	-C(49)	-C(48)	121.84(16)
C(21)	-C(22)	-C(6)	120.26(16)	C(51)	-C(50)	-C(49)	120.56(16)
C(13)	-C(22)	-C(6)	120.75(14)	C(50)	-C(51)	-C(52)	121.33(17)

C(24a)-C(23a)-C(28a)	120.1(3)	C(51) -C(52) -C(43)	118.60(16)
C(24a)-C(23a)-C(15)	123.6(7)	C(51) -C(52) -C(36)	120.00(15)
C(28a)-C(23a)-C(15)	116.3(6)	C(43) -C(52) -C(36)	121.30(14)
C(23a)-C(24a)-C(25a)	120.1(4)	C(54) -C(53) -C(58)	118.0(2)
C(26a)-C(25a)-C(24a)	119.9(4)	C(54) -C(53) -C(45)	120.03(19)
C(25a)-C(26a)-C(27a)	120.8(3)	C(58) -C(53) -C(45)	121.99(18)
C(26a)-C(27a)-C(28a)	119.9(4)	C(53) -C(54) -C(55)	120.7(3)
C(27a)-C(28a)-C(23a)	119.2(4)	C(56) -C(55) -C(54)	120.0(3)
C(24b)-C(23b)-C(28b)	118.9(3)	C(57) -C(56) -C(55)	119.8(2)
C(24b)-C(23b)-C(15)	120.2(6)	C(56) -C(57) -C(58)	120.3(3)
C(28b)-C(23b)-C(15)	120.9(6)	C(57) -C(58) -C(53)	121.2(2)

Supplementary Table 22. Torsion angles (°) of **6** with standard uncertainties in parentheses.

O(1) -C(1) -C(2) -C(3)	-162.9(2)	C(28b)-C(23b)-C(24b)-C(25b)	-2(3)
C(9) -C(1) -C(2) -C(3)	12.7(2)	C(15) -C(23b)-C(24b)-C(25b)	178(2)
C(1) -C(2) -C(3) -C(4)	16.6(2)	C(23b)-C(24b)-C(25b)-C(26b)	2(2)
C(2) -C(3) -C(4) -C(5)	-168.1(2)	C(24b)-C(25b)-C(26b)-C(27b)	-2(1)
C(2) -C(3) -C(4) -C(9)	-39.9(2)	C(25b)-C(26b)-C(27b)-C(28b)	1(1)
C(3) -C(4) -C(5) -C(11)	-59.5(2)	C(26b)-C(27b)-C(28b)-C(23b)	0(2)
C(9) -C(4) -C(5) -C(11)	176.7(2)	C(24b)-C(23b)-C(28b)-C(27b)	0(3)
C(3) -C(4) -C(5) -C(6)	-179.0(2)	C(15) -C(23b)-C(28b)-C(27b)	-179(2)
C(9) -C(4) -C(5) -C(6)	57.2(2)	O(3) -C(31) -C(32) -C(33)	-173.4(3)
C(4) -C(5) -C(6) -C(22)	176.6(1)	C(39) -C(31) -C(32) -C(33)	6.4(3)
C(11) -C(5) -C(6) -C(22)	55.4(2)	C(31) -C(32) -C(33) -C(34)	21.4(3)
C(4) -C(5) -C(6) -C(7)	-54.7(2)	C(32) -C(33) -C(34) -C(35)	-168.7(2)
C(11) -C(5) -C(6) -C(7)	-175.9(2)	C(32) -C(33) -C(34) -C(39)	-41.5(2)
C(22) -C(6) -C(7) -C(8)	-177.8(2)	C(33) -C(34) -C(35) -C(41)	-60.4(2)
C(5) -C(6) -C(7) -C(8)	54.6(2)	C(39) -C(34) -C(35) -C(41)	176.8(2)
C(6) -C(7) -C(8) -C(9)	-54.1(2)	C(33) -C(34) -C(35) -C(36)	-179.4(2)
O(1) -C(1) -C(9) -C(8)	21.6(3)	C(39) -C(34) -C(35) -C(36)	57.7(2)
C(2) -C(1) -C(9) -C(8)	-153.9(2)	C(34) -C(35) -C(36) -C(52)	175.4(1)
O(1) -C(1) -C(9) -C(10)	-102.4(2)	C(41) -C(35) -C(36) -C(52)	53.9(2)
C(2) -C(1) -C(9) -C(10)	82.1(2)	C(34) -C(35) -C(36) -C(37)	-54.6(2)
O(1) -C(1) -C(9) -C(4)	139.3(2)	C(41) -C(35) -C(36) -C(37)	-176.1(2)
C(2) -C(1) -C(9) -C(4)	-36.2(2)	C(52) -C(36) -C(37) -C(38)	-176.2(2)
C(7) -C(8) -C(9) -C(1)	165.7(2)	C(35) -C(36) -C(37) -C(38)	55.0(2)
C(7) -C(8) -C(9) -C(10)	-71.7(2)	C(36) -C(37) -C(38) -C(39)	-55.4(2)
C(7) -C(8) -C(9) -C(4)	54.5(2)	C(37) -C(38) -C(39) -C(31)	169.5(2)
C(5) -C(4) -C(9) -C(1)	179.0(2)	C(37) -C(38) -C(39) -C(34)	56.4(2)
C(3) -C(4) -C(9) -C(1)	46.8(2)	C(37) -C(38) -C(39) -C(40)	-70.3(2)
C(5) -C(4) -C(9) -C(8)	-58.1(2)	O(3) -C(31) -C(39) -C(38)	30.3(3)
C(3) -C(4) -C(9) -C(8)	169.6(2)	C(32) -C(31) -C(39) -C(38)	-149.5(2)
C(5) -C(4) -C(9) -C(10)	65.9(2)	O(3) -C(31) -C(39) -C(34)	148.6(2)
C(3) -C(4) -C(9) -C(10)	-66.4(2)	C(32) -C(31) -C(39) -C(34)	-31.2(2)

C(4)	-C(5)	-C(11)	-C(12)	178.1(2)
C(6)	-C(5)	-C(11)	-C(12)	-62.5(2)
C(5)	-C(11)	-C(12)	-C(13)	40.8(2)
C(11)	-C(12)	-C(13)	-C(22)	-12.7(2)
C(11)	-C(12)	-C(13)	-C(14)	169.6(2)
C(22)	-C(13)	-C(14)	-C(19)	-2.2(2)
C(12)	-C(13)	-C(14)	-C(19)	175.5(2)
C(22)	-C(13)	-C(14)	-C(15)	180.0(2)
C(12)	-C(13)	-C(14)	-C(15)	-2.3(2)
C(19)	-C(14)	-C(15)	-C(23a)	-102(1)
C(13)	-C(14)	-C(15)	-C(23a)	76(1)
C(19)	-C(14)	-C(15)	-C(23b)	-99(1)
C(13)	-C(14)	-C(15)	-C(23b)	79(1)
C(19)	-C(14)	-C(15)	-C(16)	27.8(2)
C(13)	-C(14)	-C(15)	-C(16)	-154.4(2)
C(23a)	-C(15)	-C(16)	-C(29)	-46.2(5)
C(14)	-C(15)	-C(16)	-C(29)	-175.3(2)
C(23b)	-C(15)	-C(16)	-C(29)	-52.0(5)
C(23a)	-C(15)	-C(16)	-C(30)	-165.7(5)
C(14)	-C(15)	-C(16)	-C(30)	65.2(2)
C(23b)	-C(15)	-C(16)	-C(30)	-171.6(5)
C(23a)	-C(15)	-C(16)	-C(17)	74.6(5)
C(14)	-C(15)	-C(16)	-C(17)	-54.5(2)
C(23b)	-C(15)	-C(16)	-C(17)	68.7(5)
C(29)	-C(16)	-C(17)	-C(18)	179.0(2)
C(30)	-C(16)	-C(17)	-C(18)	-62.5(2)
C(15)	-C(16)	-C(17)	-C(18)	56.7(2)
C(16)	-C(17)	-C(18)	-O(2)	149.1(2)
C(16)	-C(17)	-C(18)	-C(19)	-31.2(2)
C(13)	-C(14)	-C(19)	-C(20)	1.5(3)
C(15)	-C(14)	-C(19)	-C(20)	179.3(2)
C(13)	-C(14)	-C(19)	-C(18)	-179.0(2)
C(15)	-C(14)	-C(19)	-C(18)	-1.2(3)
O(2)	-C(18)	-C(19)	-C(20)	1.7(3)
C(17)	-C(18)	-C(19)	-C(20)	-178.0(2)
O(2)	-C(18)	-C(19)	-C(14)	-177.8(2)
C(17)	-C(18)	-C(19)	-C(14)	2.5(3)
C(14)	-C(19)	-C(20)	-C(21)	-0.2(3)
C(18)	-C(19)	-C(20)	-C(21)	-179.7(2)
C(19)	-C(20)	-C(21)	-C(22)	-0.5(3)
C(20)	-C(21)	-C(22)	-C(13)	-0.2(3)
C(20)	-C(21)	-C(22)	-C(6)	176.9(2)
C(14)	-C(13)	-C(22)	-C(21)	1.6(2)
C(12)	-C(13)	-C(22)	-C(21)	-176.1(2)
C(14)	-C(13)	-C(22)	-C(6)	-175.6(2)
O(3)	-C(31)	-C(39)	-C(40)	-93.3(3)
C(32)	-C(31)	-C(39)	-C(40)	86.9(2)
C(35)	-C(34)	-C(39)	-C(38)	-59.9(2)
C(33)	-C(34)	-C(39)	-C(38)	168.3(2)
C(35)	-C(34)	-C(39)	-C(31)	176.8(2)
C(33)	-C(34)	-C(39)	-C(31)	44.9(2)
C(35)	-C(34)	-C(39)	-C(40)	65.5(2)
C(33)	-C(34)	-C(39)	-C(40)	-66.3(2)
C(34)	-C(35)	-C(41)	-C(42)	177.6(1)
C(36)	-C(35)	-C(41)	-C(42)	-63.2(2)
C(35)	-C(41)	-C(42)	-C(43)	43.1(2)
C(41)	-C(42)	-C(43)	-C(52)	-14.1(2)
C(41)	-C(42)	-C(43)	-C(44)	168.3(2)
C(52)	-C(43)	-C(44)	-C(49)	-1.1(2)
C(42)	-C(43)	-C(44)	-C(49)	176.5(2)
C(52)	-C(43)	-C(44)	-C(45)	179.6(1)
C(42)	-C(43)	-C(44)	-C(45)	-2.7(2)
C(49)	-C(44)	-C(45)	-C(53)	-107.1(2)
C(43)	-C(44)	-C(45)	-C(53)	72.2(2)
C(49)	-C(44)	-C(45)	-C(46)	21.4(2)
C(43)	-C(44)	-C(45)	-C(46)	-159.3(1)
C(53)	-C(45)	-C(46)	-C(59)	-44.5(2)
C(44)	-C(45)	-C(46)	-C(59)	-171.2(1)
C(53)	-C(45)	-C(46)	-C(47)	76.6(2)
C(44)	-C(45)	-C(46)	-C(47)	-50.1(2)
C(53)	-C(45)	-C(46)	-C(60)	-164.1(2)
C(44)	-C(45)	-C(46)	-C(60)	69.2(2)
C(59)	-C(46)	-C(47)	-C(48)	-177.8(2)
C(60)	-C(46)	-C(47)	-C(48)	-59.9(2)
C(45)	-C(46)	-C(47)	-C(48)	59.0(2)
C(46)	-C(47)	-C(48)	-O(4)	142.3(2)
C(46)	-C(47)	-C(48)	-C(49)	-38.5(2)
C(43)	-C(44)	-C(49)	-C(50)	1.4(2)
C(45)	-C(44)	-C(49)	-C(50)	-179.3(2)
C(43)	-C(44)	-C(49)	-C(48)	-178.7(2)
C(45)	-C(44)	-C(49)	-C(48)	0.5(2)
O(4)	-C(48)	-C(49)	-C(50)	7.2(3)
C(47)	-C(48)	-C(49)	-C(50)	-172.1(2)
O(4)	-C(48)	-C(49)	-C(44)	-172.7(2)
C(47)	-C(48)	-C(49)	-C(44)	8.0(3)
C(44)	-C(49)	-C(50)	-C(51)	-0.8(3)
C(48)	-C(49)	-C(50)	-C(51)	179.3(2)
C(49)	-C(50)	-C(51)	-C(52)	-0.1(3)
C(50)	-C(51)	-C(52)	-C(43)	0.3(2)
C(50)	-C(51)	-C(52)	-C(36)	176.8(2)

C(12) -C(13) -C(22) -C(6)	6.7(2)	C(44) -C(43) -C(52) -C(51)	0.3(2)
C(5) -C(6) -C(22) -C(21)	154.5(2)	C(42) -C(43) -C(52) -C(51)	-177.4(2)
C(7) -C(6) -C(22) -C(21)	26.6(2)	C(44) -C(43) -C(52) -C(36)	-176.2(1)
C(5) -C(6) -C(22) -C(13)	-28.4(2)	C(42) -C(43) -C(52) -C(36)	6.2(2)
C(7) -C(6) -C(22) -C(13)	-156.3(2)	C(35) -C(36) -C(52) -C(51)	156.8(2)
C(14) -C(15) -C(23a)-C(24a)	38(3)	C(37) -C(36) -C(52) -C(51)	28.1(2)
C(16) -C(15) -C(23a)-C(24a)	-90(2)	C(35) -C(36) -C(52) -C(43)	-26.8(2)
C(14) -C(15) -C(23a)-C(28a)	-139(2)	C(37) -C(36) -C(52) -C(43)	-155.5(2)
C(16) -C(15) -C(23a)-C(28a)	92(2)	C(44) -C(45) -C(53) -C(54)	-145.2(2)
C(28a)-C(23a)-C(24a)-C(25a)	-3(3)	C(46) -C(45) -C(53) -C(54)	87.0(2)
C(15) -C(23a)-C(24a)-C(25a)	180(2)	C(44) -C(45) -C(53) -C(58)	32.9(2)
C(23a)-C(24a)-C(25a)-C(26a)	0(2)	C(46) -C(45) -C(53) -C(58)	-94.9(2)
C(24a)-C(25a)-C(26a)-C(27a)	2(1)	C(58) -C(53) -C(54) -C(55)	1.6(3)
C(25a)-C(26a)-C(27a)-C(28a)	-1(1)	C(45) -C(53) -C(54) -C(55)	179.8(2)
C(26a)-C(27a)-C(28a)-C(23a)	-2(2)	C(53) -C(54) -C(55) -C(56)	-0.7(4)
C(24a)-C(23a)-C(28a)-C(27a)	4(3)	C(54) -C(55) -C(56) -C(57)	-0.7(4)
C(15) -C(23a)-C(28a)-C(27a)	-179(1)	C(55) -C(56) -C(57) -C(58)	1.3(4)
C(14) -C(15) -C(23b)-C(24b)	35(2)	C(56) -C(57) -C(58) -C(53)	-0.4(4)
C(16) -C(15) -C(23b)-C(24b)	-90(2)	C(54) -C(53) -C(58) -C(57)	-1.0(3)
C(14) -C(15) -C(23b)-C(28b)	-145(2)	C(45) -C(53) -C(58) -C(57)	-179.2(2)
C(16) -C(15) -C(23b)-C(28b)	89(2)		

Supplementary Table 23. Computed absolute electronic energies (hartrees) and relative free Gibbs energies (kcal/mol) for all structures, and imaginary frequencies for the transition states

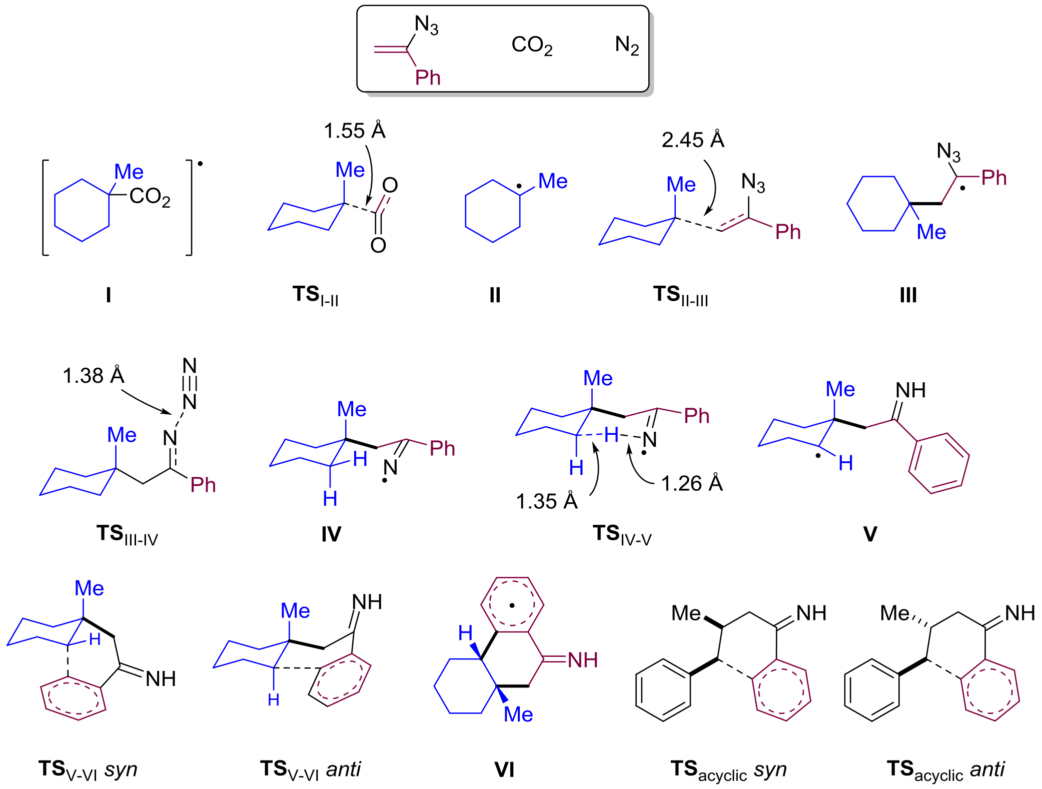

	E(M06-2X)	Correction to G	G(M06-2X)	G(relative)	Freq

CO_2	-188.577683	-0.009451	-188.587134		--
N_3-styrene	-473.163626	0.102121	-473.061505		--
N_2	-109.522521	-0.012687	-109.535208		--
**I**	-463.012738	0.162676	-462.850062	**0**	--
**TS**$_{I-II}$	-463.011575			<5	--
**II**	-274.460117	0.151762	-274.308356	-28.5	--
**TS**$_{II-III}$	-747.220871	0.281368	-746.939503	-16.3	-251.8
**III**	-747.684703	0.284215	-747.400488	-47.7	--
**TS**$_{III-IV}$	-747.676256			<5	--
**IV**	-638.232086	0.275373	-637.956714	-105.1	--
**TS**$_{IV-V}$	-638.197340	0.271976	-637.956714	-85.4	-1695.2
**V**	-638.219419	0.275852	-637.943567	-96.8	--
**TS**$_{V-VI}$ *syn*	-638.203678	0.278448	-637.925230	-85.3	-498.0
**TS**$_{V-VI}$ *anti*	-638.194829	0.277485	-637.917344	-80.4	-452.6
**VI**	-638.230609	0.280508	-637.950102	-101.0	--
**TS**$_{acyclic}$ *syn*	-713.201110	0.262142	-712.938968	**2.7***	-555.3
**TS**$_{acyclic}$ *anti*	-713.205538	0.262228	-712.943310	**0***	-574.8

*The G value of **TS**$_{acyclic}$ *syn* (2.7) is relative to **TS**$_{acyclic}$ *syn* (0)

TS$_{I-II}$ and TS$_{III-IV}$ could not be located by standard procedures due to the spontaneous character of those transformations. We conducted *relaxed* PES scans for the fragmentation trajectories, showing very early transition states ($?_{TS}$-$?_{substrate}$ ≈ 0.05 Å) and almost non existing activation barriers in both cases.

Cartesian coordinates of the computed structures

CO_2

Standard orientation:

Center Number	Atomic Number	Atomic Type	Coordinates (Angstroms) X	Y	Z
1	6	0	0.000000	0.000000	-0.000001
2	8	0	0.000000	0.000000	1.154795
3	8	0	0.000000	0.000000	-1.154794

N_2

Standard orientation:

Center Number	Atomic Number	Atomic Type	Coordinates (Angstroms) X	Y	Z
1	7	0	0.000000	0.000000	0.544938
2	7	0	0.000000	0.000000	-0.544938

Styrene-N3

Standard orientation:

Center Number	Atomic Number	Atomic Type	Coordinates (Angstroms) X	Y	Z
1	6	0	1.466654	1.648625	0.427752
2	1	0	2.534289	1.832238	0.441451
3	1	0	0.818686	2.459864	0.726771
4	6	0	0.951204	0.467056	0.073366
5	7	0	1.739591	-0.670129	-0.280593
6	7	0	2.958959	-0.530583	-0.163232
7	7	0	4.075560	-0.488858	-0.087935
8	6	0	-0.500325	0.167644	0.017973
9	6	0	-3.242529	-0.392559	-0.025669
10	6	0	-1.428753	1.185620	-0.222500
11	6	0	-0.960673	-1.136720	0.217298
12	6	0	-2.323980	-1.412919	0.200186
13	6	0	-2.789846	0.907204	-0.239990
14	1	0	-1.085352	2.194711	-0.417234
15	1	0	-0.246883	-1.932116	0.392837
16	1	0	-2.667882	-2.427459	0.363184
17	1	0	-3.497938	1.704532	-0.432308
18	1	0	-4.304207	-0.608474	-0.042877

I

Standard orientation:

Center Number	Atomic Number	Atomic Type	Coordinates (Angstroms) X	Y	Z
1	1	0	-0.130261	-0.825758	-2.004161
2	6	0	0.344186	-0.804108	-1.018479
3	6	0	2.504176	-0.423533	0.228085
4	6	0	0.321084	0.311802	1.253796
5	6	0	1.814976	0.614540	1.114192
6	6	0	-0.381391	0.225170	-0.122593
7	6	0	1.838864	-0.498230	-1.146537
8	1	0	2.439285	-1.406134	0.710920
9	1	0	0.199568	-0.650285	1.761460
10	1	0	1.961646	1.614826	0.693593
11	1	0	1.989495	0.445630	-1.680444
12	1	0	0.220997	-1.795376	-0.571925
13	1	0	3.565774	-0.186835	0.122522
14	1	0	-0.171495	1.073157	1.866075
15	1	0	2.265202	0.625333	2.109908
16	1	0	2.305836	-1.277675	-1.753665
17	6	0	-1.771509	-0.337750	0.149329
18	8	0	-2.033445	-1.427166	0.607437
19	8	0	-2.762680	0.478022	-0.146026
20	6	0	-0.472626	1.599859	-0.797488

21	1	0	0.524659	1.997194	-0.984164
22	1	0	-0.999851	2.318148	-0.164950
23	1	0	-0.988410	1.534426	-1.758288

II

Standard orientation:

Center Number	Atomic Number	Atomic Type	Coordinates (Angstroms) X	Y	Z
1	1	0	-0.879055	-2.144052	0.435653
2	6	0	-0.260660	-1.258516	0.607207
3	6	0	1.799575	-0.000077	-0.145660
4	6	0	-0.260523	1.258578	0.607182
5	6	0	0.959020	1.264878	-0.340470
6	6	0	-1.052632	0.000073	0.413572
7	6	0	0.958887	-1.264948	-0.340440
8	1	0	2.214321	-0.000083	0.870450
9	1	0	0.113272	1.311044	1.638727
10	1	0	0.606737	1.310644	-1.377728
11	1	0	0.606607	-1.310693	-1.377700
12	1	0	0.113113	-1.311032	1.638756
13	1	0	2.648215	-0.000131	-0.835317
14	1	0	-0.878823	2.144180	0.435645
15	1	0	1.564808	2.158739	-0.165808
16	1	0	1.564581	-2.158869	-0.165765
17	6	0	-2.182778	0.000048	-0.565774
18	1	0	-1.817232	-0.000820	-1.605814
19	1	0	-2.810498	0.888082	-0.452954
20	1	0	-2.811383	-0.887230	-0.451844

TS$_{II\text{-}III}$

Standard orientation:

Center Number	Atomic Number	Atomic Type	Coordinates (Angstroms) X	Y	Z
1	1	0	3.110115	1.565915	-0.640280
2	6	0	2.985180	0.476895	-0.656445
3	6	0	4.056953	-1.745449	-0.064034
4	6	0	1.552977	-1.431397	0.160511
5	6	0	2.805430	-2.139118	0.736027
6	6	0	1.776974	0.058862	0.139853
7	6	0	4.249817	-0.221331	-0.095027
8	1	0	3.952610	-2.114027	-1.094598
9	1	0	1.398708	-1.802666	-0.864447
10	1	0	2.936522	-1.847312	1.786775
11	1	0	4.438625	0.143436	0.923673
12	1	0	2.863337	0.171756	-1.707207
13	1	0	4.943972	-2.228756	0.360192
14	1	0	0.659081	-1.685661	0.741876

15	1	0	2.663549	-3.225219	0.717737
16	1	0	5.123640	0.043121	-0.700554
17	6	0	1.430968	0.828670	1.379824
18	1	0	2.064025	0.535663	2.232932
19	1	0	0.390471	0.644773	1.676151
20	1	0	1.569690	1.906342	1.233556
21	6	0	-0.031253	0.832495	-1.336160
22	1	0	0.616449	1.675057	-1.555894
23	1	0	0.127804	-0.055618	-1.932035
24	6	0	-1.139533	0.977120	-0.566831
25	7	0	-1.415599	2.198396	0.133168
26	7	0	-0.608658	3.139607	0.042228
27	7	0	0.063757	4.069946	0.060255
28	6	0	-2.143326	-0.065662	-0.296596
29	6	0	-4.075265	-2.053535	0.218883
30	6	0	-1.975775	-1.376368	-0.779528
31	6	0	-3.293298	0.226044	0.457112
32	6	0	-4.248180	-0.758252	0.709246
33	6	0	-2.931952	-2.356357	-0.526276
34	1	0	-1.091524	-1.638648	-1.348617
35	1	0	-3.432544	1.228339	0.841305
36	1	0	-5.128923	-0.510337	1.290707
37	1	0	-2.782444	-3.359478	-0.908900
38	1	0	-4.817973	-2.818017	0.414717

III

Standard orientation:

Center Number	Atomic Number	Atomic Type	Coordinates (Angstroms) X	Y	Z
1	1	0	1.225591	0.862025	1.432097
2	6	0	1.989218	0.499560	0.733957
3	6	0	4.270279	-0.553177	0.542184
4	6	0	2.477267	-1.072350	-1.154196
5	6	0	3.694691	-1.613021	-0.399118
6	6	0	1.372741	-0.537943	-0.222370
7	6	0	3.201062	-0.032963	1.505035
8	1	0	4.649045	0.285703	-0.055046
9	1	0	2.807651	-0.251634	-1.804154
10	1	0	3.416157	-2.502086	0.176517
11	1	0	2.897047	-0.835044	2.185603
12	1	0	2.326244	1.359236	0.141166
13	1	0	5.119737	-0.959021	1.098074
14	1	0	2.057358	-1.845706	-1.806808
15	1	0	4.454204	-1.933658	-1.117382
16	1	0	3.610787	0.765438	2.129401
17	6	0	0.738318	-1.689436	0.565305
18	1	0	1.465124	-2.180299	1.213494
19	1	0	0.340461	-2.448965	-0.113791
20	1	0	-0.080143	-1.331432	1.195492
21	6	0	0.302182	0.140684	-1.128305
22	1	0	0.809710	0.911999	-1.720890
23	1	0	-0.057353	-0.598671	-1.849166
24	6	0	-0.869911	0.759385	-0.427000
25	7	0	-0.822591	2.082133	-0.011572
26	7	0	0.133897	2.800657	-0.307764
27	7	0	0.947019	3.557843	-0.507315
28	6	0	-2.117716	0.095895	-0.157233

29	6	0	-4.582295	-1.193138	0.356619
30	6	0	-3.156736	0.749050	0.548918
31	6	0	-2.361881	-1.224223	-0.597514
32	6	0	-3.572875	-1.850763	-0.344959
33	6	0	-4.360087	0.111444	0.800148
34	1	0	-3.002292	1.761970	0.897782
35	1	0	-1.600968	-1.765259	-1.143255
36	1	0	-3.730237	-2.862548	-0.700500
37	1	0	-5.135896	0.636514	1.345872
38	1	0	-5.526044	-1.687011	0.553231

IV

Standard orientation:

Center Number	Atomic Number	Atomic Type	Coordinates (Angstroms) X	Y	Z
1	1	0	-1.325285	1.839753	-0.521833
2	6	0	-2.062028	1.145107	-0.106793
3	6	0	-4.283125	0.018471	-0.499173
4	6	0	-2.443460	-1.194205	0.729494
5	6	0	-3.644037	-1.340145	-0.209017
6	6	0	-1.381522	-0.196717	0.220203
7	6	0	-3.250761	0.998254	-1.059654
8	1	0	-4.694125	0.427094	0.432470
9	1	0	-2.806363	-0.843724	1.704284
10	1	0	-3.334522	-1.805890	-1.150844
11	1	0	-2.906611	0.646673	-2.038645
12	1	0	-2.420232	1.588508	0.831945
13	1	0	-5.118593	-0.095325	-1.195115
14	1	0	-1.970478	-2.168283	0.896895
15	1	0	-4.375411	-2.015407	0.243505
16	1	0	-3.707791	1.978049	-1.222814
17	6	0	-0.668509	-0.763917	-1.014178
18	1	0	-1.378719	-1.038784	-1.794922
19	1	0	-0.096291	-1.658807	-0.750840
20	1	0	0.024155	-0.034112	-1.442263
21	6	0	-0.373920	0.002768	1.376267
22	1	0	-0.894100	0.453861	2.224345
23	1	0	0.001964	-0.972030	1.697365
24	6	0	0.809851	0.894978	1.018990
25	7	0	0.757216	2.125007	1.251634
26	6	0	2.042320	0.320421	0.381826
27	6	0	4.365119	-0.693796	-0.788768
28	6	0	2.725885	1.062501	-0.585688
29	6	0	2.529690	-0.931094	0.759486
30	6	0	3.690456	-1.432579	0.178019
31	6	0	3.879705	0.554906	-1.170385
32	1	0	2.338091	2.028992	-0.886255
33	1	0	2.020731	-1.515221	1.516194
34	1	0	4.065984	-2.402078	0.482868

35	1	0	4.398305	1.131767	-1.926942
36	1	0	5.264788	-1.089801	-1.244609

TS$_{IV-V}$

Standard orientation:

Center Number	Atomic Number	Atomic Type	Coordinates (Angstroms) X	Y	Z
1	1	0	-0.891075	-1.556056	-0.456427
2	6	0	-2.005286	-0.803209	-0.630739
3	6	0	-4.400830	-0.234399	-0.576202
4	6	0	-2.630069	1.488652	0.040213
5	6	0	-4.067097	1.000546	0.268519
6	6	0	-1.603269	0.355456	0.264919
7	6	0	-3.376822	-1.373287	-0.370835
8	1	0	-4.396222	0.046340	-1.635903
9	1	0	-2.534091	1.847207	-0.991611
10	1	0	-4.220078	0.772092	1.327885
11	1	0	-3.458310	-1.765257	0.647171
12	1	0	-1.868214	-0.547422	-1.688502
13	1	0	-5.408033	-0.590679	-0.342815
14	1	0	-2.401437	2.332876	0.700082
15	1	0	-4.763299	1.808069	0.026001
16	1	0	-3.595627	-2.196234	-1.055044
17	6	0	-1.572298	-0.041050	1.750295
18	1	0	-2.541743	-0.384284	2.109981
19	1	0	-1.279495	0.822203	2.354641
20	1	0	-0.850836	-0.842198	1.925991
21	6	0	-0.196195	0.782314	-0.155508
22	1	0	-0.222600	1.248070	-1.149064
23	1	0	0.211189	1.524707	0.536455
24	6	0	0.755040	-0.418586	-0.226527
25	7	0	0.365592	-1.614498	-0.377011
26	6	0	2.225918	-0.143966	-0.125060
27	6	0	4.978276	0.328926	0.024469
28	6	0	2.748139	1.105034	-0.467991
29	6	0	3.096007	-1.152804	0.299775
30	6	0	4.462105	-0.916396	0.378820
31	6	0	4.119381	1.336795	-0.401238
32	1	0	2.090870	1.899459	-0.801496
33	1	0	2.686870	-2.118146	0.572495
34	1	0	5.126205	-1.702341	0.718876
35	1	0	4.514195	2.306413	-0.680691
36	1	0	6.044577	0.512508	0.083593

V

Standard orientation:

Center Number	Atomic Number	Atomic Type	Coordinates (Angstroms) X	Y	Z
1	6	0	-1.886171	0.983715	-0.449428
2	6	0	-4.235145	0.112720	-0.559953
3	6	0	-2.522886	-1.022844	0.892038
4	6	0	-3.714004	-1.228828	-0.045723

5	6	0	-1.355981	-0.237856	0.249785
6	6	0	-3.121108	0.877408	-1.285567
7	1	0	-4.586883	0.711089	0.288404
8	1	0	-2.870248	-0.463980	1.769345
9	1	0	-3.428510	-1.858763	-0.895375
10	1	0	-2.888547	0.339594	-2.220667
11	1	0	-1.198651	1.801009	-0.643844
12	1	0	-5.085851	-0.035054	-1.229983
13	1	0	-2.145070	-1.985374	1.253261
14	1	0	-4.504350	-1.763786	0.487564
15	1	0	-3.461498	1.871595	-1.585010
16	6	0	-0.634165	-1.139775	-0.776012
17	1	0	-1.337949	-1.507933	-1.524982
18	1	0	-0.182241	-2.002748	-0.276401
19	1	0	0.155817	-0.597843	-1.300272
20	6	0	-0.393805	0.157144	1.403359
21	1	0	-0.968931	0.717198	2.146158
22	1	0	-0.048826	-0.762127	1.884230
23	6	0	0.778522	1.014532	0.980085
24	7	0	0.779561	2.287575	1.068774
25	6	0	1.982045	0.361095	0.379239
26	6	0	4.240868	-0.805126	-0.792473
27	6	0	2.672626	1.019485	-0.643310
28	6	0	2.437228	-0.887853	0.808102
29	6	0	3.566468	-1.462482	0.231792
30	6	0	3.789417	0.438377	-1.230401
31	1	0	2.312763	1.984857	-0.978351
32	1	0	1.925542	-1.416160	1.603585
33	1	0	3.916889	-2.426144	0.582236
34	1	0	4.306919	0.952809	-2.031860
35	1	0	5.112802	-1.259112	-1.248446
36	1	0	-0.103569	2.609575	1.469783

TS$_{V-VI}$ *syn*

Standard orientation:

Center Number	Atomic Number	Atomic Type	Coordinates (Angstroms) X	Y	Z
1	1	0	-0.759349	0.527966	-2.184306
2	6	0	-1.150379	0.127878	-1.255938
3	6	0	-2.936704	-0.393650	0.858861
4	6	0	-1.214934	0.994452	-0.127435
5	6	0	-2.084684	-0.938471	-1.336547
6	6	0	-2.930832	-1.212597	-0.280707
7	6	0	-2.083366	0.704880	0.920706
8	1	0	-2.116086	-1.548263	-2.232480
9	1	0	-3.613082	-2.053185	-0.340860
10	1	0	-2.071505	1.325858	1.811355
11	1	0	-3.601267	-0.613230	1.685317
12	6	0	-0.213450	2.081317	-0.016713
13	1	0	-1.365247	3.265274	0.904990
14	7	0	-0.398449	3.196597	0.579372
15	6	0	1.142706	1.769937	-0.609230
16	1	0	1.100010	1.894853	-1.699043
17	1	0	1.849872	2.509541	-0.226283
18	6	0	1.643076	0.337851	-0.293697
19	6	0	1.580591	0.089328	1.230685

20	1	0	0.630247	0.456679	1.629389
21	1	0	2.368263	0.682710	1.707056
22	6	0	0.852698	-0.690052	-1.086805
23	1	0	1.031577	-0.613163	-2.159568
24	6	0	3.109516	0.247109	-0.765365
25	1	0	3.185682	0.478525	-1.831006
26	1	0	3.516597	-0.754141	-0.606556
27	1	0	3.728747	0.959979	-0.213084
28	6	0	0.775619	-2.104793	-0.587743
29	1	0	-0.023969	-2.638885	-1.110564
30	1	0	1.711234	-2.618880	-0.859929
31	6	0	1.695129	-1.384320	1.619902
32	1	0	1.615948	-1.478167	2.706175
33	1	0	2.675210	-1.786225	1.337898
34	6	0	0.596952	-2.188747	0.926458
35	1	0	-0.383056	-1.780273	1.201751
36	1	0	0.617687	-3.233879	1.245551

TS$_{V\text{-}VI}$ *anti*

Standard orientation:

Center Number	Atomic Number	Atomic Type	Coordinates (Angstroms) X	Y	Z
1	1	0	-0.852677	-0.339499	-2.080259
2	6	0	-1.236364	-0.582094	-1.096764
3	6	0	-3.062072	-1.087312	0.983496
4	6	0	-1.722619	0.483512	-0.286871
5	6	0	-1.769064	-1.882435	-0.895460
6	6	0	-2.631537	-2.132698	0.152912
7	6	0	-2.611780	0.208648	0.748963
8	1	0	-1.475243	-2.683704	-1.563802
9	1	0	-2.999411	-3.138699	0.320806
10	1	0	-2.922283	1.011428	1.410763
11	1	0	-3.742619	-1.285684	1.802264
12	6	0	-1.106115	1.829727	-0.409572
13	1	0	-2.706157	2.758991	-0.018421
14	7	0	-1.714950	2.933181	-0.198763
15	6	0	0.378802	1.852012	-0.721217
16	1	0	0.523968	1.711514	-1.800425
17	1	0	0.755441	2.845662	-0.466663
18	6	0	1.160850	0.755623	0.032355
19	6	0	0.816393	0.814019	1.531057
20	1	0	-0.199879	0.474493	1.732928
21	1	0	0.908410	1.846180	1.882160
22	6	0	0.890132	-0.584876	-0.611573
23	1	0	1.145143	-0.570416	-1.673221
24	6	0	2.689612	0.961940	-0.139452
25	1	0	2.910241	1.055336	-1.209218
26	1	0	2.984780	1.904502	0.335875
27	6	0	1.497268	-1.765577	0.079223
28	1	0	1.198644	-2.703883	-0.395879
29	1	0	1.485379	0.200030	2.132581
30	6	0	3.498672	-0.203982	0.453621
31	1	0	3.445193	-0.172052	1.545198
32	1	0	4.554029	-0.069762	0.201155
33	6	0	3.031561	-1.582214	-0.039103
34	1	0	3.302148	-1.695549	-1.094798

35	1	0	3.550652	-2.372339	0.510754
36	1	0	1.196455	-1.814564	1.129855

VI

Standard orientation:

Center Number	Atomic Number	Atomic Type	Coordinates (Angstroms) X	Y	Z
1	1	0	-0.857355	0.411556	-2.096816
2	6	0	-0.834881	-0.111661	-1.126358
3	6	0	-3.085632	-0.312759	0.693752
4	6	0	-1.156134	0.924943	-0.086132
5	6	0	-1.863917	-1.194703	-1.202407
6	6	0	-2.902847	-1.282902	-0.338088
7	6	0	-2.220366	0.779487	0.779563
8	1	0	-1.755381	-1.928277	-1.994383
9	1	0	-3.614295	-2.096054	-0.429353
10	1	0	-2.382076	1.515913	1.561014
11	1	0	-3.901365	-0.417322	1.397313
12	6	0	-0.233986	2.069422	-0.031476
13	1	0	-1.437177	3.259537	0.828766
14	7	0	-0.474314	3.216015	0.489411
15	6	0	1.118776	1.794091	-0.644034
16	1	0	1.049967	1.918316	-1.733413
17	1	0	1.827759	2.538689	-0.274023
18	6	0	1.599057	0.365444	-0.326809
19	6	0	1.619116	0.193180	1.212436
20	1	0	0.694437	0.588069	1.644374
21	1	0	2.432131	0.811539	1.608905
22	6	0	0.657897	-0.651393	-1.039897
23	1	0	0.994756	-0.705422	-2.080717
24	6	0	3.020970	0.195029	-0.875485
25	1	0	3.044502	0.392656	-1.950749
26	1	0	3.400570	-0.815764	-0.710511
27	1	0	3.704533	0.894158	-0.385894
28	6	0	0.790106	-2.065908	-0.455213
29	1	0	0.070993	-2.736247	-0.929736
30	1	0	1.782156	-2.450293	-0.718720
31	6	0	1.753671	-1.251975	1.688514
32	1	0	1.700439	-1.280280	2.780100
33	1	0	2.729053	-1.664843	1.406982
34	6	0	0.649202	-2.101585	1.063788
35	1	0	-0.332818	-1.706873	1.357286
36	1	0	0.703181	-3.133422	1.420777

TS$_{\text{acyclic}}$ *syn*

Standard orientation:

Center Number	Atomic Number	Atomic Type	Coordinates (Angstroms) X	Y	Z
1	1	0	0.245049	0.203102	-1.420976
2	6	0	0.844758	0.715637	-0.675053
3	6	0	2.813448	2.352603	0.489434
4	6	0	2.148377	0.201986	-0.395558
5	6	0	0.625866	2.114981	-0.508353
6	6	0	1.579090	2.900780	0.100683
7	6	0	3.091462	1.013194	0.225228
8	1	0	-0.319062	2.541327	-0.825876
9	1	0	1.384018	3.953665	0.269727
10	1	0	4.045959	0.593333	0.526457
11	1	0	3.552735	2.972442	0.981332
12	6	0	2.368135	-1.248904	-0.590275
13	1	0	4.217961	-1.098830	-0.955758
14	7	0	3.489770	-1.806999	-0.839038
15	6	0	1.132245	-2.089965	-0.377253
16	1	0	0.481562	-2.025857	-1.255839
17	1	0	1.441425	-3.131630	-0.278444
18	6	0	0.383033	-1.631504	0.895945
19	6	0	-0.268510	-0.253593	0.792413
20	1	0	1.151236	-1.531363	1.670193
21	6	0	-0.592903	-2.712741	1.379422
22	1	0	-1.328600	-2.967662	0.615159
23	1	0	-0.040024	-3.619847	1.633486
24	1	0	-1.132478	-2.378661	2.268232
25	6	0	-1.635971	-0.044727	0.323222
26	6	0	-2.376624	1.032718	0.841410
27	6	0	-2.227796	-0.816995	-0.693299
28	6	0	-3.658620	1.311352	0.386598
29	1	0	-1.932711	1.649485	1.616365
30	6	0	-3.510332	-0.537008	-1.147415
31	1	0	-1.681406	-1.640393	-1.137641
32	6	0	-4.235269	0.524595	-0.608617
33	1	0	-4.210368	2.142439	0.810751
34	1	0	-3.946456	-1.149430	-1.928221
35	1	0	-5.236427	0.738003	-0.963544
36	1	0	-0.003145	0.404413	1.616662

TS$_{acyclic}$ *anti*

Standard orientation:

Center Number	Atomic Number	Atomic Type	Coordinates (Angstroms) X	Y	Z
1	1	0	0.068278	0.152285	-1.512937
2	6	0	0.644454	0.705662	-0.777903
3	6	0	2.501690	2.496721	0.347274
4	6	0	1.995362	0.301541	-0.535561
5	6	0	0.318955	2.084899	-0.603382
6	6	0	1.217835	2.943514	-0.012583
7	6	0	2.881811	1.186663	0.068598
8	1	0	-0.662757	2.435234	-0.900089
9	1	0	0.942860	3.977253	0.163680
10	1	0	3.873024	0.845073	0.349407

11	1	0	3.198765	3.173391	0.825803
12	6	0	2.328521	-1.127177	-0.727088
13	1	0	4.168654	-0.841762	-1.070748
14	7	0	3.492559	-1.600662	-0.959712
15	6	0	1.154493	-2.055453	-0.541636
16	1	0	0.496949	-1.990221	-1.415145
17	1	0	1.526036	-3.079668	-0.487439
18	6	0	0.361182	-1.711195	0.744187
19	6	0	-0.315662	-0.345563	0.722068
20	6	0	1.267719	-1.835482	1.971978
21	1	0	0.691650	-1.710603	2.891137
22	1	0	1.752297	-2.814512	1.997529
23	1	0	2.048920	-1.069601	1.959196
24	1	0	-0.426471	-2.466950	0.828811
25	1	0	-0.010291	0.318595	1.528332
26	6	0	-1.722627	-0.222422	0.354279
27	6	0	-2.471418	0.880862	0.801589
28	6	0	-2.350814	-1.142087	-0.504227
29	6	0	-3.794879	1.050448	0.418770
30	1	0	-2.000752	1.603320	1.461008
31	6	0	-3.675116	-0.969037	-0.887352
32	1	0	-1.801633	-2.002123	-0.872066
33	6	0	-4.404847	0.125758	-0.428494
34	1	0	-4.354995	1.904360	0.782408
35	1	0	-4.140422	-1.692467	-1.547032
36	1	0	-5.437984	0.257106	-0.726955

Supplementary methods

1. General Information

NMR spectra were recorded on AV2 300, AV2 400 or AV2 500 MHz Bruker spectrometers. Chemical shifts are given in ppm. The spectra are calibrated to the residual ^{1}H and ^{13}C signals of the solvents. Multiplicities are abbreviated as follows: singlet (s), doublet (d), triplet (t), quartet (q), doublet-doublet (dd), quintet (quint), septet (sept), multiplet (m), and broad (br). Infrared spectra were recorded on a JASCO FT/IR-4100 spectrometer. High-resolution electrospray ionization and electronic impact mass spectrometry was performed on a Finnigan MAT 900 (Thermo Finnigan, San Jose, CA; USA) double focusing magnetic sector mass spectrometer. Ten spectra were acquired. A mass accuracy ≤ 2 ppm was obtained in the peak matching acquisition mode by using a solution containing 2 <l PEG200, 2 <l PPG450, and 1.5 mg NaOAc (all obtained from Sigma-Aldrich, CH-Buchs) dissolved in 100 mL MeOH (HPLC Supra grade, Scharlau, E-Barcelona) as internal standard. GC-MS analysis was done on a Finnigan Voyager GC8000 Top.

Materials and Methods: Unless otherwise stated, starting materials were purchased from Aldrich and/or Fluka. Solvents were purchased in HPLC quality, degassed by purging throughly with nitrogen and dried over activated molecular sieves of appropriate size. Alternatively, they were purged with argon and passed through alumina columns in a solvent purification system (Innovative Technology). Conversion was monitored by thin layer chromatography (TLC) using Merck TLC silica gel 60 F254. Compounds were visualized by UVlight at 254 nm and by dipping the plates in an ethanolic vanillin/sulfuric acid solution or an aqueous potassium permanganate solution followed by heating. Flash column chromatography was performed over silica gel (230-400 mesh).

2. General Procedures

Standard Procedure

Vinyl azide (0.3 mmol, 1.5 equiv), carboxylic acid (0.2 mmol, 1.0 equiv), Ag_2CO_3 (0.06 mmol, 0.3 equiv) and $K_2S_2O_8$ (0.4 mmol, 2.0 equiv) were placed in a dry Schlenk-tube. The reaction vessel was evacuated and filled with nitrogen three times. Acetonitrile (0.5 mL), acetone (0.2 mL), distilled water (1.5 mL) and 2,6-lutidine (0.24 mmol, 1.2 equiv) were sequentially added at 25 °C. The reaction mixture was stirred at 50°C for 10 h. The resulting mixture was extracted with EtOAc (15 mL) and the organic layer was washed with brine (10 mL), dried over anhydrous $MgSO_4$, filtered and concentrated under reduced pressure. The crude product was purified by column chromatography on silica gel with hexane:ethyl acetate mixtures as eluent to give the corresponding products in pure form.

3. Synthesis and Characterization of New Substrates and Products

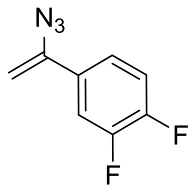

1-(1-Azidovinyl)-3,4-difluorobenzene (**S1**). Colorless oil. This compound was synthesized according to the procedure described by Bi *et al.*[1] ^{1}H NMR (400 MHz, $CDCl_3$): δ 7.38 (ddd, J = 11.6, 7.6, 2.3 Hz, 1H), 7.34 – 7.28 (m, 1H), 7.13 (td, J = 9.9, 8.2 Hz, 1H), 5.41 (d, J = 2.8 Hz, 1H), 4.98 (d, J = 2.8 Hz, 1H). ^{13}C NMR (100 MHz, $CDCl_3$): δ 150.94 (dd, J_{C1-F} = 246.9 Hz, J_{C2-F} = 8.6 Hz), 150.32 (dd, J_{C1-F} = 246.9 Hz, J_{C2-F} = 11.6 Hz), 143.33 (dd, J_{C4-F} = 1.8 Hz, J_{C5-F} = 1.7 Hz), 131.53 (dd,

J_{C3-F} = 6.1 Hz, J_{C4-F} = 3.9 Hz), 121.85 (dd, J_{C3-F} = 6.4 Hz, J_{C4-F} =3.6 Hz), 117.36 (d, J_{C2-F} = 17.6 Hz), 114.93 (d, J_{C2-F} = 19.0 Hz), 98.42 (d, J_{C5-F} = 1.6 Hz). ^{19}F NMR (376 MHz, $CDCl_3$): -136.68 – -136.81 (m, 1F), -137.22 (ddd, *J* = 8.2, 11.6, 20.6 Hz, 1F). IR (film): ν (cm^{-1}) 2117, 1598, 1513, 1431, 1311, 1278, 1117. HR-MS (EI) m/z calcd for $C_8H_5N_3F_2$ [M^+] 181.04461, found 181.04434.

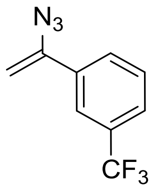

1-(1-Azidovinyl)-3-(trifluoromethyl)benzene (**S2**). Colorless oil. This compound was synthesized according to the procedure described by Chiba *et al.* modified from Hassner's method.[2,3] ^{1}H NMR (400 MHz, $CDCl_3$): δ 7.82 (s, 1H), 7.75 (d, *J* = 7.8 Hz, 1H), 7.60 (d, *J* = 7.8 Hz, 1H), 7.48 (t, *J* = 7.8 Hz, 1H), 5.54 (d, *J* = 2.8 Hz, 1H), 5.05 (d, *J* = 2.8 Hz, 1H). ^{13}C NMR (100 MHz, $CDCl_3$): δ 144.07, 135.24, 131.16 (q, J_{C2-F} = 32.6 Hz), 129.14, 128.86, 125.87 (q, J_{C3-F} = 3.7 Hz), 124.07 (q, J_{C1-F} = 272.5 Hz), 122.60 (q, J_{C3-F} = 3.7 Hz), 99.18. ^{19}F NMR (376 MHz, $CDCl_3$): -62.78 (s, 3F). IR (film): ν (cm^{-1}) 2139, 2104, 1616, 1332, 1280, 1166, 1122, 1070, 911. HR-MS (EI) m/z calcd for $C_9H_6N_3F_3$ [M^+] 213.05083, found 213.05060.

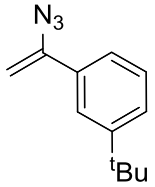

1-(1-Azidovinyl)-3-(*tert*-butyl)benzene (**S3**). Colorless oil. This compound was synthesized according to the procedure described by Chiba *et al.* modified from Hassner's method.[2,3] ^{1}H NMR (300 MHz, $CDCl_3$): δ 7.59 (s, 1H), 7.43 –7.36 (m, 2H), 7.34 –7.27 (m, 1H), 5.42 (d, *J* = 2.2 Hz, 1H), 4.96 (d, *J* = 2.2 Hz, 1H), 1.35 (s, 9H). ^{13}C NMR (75 MHz, $CDCl_3$): δ 151.54, 145.69, 134.16, 128.33, 126.37, 123.04, 122.73, 98.03, 34.90, 31.44. IR (film): ν (cm^{-1}) 2963, 2106, 1595, 1483, 1300, 1262, 836, 796. HR-MS (ESI) m/z calcd for $C_{12}H_{16}N_3$ [$M+H^+$] 202.13387, found 202.13405.

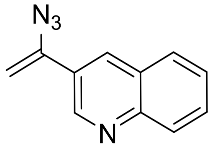

3-(1-azidovinyl)quinoline (**S4**). White solid. This compound was synthesized according to the procedure described by Bi *et al.*[1] ^{1}H NMR (300 MHz, $CDCl_3$): δ 9.12 (d, *J* = 2.2 Hz, 1H), 8.29 (d, *J* = 2.2 Hz, 1H), 8.10 (d, *J* = 8.4 Hz, 1H), 7.84 (dd, *J* = 8.1, 1.3 Hz, 1H), 7.73 (ddd, *J* = 8.4, 7.0, 1.3 Hz, 1H), 7.57 (dd, *J* = 8.1, 7.0 Hz, 1H), 5.69 (d, *J* = 2.9 Hz, 1H), 5.15 (d, *J* = 2.9 Hz, 1H). ^{13}C NMR (75 MHz, $CDCl_3$): δ 148.20, 147.91, 142.93, 132.53, 130.22, 129.40, 128.48, 127.46, 127.37, 127.21, 99.34. IR (film): ν (cm^{-1}) 2970, 1738, 1569, 1496, 1366, 1270, 1228, 1217. HR-MS (ESI) m/z calcd for $C_{11}H_9N_4$ [$M+H^+$] 197.08217, found 197.08241.

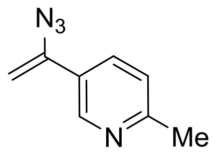

3-(1-azidovinyl)-6-methylpyridine (**S5**). Colorless oil. This compound was synthesized according to the procedure described by Bi *et al.*[1] ^{1}H NMR (300 MHz, $CDCl_3$): δ 8.66 (d, *J* = 2.2 Hz, 1H), 7.67 (dd, *J* = 8.1, 2.2 Hz, 1H), 7.09 (d, *J* = 8.1 Hz, 1H), 5.41 (d, *J* = 2.6 Hz, 1H), 4.95 (d, *J* = 2.6 Hz, 1H), 2.52 (s, 3H). ^{13}C NMR (75 MHz, $CDCl_3$): δ 159.19, 146.37, 142.82, 133.22, 127.31, 122.76, 98.31, 24.29. IR (film): ν (cm^{-1}) 2970, 2134, 2100, 1738, 1610, 1490, 1373, 1293, 1216, 1023. HR-MS (ESI) m/z calcd for $C_8H_9N_4$ [$M+H^+$] 161.08217, found 161.08187.

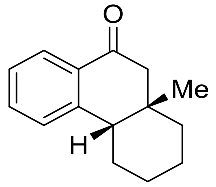

10a-Methyl-2,3,4,4a,10,10a-hexahydrophenanthren-9(1*H*)-one (**1a**). Colorless oil. ^{1}H NMR (400 MHz, $CDCl_3$): δ 7.99 (ddd, *J* = 7.6, 1.1, 0.4 Hz, 1H), 7.48 (td, *J* = 7.6, 1.5 Hz, 1H), 7.28 (td, *J* = 7.6, 1.1 Hz, 1H), 7.24 – 7.21 (m, 1H), 3.03 (d, *J* = 17.3 Hz, 1H), 2.53 (dd, *J* = 11.4, 3.9 Hz, 1H), 2.12 (dd, *J* = 17.3, 1.3 Hz, 1H), 1.85 – 1.75 (m, 2H), 1.68 – 1.61 (m, 1H), 1.58 – 1.33 (m, 5H), 0.94 (d, *J* = 1.0 Hz, 3H). ^{13}C NMR (100 MHz, $CDCl_3$): δ 199.26, 148.19, 134.01, 130.81, 129.29, 126.83, 126.52, 47.92, 44.53, 39.21, 35.14, 33.09, 28.89, 26.11, 21.92. IR (film): ν (cm^{-1}) 2969, 2926, 1736, 1680, 1449, 1366, 1228, 1215. HR-MS (ESI) m/z calcd for $C_{15}H_{19}O$ [$M+H^+$] 215.14304, found 215.14305.

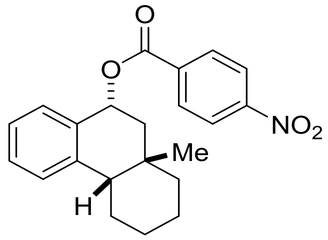

1a`

Methyl-1,2,3,4,4a,9,10,10a-octahydrophenanthren-9-yl 4-nitrobenzoate: To a Schlenk-tube were added **1a** (214 mg, 1.0 mmol), $NaBH_4$ (46 mg, 1.2 mmol) and MeOH (10 mL) under nitrogen, the resulting mixture was stirred at room temperature for 12 h. The reaction was then quenched by water and the organic solvent was removed under vacuum. The residue was extracted with ethyl acetate (200 mL). The organic layer was washed with water (50 mL), brine (50 mL), and dried over $MgSO_4$. Filtration and evaporation gave a viscous oil, which was directly used for the next step.To a Schlenk-tube were added the residue of the previous step, DCM (100 mL) and Et_3N (202 mg, 2.0 mmol). The reaction was cooled to 0 °C, and 4-nitrobenzoyl chloride (223 mg, 1.2 mmol) was added dropwise. The resulting mixture was warmed to room temperature and stirred for 15 h. The reaction was quenched by water, and extracted with DCM (200 mL). The organic layer was washed with diluted HCl aqueous solution (1 M, 50 mL), and brine (50 mL), dried over anhydrous $MgSO_4$, filtered and concentrated under reduced pressure. The crude product was purified by column chromatography on silica gel (hexane:ethyl acetate = 5:1) to give **1a`** (189 mg, 52%). ^{1}H NMR (300 MHz, $CDCl_3$): δ 8.23 – 8.16 (m, 4H), 7.21 – 7.16 (m, 2H), 7.14 – 7.10 (m, 1H), 7.08 – 7.05 (m, 1H), 6.29 (dd, *J* = 7.5, 5.7 Hz, 1H), 2.31 (dd, *J* = 8.7, 2.4 Hz, 1H), 2.21 (dd, *J* = 9.6, 7.5 Hz, 1H), 1.83 (ddd, *J* = 9.6, 5.7, 1.2 Hz, 1H), 1.80 – 1.75 (m, 2H), 1.57 –

1.25 (m, 6H), 0.95 (s, 3H). ^{13}C NMR (75 MHz, $CDCl_3$): δ 164.88, 150.59, 142.87, 135.98, 132.26, 130.88, 129.49, 128.19, 127.52, 126.19, 123.55, 73.09, 46.96, 40.59, 34.05, 33.82, 33.76, 27.95, 26.66, 21.63.

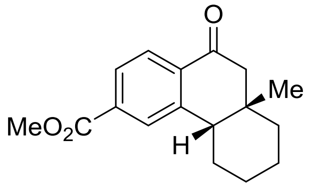

Methyl 8a-methyl-10-oxo-4b,5,6,7,8,8a,9,10-octahydrophenanthrene-3-carboxylate (**1b**). Yellow solid. ^{1}H NMR (400 MHz, $CDCl_3$): δ 8.04 (d, *J* = 8.0 Hz, 1H), 7.95 – 7.90 (m, 2H), 3.93 (s, 3H), 3.05 (d, *J* = 17.3 Hz, 1H), 2.61 (dd, *J* = 11.0, 3.9 Hz, 1H), 2.16 (dd, *J* = 17.3, 1.3 Hz, 1H), 1.89 – 1.76 (m, 2H), 1.73 – 1.63 (m, 1H), 1.62 – 1.52 (m, 1H), 1.52 – 1.34 (m, 4H), 0.94 (d, *J* = 0.7 Hz, 3H). ^{13}C NMR (100 MHz, $CDCl_3$): δ 198.81, 166.54, 148.11, 134.69, 133.83, 130.85, 127.42, 127.03, 52.53, 47.90, 44.57, 39.12, 35.10, 33.01, 28.84, 26.05, 21.87. IR (film): ν (cm^{-1}) 2970, 2945, 1736, 1438, 1366, 1228, 1215. HR-MS (ESI) m/z calcd for $C_{17}H_{21}O_3$ [$M+H^+$] 273.14852, found 273.14862.

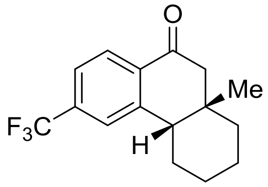

10a-Methyl-6-(trifluoromethyl)-2,3,4,4a,10,10a-hexahydrophenanthren-9(1*H*)-one (**1c**). Colorless oil. ^{1}H NMR (400 MHz, $CDCl_3$): δ 8.10 (d, *J* = 8.0 Hz, 1H), 7.56 – 7.51 (m, 2H), 3.06 (d, *J* = 17.4 Hz, 1H), 2.61 (dd, *J* = 11.3, 4.1 Hz, 1H), 2.18 (dd, *J* = 17.4, 1.3 Hz, 1H), 1.89 – 1.78 (m, 2H), 1.71 – 1.64 (m, 1H), 1.61 – 1.54 (m, 1H), 1.52 – 1.38 (m, 4H), 0.95 (d, *J* = 0.9 Hz, 3H). ^{13}C NMR (100 MHz, $CDCl_3$): δ 198.27, 148.67, 135.19 (q, J_{C2-F} = 32.3 Hz), 133.28, 127.59, 126.41 (q, J_{C3-F} = 3.8 Hz), 123.79 (q, J_{C1-F} = 272.9 Hz), 123.38 (q, J_{C3-F} = 3.7 Hz), 47.96, 44.45, 39.09, 35.10, 33.07, 28.82, 26.02, 21.82. ^{19}F NMR (376 MHz, $CDCl_3$): δ -63.16 (s, 3F). IR (film): ν (cm^{-1}) 2970, 1718, 1435, 1374, 1216, 1122. HR-MS (EI) m/z calcd for $C_{16}H_{17}OF_3$ [M^+] 282.12260, found 282.12254.

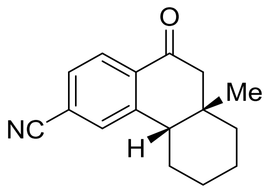

8a-Methyl-10-oxo-4b,5,6,7,8,8a,9,10-octahydrophenanthrene-3-carbonitrile (**1d**). Colorless solid. ^{1}H NMR (400 MHz, $CDCl_3$): δ 8.07 (d, *J* = 8.4 Hz, 1H), 7.60 – 7.53 (m, 2H), 3.05 (d, *J* = 17.4 Hz, 1H), 2.62 – 2.53 (m, 1H), 2.19 (d, *J* = 17.4 Hz, 1H), 1.87 – 1.78 (m, 2H), 1.73 – 1.65 (m, 1H), 1.62 – 1.54 (m, 1H), 1.52 – 1.35 (m, 4H), 0.94 (s, 3H). ^{13}C NMR (100 MHz, $CDCl_3$): δ 197.89, 148.71, 133.63, 133.45, 129.97, 127.61, 118.25, 117.16, 47.62, 44.39, 38.96, 35.00, 33.01, 28.76, 25.93, 21.74. IR (film): ν (cm^{-1}) 2915, 2852, 2226, 1684, 1606, 1449, 1408, 1276. HR-MS (ESI) m/z calcd for $C_{16}H_{18}ON$ [$M+H^+$] 240.13829, found 240.13830.

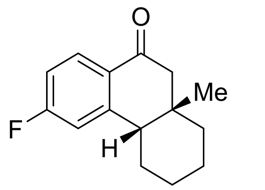

6-Ffluoro-10a-methyl-2,3,4,4a,10,10a-hexahydrophenanthren-9(1*H*)-one (**1e**). Colorless oil. ^{1}H NMR (400 MHz, CDCl$_3$): δ 8.03 (dd, *J* = 9.0 Hz, $J_{H\text{-}F}$ = 6.0 Hz, 1H), 6.96 (ddd, *J* = 9.0, 2.5 Hz, $J_{H\text{-}F}$ = 9.0 Hz, 1H), 6.91 (dd, *J* = 2.5 Hz, $J_{H\text{-}F}$ = 9.0 Hz, 1H), 3.01 (d, *J* = 17.3 Hz, 1H), 2.52 (dd, *J* = 11.6, 3.4 Hz, 1H), 2.12 (dd, *J* = 17.3, 1.3 Hz, 1H), 1.86 – 1.76 (m, 2H), 1.70 – 1.63 (m, 1H), 1.59 – 1.51 (m, 1H), 1.51 – 1.45 (m, 1H), 1.45 – 1.33 (m, 3H), 0.95 (d, *J* = 0.9 Hz, 3H). ^{13}C NMR (100 MHz, CDCl$_3$): δ 197.76, 166.37 (d, $J_{C1\text{-}F}$ = 255.3 Hz), 151.40 (d, $J_{C3\text{-}F}$ = 8.5 Hz), 130.06 (d, $J_{C3\text{-}F}$ = 9.8 Hz), 127.54 (d, $J_{C4\text{-}F}$ = 2.5 Hz), 115.55 (d, $J_{C2\text{-}F}$ = 21.0 Hz), 114.23 (d, $J_{C2\text{-}F}$ = 22.0 Hz), 48.18, 44.37, 39.03, 35.28, 32.88, 28.87, 26.00, 21.85. ^{19}F NMR (376 MHz, CDCl$_3$): δ -104.45 (td, *J* = 9.0, 6.0 Hz, 1F). IR (film): ν (cm^{-1}) 2970, 1738, 1436, 1366, 1228, 1216. HR-MS (ESI) m/z calcd for $C_{15}H_{18}OF$ [M+H$^+$] 233.13362, found 233.13367.

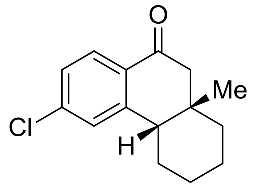

6-Chloro-10a-methyl-2,3,4,4a,10,10a-hexahydrophenanthren-9(1*H*)-one (**1f**). Colorless oil. ^{1}H NMR (400 MHz, CDCl$_3$): δ 7.94 (d, *J* = 8.3 Hz, 1H), 7.28 – 7.22 (m, 2H), 3.01 (d, *J* = 17.4 Hz, 1H), 2.50 (dd, *J* = 11.4, 3.4 Hz, 1H), 2.12 (dd, *J* = 17.4, 1.3 Hz, 1H), 1.85 – 1.77 (m, 2H), 1.70 – 1.62 (m, 1H), 1.59 – 1.52 (m, 1H), 1.52 – 1.32 (m, 4H), 0.95 (d, *J* = 0.8 Hz, 3H). ^{13}C NMR (100 MHz, CDCl$_3$): δ 198.17, 149.88, 140.20, 129.33, 129.22, 128.66, 127.12, 47.92, 44.38, 39.09, 35.23, 32.96, 28.88, 26.03, 21.85. IR (film): ν (cm^{-1}) 2970, 1740, 1445, 1366, 1228, 1215. HR-MS (ESI) m/z calcd for $C_{15}H_{18}OCl$ [M+H$^+$] 249.10407, found 249.10408.

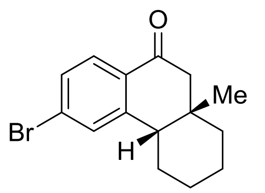

6-Bromo-10a-methyl-2,3,4,4a,10,10a-hexahydrophenanthren-9(1*H*)-one (**1g**). Colorless oil. ^{1}H NMR (400 MHz, CDCl$_3$): δ 7.86 (dd, *J* = 7.8, 1.2 Hz, 1H), 7.40 - 7.44 (m 2H), 3.01 (d, *J* = 17.4 Hz, 1H), 2.50 (dd, *J* = 11.6, 3.7 Hz, 1H), 2.12 (dd, *J* = 17.4, 1.3 Hz, 1H), 1.85 – 1.77 (m, 2H), 1.70- 1.62 (m, 1H), 1.58 – 1.51 (m, 1H), 1.51 – 1.34 (m, 4H), 0.93 (d, *J* = 1.0 Hz, 3H). ^{13}C NMR (100 MHz, CDCl$_3$): δ 198.35, 149.97, 132.24, 130.07, 129.71, 129.09, 128.71, 47.86, 44.36, 39.10, 35.22, 32.98, 28.86, 26.03, 21.83. IR (film): ν (cm^{-1}) 2971, 1684, 1585, 1406, 1260, 1076. HR-MS (ESI) m/z calcd for $C_{15}H_{18}OBr$ [M+H$^+$] 293.05355, found 293.05347.

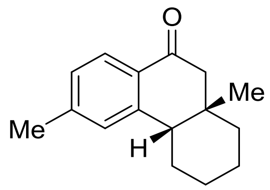

6,10a-Dimethyl-2,3,4,4a,10,10a-hexahydrophenanthren-9(1*H*)-one (**1h**). Colorless solid. ^{1}H NMR (400 MHz, $CDCl_3$): δ 7.90 (d, *J* = 8.0 Hz, 1H), 7.09 (dd, *J* = 8.0, 1.0 Hz, 1H), 7.05 – 7.02 (m, 1H), 3.01 (d, *J* = 17.3 Hz, 1H), 2.47 (dd, *J* = 11.2, 3.6 Hz, 1H), 2.37 (s, 3H), 2.09 (dd, *J* = 17.3, 1.3 Hz, 1H), 1.84 – 1.75 (m, 2H), 1.70 – 1.61 (m, 1H), 1.57 – 1.34 (m, 5H), 0.94 (d, *J* = 0.9 Hz, 3H). ^{13}C NMR (100 MHz, $CDCl_3$): δ 199.02, 148.34, 144.82, 129.76, 128.56, 127.59, 127.00, 48.00, 44.50, 39.26, 35.19, 33.12, 28.97, 26.17, 21.96, 21.90. IR (film): ν (cm^{-1}) 2970, 1718, 1436, 1364, 1228. HR-MS (ESI) m/z calcd for $C_{16}H_{21}O$ [M+H$^+$] 229.15869, found 229.15866.

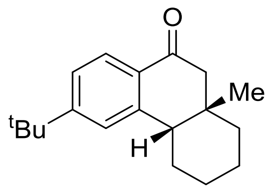

6-(*tert*-Butyl)-10a-methyl-2,3,4,4a,10,10a-hexahydrophenanthren-9(1*H*)-one (**1i**). Colorless oil. ^{1}H NMR (400 MHz, $CDCl_3$): δ 7.92 (d, *J* = 8.3 Hz, 1H), 7.31 (dd, *J* = 8.3, 2.0 Hz, 1H), 7.21 (d, *J* = 2.0 Hz, 1H), 3.02 (d, *J* = 17.3 Hz, 1H), 2.51 (dd, *J* = 11.4, 3.5 Hz, 1H), 2.09 (dd, *J* = 17.3, 1.3 Hz, 1H), 1.85 – 1.77 (m, 2H), 1.70 – 1.60 (m, 1H), 1.58 – 1.36 (m, 5H), 1.33 (s, 9H), 0.95 (d, *J* = 0.9 Hz, 3H). ^{13}C NMR (100 MHz, $CDCl_3$): δ 199.11, 157.75, 148.12, 128.46, 126.67, 125.88, 123.91, 48.31, 44.54, 39.37, 35.29, 35.27, 33.35, 31.24, 29.03, 26.23, 22.01. IR (film): ν (cm^{-1}) 2970, 1716, 1436, 1366, 1216. HR-MS (ESI) m/z calcd for $C_{19}H_{27}O$ [M+H$^+$] 271.20564, found 271.20597.

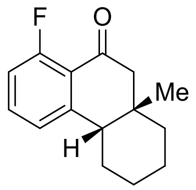

8-Fluoro-10a-methyl-2,3,4,4a,10,10a-hexahydrophenanthren-9(1*H*)-one (**1j**). Colorless oil. ^{1}H NMR (400 MHz, $CDCl_3$): δ 7.43 (ddd, *J* = 8.2, 7.7 Hz, J_{H-F} = 5.1 Hz, 1H), 7.02 (ddd, *J* = 7.7, 0.8 Hz, J_{H-F} = 0.8 Hz, 1H), 6.95 (ddd, *J* = 8.2, 0.8 Hz, J_{H-F} = 11.3 Hz, 1H), 3.04 (d, *J* = 17.2 Hz, 1H), 2.55 (dd, *J* = 11.8, 4.0 Hz, 1H), 2.11 (dd, *J* = 17.2, 1.4 Hz, 1H), 1.84 – 1.73 (m, 2H), 1.68 – 1.61 (m, 1H), 1.57 – 1.50 (m, 1H), 1.50 – 1.31 (m, 4H), 0.97 (d, *J* = 0.8 Hz, 3H). ^{13}C NMR (100 MHz, $CDCl_3$): δ 197.13, 161.96 (d, J_{C1-F} = 265.6 Hz), 150.82, 134.96 (d, J_{C2-F} = 10.4 Hz), 125.06 (d, J_{C3-F} = 3.9 Hz), 119.74 (d, J_{C3-F} = 4.7 Hz), 114.96 (d, J_{C2-F} = 22.1 Hz), 48.32 (d, J_{C4-F} = 2.2 Hz), 45.64, 38.98, 34.84, 33.20, 28.87, 26.07, 21.79. ^{19}F NMR (376 MHz, $CDCl_3$): δ -112.13 (dd, *J* = 11.3, 5.1 Hz, 1F). IR (film): ν (cm^{-1}) 2970, 1738, 1449, 1366, 1228, 1216. HR-MS (ESI) m/z calcd for $C_{15}H_{18}OF$ [M+H$^+$] 233.13362, found 233.13352.

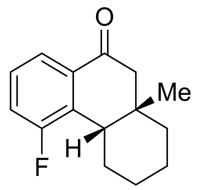

5-Fluoro-10a-methyl-2,3,4,4a,10,10a-hexahydrophenanthren-9(1*H*)-one (1k) (Major regioisomer). Colorless oil. ^{1}H NMR (400 MHz, $CDCl_3$): δ 7.82 (dd, *J* = 7.1, 2.0 Hz, 1H), 7.30 – 7.18 (m, 2H), 3.06 (d, *J* = 17.3 Hz, 1H), 2.88 (dd, *J* = 11.5, 4.2 Hz, 1H), 2.12 (dd, *J* = 17.3, 1.4 Hz, 1H), 1.98 – 1.91 (m, 1H), 1.89 – 1.76 (m, 1H), 1.70 – 1.63 (m, 1H), 1.60 – 1.54 (m, 1H), 1.54 – 1.32 (m, 4H), 0.94 (d, *J* = 1.0 Hz, 3H). ^{13}C NMR (100 MHz, $CDCl_3$): δ 198.34 (d, $J_{C4\text{-}F}$ = 3.1 Hz), 160.78 (d, $J_{C1\text{-}F}$ = 246.0 Hz), 134.74 (d, $J_{C2\text{-}F}$ = 16.5 Hz), 132.80 (d, $J_{C3\text{-}F}$ = 3.8 Hz), 127.26 (d, $J_{C3\text{-}F}$ = 8.2 Hz), 122.63 (d, $J_{C4\text{-}F}$ = 3.3 Hz), 120.57 (d, $J_{C2\text{-}F}$ = 22.4 Hz), 44.18, 40.74 (d, $J_{C3\text{-}F}$ = 1.2 Hz), 39.54, 34.82, 30.46, 28.83, 25.99, 21.94. ^{19}F NMR (376 MHz, $CDCl_3$): δ -120.59 (dd, *J* = 8.9, 5.7 Hz, 1F). IR (film): ν (cm^{-1}) 2927, 1686, 1609, 1455, 1295, 1278, 1237, 791. HR-MS (ESI) m/z calcd for $C_{15}H_{18}OF$ [M+H$^+$] 233.13362, found 233.13351.

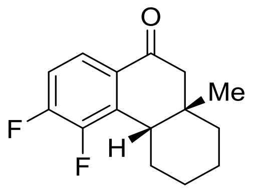

5,6-Difluoro-10a-methyl-2,3,4,4a,10,10a-hexahydrophenanthren-9(1*H*)-one (1l) (Major regioisomer). Colorless oil. ^{1}H NMR (400 MHz, $CDCl_3$): δ 7.83 (ddd, *J* = 8.8, 5.1, 1.7 Hz, 1H), 7.09 (ddd, *J* = 9.5, 8.8, 7.4 Hz, 1H), 3.04 (d, *J* = 17.4 Hz, 1H), 2.94 – 2.90 (m, 1H), 2.12 (dd, *J* = 17.4, 1.4 Hz, 1H), 2.00 – 1.91 (m, 1H), 1.90 – 1.75 (m, 1H), 1.71 – 1.63 (m, 1H), 1.63 – 1.53 (m, 1H), 1.52 – 1.36 (m, 4H), 0.94 (d, *J* = 0.9 Hz, 3H). ^{13}C NMR (100 MHz, $CDCl_3$): δ 197.03, 154.22 (dd, $J_{C1\text{-}F}$ = 257.0 Hz, $J_{C2\text{-}F}$ = 13.4 Hz), 148.19 (dd, $J_{C1\text{-}F}$ = 247.6 Hz, $J_{C2\text{-}F}$ = 12.7 Hz), 127.97 (dd, $J_{C3\text{-}F}$ = 2.9 Hz, $J_{C4\text{-}F}$ = 2.5 Hz), 123.79 (dd, $J_{C3\text{-}F}$ = 7.9 Hz, $J_{C4\text{-}F}$ = 4.0 Hz), 117.64 (d, $J_{C2\text{-}F}$ = 17.2 Hz), 115.22 (d, $J_{C2\text{-}F}$ = 18.0 Hz), 43.90, 41.11, 39.38, 34.97, 30.41, 28.80, 25.90, 21.83. ^{19}F NMR (376 MHz, $CDCl_3$): δ -128.91 (ddd, *J* = 20.6, 9.5, 5.1 Hz, 1F), -144.45 (dd, *J* = 20.6, 7.4 Hz, 1F). IR (film): ν (cm^{-1}) 2970, 1738, 1446, 1366, 1228, 1215. HR-MS (ESI) m/z calcd for $C_{15}H_{17}OF_2$ [M+H$^+$] 251.12420, found 251.12418.

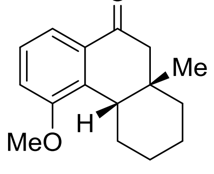

5-Methoxy-10a-methyl-2,3,4,4a,10,10a-hexahydrophenanthren-9(1*H*)-one (1m) (Major regioisomer). Colorless oil. ^{1}H NMR (400 MHz, $CDCl_3$): δ 7.64 (dd, *J* = 8.0, 1.1 Hz, 1H), 7.24 (t, *J* = 8.0 Hz, 1H), 7.04 (dd, *J* = 8.0, 1.1 Hz, 1H), 3.86 (s, 3H), 3.06 (d, *J* = 17.2 Hz, 1H), 2.92 (ddd, *J* = 12.0, 4.1, 1.3 Hz, 1H), 2.08 (dd, *J* = 17.2, 1.3 Hz, 1H), 1.99 – 1.90 (m, 1H), 1.81 – 1.75 (m, 1H), 1.69 – 1.60 (m, 1H), 1.58 – 1.37 (m, 4H), 1.27 – 1.20 (m, 1H), 0.91 (d, *J* = 1.0 Hz, 3H). ^{13}C NMR (100 MHz, $CDCl_3$): δ 199.73, 157.18, 136.73, 132.00, 126.70, 118.76, 115.22, 55.82, 44.23, 41.09, 39.78, 34.85, 29.57, 28.92, 26.19, 22.15. IR (film): ν (cm^{-1}) 2909, 1738, 1438, 1365, 1228, 1217. HR-MS (ESI) m/z calcd for $C_{16}H_{21}O_2$ [M+H$^+$] 245.15361, found 245.15369.

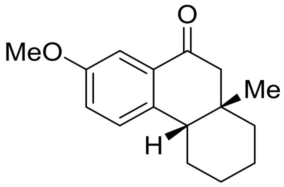

7-Methoxy-10a-methyl-2,3,4,4a,10,10a-hexahydrophenanthren-9(1*H*)-one (**1m**) (Minor regioisomer). Colorless oil. ^{1}H NMR (400 MHz, $CDCl_3$): δ 7.49 (d, *J* = 2.8 Hz, 1H), 7.15 (d, *J* = 8.4 Hz, 1H), 7.08 (dd, *J* = 8.4, 2.8 Hz, 1H), 3.84 (s, 3H), 3.02 (d, *J* = 17.3 Hz, 1H), 2.49 (dd, *J* = 11.1, 4.1 Hz, 1H), 2.12 (dd, *J* = 17.3, 1.3 Hz, 1H), 1.85 – 1.74 (m, 2H), 1.68 – 1.62 (m, 1H), 1.60 – 1.51 (m, 1H), 1.50 – 1.33 (m, 4H), 0.95 (d, *J* = 0.9 Hz, 3H). ^{13}C NMR (100 MHz, $CDCl_3$): δ 199.34, 158.33, 140.94, 131.68, 130.52, 122.39, 108.87, 55.64, 47.18, 44.50, 39.29, 35.37, 33.12, 28.90, 26.07, 22.01. IR (film): ν (cm^{-1}) 2909, 1738, 1438, 1365, 1228, 1217. HR-MS (ESI) m/z calcd for $C_{16}H_{21}O_2$ [$M+H^+$] 245.15361, found 245.15364.

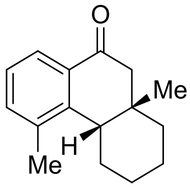

5,10a-Dimethyl-2,3,4,4a,10,10a-hexahydrophenanthren-9(1*H*)-one (**1n**) (Major regioisomer). Colorless oil.^{1}H NMR (400 MHz, $CDCl_3$): δ 7.93 – 7.89 (m, 1H), 7.38 – 7.34 (m, 1H), 7.19 (t, *J* = 7.6 Hz, 1H), 3.08 (d, *J* = 17.7 Hz, 1H), 2.67 (dd, *J* = 11.5, 3.3 Hz, 1H), 2.37 (s, 3H), 2.11 (dd, *J* = 17.7, 1.3 Hz, 1H), 1.87 – 1.76 (m, 2H), 1.72 – 1.64 (m, 1H), 1.60 – 1.48 (m, 2H), 1.47 – 1.25 (m, 3H), 0.91 (d, *J* = 0.9 Hz, 3H). ^{13}C NMR (100 MHz, $CDCl_3$): δ 199.83, 146.10, 136.16, 136.05, 131.13, 126.09, 125.24, 44.67, 43.87, 39.58, 34.97, 29.96, 29.15, 26.29, 21.99, 18.89. IR (film): ν (cm^{-1}) 2970, 1738, 1436, 1366, 1228, 1217. HR-MS (ESI) m/z calcd for $C_{16}H_{21}O$ [$M+H^+$] 229.15869, found 229.15880.

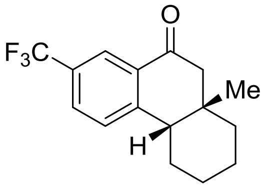

10a-Methyl-7-(trifluoromethyl)-2,3,4,4a,10,10a-hexahydrophenanthren-9(1*H*)-one (**1o**) (Major regioisomer). Colorless oil. ^{1}H NMR (400 MHz, $CDCl_3$): δ 8.29 – 8.26 (m, 1H), 7.73 (dd, *J* = 8.0, 2.0 Hz, 1H), 7.38 (d, *J* = 8.0 Hz, 1H), 3.06 (d, *J* = 17.4 Hz, 1H), 2.65 – 2.57 (m, 1H), 2.19 (dd, *J* = 17.4, 1.3 Hz, 1H), 1.89 – 1.78 (m, 2H), 1.74 – 1.65 (m, 1H), 1.62 – 1.53 (m, 1H), 1.52 – 1.36 (m, 4H), 0.95 (d, *J* = 0.9 Hz, 3H). ^{13}C NMR (100 MHz, $CDCl_3$): δ 197.97, 151.70, 131.15, 130.20 (q, J_{C3-F} = 3.8 Hz), 130.16, 129.30 (q, J_{C2-F} = 33.0 Hz), 124.22 (q, J_{C3-F} = 3.9 Hz), 123.98 (q, J_{C1-F} = 272.2 Hz), 47.95, 44.34, 39.06, 35.07, 32.95, 28.82, 26.05, 21.83. ^{19}F NMR (376 MHz, $CDCl_3$): δ -62.67 (s, 3F). IR (film): ν (cm^{-1}) 2930, 1691, 1334, 1252, 1126. HR-MS (ESI) m/z calcd for $C_{16}H_{18}OF_3$ [$M+H^+$] 283.13043, found 283.13040.

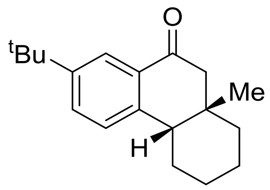

7-(*tert*-Butyl)-10a-methyl-2,3,4,4a,10,10a-hexahydrophenanthren-9(1*H*)-one (**1p**). Colorless oil. ^{1}H NMR (400 MHz, $CDCl_3$): δ 8.02 (d, *J* = 2.2 Hz, 1H), 7.54 (dd, *J* = 8.0, 2.2 Hz, 1H), 7.17 (d, *J* = 8.0 Hz, 1H), 3.02 (d, *J* = 17.2 Hz, 1H), 2.52 (dd, *J* = 11.7, 3.9 Hz, 1H), 2.12 (dd, *J* = 17.2, 1.2 Hz, 1H), 1.86 – 1.76 (m, 2H), 1.69 – 1.61 (m, 1H), 1.59 – 1.35 (m, 5H), 1.33 (s, 9H), 0.95 (s, 3H). ^{13}C NMR (100 MHz, $CDCl_3$): δ 199.78, 149.51, 145.40, 131.46, 130.42, 129.05, 123.32, 47.48, 44.77, 39.28, 35.26, 34.77, 33.04, 31.41, 29.00, 26.13, 22.02. IR (film): ν (cm^{-1}) 2925, 1682, 1448, 1363, 1240, 824. HR-MS (ESI) m/z calcd for $C_{19}H_{27}O$ [M+H$^+$] 271.20564, found 271.20546.

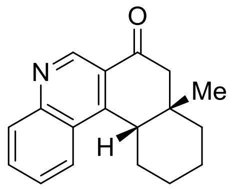

8a-Methyl-8a,9,10,11,12,12a-hexahydrobenzo[*k*]phenanthridin-7(8*H*)-one (**1q**). Colorless oil. ^{1}H NMR (300 MHz, $CDCl_3$): δ 9.44 (s, 1H), 8.14 (m, 2H), 7.81 (ddd, *J* = 8.4, 7.0, 1.3 Hz, 1H), 7.64 (ddd, *J* = 8.2, 7.0, 1.2 Hz, 1H), 3.36 – 3.25 (m, 1H), 3.15 (d, *J* = 17.4 Hz, 1H), 2.24 (dd, *J* = 17.4, 0.9 Hz, 1H), 2.11 – 1.99 (m, 1H), 1.96 – 1.85 (m, 1H), 1.80 – 1.73 (m, 1H), 1.73 – 1.45 (m, 5H), 0.98 (s, 3H). ^{13}C NMR (75 MHz, $CDCl_3$): δ 198.54, 154.82, 150.32, 148.69, 131.65, 130.91, 127.44, 125.76, 124.60, 122.57, 43.95, 43.38, 39.45, 35.41, 31.37, 28.85, 26.20, 21.98. IR (film): ν (cm^{-1}) 2970, 1738, 1436, 1366, 1229. HR-MS (ESI) m/z calcd for $C_{18}H_{20}ON$ [M+H$^+$] 266.15394, found 266.15441.

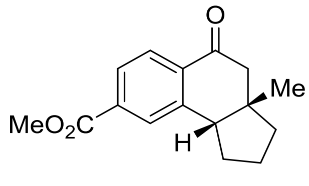

Methyl-3a-methyl-5-oxo-2,3,3a,4,5,9b-hexahydro-1*H*-cyclopenta[*a*]naphthalene-8-carboxylate (**2a**). Colorless oil. ^{1}H NMR (400 MHz, $CDCl_3$): δ 8.03 (d, *J* = 8.1 Hz, 1H), 7.94 – 7.93 (m, 1H), 7.91 (dd, *J* = 8.1, 1.6 Hz, 1H), 3.93 (s, 3H), 2.94 (t, *J* = 9.0 Hz, 1H), 2.71 (dd, *J* = 16.3, 0.6 Hz, 1H), 2.37 (dd, *J* = 16.3, 1.3 Hz, 1H), 2.38 – 2.30 (m, 1H), 1.91 – 1.79 (m, 3H), 1.72 – 1.67 (m, 2H), 1.10 (d, *J* = 0.6 Hz, 3H). ^{13}C NMR (100 MHz, $CDCl_3$): δ 198.55, 166.57, 145.77, 134.53, 133.64, 131.35, 127.37, 127.05, 52.55, 50.11, 46.47, 44.58, 40.27, 34.23, 25.76, 22.52. IR (film): ν (cm^{-1}) 2970, 1716, 1435, 1368, 1216. HR-MS (ESI) m/z calcd for $C_{16}H_{19}O_3$ [M+H$^+$] 259.13287, found 259.13288.

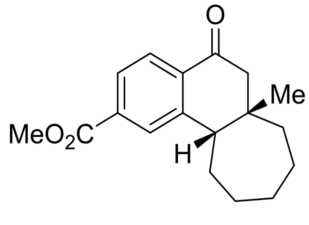

Methyl-6a-methyl-5-oxo-6,6a,7,8,9,10,11,11a-octahydro-5*H*-cyclohepta[*a*]naphthalene-2-carboxylate (**2b**). Colorless oil. ^{1}H NMR (400 MHz, $CDCl_3$): δ 7.98 (d, *J* = 8.1 Hz, 1H), 7.96 (d, *J* = 1.6 Hz, 1H), 7.87 (dd, *J* = 8.1, 1.6 Hz, 1H), 3.93 (s, 3H), 2.83 (d, *J* = 9.7 Hz, 1H), 2.75 (d, *J* = 16.5 Hz, 1H), 2.32 (dd, *J* = 16.5, 1.3 Hz, 1H), 2.04 – 1.94 (m, 1H), 1.94 – 1.85 (m, 2H), 1.77 – 1.61 (m, 4H), 1.56 – 1.44 (m, 2H), 1.41 – 1.29 (m, 1H), 0.97 (s, 3H). ^{13}C NMR (100 MHz, $CDCl_3$): δ 198.00, 166.55, 149.31, 134.78, 133.63, 131.85, 127.15, 126.35, 52.53, 50.95, 48.48, 42.37, 37.85, 33.41, 30.57, 30.40, 27.34, 21.42. IR (film): ν (cm^{-1}) 2970, 1716, 1436, 1373, 1228. HR-MS (ESI) m/z calcd for $C_{18}H_{23}O_3$ [M+H$^+$] 287.16417, found 287.16418.

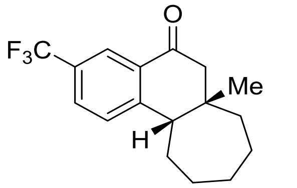

6a-Methyl-3-(trifluoromethyl)-6,6a,7,8,9,10,11,11a-octahydro-5*H*-cyclohepta[*a*]naphthalen-5-one (**2c**). Colorless oil. ^{1}H NMR (400 MHz, $CDCl_3$): δ 8.23 (s, 1H), 7.72 (dd, *J* = 8.2, 1.7 Hz, 1H), 7.42 (d, *J* = 8.2 Hz, 1H), 2.83 (d, *J* = 9.6 Hz, 1H), 2.76 (d, *J* = 16.6 Hz, 1H), 2.35 (dd, *J* = 16.6, 1.3 Hz, 1H), 2.09 – 1.85 (m, 3H), 1.77 – 1.63 (m, 3H), 1.59 – 1.32 (m, 4H), 0.99 (s, 3H). ^{13}C NMR (100 MHz, $CDCl_3$): δ 197.20, 152.90, 131.12, 131.00, 130.19 (q, J_{C3-F} = 3.4 Hz), 129.14 (q, J_{C2-F} = 33.1 Hz), 123.96 (q, J_{C1-F} = 272.4 Hz), 123.60 (q, J_{C3-F} = 3.9 Hz), 51.05, 48.28, 42.32, 37.84, 33.38, 30.59, 30.34, 27.38, 21.41. ^{19}F NMR (376 MHz, $CDCl_3$): δ -62.73 (s, 3F). IR (film): ν (cm^{-1}) 2928, 1693, 1619, 1334, 1260, 1164, 1123, 1073. HR-MS (ESI) m/z calcd for $C_{17}H_{20}OF_3$ [M+H$^+$] 297.14608, found 297.14606.

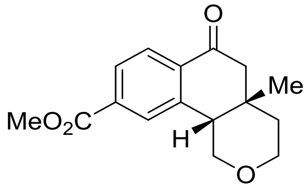

Methyl-4a-methyl-6-oxo-3,4,4a,5,6,10b-hexahydro-1*H*-benzo[*h*]isochromene-9-carboxylate (**2d**). Colorless solid. ^{1}H NMR (400 MHz, $CDCl_3$): δ 8.09 (d, *J* = 8.1 Hz, 1H), 7.98 (dd, *J* = 8.1, 1.6 Hz, 1H), 7.97 – 7.95 (m, 1H), 3.95 (s, 3H), 3.97 – 3.89 (m, 2H), 3.71 (td, *J* = 12.5, 1.8 Hz, 1H), 3.45 (t, *J* = 11.2 Hz, 1H), 3.23 (d, *J* = 17.2 Hz, 1H), 2.97 (dd, *J* = 11.2, 5.6 Hz, 1H), 2.27 (dd, *J* = 17.2, 1.8 Hz, 1H), 1.82 (ddd, *J* = 14.2, 12.5, 5.1 Hz, 1H), 1.45 (dt, *J* = 14.2, 1.8 Hz, 1H), 1.04 (s, 3H). ^{13}C NMR (100 MHz, $CDCl_3$): δ 197.44, 166.26, 141.79, 134.98, 134.57, 131.39, 128.38, 127.36, 69.82, 64.08, 52.68, 46.96, 43.62, 38.18, 33.26, 28.50. IR (film): ν (cm^{-1}) 2953, 1723, 1687, 1436, 1415, 1287, 1261, 1226, 1095. HR-MS (ESI) m/z calcd for $C_{16}H_{19}O_4$ [M+H$^+$] 275.12779, found 275.12779.

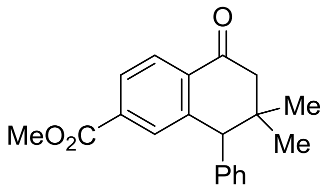

Methyl 7,7-dimethyl-5-oxo-8-phenyl-5,6,7,8-tetrahydronaphthalene-2-carboxylate (**2e**). Colorless oil. ^{1}H NMR (400 MHz, $CDCl_3$): δ 8.18 (d, *J* = 8.2 Hz, 1H), 8.01 – 7.95 (m, 1H), 7.82 – 7.77 (m, 1H), 7.33 – 7.22 (m, 3H), 7.04 – 6.99 (m, 2H), 4.11 – 4.06 (m, 1H), 3.87 (s, 3H), 2.76 (d, *J* =

17.0 Hz, 1H), 2.43 (dd, *J* = 17.0, 0.9 Hz, 1H), 1.12 (s, 3H), 0.89 (s, 3H). ^{13}C NMR (100 MHz, $CDCl_3$): δ 198.07, 166.35, 145.49, 140.17, 135.09, 134.80, 132.10, 130.17, 128.34, 127.83, 127.10, 126.77, 57.07, 52.49, 49.11, 36.76, 29.38, 27.01. IR (film): ν (cm^{-1}) 2970, 1717, 1436, 1360, 1227, 1092, 897. HR-MS (ESI) m/z calcd for $C_{20}H_{21}O_3$ [M+H$^+$] 309.14852, found 309.14835.

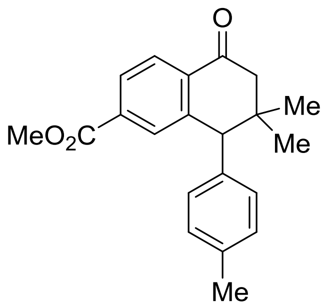

Methyl 7,7-dimethyl-5-oxo-8-(*p*-tolyl)-5,6,7,8-tetrahydronaphthalene-2-carboxylate (**2f**). Colorless oil. ^{1}H NMR (400 MHz, $CDCl_3$): δ 8.15 (d, *J* = 8.2 Hz, 1H), 7.96 (dd, *J* = 8.2, 1.6 Hz, 1H), 7.81 – 7.76 (m, 1H), 7.08 (d, *J* = 7.9 Hz, 2H), 6.88 (d, *J* = 7.9 Hz, 2H), 4.05 – 4.03 (m, 1H), 3.85 (s, 3H), 2.74 (d, *J* = 17.0 Hz, 1H), 2.40 (dd, *J* = 17.0, 0.9 Hz, 1H), 2.31 (s, 3H), 1.09 (s, 3H), 0.88 (s, 3H). ^{13}C NMR (100 MHz, $CDCl_3$): δ 198.24, 166.40, 145.77, 137.14, 136.73, 135.06, 134.79, 132.11, 130.05, 129.05, 127.74, 126.74, 56.72, 52.48, 49.14, 36.74, 29.36, 27.03, 21.11. IR (film): ν (cm^{-1}) 2970, 1719, 1435, 1366, 1230, 1102. HR-MS (ESI) m/z calcd for $C_{21}H_{23}O_3$ [M+H$^+$] 323.16417, found 323.16421.

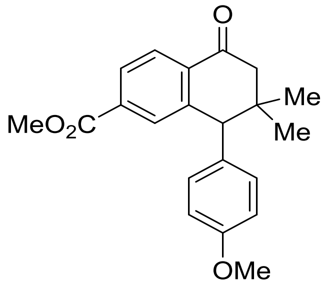

Methyl 8-(4-methoxyphenyl)-7,7-dimethyl-5-oxo-5,6,7,8-tetrahydronaphthalene-2-carboxylate (**2g**). Colorless oil. ^{1}H NMR (400 MHz, $CDCl_3$): δ 8.14 (d, *J* = 8.2 Hz, 1H), 7.99 – 7.93 (m, 1H), 7.79 – 7.76 (m, 1H), 6.94 – 6.88 (m, 2H), 6.85 – 6.78 (m, 2H), 4.04 – 4.01 (m, 1H), 3.85 (s, 3H), 3.78 (s, 3H), 2.72 (d, *J* = 17.0 Hz, 1H), 2.41 (dd, *J* = 17.0, 0.9 Hz, 1H), 1.08 (s, 3H), 0.87 (s, 3H). ^{13}C NMR (100 MHz, $CDCl_3$): δ 198.17, 166.39, 158.65, 145.85, 135.03, 134.77, 132.22, 132.07, 131.14, 127.71, 126.72, 113.74, 56.28, 55.35, 52.48, 49.27, 36.85, 29.31, 26.80. IR (film): ν (cm^{-1}) 2969, 1732, 1687, 1609, 1510, 1367, 1281, 1229, 1203, 1101, 1033. HR-MS (ESI) m/z calcd for $C_{21}H_{23}O_4$ [M+H$^+$] 339.15909, found 339.15919.

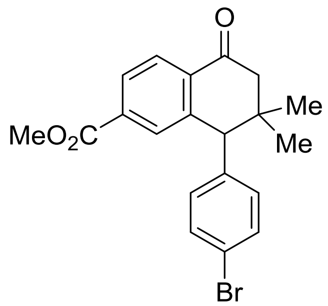

Methyl 8-(4-bromophenyl)-7,7-dimethyl-5-oxo-5,6,7,8-tetrahydronaphthalene-2-carboxylate-2-yl)carbamate (**2h**). Colorless oil. ^{1}H NMR (400 MHz, $CDCl_3$): δ 8.16 (d, *J* = 8.2 Hz, 1H), 8.00 – 7.95 (m, 1H), 7.74 – 7.72 (m, 1H), 7.42 (d, *J* = 8.5 Hz, 2H), 6.89 (d, *J* = 8.5 Hz, 2H), 4.06 – 4.04 (m, 1H), 3.87 (s, 3H), 2.69 (d, *J* = 17.0 Hz, 1H), 2.44 (dd, *J* = 17.0, 0.8 Hz, 1H), 1.09 (s, 3H), 0.87 (s, 3H). ^{13}C NMR (100 MHz, $CDCl_3$): δ 197.66, 166.25, 144.83, 139.24, 135.06, 134.93, 131.90, 131.85, 131.55, 128.08, 126.96, 121.25, 56.52, 52.58, 49.28, 36.77, 29.36, 26.62. IR (film): ν (cm^{-1}) 2970, 1738, 1436, 1370, 1277, 1228, 1216, 1097. HR-MS (ESI) m/z calcd for $C_{20}H_{20}O_3Br$ [$M+H^+$] 387.05903, found 387.05930.

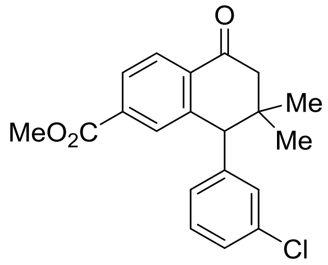

Methyl 8-(3-chlorophenyl)-7,7-dimethyl-5-oxo-5,6,7,8-tetrahydronaphthalene-2-carboxylate (**2i**). Colorless oil. ^{1}H NMR (400 MHz, $CDCl_3$): δ 8.17 (d, *J* = 8.1 Hz, 1H), 8.02 – 7.97 (m, 1H), 7.77 – 7.73 (m, 1H), 7.25 – 7.17 (m, 2H), 7.05 – 6.99 (m, 1H), 6.89 – 6.85 (m, 1H), 4.08 – 4.01 (m, 1H), 3.87 (s, 3H), 2.72 (d, *J* = 17.0 Hz, 1H), 2.44 (dd, *J* = 17.0, 0.8 Hz, 1H), 1.10 (s, 3H), 0.89 (s, 3H). ^{13}C NMR (100 MHz, $CDCl_3$): δ 197.61, 166.25, 144.62, 142.36, 135.00, 134.97, 134.34, 131.97, 130.22, 129.62, 128.27, 128.16, 127.43, 127.00, 56.74, 52.57, 49.08, 36.79, 29.36, 26.93. IR (film): ν (cm^{-1}) 2956, 1724, 1687, 1592, 1570, 1473, 1435, 1409, 1275, 1200, 1163, 1103, 911. HR-MS (ESI) m/z calcd for $C_{20}H_{20}O_3Cl$ [$M+H^+$] 343.10955, found 343.10948.

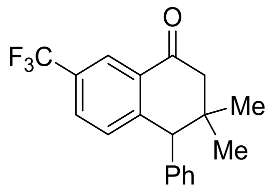

3,3-Dimethyl-4-phenyl-7-(trifluoromethyl)-3,4-dihydronaphthalen-1(2*H*)-one (**2j**). Colorless oil. ^{1}H NMR (400 MHz, $CDCl_3$): δ 8.39 (s, 1H), 7.66 (ddd, *J* = 8.1, 2.1, 0.5 Hz, 1H), 7.34 – 7.27 (m, 3H), 7.22 (dd, *J* = 8.1, 0.5 Hz, 1H), 7.05 – 7.00 (m, 2H), 4.09 (s, 1H), 2.75 (d, *J* = 16.8 Hz, 1H), 2.48 (dd, *J* = 16.8, 0.7 Hz, 1H), 1.11 (s, 3H), 0.90 (s, 3H). ^{13}C NMR (100 MHz, $CDCl_3$): δ 197.22, 149.06, 139.85, 132.61, 131.48, 130.30, 130.14 (q, J_{C3-F} = 3.5 Hz), 129.68 (q, J_{C2-F} = 33.2 Hz), 128.47, 127.34, 123.91 (q, J_{C1-F} = 272.3 Hz), 123.90 (q, J_{C3-F} = 3.8 Hz), 57.17, 49.63, 36.97, 29.47, 26.30.

^{19}F NMR (376 MHz, $CDCl_3$): δ -62.76 (s, 3F). IR (film): ν (cm^{-1}) 2961, 1691, 1619, 1336, 1253, 1165, 1105, 1071. HR-MS (ESI) m/z calcd for $C_{19}H_{18}OF_3$ [$M+H^+$] 319.13043, found 319.13055.

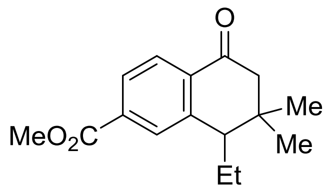

Methyl 8-ethyl-7,7-dimethyl-5-oxo-5,6,7,8-tetrahydronaphthalene-2-carboxylate (**2k**). Colorless oil. ^1H NMR (400 MHz, $CDCl_3$): δ 8.06 (d, *J* = 8.1 Hz, 1H), 7.95 (dd, *J* = 8.1, 1.7 Hz, 1H), 7.88 – 7.86 (m, 1H), 3.94 (s, 3H), 2.72 (dd, *J* = 18.2, 0.7 Hz, 1H), 2.45 (dd, *J* = 10.4, 3.3 Hz, 1H), 2.36 (dd, *J* = 18.2, 1.3 Hz, 1H), 2.03 (dqd, *J* = 13.5, 7.4, 3.3 Hz, 1H), 1.39 – 1.30 (m, 1H), 1.17 (s, 3H), 0.93 (s, 3H), 0.89 (t, *J* = 7.4 Hz, 3H). ^{13}C NMR (100 MHz, $CDCl_3$): δ 198.08, 166.66, 147.29, 134.09, 133.64, 131.60, 127.69, 127.59, 52.57, 52.28, 48.13, 35.70, 29.24, 27.67, 24.92, 13.00. IR (film): ν (cm^{-1}) 2970, 1736, 1725, 1686, 1435, 1365, 1278, 1228, 1215, 1107. HR-MS (ESI) m/z calcd for $C_{16}H_{21}O_3$ [$M+H^+$] 261.14852, found 261.14848.

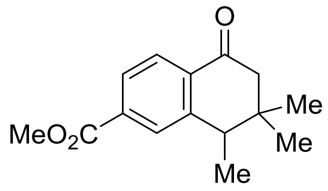

Methyl 7,7,8-trimethyl-5-oxo-5,6,7,8-tetrahydronaphthalene-2-carboxylate (**2l**). Colorless oil. ^1H NMR (400 MHz, $CDCl_3$): δ 8.04 (d, *J* = 8.1 Hz, 1H), 8.01 – 7.97 (m, 1H), 7.92 (dd, *J* = 8.1, 1.6 Hz, 1H), 3.93 (s, 3H), 2.85 (q, *J* = 7.2 Hz, 1H), 2.68 (d, *J* = 17.2 Hz, 1H), 2.41 (dd, *J* = 17.2, 0.8 Hz, 1H), 1.33 (d, *J* = 7.2 Hz, 3H), 1.03 (s, 6H). ^{13}C NMR (100 MHz, $CDCl_3$): δ 197.91, 166.59, 148.70, 134.65, 134.06, 130.28, 127.39, 127.04, 52.55, 49.11, 44.39, 35.62, 28.77, 25.63, 17.59. IR (film): ν (cm^{-1}) 2958, 1723, 1687, 1436, 1270, 1220, 1106. HR-MS (ESI) m/z calcd for $C_{15}H_{19}O_3$ [$M+H^+$] 247.13287, found 247.13268.

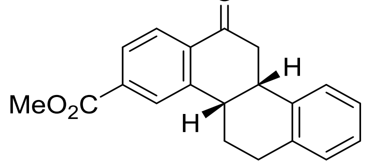

Methyl 12-oxo-4b,5,6,10b,11,12-hexahydrochrysene-3-carboxylate (**2m**). Colorless solid. ^1H NMR (400 MHz, $CDCl_3$): δ 8.12 – 8.08 (m, 2H), 7.98 (dd, *J* = 8.1, 1.6 Hz, 1H), 8.12 – 8.08 (m, 4H), 3.96 (s, 3H), 3.64 (td, *J* = 9.2, 5.1 Hz, 1H), 3.34 (dt, *J* = 11.2, 5.0 Hz, 1H), 3.11 (ddd, *J* = 17.5, 11.2, 6.9 Hz, 1H), 3.00 (ddd, *J* = 17.5, 5.0, 2.8 Hz, 1H), 2.91 – 2.86 (m, 2H), 2.22 – 2.08 (m, 2H). ^{13}C NMR (100 MHz, $CDCl_3$): δ 197.21, 166.45, 147.92, 138.43, 135.30, 134.81, 134.42, 130.89, 129.51, 128.98, 127.81, 127.33, 126.88, 126.48, 52.64, 43.96, 38.77, 37.43, 29.88, 27.11. IR (film): ν (cm^{-1}) 2970, 1719, 1436, 1365, 1229, 1092, 899. HR-MS (ESI) m/z calcd for $C_{20}H_{19}O_3$ [$M+H^+$] 307.13287, found 307.13280.

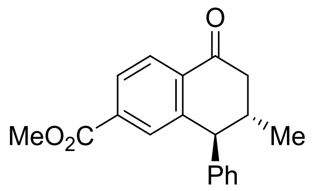

Methyl-7-methyl-5-oxo-8-phenyl-5,6,7,8-tetrahydronaphthalene-2-carboxylate (**2n**). Colorless solid. ^{1}H NMR (400 MHz, $CDCl_3$): δ 8.14 (d, *J* = 8.1 Hz, 1H), 7.98 – 7.92 (m, 1H), 7.58 – 7.56 (m, 1H), 7.37 – 7.24 (m, 3H), 7.11 – 7.06 (m, 2H), 3.93 (d, *J* = 8.0 Hz, 1H), 3.83 (s, 3H), 2.92 – 2.78 (m, 1H), 2.57 – 2.43 (m, 2H), 1.00 (d, *J* = 6.3 Hz, 3H). ^{13}C NMR (100 MHz, $CDCl_3$): δ 197.62, 166.37, 146.21, 142.72, 135.52, 134.53, 131.55, 129.15, 128.95, 127.75, 127.24, 126.91, 53.34, 52.48, 45.41, 37.50, 20.38. IR (film): ν (cm^{-1}) 2970, 1722, 1435, 1370, 1216. HR-MS (ESI) m/z calcd for $C_{19}H_{19}O_3$ [M+H$^+$] 295.13287, found 295.13285.

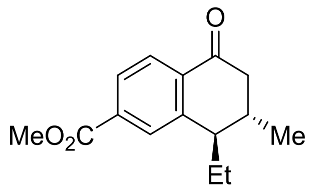

Methyl 8-ethyl-7-methyl-5-oxo-5,6,7,8-tetrahydronaphthalene-2-carboxylate (**2o**). Colorless oil. ^{1}H NMR (400 MHz, $CDCl_3$): δ 8.07 – 8.02 (m, 1H), 7.96 – 7.90 (m, 2H), 3.94 (s, 3H), 2.91 (dd, *J* = 18.3, 5.7 Hz, 1H), 2.66 (td, *J* = 7.0, 3.3 Hz, 1H), 2.49 – 2.38 (m, 2H), 1.89 – 1.78 (m, 1H), 1.78 – 1.67 (m, 1H), 1.02 (d, *J* = 6.8 Hz, 3H), 1.00 (t, *J* = 7.4 Hz, 3H). ^{13}C NMR (100 MHz, $CDCl_3$): δ 197.68, 166.61, 146.51, 134.64, 134.31, 131.20, 127.54, 127.20, 52.56, 47.10, 41.76, 31.41, 28.63, 20.41, 12.04. IR (film): ν (cm^{-1}) 2970, 1726, 1687, 1436, 1365, 1280, 1228, 1201, 1108. HR-MS (ESI) m/z calcd for $C_{15}H_{19}O_3$ [M+H$^+$] 247.13287, found 247.13284.

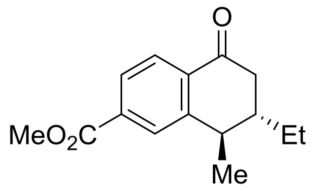

Methyl-7-ethyl-8-methyl-5-oxo-5,6,7,8-tetrahydronaphthalene-2-carboxylate (**2p**). Colorless oil. ^{1}H NMR (400 MHz, $CDCl_3$): δ 8.02 (d, *J* = 8.1 Hz, 1H), 7.99 – 7.97 (m, 1H), 7.91 (dd, *J* = 8.1, 1.6 Hz, 1H), 3.93 (s, 3H), 3.02 (qd, *J* = 7.1, 4.0 Hz, 1H), 2.93 (dd, *J* = 17.2, 4.8 Hz, 1H), 2.54 (ddd, *J* = 17.2, 5.0, 0.7 Hz, 1H), 2.01 – 1.92 (m, 1H), 1.47 – 1.40 (m, 1H), 1.43 (d, *J* = 7.1 Hz, 3H), 1.40 – 1.33 (m, 1H), 0.92 (t, *J* = 7.4 Hz, 3H). ^{13}C NMR (100 MHz, $CDCl_3$): δ 197.75, 166.56, 147.84, 134.62, 134.58, 130.49, 127.42, 127.04, 52.55, 41.84, 39.86, 37.81, 26.69, 21.88, 11.61. IR (film): ν (cm^{-1}) 2961, 1723, 1686, 1436, 1414, 1277, 1195, 1107. HR-MS (ESI) m/z calcd for $C_{15}H_{19}O_3$ [M+H$^+$] 247.13287, found 247.13283.

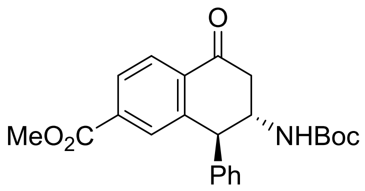

Methyl-7-(*tert*-butoxycarbonylamino)-5-oxo-8-phenyl-5,6,7,8-tetrahydronaphthalene-2-carboxylate (**2q**). Colorless solid. ^{1}H NMR (400 MHz, $CDCl_3$): δ 8.18 (d, *J* = 8.2 Hz, 1H), 8.04 (dd, *J* = 8.2, 1.6 Hz, 1H), 7.83 – 7.81 (m, 1H), 7.34 – 7.24 (m, 3H), 7.10 – 7.04 (m, 2H), 4.76 – 4.74 (m, 1H), 4.58 – 4.59 (m, 1H), 4.41 – 4.39 (m, 1H), 3.88 (s, 3H), 2.92 (dd, *J* = 17.3, 4.1 Hz, 1H), 2.72 (dd, *J* = 17.3, 5.3 Hz, 1H), 1.39 (s, 9H). ^{13}C NMR (100 MHz, $CDCl_3$): δ 195.62, 166.11, 155.05, 142.74, 140.47, 135.37, 135.32, 132.41, 129.00, 128.91, 128.57, 127.54, 127.24, 80.20, 53.62, 52.64, 50.59, 40.93, 28.43. IR (film): ν (cm^{-1}) 3361, 2976, 1688, 1518, 1283, 1241, 1164, 1106. HR-MS (ESI) m/z calcd for $C_{23}H_{25}O_5NNa$ [M+Na^{+}] 418.16249, found 418.16249.

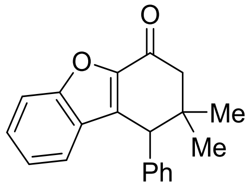

2,2-Dimethyl-1-phenyl-2,3-dihydrodibenzo[*b*,*d*]furan-4(1*H*)-one (**2r**). Colorless oil. ^{1}H NMR (300 MHz, $CDCl_3$): δ 7.57 (d, *J* = 8.4 Hz, 1H), 7.46 – 7.38 (m, 1H), 7.35 – 7.29 (m, 3H), 7.21 – 7.16 (m, 2H), 7.10 (t, *J* = 7.6 Hz, 1H), 6.92 (d, *J* = 7.9 Hz, 1H), 4.20 (s, 1H), 2.75 (d, *J* = 16.5 Hz, 1H), 2.55 (d, *J* = 16.5 Hz, 1H), 1.23 (s, 3H), 0.93 (s, 3H). ^{13}C NMR (75 MHz, $CDCl_3$): δ 188.06, 156.62, 147.65, 137.66, 134.87, 129.93, 128.94, 128.41, 127.66, 126.44, 123.59, 122.99, 112.94, 51.39, 51.33, 40.06, 29.40, 25.27. IR (film): ν (cm^{-1}) 2970, 1736, 1436, 1366, 1228. HR-MS (ESI) m/z calcd for $C_{20}H_{19}O_2$ [M+H^{+}] 291.13796, found 291.13779.

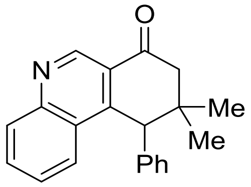

9,9-Dimethyl-10-phenyl-9,10-dihydrophenanthridin-7(8*H*)-one (**2s**). White solid. ^{1}H NMR (300 MHz, $CDCl_3$): δ 9.52 (s, 1H), 8.04 (d, *J* = 8.3 Hz, 1H), 7.88 (d, *J* = 8.3 Hz, 1H), 7.65 (dd, *J* = 8.3, 7.0 Hz, 1H), 7.39 (dd, *J* = 8.3, 7.0 Hz, 1H), 7.23 – 7.10 (m, 3H), 7.06 – 6.97 (m, 2H), 4.53 (s, 1H), 2.76 (d, *J* = 17.4 Hz, 1H), 2.31 (dd, *J* = 17.4, 1.1 Hz, 1H), 1.11 (s, 3H), 0.90 (s, 3H). ^{13}C NMR (75 MHz, $CDCl_3$): δ 198.11, 151.61, 150.50, 148.30, 138.78, 131.59, 130.62, 129.41, 128.76, 127.64, 127.49, 126.09, 125.54, 123.87, 52.81, 46.75, 36.38, 29.18, 29.16. IR (film): ν (cm^{-1}) 2970, 1733, 1435, 1369, 1228. HR-MS (ESI) m/z calcd for $C_{21}H_{20}ON$ [M+H^{+}] 302.15394, found 302.15456.

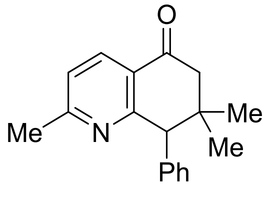

2,7,7-Trimethyl-8-phenyl-7,8-dihydroquinolin-5(6*H*)-one (**2t**). Colorless oil. ^{1}H NMR (400 MHz, $CDCl_3$): δ 8.26 (d, *J* = 8.0 Hz, 1H), 7.29 – 7.19 (m, 3H), 7.16 (d, *J* = 8.0 Hz, 1H), 7.00 – 6.95 (m, 2H), 4.15 (s, 1H), 2.74 (dd, *J* = 17.5, 0.8 Hz, 1H), 2.51 (s, 3H), 2.32 (dd, *J* = 17.5, 1.4 Hz, 1H), 1.15 (s, 3H), 0.88 (s, 3H). ^{13}C NMR (100 MHz, $CDCl_3$): δ 198.31, 164.63, 163.91, 139.56, 134.82, 129.99, 128.27, 126.86, 125.39, 122.53, 59.68, 47.48, 35.90, 29.23, 28.32, 25.26. IR (film): ν (cm^{-1}) 2959, 1683, 1587, 1452, 1387, 1295, 1268, 1115. HR-MS (ESI) m/z calcd for $C_{18}H_{20}ON$ [$M+H^+$] 266.15394, found 266.15437.

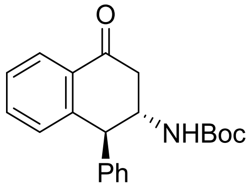

***tert*-Butyl ((*trans*)-4-oxo-1-phenyl-1,2,3,4-tetrahydronaphthalen-2-yl)carbamate** (**3**). Colorless solid. ^{1}H NMR (400 MHz, $CDCl_3$): δ 8.14 (dd, *J* = 7.6, 1.4 Hz, 1H), 7.53 (td, *J* = 7.6, 1.4 Hz, 1H), 7.42 (td, *J* = 7.6, 1.0 Hz, 1H), 7.34 – 7.23 (m, 3H), 7.14 – 7.08 (m, 3H), 4.78 – 4.76 (m, 1H), 4.49 - 4.47 (m, 1H), 4.42 – 4.40 (m, 1H), 2.92 (dd, *J* = 17.2, 4.1 Hz, 1H), 2.70 (ddd, *J* = 17.2, 6.1, 0.7 Hz, 1H), 1.39 (s, 9H). ^{13}C NMR (100 MHz, $CDCl_3$): δ 196.06, 155.07, 142.82, 140.97, 134.62, 132.57, 130.92, 129.01, 128.85, 127.78, 127.33, 127.07, 80.01, 53.59, 50.84, 41.30, 28.42. IR (film): ν (cm^{-1}) 3361, 2977, 1683, 1599, 1514, 1453, 1366, 1252, 1166, 1044. HR-MS (ESI) m/z calcd for $C_{21}H_{23}O_3NNa$ [$M+Na^+$] 360.15701, found 360.15695.

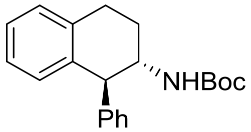

***tert*-Butyl ((*trans*)-1-phenyl-1,2,3,4-tetrahydronaphthalen-2-yl)carbamate** (**S6**). Synthesized according to Samec *et al.*.[4] $NaBH_4$ (5 mg, 0.13 mmol) was added to a solution of ketone **3** (34 mg, 0.1 mmol) in THF/MeOH (10:1) (3 mL) and the mixture was stirred at 25 °C for 2 h. The reaction mixture was quenched with water and extracted with EtOAc. The combined organic extracts were dried over $MgSO_4$ and concentrated under reduced pressure. The residue was dissolved in MeOH (2.5 mL) and 10% Pd/C (2 mg, 0.01 mmol) and HCO_2NH_4 (6 mg, 0.1 mmol) were added followed by H_2O (0.6 mL) and HCO_2H (18 mg, 0.4 mmol). The mixture was stirred at 80 ºC for 12 h and was allowed to warm to 25 ºC. The solvent was removed under reduced pressure and the residue was filtered through Celite with EtOAc. Further evaporation of the solvent afforded amine **S6** as a white solid (26 mg, 81 %). White solid. ^{1}H NMR (400 MHz, $CDCl_3$): δ 7.30 – 7.24 (m, 2H), 7.24 – 7.16 (m, 3H), 7.12 – 7.05 (m, 3H), 6.87 (d, *J* = 7.7 Hz, 1H), 4.76 – 4.54 (m, 1H), 4.16 – 3.98 (m, 2H), 3.00 (dt, *J* = 17.4, 6.4 Hz, 1H), 2.90 (ddd, *J* = 17.4, 7.8, 6.2 Hz, 1H), 2.13 – 2.01 (m, 1H), 1.86 – 1.71 (m, 1H), 1.39 (s, 9H). ^{13}C NMR (100 MHz, $CDCl_3$): δ 155.44, 144.30, 136.86, 136.17, 131.25, 129.41, 128.91, 128.43, 126.61, 126.59, 126.32, 79.42, 52.88, 51.73, 28.50, 26.26, 25.11. IR (film): ν (cm^{-1}) 3360, 2930, 1699, 1492, 1452, 1365, 1234, 1170. HR-MS (ESI) m/z calcd for $C_{21}H_{25}O_2NNa$ [$M+Na^+$] 346.17775, found 346.17770.

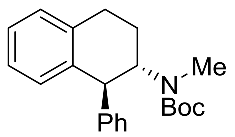

***tert*-Butyl methyl((*trans*)-1-phenyl-1,2,3,4-tetrahydronaphthalen-2-yl)carbamate** (**S7**). Synthesized according to Zacuto *et al.*.[5] A 60% dispersion of NaH in mineral oil (8 mg, 0.2 mmol) was added in one portion to a solution of amine **S6** (32 mg, 0.1 mmol) in DMF (5 mL) at 0 °C and the mixture was stirred for 10 min. MeI (43 mg, 0.3 mmol) was added and the reaction was allowed to warm to 25 °C and stirred for further 16 h. The reaction mixture was quenched with water and extracted three times with EtOAc. The combined organic extracts were dried over $MgSO_4$ and concentrated under reduced pressure. The residue was then purified by flash column chromatography (hexane/EtOAc, 7:1) affording methyl amine **S7** as a colorless oil (31 mg, 92 %). ^1H NMR (400 MHz, $CDCl_3$): δ 7.28 - 6.86 (m, 8H), 6.70 - 6.63 (m, 1H), 4.58 - 4.29 (m, 1H), 4.06 (d, *J* = 10.8 Hz, 1H), 3.19 - 3.00 (m, 1H), 2.95 - 2.86 (m, 1H), 2.77 - 2.63 (m, 3H), 2.11 - 1.85 (m, 2H), 1.19 - 1.05 (m, 9H). ^{13}C NMR (100 MHz, $CDCl_3$): δ 155.43, 144.04, 139.61, 136.24, 130.63, 129.65, 129.18, 128.51, 126.67, 126.15, 126.05, 79.35, 59.42, 58.13, 49.30, 30.30, 28.30, 28.19. IR (film): ν (cm-1) 3016, 2945, 1738, 1437, 1365, 1228, 1217. HR-MS (ESI) m/z calcd for [M+Na$^+$], 360.19340 found 360.19288.

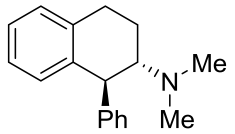

(*trans*)-*N*,*N*-Dimethyl-1-phenyl-1,2,3,4-tetrahydronaphthalen-2-amine (**4**). Synthesized according to Zacuto *et al.*.[5] $LiAlH_4$ (10 mg, 0.25 mmol) was added in one portion to a solution of carbamate **S7** (16.6 mg, 0.05 mmol) in THF (4 mL) and the mixture was stirred at reflux temperature for 3 days. The reaction mixture was quenched with a saturated Rochelle salt solution and was extracted three times with EtOAc. The combined organic phases were dried over $MgSO_4$ and concentrated under reduced pressure. The residue was then purified by flash column chromatography (EtOAc) affording dimethyl amine **4** as a colorless oil (10.4 mg, 83 %).^1H NMR (400 MHz, $CDCl_3$): δ 7.40 – 7.01 (m, 8H), 6.90 – 6.76 (m, 1H), 4.33 – 4.21 (m, 1H), 3.07 – 2.77 (m, 3H), 2.52 – 2.27 (m, 6H), 2.12 – 1.95 (m, 1H), 1.92 – 1.74 (m, 1H). ^{13}C NMR (100 MHz, $CDCl_3$): δ 146.53, 138.93, 137.16, 130.87, 129.40, 128.60, 128.45, 128.28, 126.12, 125.99, 67.60, 48.59, 42.14, 28.02, 21.31. IR (film): ν (cm^{-1}) 2970, 1448, 1366, 1228, 1217. HR-MS (ESI) *m/z* calcd for $C_{18}H_{22}N$ [M+H$^+$] 252.17468, found 252.17471.

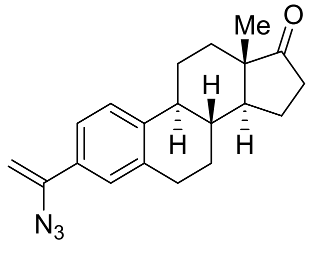

(8*R*,9*S*,13*S*,14*S*)-3-(1-Azidovinyl)-13-methyl-6,7,8,9,11,12,13,14,15,16-decahydro-17*H*-cyclopenta[*a*]phenanthren-17-one (**5**). This compound was synthesized from 3-ethyn-1-yl-estra-1,3,5(10)-trien-17-one according to the procedure described by Bi *et al.*[1] White solid. ^1H

NMR (400 MHz, acetone-d_6): δ 7.42 - 7.30 (m 3H), 5.57 (d, J = 1.9 Hz, 1H), 4.98 (d, J = 1.9 Hz, 1H), 3.00 – 2.92 (m, 2H), 2.87 – 2.78 (m, 2H), 2.54 – 2.43 (m, 2H), 2.41 – 2.31 (m, 1H), 2.17 – 2.10 (m, 1H), 1.95 – 1.86 (m, 1H), 1.75 – 1.45 (m, 6H), 0.91 (s, 3H). ^{13}C NMR (100 MHz, $CDCl_3$): δ 220.88, 145.06, 141.11, 136.78, 132.00, 126.26, 125.64, 123.14, 97.56, 50.65, 48.10, 44.58, 38.19, 35.99, 31.71, 29.56, 26.56, 25.81, 21.73, 13.98. IR (film): ν (cm^{-1}) 2932, 2870, 2097, 1732, 1600, 1505, 1406, 1302, 1275, 1254, 1080, 1051. HR-MS (ESI) *m/z* calcd for $C_{20}H_{23}N_3ONa$ [M+Na$^+$] 344.17333, found 344.17321.

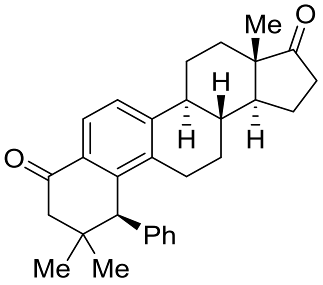

(3a*S*,5a*S*,11*R*,13a*R*,13b*S*)-3a,10,10-Trimethyl-11-phenyl-1,3a,4,5,5a,9,10,11,12,13,13a,13b-dodecahydro-3*H*-cyclopenta[*a*]chrysene-3,8(2*H*)-dione (**6**). This compound was synthesized from vinyl azide **5** using the standard conditions (Table 1 entry 12). White solid. ^{1}H NMR (400 MHz, $CDCl_3$): δ 8.01 (d, J = 8.3 Hz, 1H), 7.37 (d, J = 8.3 Hz, 1H), 7.27 - 7.20 (m, 3H), 7.03 - 6.96 (m, 2H), 4.02 (s, 1H), 2.97 (dd, J = 17.2, 4.7 Hz, 1H), 2.68 (dd, J = 17.4, 0.8 Hz, 1H), 2.51 – 2.39 (m, 2H), 2.36 – 2.27 (m, 1H), 2.22 (dd, J = 17.4, 1.3 Hz, 1H), 2.17 – 2.05 (m, 2H), 2.04 – 1.88 (m, 3H), 1.56 – 1.32 (m, 6H), 1.13 (s, 3H), 0.87 (s, 3H), 0.82 (s, 3H).^{13}C NMR (100 MHz, $CDCl_3$): δ 220.60, 199.09, 146.96, 143.31, 139.38, 135.43, 130.35, 130.28, 128.14, 126.65, 124.27, 124.23, 53.49, 50.77, 47.84, 46.64, 45.28, 36.76, 36.05, 35.94, 31.71, 29.39, 29.35, 26.55, 26.14, 25.75, 21.59, 13.92. IR (film): ν (cm^{-1}) 2931, 1738, 1678, 1588, 1453, 1410, 1292, 1255, 1030. HR-MS (ESI) *m/z* calcd for $C_{30}H_{35}O_2$ [M+H$^+$] 427.26316, found 427.26314.

4. Control Experiments

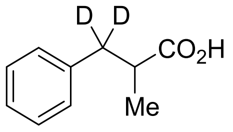

2-Methyl-3-phenylpropanoic-3,3-*d₂* acid (*d₂*-8). *tert*-Butyl propanoate (2.6 g, 20 mmol) was added dropwise to a solution of LDA (20 mmol) in THF (50 mL) at – 78 °C. After stirring for 30 min, d_2-BnBr (4.94 mL, 30 mmol) was added dropwise and the resulting solution was allowed to warm to 25 °C over 2 h. The reaction was quenched by the addition of pH = 7 phosphate buffer solution and brine and the mixture was extracted with Et_2O. The combined organic extracts were dried over $MgSO_4$ and concentrated in vacuo. The residue was dissolved in CH_2Cl_2 (10 mL) and TFA (10 mL) was added. The solution was warmed to 30 °C and then allowed to stand for 30 min. The reaction mixture was concentrated in vacuo and the residue was dissolved in 1 M aq. NaOH. The solution was washed with Et_2O and acidified to pH = 2 by the addition of conc. aq. HCl. The mixture was extracted with Et_2O and the combined organic extracts were dried over $MgSO_4$ and concentrated under reduced pressure. The residue was then purified by flash column chromatography (hexane/EtOAc, 3:1) affording deuterated acid ***d_2-8*** as a colorless oil (2.82 g, 85 %). ^{1}H NMR (400 MHz, $CDCl_3$): δ 7.33 – 7.28 (m, 2H), 7.25 – 7.18 (m, 3H), 2.77 (q, J = 7.0 Hz, 1H),

1.19 (d, J = 7.0 Hz, 3H). ^{13}C NMR (100 MHz, $CDCl_3$): δ 182.74, 139.08, 129.13, 128.56, 126.57, 41.24, 38.72 (quint, J = 19.6 Hz), 16.54. IR (film): ν (cm^{-1}) 2970, 1738, 1448, 1368, 1228, 1216, 911. HR-MS (ESI) m/z calcd for $C_{10}H_9D_2O_2$ [M-H$^-$] 165.08901, found 165.08885.

Kinetic Isotope Effect experiments

The KIE experiments were carried out in two sets of competition reactions, i.e. 1) intramolecular KIE experiment and 2) intermolecular KIE experiment. The rate of H/D was calculated based on the ratio of regular product **2l** and deuterated **2l** (*d*-**2l**) determined by ^{1}H NMR and mass spectroscopy.

1) Intermolecular KIE experiment:

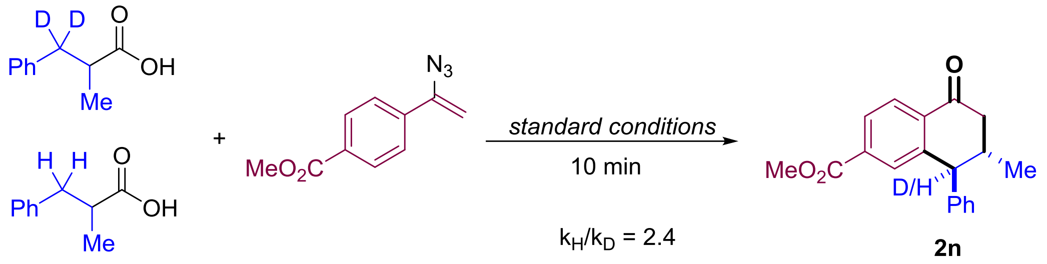

8 : ***d₂*-8** = 1:1

Methyl 4-(1-azidovinyl)benzoate (61 mg, 0.3 mmol), 3,3-d_2-2-methyl-3-phenylpropanoic acid d_2-**8** (16.6 mg, 0.1 mmol), 2-methyl-3-phenylpropanoic acid **8** (16.4 mg, 0.1 mmol), Ag_2CO_3 (16.8 mg, 0.06 mmol) and $K_2S_2O_8$ (108 mg, 0.4 mmol) were placed in a dry Schlenk-tube. The reaction vessel was evacuated and filled with nitrogen for three times. Acetonitrile (0.5 mL), acetone (0.2 mL), distilled water (1.5 mL) and 2,6-lutidine (25.8 mg, 0.24 mmol) were sequentially added. The resulting mixture was stirred at 50°C for 10 min. The reaction was cooled to room temperature and diluted with water (10 ml) and extracted with EtOAc (15 mL). The organic layer was washed with brine (10 mL), dried over anhydrous $MgSO_4$, filtered and concentrated under reduced pressure. The crude product was purified by column chromatography on silica gel (hexane/ethyl acetate, 5:1) to afford the desired product **2n** as a colorless oil (13 mg). ^{1}H NMR (400 MHz, $CDCl_3$): δ 8.14 (d, J = 8.1 Hz, 1H), 7.98 – 7.92 (m, 1H), 7.58 – 7.56 (m, 1H), 7.37 – 7.24 (m, 3H), 7.11 – 7.06 (m, 2H), 3.93 (d, J = 8.0 Hz, 0.69H), 3.83 (s, 3H), 2.92 – 2.78 (m, 1H), 2.57 – 2.43 (m, 2H), 1.01 (d, J = 6.3 Hz, 3H). A k_H/k_D = 2.4 could be determined (See Supplementary Figure 45).

2) Intramolecular KIE experiment:

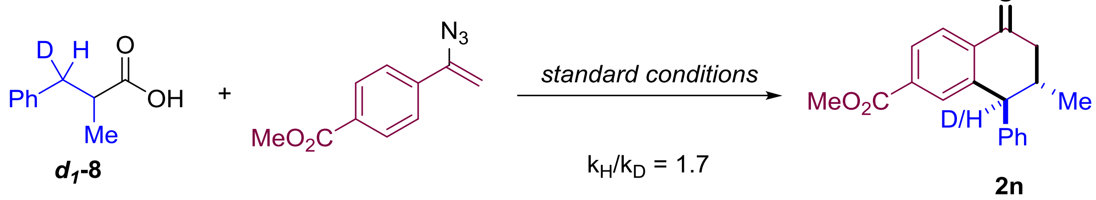

***d₁*-8**

Methyl 4-(1-azidovinyl)benzoate (61 mg, 0.3 mmol), 3-*d*-2-methyl-3-phenylpropanoic acid d_1-**8** (33 mg, 0.2 mmol), Ag_2CO_3 (16.8 mg, 0.06 mmol) and $K_2S_2O_8$ (108 mg, 0.4 mmol) were placed in a dry Schlenk-tube. The reaction vessel was evacuated and filled with nitrogen for three times. Acetonitrile (0.5 mL), acetone (0.2 mL), distilled water (1.5 mL) and 2,6-lutidine (25.8 mg, 0.24 mmol) were sequentially added. The resulting mixture was stirred at 50°C for 10 h. The reaction was cooled to room temperature and diluted with water (10 ml) and extracted with EtOAc (15

mL). The organic layer was washed with brine (10 mL), dried over anhydrous $MgSO_4$, filtered and concentrated under reduced pressure. The crude product was purified by column chromatography on silica gel (hexane/ethyl acetate, 5:1) to afford the desired product **2n** as a colorless oil (45 mg). ^{1}H NMR (400 MHz, $CDCl_3$): δ 8.14 (d, *J* = 8.1 Hz, 1H), 7.98 – 7.92 (m, 1H), 7.58 – 7.56 (m, 1H), 7.37 – 7.24 (m, 3H), 7.11 – 7.06 (m, 2H), 3.93 (d, *J* = 8.4 Hz, 0.38H), 3.83 (s, 3H), 2.92 – 2.78 (m, 1H), 2.57 – 2.43 (m, 2H), 1.01 (d, *J* = 6.3 Hz, 3H). A k_H/k_D = 1.7 was determined (See Supplementary Figure 46).

5. Reactions of internal vinyl azides with primary, secondary and tertiary carboxylic acids

Vinyl azide (0.3 mmol, 1.5 equiv), carboxylic acid (0.2 mmol, 1.0 equiv), Ag_2CO_3 (0.06 mmol, 0.3 equiv) and $K_2S_2O_8$ (0.4 mmol, 2.0 equiv) were placed in a dry Schlenk-tube. The reaction vessel was evacuated and filled with nitrogen three times. Acetonitrile (0.5 mL), acetone (0.2 mL), distilled water (1.5 mL) and 2,6-lutidine (0.24 mmol, 1.2 equiv) were sequentially added at 25 °C. The reaction mixture was stirred at 50°C or 80°C for 10 h. The resulting mixture was extracted with EtOAc (15 mL) and the organic layer was washed with brine (10 mL), dried over anhydrous $MgSO_4$, filtered and concentrated under reduced pressure. The crude was analyzed by ^{1}H-NMR but no significant amount of product was detected.

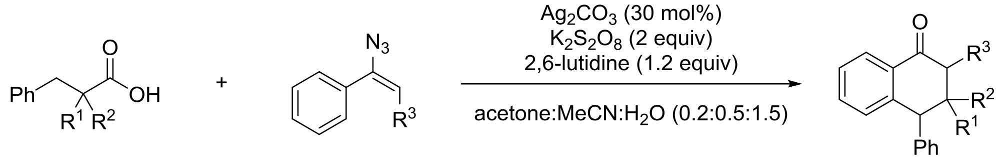

Acid 1 R^1 = H; R^2 = H
Acid 2 R^1 = H; R^2 = Me
Acid 3 R^1 = Me; R^2 = Me

R^3 = Me, Ph, Cyclopropyl

6. Computational Study

All reported structures were optimized at Density Functional Theory level by using the unrestricted M06-2X[6] functional as implemented in Gaussian 09.[7] Optimizations were carried out by using the 6-311++G(d,p) basis set for all atoms, in a solvent model (IEFPCM, solvent = acetone).[8–10] Reported energy values correspond to Gibbs Free (G) energies. The critical stationary points were characterized by frequency calculations in order to verify that they have the right number of imaginary frequencies, and the intrinsic reaction coordinates (IRC)[11] were followed to verify the energy profiles connecting the key transition structures to the correct associated local minima.

The transition states for the decarboxylation ($\mathbf{TS}_{\text{I-II}}$) and N_2 fragmentation ($\mathbf{TS}_{\text{III-IV}}$) could not be located. A relaxed PES scan was performed, varying the C-C or N-N distances at 0.05 Å steps. Both transition states were found to be extremely early, at distances of 1.55 Å and 1.40 Å, showing E energies of only ca. 2.5 kcal/mol above the corresponding starting materials. Taking into consideration that, in both cases, the reaction is highly exergonic due to the formation of two fragments from a single molecule, we consider that the $\Delta G^{\ddagger}$ can be safely predicted to be lower than 5 kcal/mol (See Supplementary Table 23).

Supplementary References

1. Liu, Z., Liao, P. & Bi, X. General silver-catalyzed hydroazidation of terminal alkynes by combining TMS-N_3 and H_2O: Synthesis of vinyl azides. *Org. Lett.* **16,** 3668–3671 (2014).

2. Wang, Y. F., Toh, K. K., Lee, J. Y. & Chiba, S. Synthesis of isoquinolines from α-aryl vinyl azides and internal alkynes by Rh-Cu bimetallic cooperation. *Angew. Chemie Int. Ed.* **50,** 5927–5931 (2011).

3. Fowler, F. W., Hassner, A. & Levy, L. A. Stereospecific Introduction of Azide Functions into Organic Molecules. *J. Am. Chem. Soc.* **89,** 2077–2082 (1967).

4. Sawadjoon, S., Lundstedt, A. & Samec, J. S. M. Pd-catalyzed transfer hydrogenolysis of primary, secondary, and tertiary benzylic alcohols by formic acid: A mechanistic study. *ACS Catal.* **3,** 635–642 (2013).

5. Zacuto, M. J., Tomita, D., Pirzada, Z. & Xu, F. Chemoselectivity of the Ru-catalyzed cycloisomerization reaction for the synthesis of dihydropyrans; application to the synthesis of L-forosamine. *Org. Lett.* **12,** 684–687 (2010).

6. Zhao, Y. & Truhlar, D. G. The M06 suite of density functionals for main group thermochemistry, thermochemical kinetics, noncovalent interactions, excited states, and transition elements: Two new functionals and systematic testing of four M06-class functionals and 12 other functionals. *Theor. Chem. Acc.* **120,** 215–241 (2008).

7. Gaussian 09, Revision D.01; M. J. Frisch, G. W. Trucks, H. B. Schlegel, G. E. Scuseria, M. A. Robb, J. R. Cheeseman, G. Scalmani, V. Barone, B. Mennucci, G. A. Petersson, H. Nakatsuji, M. Caricato, X. Li, H. P. Hratchian, A. F. Izmaylov, J. Bloino, G. Zheng, J. L. Sonnenberg, M. Hada, M. Ehara, K. Toyota, R. Fukuda, J. Hasegawa, M. Ishida, T. Nakajima, Y. Honda, O. Kitao, H. Nakai, T. Vreven, J. A. Montgomery, Jr., J. E. Peralta, F. Ogliaro, M. Bearpark, J. J. Heyd, E. Brothers, K. N. Kudin, V. N. Staroverov, T. Keith, R. Kobayashi, J. Normand, K. Raghavachari, A. Rendell, J. C. Burant, S. S. Iyengar, J. Tomasi, M. Cossi, N. Rega, J. M. Millam, M. Klene, J. E. Knox, J. B. Cross, V. Bakken, C. Adamo, J. Jaramillo, R. Gomperts, R. E. Stratmann, O. Yazyev, A. J. Austin, R. Cammi, C. Pomelli, J. W. Ochterski, R. L. Martin, K. Morokuma, V. G. Zakrzewski, G. A. Voth, P. Salvador, J. J. Dannenberg, S. Dapprich, A. D. Daniels, O. Farkas, J. B. Foresman, J. V. Ortiz, J. Cioslowski, and D. J. Fox, Gaussian, Inc., Wallingford CT, (2013).

8. Cancès, E., Mennucci, B. & Tomasi, J. A new integral equation formalism for the polarizable continuum model: Theoretical background and applications to isotropic and anisotropic dielectrics. *J. Chem. Phys.* **107,** 3032–3047 (1997).

9. Cossi, M., Barone, V., Mennucci, B. & Tomasi, J. Ab initio study of ionic solutions by a polarizable continuum dielectric model. *Chem. Phys. Lett.* **286,** 253–260 (1998).

10. Tomasi, J., Mennucci, B. & Cancès, E. The IEF version of the PCM solvation method: An overview of a new method addressed to study molecular solutes at the QM ab initio level. *J. Mol. Struct. THEOCHEM* **464,** 211–226 (1999).

11. Gonzalez, C. & Schlegel, H. B. Reaction path following in mass-weighted internal coordinates. *J. Phys. Chem.* **94,** 5523–5527 (1990).
